# *Metarhizium* fight club: Within-host competitive exclusion and resource partitioning

**DOI:** 10.1371/journal.ppat.1012639

**Published:** 2024-11-07

**Authors:** Huiyu Sheng, Raymond J. St. Leger

**Affiliations:** Department of Entomology, University of Maryland, College Park, Maryland, United States of America; University of Melbourne, AUSTRALIA

## Abstract

Both *Metarhizium robertsii* ARSEF 2575 (Mr2575) and *Metarhizium anisopliae* ARSEF 549 (Ma549) infect a range of insects whilst also interacting with plants; however, little is known about the traits that affect the competitive ability of different strains. We examined the interactions between Mr2575 and Ma549 in culture and during co-infection of plants (*Arabidopsis thaliana*) and insects. Mr2575 outcompetes Ma549 under nutrient-limiting conditions, including root exudates, giving it a priority advantage on *Arabidopsis* roots. However, during co-infection of *Manduca sexta* or *Drosophila melanogaster*, Ma549’s higher blastospore production enhanced its competitive ability within the host. In large *M*. *sexta* (fifth instar), blastospores facilitate dispersal, suppress host melanization and prevent Mr2575 from spreading from infection sites, reducing conidia production. However, colonization of smaller hosts such as first instar *M*. *sexta* and *D*. *melanogaster* did not provide Ma549 with a competitive advantage, as conidial production was dependent on retaining control of the cuticle through which conidiating hyphae emerge. Unexpectedly, Ma549 and Mr2575 segregate within hosts, suggesting resource partitioning with Mr2575 predominating in the thoraxes of *Drosophila*, especially in females, and Ma549 in the abdomen. In fifth instar *M*. *sexta*, Mr2575 was most prevalent around spiracles and the front end of segments, despite Ma549 and Mr2575 having similar susceptibility to hypoxia. Dispersing conidia homogeneously into the hemocoel of fifth instar *M*. *sexta* eliminated the blastospore production advantage, making Ma549 and Mr2575 equally competitive, with strict partitioning of Mr2575 at the anterior and Ma549 at the posterior ends of segments. As *Metarhizium* species have multiple roles in natural ecosystems and agroecosystems these discoveries are relevant to understanding their impact on maintaining biodiversity and for exploiting them to enhance food security.

## Author summary

*Metarhizium* spp. are keystone fungi providing essential ecosystem services as plant symbionts and insect pathogens, yet little is known about how different strains interact. We analyzed two strains with different insect-killing strategies: Mr2575 kills insects with toxins before colonizing cadavers, whereas Ma549 rapidly colonizes living insects. Ma549’s rapid growth within insects serves as a strong anti-competitive strategy in larger insects. However, in smaller insects, Mr2575 remains competitive in reproductive potential (spore production on cadavers) if it maintains control of the cuticle through which it entered the insect. Consequently, small hosts may be more critical than large hosts for preserving pathogen diversity. Unexpectedly, Mr2575 and Ma549 partition insect hosts between them, promoting coexistence. We also investigated trade-offs between virulence traits and plant colonization traits, finding that Mr2575’s rapid growth in sparse nutrients enhances its competitiveness on plant roots. Thus, Mr2575 may be more competitive on plants, while Ma549 excels in large insects, with both strains showing similar competitiveness in small insects. These insights are important for understanding *Metarhizium* species’ roles in biodiversity and their potential to boost food security in natural and agricultural ecosystems.

## Introduction

*Metarhizium* spp. are keystone taxa with crucial roles in natural ecosystems and that benefit agroecosystems by acting as decomposers, insect pathogens and plant growth promoters [1,2,3]. Genomic studies suggest that the very common PARB clade (*Metarhizium* *p**ingshaense*, *Metarhizium* *a**nisopliae*, *Metarhizium* *r**obertsii*, and *Metarhizium* *b**runneum*) of plant associating broad-host range entomopathogenic fungi could have evolved from a narrow host range lineage that left Asia ∼15 million years ago [4], coinciding with the appearance of a latitudinal biodiversity gradient [5]. Briefly, *Metarhizium* spp. may have evolved to colonize plants in seasonal environments to improve their survival when insects are rare, with a facultative broad host range that increases access to resources when insects are common [6]. This strategy works for *M*. *robertsii* strain ARSEF2575 (Mr2575); a root-colonizing, non-insect pathogenic mutant of Mr2575 survived better in grassland soils and crop plants than an insect pathogenic mutant unable to adhere to root surfaces [7]. Furthermore, *Metarhizium* overwinters more successfully on the dead roots of annual plants than it does in bare soil [8]. Like Mr2575, *M*. *anisopliae* ARSEF 549 (Ma549) is commonly used to study *Metarhizium* infection strategies, and at least under laboratory conditions, Ma549 colonizes the roots and improves the growth of industrial hemp *Cannabis sativa* in a fashion similar to Mr2575 [9]. Being generalists that infect multiple host species should increase the likelihood of PARB clade strains encountering potential hosts and thus participate in co-infections. However, empirical studies of co-infecting insects with multiple fungal strains suggest a pattern of competitive exclusion, with no evidence of synergistic effects between strains [10]. To date, the critical factors that determine the outcome of mixed infections, and their relationship and potential trade-offs with factors promoting plant colonization remain unclear.

*Metarhizium* spp. have evolved numerous mechanisms that enable them to attack, parasitize, and gain nutrients from insects [11]. However, studies with different *Metarhizium* strains, especially Mr2575 and Ma549, have shown that they all produce appressorial infection structures and a broad range of lytic enzymes to invade insects by direct penetration of the cuticle [6]. Genetic studies have helped characterize *Drosophila melanogaster* host responses to Ma549 [12], and *D*. *melanogaster* has been used to evaluate the relative importance of pathogen traits, including host range, speed of growth, and metabolic flexibility, as well as their impact on pathogen evolution [13]. However, to reveal the biochemical mechanisms underlying the response of insects to different pathogens, it is useful to use larger insects than *Drosophila*. The larvae of the tobacco hornworm, *Manduca sexta*, are considered a significant agricultural pest and are also relatively large and easy to manipulate. Mr2575 and Ma549 have been compared in *M*. *sexta* in order to model infection processes [14]. Although Ma549 kills diverse insect hosts, it resembles most narrow-host range entomopathogens by having biotrophic characteristics, and it colonizes still living *M*. *sexta* through blastospores (a yeast-like phase). In contrast, Mr2575 is a model for most broad-spectrum pathogens as it has necrotrophic characteristics, killing *M*. *sexta* with various broad-spectrum destruxin toxins (principally destruxin A), and only then colonizing cadavers [14]. Studies on plant pathogens suggest that necrotrophic broad host range characteristics favor colonization of new environments [15].

In this study, we aimed to identify traits that affect the competitive ability of these fungi on plants and different insects. To this end, we visualized the colonization of *Arabidopsis thaliana* roots and determined whether the competitive abilities of Ma549 and Mr2575 were influenced by host genotype and phenotype by co-inoculating Ma549 and Mr2575 onto insect hosts *M*. *sexta*, *D*. *melanogaster*, and *Sarcophaga bullata* (a dipteran intermediate in size between *Drosophila* and *Manduca*). We investigated differences in both the qualitative and quantitative outcomes of single and mixed infections by assessing multiple traits, including virulence and spore production. We then used this data to investigate the extent to which competition between strains is influenced by host phenotype, pathogen infection strategies and alternative habitat options on plants.

## Results

### In vitro characterization of Mr2575 and Ma549

**Fig 1:**
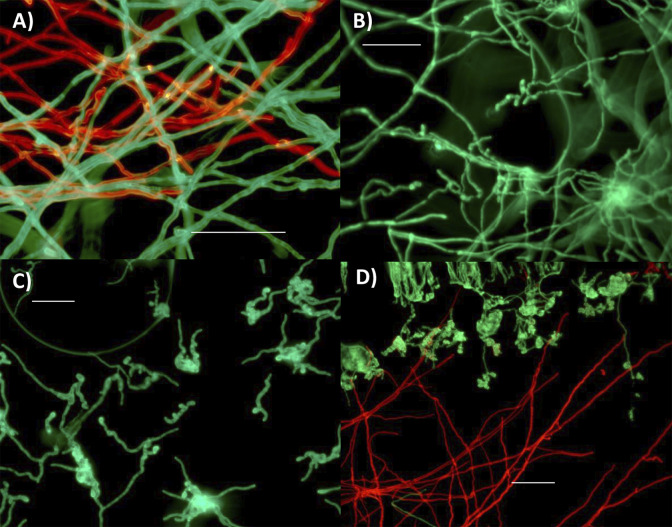
Morphological observation of Mr2575 and Ma549. A) Coexistence of Mr2575-Cherry and Ma549-GFP with hyphae growing over each other 24 h post-inoculation in a petri dish containing 0.0125% YE. B) Mr2575-GFP and C) Ma549-GFP 14 h post inoculation in petri dishes containing a dichloromethane extract of *M*. *sexta* fifth instar cuticular lipids. D. Mr2575-Cherry and Ma549-GFP growing against a dry cover slip (coverslip cultures, see methods) sitting on blocks of agar inoculated with either Mr2575-Cherry or Ma549-GFP. The agar blocks contained 5 mg/ml *M*. *sexta* cuticle extract. Panels A and D represent image overlays of the respective GFP and Cherry images shown in S4Ai and S4Aii Fig. Bar = 20 μm.

Given the extensive period of the life cycle spent outside insect hosts we first assessed the interactions of Mr2575 and Ma549 in culture media, investigating several life history traits including growth rates. We co-inoculated Ma549 and Mr2575 separately and together in nutrient-rich liquid cultures to achieve homologous mixing and effectively create a single population, and on agar which can be used to model microbial coexistence in hosts as it provides spatial structure allowing the strains to dominate in different locations as they might do in a caterpillar [16]. Both Ma549 and Mr2575 grew as a mixture of hyphal pellets and single-celled blastospores when cultured separately in SDB medium. However, by 60 h post-inoculation Mr2575 had produced 1.67-fold more single-celled blastospores than Ma549 (60.33 ± 3.67 blastospores per μl versus 36.2 ± 2.67, t = 5.32, p < 0.00001, n = 15) (Table A in S1 Data). Co-culturing Ma549 and Mr2575 reduced Mr2575 and Ma549 blastospore counts by 9% (t = 1.188, p < 0.05) and 16% (t = 1.643, p < 0.05) respectively, suggesting that inter-strain interactions in SDB did not significantly reduce production of blastospores, and Mr2575 still produced significantly more blastospores than Ma549 in co-cultures (54.93 ± 2.54 blastospores per μl versus 30.47 ± 2.253, t = 6.985, p < 0.00001, n = 15) (Table A in S1 Data). The behavior of the fungi in liquid culture revealed no obvious antagonistic cell-cell contact interactions, and mixed cultures of Ma549 and Mr2575 in 0.1% yeast extract medium (YEM) grew over each other’s hyphae as well as their own (Fig 1A).

We tested the ability of Ma2575 and Ma549 to germinate in liquid media with different nutritional complexities. Unlike Ma549, a minority of Mr2575 conidia could germinate on water alone, and >99% of Mr2575 conidia germinated on a very low level of nitrogenous nutrients (0.0125% yeast extract medium) as compared to 61.3% Ma549 conidia (Tables 1 and B in S1 Data). Both strains showed 100% germination on a dichloromethane extract of *M*. *sexta* fifth instar larval cuticle, but Mr2575 developed long straight hyphae (Fig 1B), while Ma549 exhibited shorter lengths with terminal swellings or swollen cell clumps (Fig 1C), suggesting possible appressoria (infection structures that facilitate penetration). This effect was also observed with fungi growing on coverslip cultures sitting on agar cubes containing *M*. *sexta* extract (Fig 1D).

**Table 1:** Percent germination and mean hyphal length ± standard error (N = 33) of Mr2575 and Ma549 spores incubated for 16 hours in water, 0.0125% yeast extract medium (YEM), 0.0125 or 0.1% root exudate, or a dichloromethane lipid extract of fifth instar *M*. *sexta* larval cuticles (Table B in S1 Data for raw data).

	Mr2575		Ma549	
	%Germination	Mean hyphal length (μm)	%Germination	Mean hyphal length (μm)
Water	5.79%	15.42 ± 1.179	0	NA
0.0125% Root exudate	32.50%	39.58 ± 4.061	0	NA
0.1% Root exudate	46.40%	57.64 ± 3.648	10.26%	48.81 ± 1.223
0.0125% YEM	99.14%	41.09 ± 2.327	61.29%	11.42 ± 0.745
*Manduca* cuticle extract	100%	71.52 ± 2.989	100%	30.48 ± 1.937

Some of the genes required by Mr2575 to colonize roots are expressed in response to root exudates [17]. About a third of Mr2575 conidia germinated within 16 hours in 0.0125% root exudate medium which did not support germination of Ma549 spores. Only 10% of Ma549 spores germinated in 0.1% root exudate (Table 1). Roots were a more favorable environment for germination than root exudate with 93% of Mr2575 conidia germinating within 10 hours post inoculation as compared to 46% Ma549 conidia (Table C in S1 Data). Mr2575 germ tube elongation (21.17 ± 2.11 μm) on roots was also faster than Ma549 germlings (16.58 ± 2.27 μm) (t = 2.12, p < 0.05).

Co-cultures on agar have been used to simulate the physiological conditions that occur during the interactions of fungi on solid environments [16]. Colonies of both Mr2575 and Ma549 grown on potato dextrose agar ceased spreading when they contacted each other (S1 Fig). Mr2575 produced aerial hyphae that sometimes-overlapped Ma549 colonies (S1 Fig) but single colonies co-existed as discrete individuals in a spatially structured community for months. Although both Mr2575 and Ma549 consolidated acquired habitat their growth patterns were quite different. Lateral growth of single colonies of Mr2575 and Ma549 distantly (3 cm) separated from other individuals was 22.47 ± 0.3 and 16.52 ± 0.21 (n = 16), respectively (t = 16.28 p <0.00001). Growth of Mr2575 (Ma549) was reduced (p <0.00001) to 16.5 ± 0.27 (13.29 ± 0.19) with other individuals in close (1.5 cm) vicinity (Table D in S1 Data). Irrespective of other individuals Ma549 spread more slowly than Mr2575 but sporulated faster (within 3 days compared to 4 days for Mr2575) and more profusely (S1 Fig), suggesting selection on Mr2575 for more long-range foraging growth which in Ma549 is offset by rapid allocation of resources to condiogenesis and dispersal. Both foraging growth and spore dispersal offer opportunities for expanding feeding range but aggregate chances of finding additional nutrients by one or other of these two methods, rather than dying *en route*, may differ for Mr2575 and Ma549 (see Andrews (1992) for a discussion of fungal growth strategies [18]). Selection on Ma549 for sporulation is consistent with it exploiting small spatially localized resources in nature, and a greater need than Mr2575 to be able to reach relatively distant nutrient sources not accessible by hyphal spread.

### Disease progression and mortality of *Drosophila melanogaster* and *Sarcophaga bullata* infected by Ma549 or Mr2575

**Fig 2:**
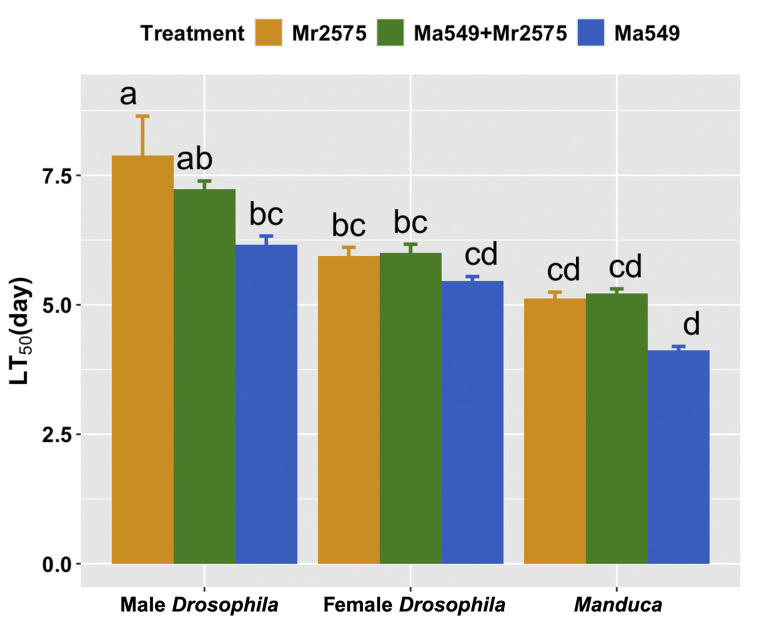
Median lethal times (LT_50_s ± SE) of *Drosophila* (both sexes) and *Manduca* infected by Ma549, Mr2575 or Ma549 + Mr2575. The different lowercase letters at the top of each bar indicate significant differences in LT_50_s by Tukey’s test (Two-way ANOVA, P < 0.05).

*Drosophila* provides a model that facilitates testing large numbers of insects that can be segregated by sex (Fig 2). As we found before [19], male *Drosophila* are more resistant to fungal disease than females (Fig 2 and Table E in S1 Data) implying that the sexes differ in their interactions with pathogens. The log-rank test (Mantel-Cox test) was used to evaluate differences between survival curves. Ma549 killed *Drosophila* faster than Mr2575 (*χ*2 = 18.92 (1), p<0.0001) (N ≥ 125 per sex per fungus) (Table E in S1 Data, survival curves are below the raw mortality data). Classical “tragedy of the commons” virulence theory predicts that co-infecting pathogens should race each other for resources resulting in faster kill [20,21]; however, time of death following mixed infections was not determined by the faster killing strain Ma549. In males the lethality of mixed infections was intermediate between Mr2575 (*χ*2 = 8.534 (1), p = 0.0035) and Ma549 (*χ*2 = 43.03 (1), p<0.0001), whereas in females the lethality of mixed Ma549+Mr2575 infections was similar to Mr2575 alone (*χ*2 = 0.002 (1), p = 0.96).

Colonization of the hemocoel was estimated as colony forming units (CFU) in 20 randomly selected living male or female flies from each treatment (Table F in S1 Data). At the LT_50_ time point (i.e., when 50% of the flies were dead) Ma549 CFUs in surviving flies outnumbered those of Mr2575 >29-fold, with no significant differences in Ma549 CFU counts between single or mixed infections. However, there were significantly more Ma549 CFUs in female flies (204 ± 21.56/fly) than males (133 ± 18.31) (t = 2.50954, p = 0.016469) showing that females were less able to resist colonization by Ma549.

**Fig 3:**
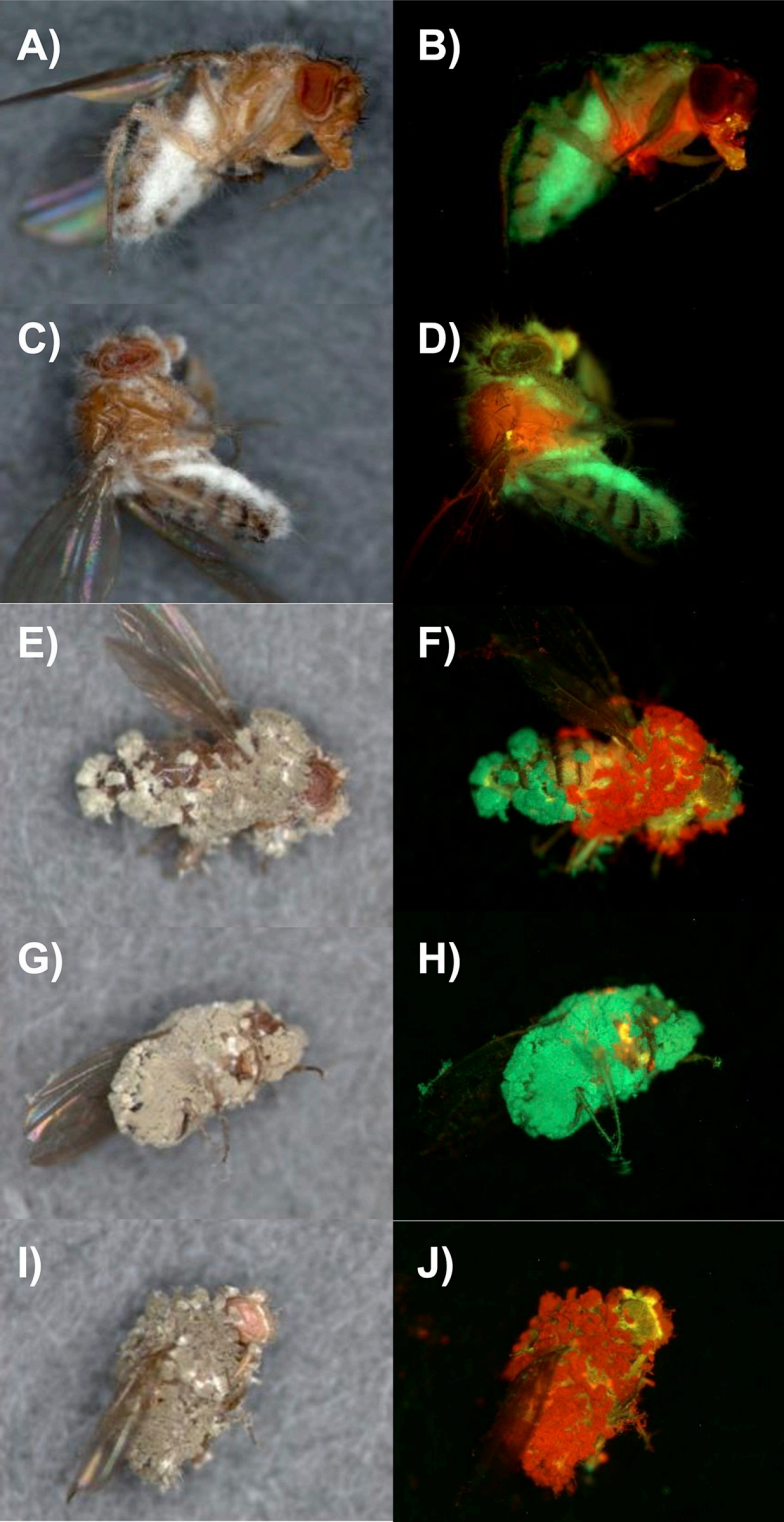
Colonization post-mortem of *Drosophila* by topically applied Mr2575-Cherry and Ma549-GFP. A) bright field and B) overlay of Cherry and GFP images of a cadaver ~24 hrs. post-mortem. C) bright field and D) overlay of Cherry and GFP images of the surface of cadaver ~48 hrs. postmortem. E, G, I) bright field and F, H, J) overlays of Cherry and GFP images of exemplar cadavers five-days postmortem showing variation in sporulation. The respective GFP and Cherry images comprising overlays are shown in S2 Fig along with additional examples from a time course of *Drosophila* infection.

We might expect that the greater competitiveness of Ma549 in the hemocoel following mixed infections would result in Ma549 emerging over most of the cadavers but that was often not the case. Ma549-GFP and Mr2575-Cherry were used to track infections in 10 randomly selected male or female flies from each treatment at daily intervals. Both fluorophores were sufficiently bright as to be clearly visible from outside the infected insect’s body (Fig 3). Green fluorescence was dispersed throughout most female and male cadavers within a few hours of being killed by single infections of Ma549-GFP. In eight out of 10 females and six of 10 males killed by Mr2575, cherry fluorescence was brightest in the thorax (S2 Fig). One day postmortem Mr2575 single infections had dispersed throughout cadavers, whereas in mixed infections one-, two- and three-days post-mortem, Mr2575 predominated in the thoraxes of significantly more (Z score test: *z* = 2.8545, p = 0.00438) females (73.3%, n = 30) than males (36.7%, n = 30) indicating that spatial partitioning between Ma549 and Mr2575 was influenced by sex. Mr2575-Cherry fluorescence was greater than Ma549-GFP in the abdomen of two males (6.7%), and some abdomens contained an overlap of cherry and GFP signals (S2 Fig) suggesting that co-infecting strains can coexist and not mutually exclude each other.

Ma549 emerged from cadavers with single or mixed infections one day post-mortem, usually through the abdominal intersegmental membranes which represent an area of cuticle weakness (Figs 3 and S2). Mr2575 emerged from cadavers within three-days post-mortem but by five days both Ma549 and Mr2575 had fully sporulated. There was a broad range of variation in spore production ranging from flies almost covered in Ma549 spores to flies almost covered in Mr2575 spores. This indicates that although Ma549 is more virulent than Mr2575, and has higher competitive ability during host colonization, that does not necessarily translate to higher transmission potential. This could be explained by Mr2575 at its initial infection sites being the source of the emergent hyphae rather than blastospores in the hemolymph. Given the equivalency in spore doses, environment and the inbred fly population this variation also indicates that chance events may contribute to the outcomes of mixed infections.

Host body size could influence the ability of Ma549 and Mr2575 to establish territories. Our results with *Drosophila* demonstrated that even a small insect body (male and female *D*. *melanogaster* weigh about 0.5 and 0.7 mg, respectively [22]) can support co-infections, but it seemed likely that these would be easier to study in a large insect. *Sarcophaga bullata* weighs about 45 mg [23]. Mixed infections with Mr2575 and Ma549 resulted in *S*. *bullata* cadavers that were almost totally covered in Ma549 (S3 Fig). *S*. *bullata* is not a common model system so we followed up these studies with *M*. *sexta*.

### Disease progression and mortality of fifth instar *M*. *sexta* caterpillar infected by Ma549 or Mr2575

**Fig 4:**
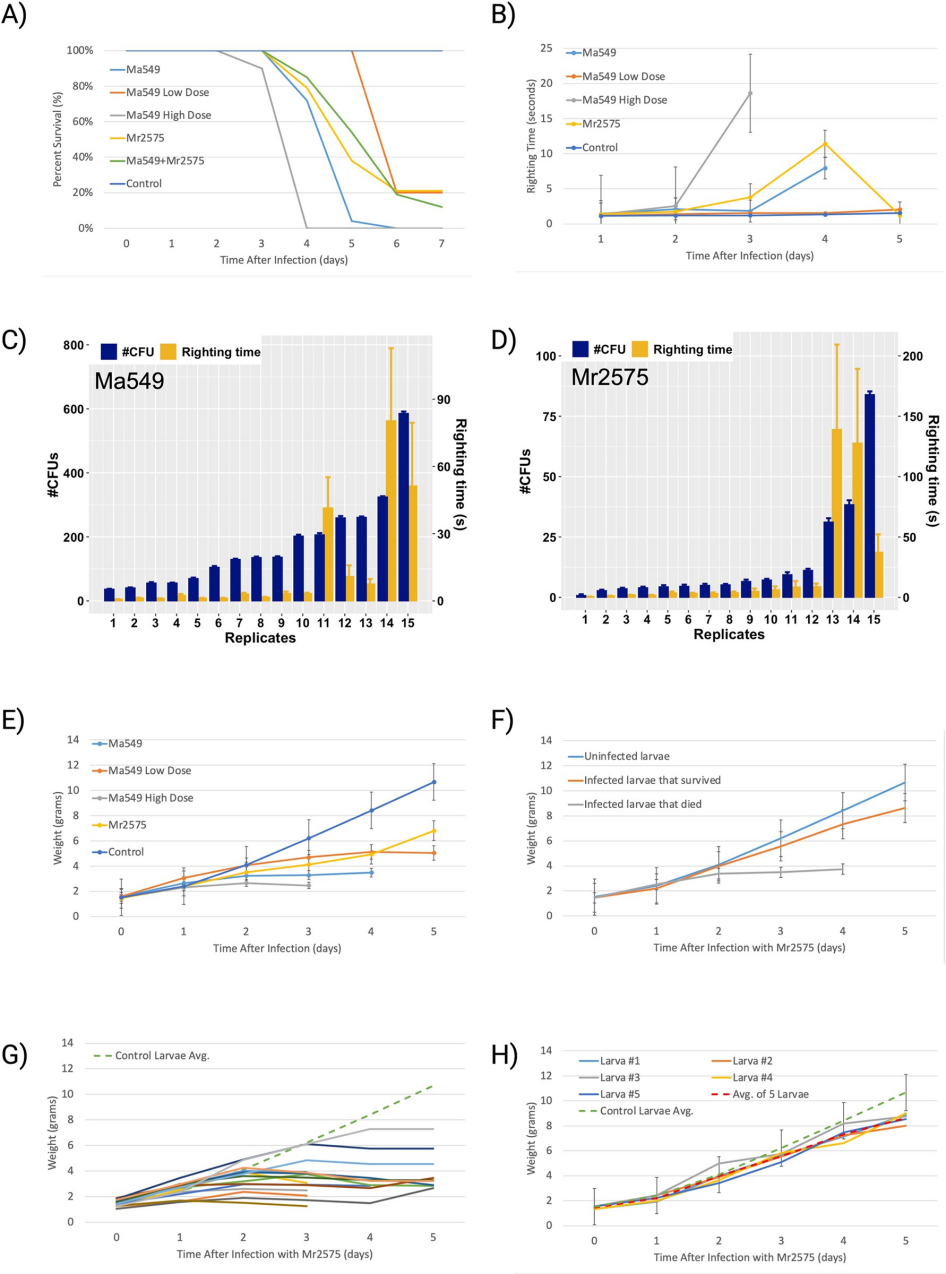
Effect of *Metarhizium* mycosis on fifth instar *M*. *sexta* larvae. A) Time courses of infection after larvae were dipped into conidial suspensions of Ma549 (2 x 10^6^ conidia/ml), Ma549 low dose (5 x 10^5^conidia/ml), Ma549 high dose (4 x 10^6^ conidia/ml), Mr2575 (2 x 10^6^conidia/ml), Ma549 + Mr2575 (1 x 10^6^ Ma549 conidia/ml + 1 x 10^6^ Mr2575 conidia/ml). Mean ± SE (*N* >10). Mortality was assessed daily and there was no mortality on days 0–2. B) Time course of how quickly larvae righted themselves after being placed on their backs (righting time) when inverted 10 times in quick succession. Mean ± SE (*N >* 10). C) hemolymph CFUs (Left, mean ± SE) and righting times (Right, mean ± SE) for 15 individual *M*. *sexta* larvae within 24 h of death by Ma549. D) hemolymph CFUs (Left, mean ± SE) and righting times (Right, mean ± SE) for 15 *M*. *sexta* within 24 h of death by Mr2575. E) Effect of mycosis on weight gain (in grams) of larvae. Mean ± SE (*N >* 10). F) Effect of Mr2575 (2 x 10^6^ conidia/ml) mycosis on weight gain distinguishing between larvae that survived mycosis and larvae that died. G) Weight gain of 15 individual larvae that died due to Mr2575 mycosis compared to uninfected control larvae (dashed green line). H) weight gain of five individual larvae that survived Mr2575 mycosis and successfully pupated. Controls were dipped in 0.01% Tween 80. The larvae were weighed and tested for righting time daily 5–7 hours after lights-on. Where no point is shown, the insects were dead due to mycosis.

We used fifth instar *M*. *sexta* larvae to elucidate the interactions between co-infecting Ma549 and Mr2575. Resembling *Drosophila*, we found that LT_50_ values (the median lethal times after exposure) was slightly lower in mixed Mr2575/Ma549 infections (natural route) than with single genotype infections even though total inoculum size was kept constant (Fig 4A and Tables G-J in S1 Data). Ma549 kills *Manduca* caterpillars significantly faster than Mr2575 (Log-rank *χ*2 = 6.20 (1), p = 0.0128). Mr2575 kills slightly faster than Ma549+Mr2575 but the difference was not significant (Log-rank *χ*2 = 0.01 (1), p = 0.9202, N = 25 for each treatment). Overall, these results with *M*. *sexta* and *D*. *melanogaster* do not support interactions between co-infecting genotypes changing rates of exploitation and hence virulence. Interactions might have been expected if they were toxic to each other reducing population growth rate or conversely within host competition resulted in more rapid host exploitation.

We next investigated the association between infection, fungal load, and disease progression in isogenic infections. Melanization of the cuticle and reduction in weight gain are known symptoms of *M*. *anisopliae* infection in *M*. *sexta* [24]. Fifth-instar *M*. *sexta* larvae were weighed daily 5–7 hours after lights-on until the end of the growth phase (i.e., peak weight), which is the day on which larvae entered the wandering stage (indicated by the heart becoming visible through the cuticle) and stopped feeding. Uninfected control caterpillars showed growth patterns very similar to those previously reported [25], with the last (fifth) larval instar growing from a mass of approximately 1.2 g to about 11 g (Fig 4E and Table I in S1 Data), hence almost 90% of the final mass of the larva is gained during this single instar [24]. The failure to flourish, as shown by reductions in weight gain, was linked to disease progression by examining fungal load in hemolymph samples by fluorescence microscopy and counting CFUs (Fig 4C and 4D). We also investigated how quickly the larvae righted themselves after being placed on their backs (righting time) (Fig 4C and 4D and Tables G and H in S1 Data). Healthy larvae usually righted themselves within two seconds even when inverted 10 times in quick succession (Table I in S1 Data), indicating a strong righting reflex.

Melanization is the first symptom of infection with either Ma549 or Mr2575, sometimes appearing within 30 hrs. of infection, when the righting time and weight gain were within normal ranges. Failure to gain weight and subsequent weight loss began two to three days post infection (Fig 4E). Close to death, many diseased caterpillars showed variable responses to being inverted. When inverted 10 times in succession they sometimes righted themselves quickly and sometimes stayed on their backs for minutes resulting in high standard errors (Fig 4C and 4D). Even some heavily melanized and limp Ma549-infected caterpillars that had lost turgor-induced rigidity (probably because they did not eat), could still occasionally right themselves quickly after inversions. All Ma549 caterpillars that showed any symptoms by day two died within six days, irrespective of the spore dose, suggesting that caterpillars cannot defeat an Ma549 infection that produces symptoms. The few survivors of low dose Ma549 gained weight and showed no cuticular melanization detectable with a magnifying lens.

There was a nonlinear relationship between Ma549 fungal load and average righting time/caterpillar, with no apparent effect until fungal loads reached high levels, which occurred at the earliest three-days post-infection which was one to two days preceding death. Consistent with dissemination of Ma549 in living hosts, within 24 h preceding death hemolymph samples contained 37–585 Ma549 CFUs per μl (X = 176.5 ± 42.98). There was a moderately significant correlation between CFUs within a day before death and righting time (r = 0.72, p = 0.024, n = 15). Despite having much lower (t = 4.21. p = 0.000239) fungal loads one day preceding death (range 2.8 to 84 CFUs per μl, X = 14.56 ± 5.67), the association between Mr2575 CFUs and righting time was still positive (r = 0.542, p = 0.037, n = 15). The impact on righting time of a comparatively small number of Mr2575 blastospores is consistent with toxin-induced paralysis by Mr2575, whereas the failure of Ma549-infected insects to right themselves may be due to energy depletion.

Larvae showed a rather broad range of individually variable responses to Ma2575. About a quarter of Mr2575 infected caterpillars that showed melanization and a temporary failure to gain weight survived to pupation (Fig 4F–4H), indicating that neither symptom necessarily precedes death. Two out of 25 hemolymph samples contained single CFUs four days post-infection in caterpillars that went on to survive providing no evidence that survivors were more tolerant of Mr2575. Mr2575-infected caterpillars were darker overall than caterpillars infected with Ma549. Four days post-infection, seven out of 35 Mr2575 larvae with lethal infections blackened over their entire body; hemolymph samples from these caterpillars contained yeast and bacterial contaminants (S5 Fig) whereas caterpillars with substantial areas of green cuticle did not. Sections of blackened caterpillars showed the gut disrupted or even missing suggesting that the gut was the source of the septicemia. The hemolymph of Ma549 infected caterpillars appeared to be a monoculture of Ma549 (S5 Fig).

### *In vivo* examination of *M*. *sexta* caterpillar colonization by Ma549 and Mr2575

**Fig 5:**
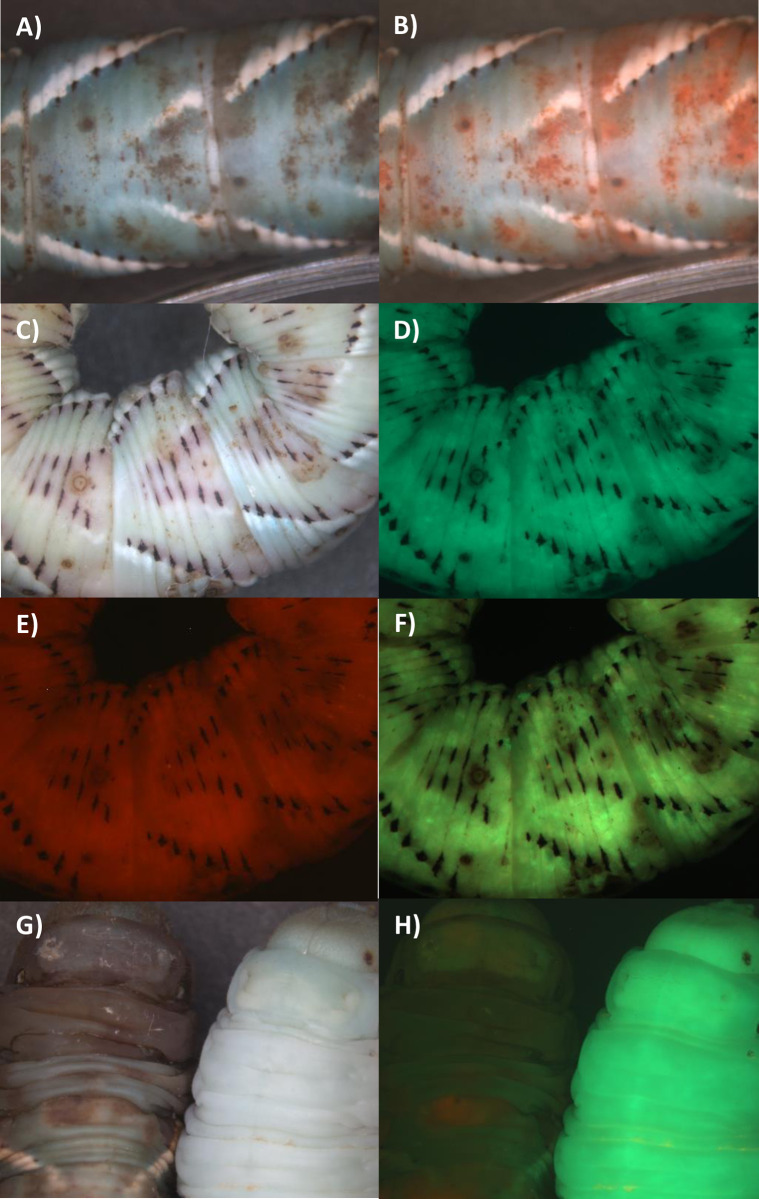
Colonization of fifth instar *M*. *sexta* larvae by topically applied Mr2575-Cherry and GFP or dsRed labelled Ma549. A) bright field and B) bright field overlaid with Cherry image showing Cherry fluorescence localized to melanized penetration sites in a caterpillar three days after topical infection with Mr2575-Cherry. C) bright field, D) GFP image, E) dsRed image and F) overlay of GFP and dsRed images of a caterpillar three days post infection with both Ma549-GFP and Ma549-dsRed. G) bright field and H) overlay of Cherry and GFP images of caterpillars four days post infection with Mr2575-Cherry (left) or Ma549-GFP (right). Both caterpillars responded weakly to stimuli but the Mr2575 infected caterpillar has darkened and desiccated and shows weaker fluorescence than the Ma549 infected caterpillar.

We visualized the stages of colonization of fifth instar *Manduca* caterpillars using fluorescently labeled strains (Ma549 expressing GFP or dsRed, Mr2575 expressing GFP or Cherry) (Fig 5). We observed no differences in growth or virulence between isogenic strains expressing different fluorophore markers and unless stated otherwise our results describe Mr2575-Cherry and Ma549-GFP. Thirty to 36 hours after natural inoculation, dark melanization spots were observed on caterpillars infected with Mr2575-Cherry with Cherry fluorescence localized around those melanized spots (Fig 5A and 5B), indicating that this is where the fungus had penetrated eliciting an immune response. Melanization spots were lighter following infection with both Ma549-GFP and Ma549-ds-Red, and fluorescence was not limited to these spots (Fig 5C, 5D, 5E and 5F). Instead by the time of death Ma549 was systemically distributed throughout most of the caterpillars, showing that the infection had disseminated, presumably through the insect’s open circulatory system (Fig 5F). At the time of death, Mr2575-Cherry fluorescence remained weak and was localized to the melanized cuticle (Fig 5G and 5H).

**Fig 6:**
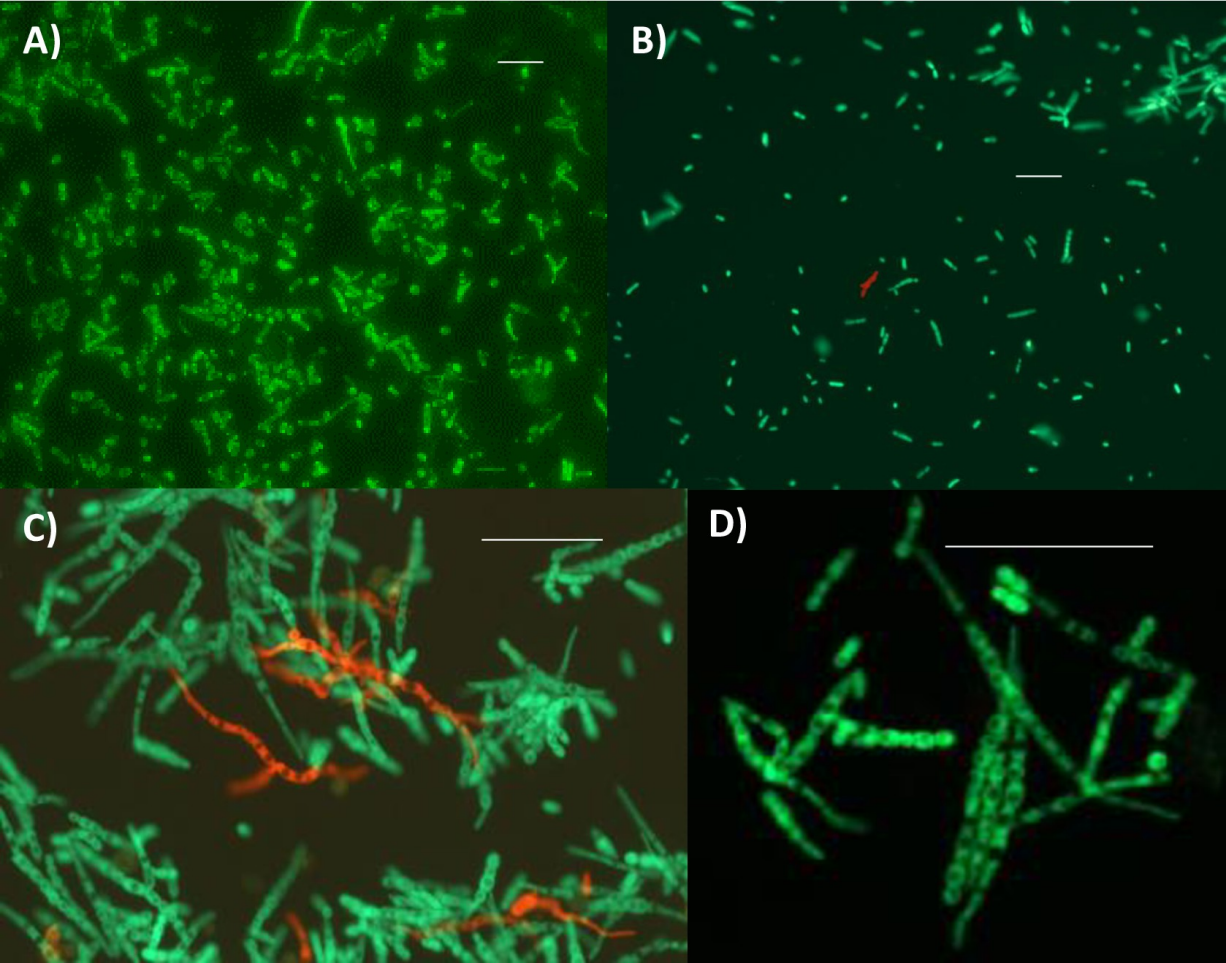
Colonization of fifth instar *M*. *sexta* larvae by topically applied Cherry-Mr2575 and GFP-Ma549 A) Smear of fat body tissue from a moribund but still living caterpillar four days after topical infection with GFP-Ma549 showing colonization of the fat body by budding blastospores. Hemolymph samples taken several hours pre-death (A) and post-death (B) from caterpillars topically inoculated with both GFP-Ma549 and Cherry-Mr2575 showing that Ma549 proliferates more abundantly but developmental processes are similar to Mr2575 with blastospores in living insects transitioning to pseudohyphae about time of death. D) The pseudohyphae consist of round yeast like cells attached to each other. The GFP and Cherry images comprising panel B are shown in S4B Fig. Bar = 50 μm.

Consistent with CFU counts, few Mr2575 blastospores were observed in hemolymph, whereas Ma549 blastopores proliferated abundantly. Samples of fat body tissue also contained numerous Ma549 blastospores by the time of insect death (Fig 6A). The predominance of Ma549 blastospores in the hemolymph was obvious when spore mixtures of Ma549-GFP and Mr2575-Cherry were topically applied to the cuticle (natural infection) (Fig 6B and 6C). Both fungi only produced budding blastopores (Fig 6B) until just preceding death when long hyphal chains (Fig 6C) appeared in nearly comatose larvae, and these in turn budded of more blastopores (S6 Fig). The hyphal chains had constrictions at the septal junctions, potentially leading to a loss of cytoplasmic continuity between cells (Fig 6D) and distinguishing them as pseudohyphae rather than true hyphae [26]. At this stage, blastospores may no longer facilitate rapid dissemination in an insect body as there is no hemolymph flow, and pseudohyphae could be the morphological intermediate between blastospores and the hyphae that exit through the cadaver cuticle to sporulate. Intermediate morphologies including elongated single blastospores were also common (S6 Fig). GFP-fluorescence can be used as a proxy for cell viability [19]; all 400+ Ma549-GFP blastospores/pseudohyphae in several fields of view fluoresced showing that most of the fungus was alive.

**Fig 7:**
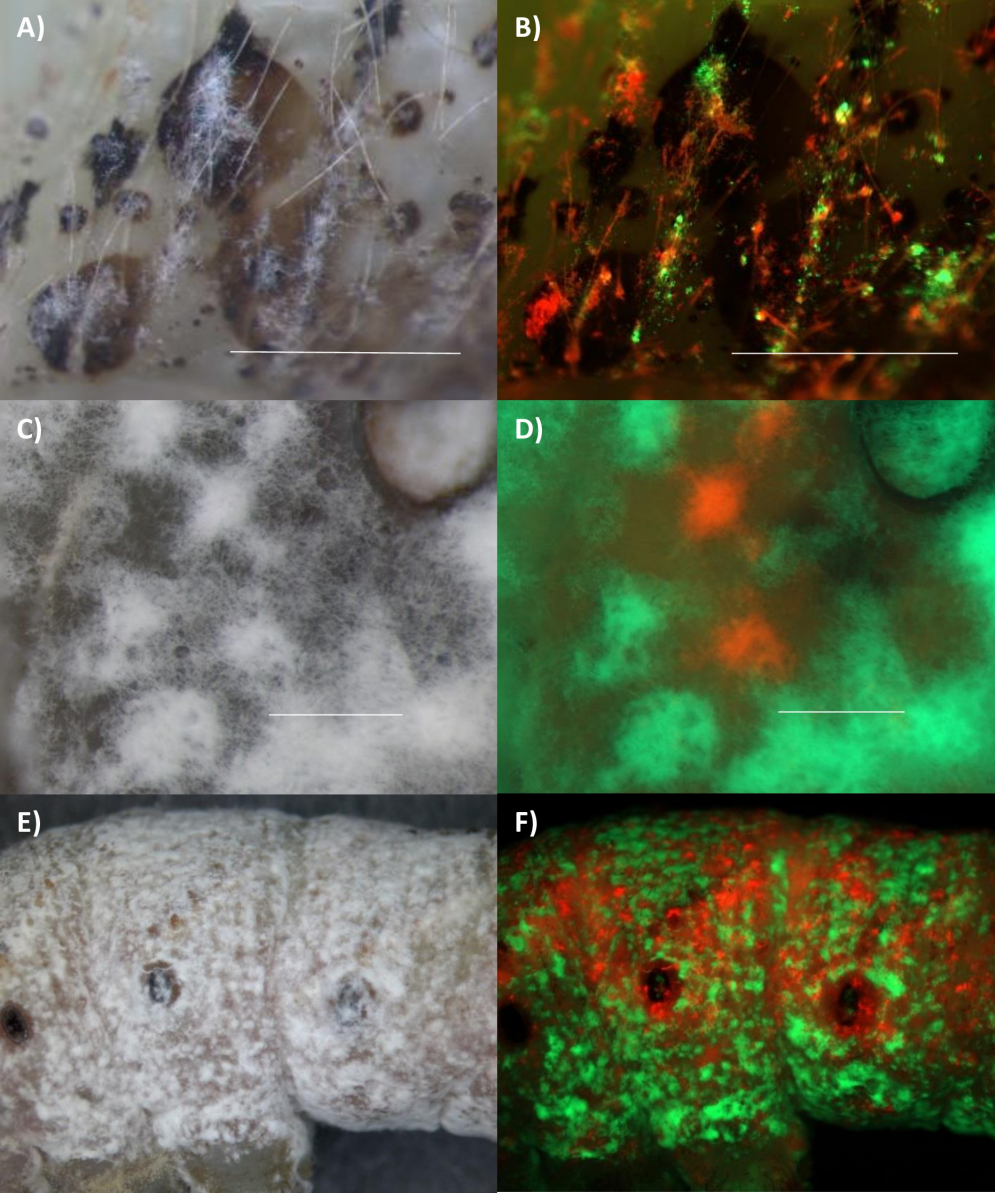
Emergence of Mr2575-Cherry and Mr2575-GFP from fifth instar *M*. *sexta* cadavers. A) bright field and B) overlay of cherry and GFP images of the surface of cadaver ~6 hrs. postmortem, with Mr2575-Cherry and Mr2575-GFP emerging through the same patches of melanized cuticle. C) Bright field and D) overlay of Cherry and GFP image of surface of cadaver with partial view of spiracle (top right corner) ~6 hrs postmortem showing separate GFP and Cherry fluorescing colonies. E) Bright field and F) overlay of Cherry and GFP image of cadaver ~18 hrs postmortem showing that Mr2575-Cherry and Mr2575-GFP clones had retained their separate identities *in insecta*. The respective GFP and Cherry images comprising overlays are shown in S4C Fig. Bar = 200 μm.

Both Ma549 and Mr2575 emerged from cadavers within 24 hours of mortality. Hyphae of both fungi emerged singly or as small tufts, and frequently emerged at hair sockets (S7 Fig), which may represent a point of weakness in the cuticle. Ma549 usually grew as round domes of fungi, whereas Mr2575 showed less dense growth that spread laterally over the cuticle surface (S7 Fig). Mr2575 initially emerged at the melanization sites, consistent with the fungus being localized at these sites at death (Fig 7A and 7B), and co-infections with Mr2575-Cherry and Mr2575-GFP produced separate Cherry and GFP labelled colonies on the cadaver (Fig 7). This is consistent with Mr2575 at the initial infection sites being the source of the emergent hyphae rather than the source being a mixed population of Cherry and GFP labelled Mr2575 in the hemolymph. However, large melanized areas usually produced both cherry and GFP-tagged Mr2575 colonies, so multiple conidia had initiated infection at these sites (Fig 7B).

**Fig 8:**
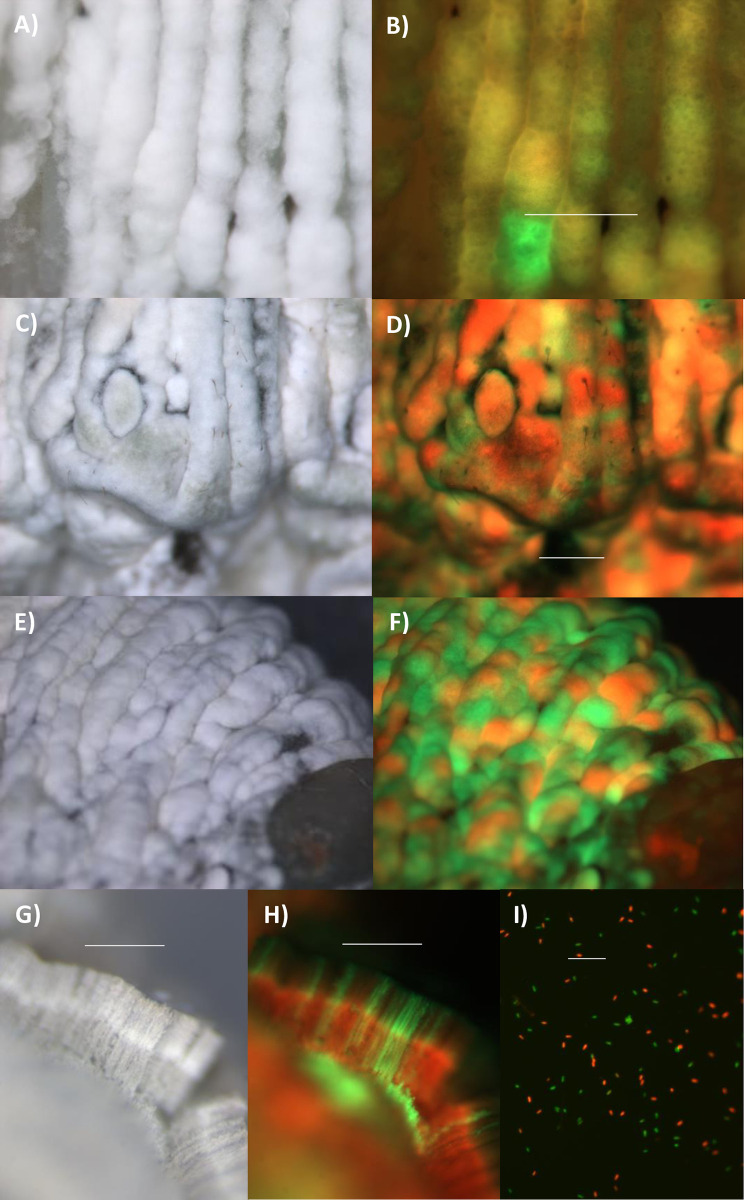
Infection of fifth instar *M*. *sexta* larvae by simultaneous application of GFP or dsRed labelled Ma549 to the cuticle. Emergence of GFP or dsRed-Ma549 from cadavers. A), C) and D) bright field images, and B), D and E) overlays of dsRed and GFP images of caterpillars showing that many Ma549 colonies emerging from cadavers fluoresced predominately red or green whereas others fluoresced orange indicating mixed colonies. G) bright field and H) overlay of dsRed and GFP showing Ma549 conidiophores on a cadaver being either red or green. I) spores scraped from a cadaver coinfected with Ma549-GFP and Ma549-dsRed showing that spores were either red or green. The respective GFP and dsRed images comprising overlays are shown in S4D Fig. Bar = 200 μm for B) and D), and 100 μm for G), H) and I).

Unlike Mr2575, Ma549 emerged over the entire cadaver rather than at melanization sites. Mixed Ma549-GFP and Ma549-dsRed infections produced a fluorescent spectrum with some areas of cadavers having discrete dsRed and GFP colonies and other areas showing intermixed colonies with a yellowish orange appearance in overlays (Fig 8). However, even in mixed dsRed and GFP colonies that appeared yellow overall, spores fluoresced either red or green (Fig 8G–8I) suggesting that the hyphae that produced them had not undergone vegetative fusion between lines encoding different fluorophores.

**Fig 9:**
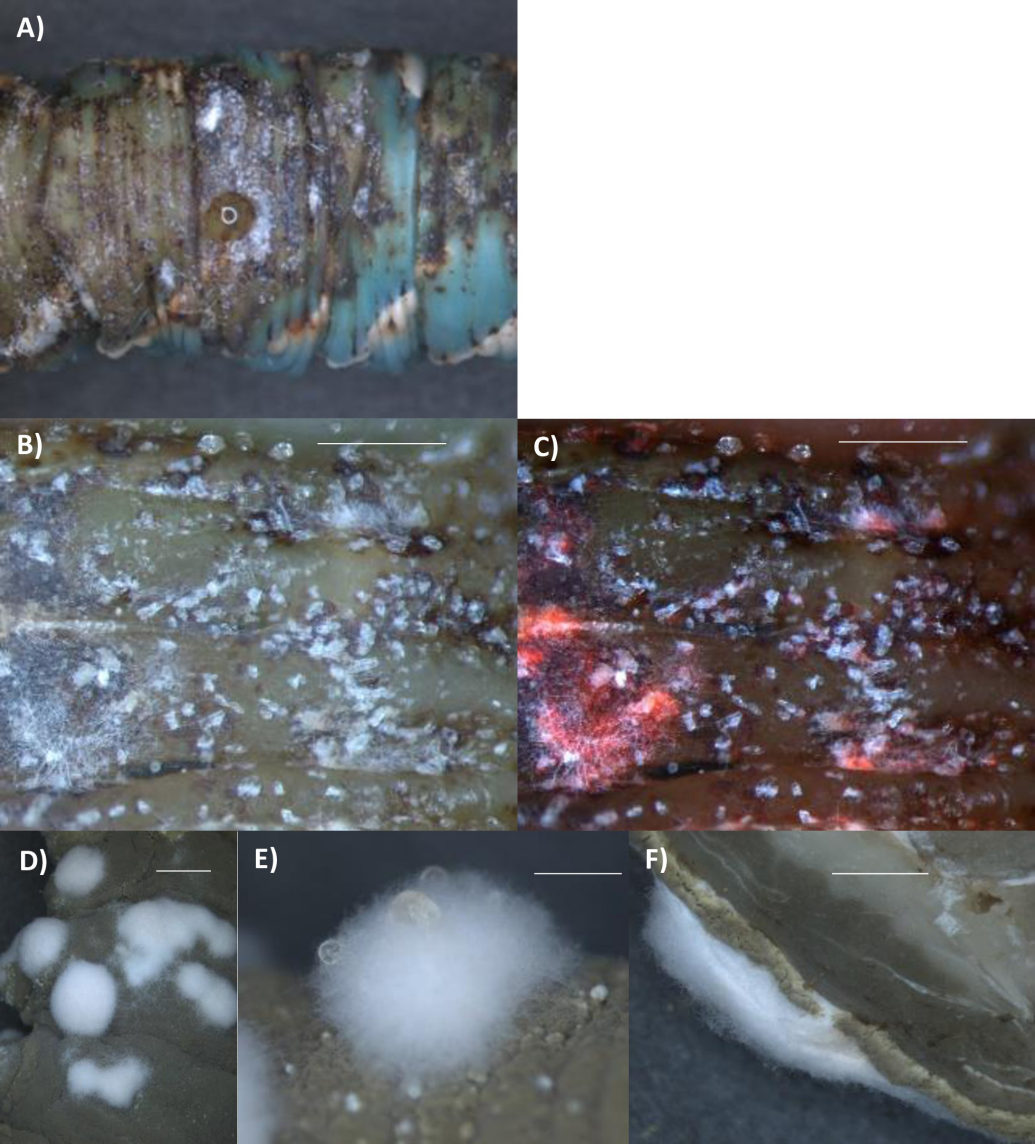
Mr2575 infection weakens the cuticle. A) dorsal surface of cadaver ~6 hrs postmortem. The arrow points to leakage from a hole made by gentle pressure from a blunt forceps. B) bright field and C) bright field overlaid with Cherry image showing white crystals on melanized cuticle. C) Mr2575 producing round clumps of non-sporulating aerial hyphae on mature cadavers (following sporulation), that usually exuded several guttation droplets (liquid droplets) shown by arrows (D). A) bright field image of cross section of cadaver showing aerial hyphae growing through a narrow breach between sporulating hyphae. Additional examples with fluorescence images are shown in S9 Fig. Bar = 500 μm.

The mechanical strength of the cuticle following death greatly diminished as the fungus re-emerged through it, or even before in the case of some Mr2575 infected caterpillars. The black melanized cuticle occupied by Mr2575 was particularly soft and often split spontaneously or could be easily punctured by gentle pressure with the blunt tip of a forceps (Fig 9A). In contrast to the cadavers from Ma549 infected caterpillars, Mr2575 killed caterpillars often continued to darken post mortem, and melanized cuticle “sweated” releasing fluids through the cuticle which evaporated leaving white crystals on the cadaver and its environs (Fig 9B and 9C), and resulted in the volume of the cadaver shrinking so the cuticles of Mr2575 killed caterpillars were wrinkled (Fig 5G).

Unlike Ma549, Mr2575 produced round clumps of non-sporulating aerial hyphae on mature cadavers (following sporulation) (Figs 9D–9F and S9), that appeared to grow through narrow breaches between sporulating hyphae (Fig 9F). These clumps of aerial hyphae usually exuded several guttation (liquid) droplets (Fig 9E). Guttation droplets generated by *Metarhizium* in some culture conditions contain lytic enzymes and destruxins [27], potentially serving as a reservoir of biologically active molecules as Mr2575 spores produced on cadavers have much higher levels of lytic enzymes on their surfaces than spores produced in culture [28].

#### Spatial exclusion plays a major role in the competitive advantage of Ma549 in mixed infections of fifth instar *M*. *sexta*

**Fig 10:**
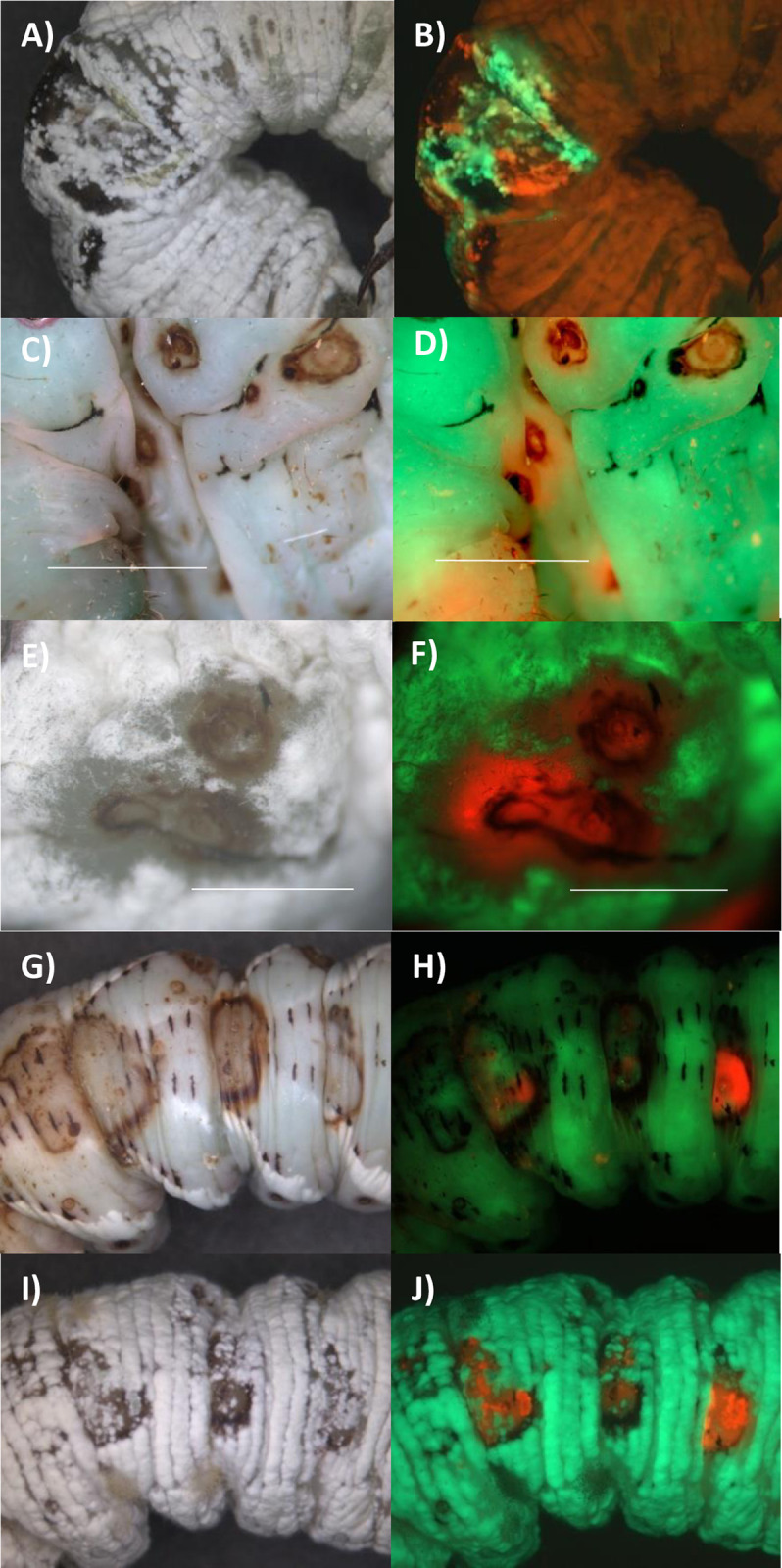
Infection of fifth instar *M*. *sexta* larvae by simultaneous topical application of Cherry-labelled Mr2575 and GFP or dsRed labelled Ma549 to the cuticle. A) bright field and B) overlay of Cherry/dsRed and GFP images of caterpillar infected with equal spore doses of Mr2575-Cherry, Mr2575-GFP and Ma549-dsRed. Cherry is brighter compared to dsRed allowing the two to be distinguished. C) bright field and D) overlay of GFP and Cherry images showing Cherry fluorescence localized to melanized penetration sites in a living caterpillar three days after topical co-infection with Mr2575-Cherry and Ma549-GFP. E) bright field, F) overlay of GFP and Cherry images, showing emergent Ma549-GFP hyphae not encroaching on Mr2575 penetration sites. G) and H) bright field images of the same caterpillar ~5 hrs and 32 hrs postmortem with corresponding overlays of GFP and Cherry images (F, H) showing Mr2575-Cherry fluorescence localized on melanized zones. The respective GFP and Cherry/dsRed images comprising overlays are shown in S4E Fig. Bar = 200 μm.

Interactions between fungi were visualized by infecting caterpillars with various combinations of Mr2575 expressing Cherry or GFP, and Ma549 expressing dsRed or GFP. As shown in Fig 10A and 10B, it was possible to distinguish between Ma549-dsRed and the more brightly fluorescing Mr2575-Cherry in caterpillars infected with both these two strains as well as with Mr2575-GFP.

With natural infections of fifth instar *M*. *sexta* (i.e., conidia of Ma549 + Mr2575 applied to the cuticle), most cadavers mummified rapidly as with Ma549 infections alone and Ma549 was dispersed through the cadaver whereas Mr2575 was localized at dark melanization sites (Fig 10C and 10D). Ma549 emerged and sporulated over most of the cadaver but seldom extensively encroached into the dark patches possessed by Mr2575 (Fig 10E and 10F). As with *Drosophila* infections (Fig 3) Ma549 usually began emerging from cadavers a few hours earlier than Mr2575 (Fig 10E–10J) although there were exceptions (S10 Fig).

**Fig 11:**
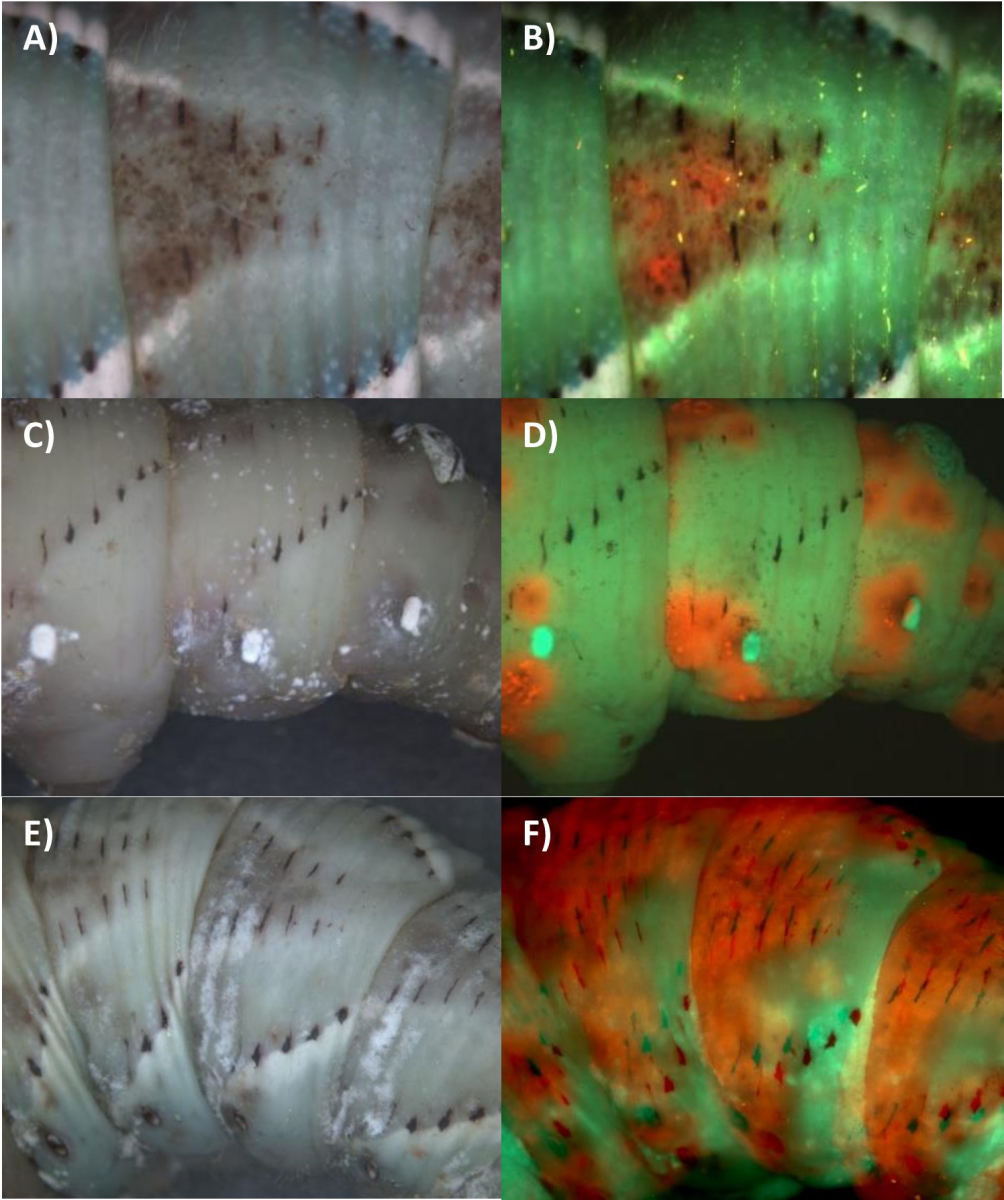
Pictures show recent (< 18 hrs) cadavers of *Manduca* infected by mixed injections of Ma549-GFP + Mr2575-Cherry. A), C), E) bright field and B), D) and F) overlays of Cherry and GFP images showing localization of Mr2575 to melanized patches near the front of segments, particularly within the dorsal spots and anterior to the spiracle. The respective GFP and Cherry images comprising overlays are shown in S4F Fig along with additional examples of localized distribution within segments (S9 Fig).

Coinfecting fifth instar *M*. *sexta* by injecting spores directly into the hemolymph, bypassing the cuticle, produced roughly equal relative representation of both genotypes emerging from cadavers (Fig 11). Thus, injection neutralizes the competitive dominance of Ma549 producing a much less one-sided infection, suggesting that as seen *in vitro* on different media the intrinsic rate of growth of Ma549 is not superior to Mr2575. Areas of the larvae colonized by Mr2575 were darker than those for Ma549 and the overlying cuticle usually melanized even though infection was by injection (i.e., the cuticle was not the site of infection) (Fig 11).

**Fig 12:**
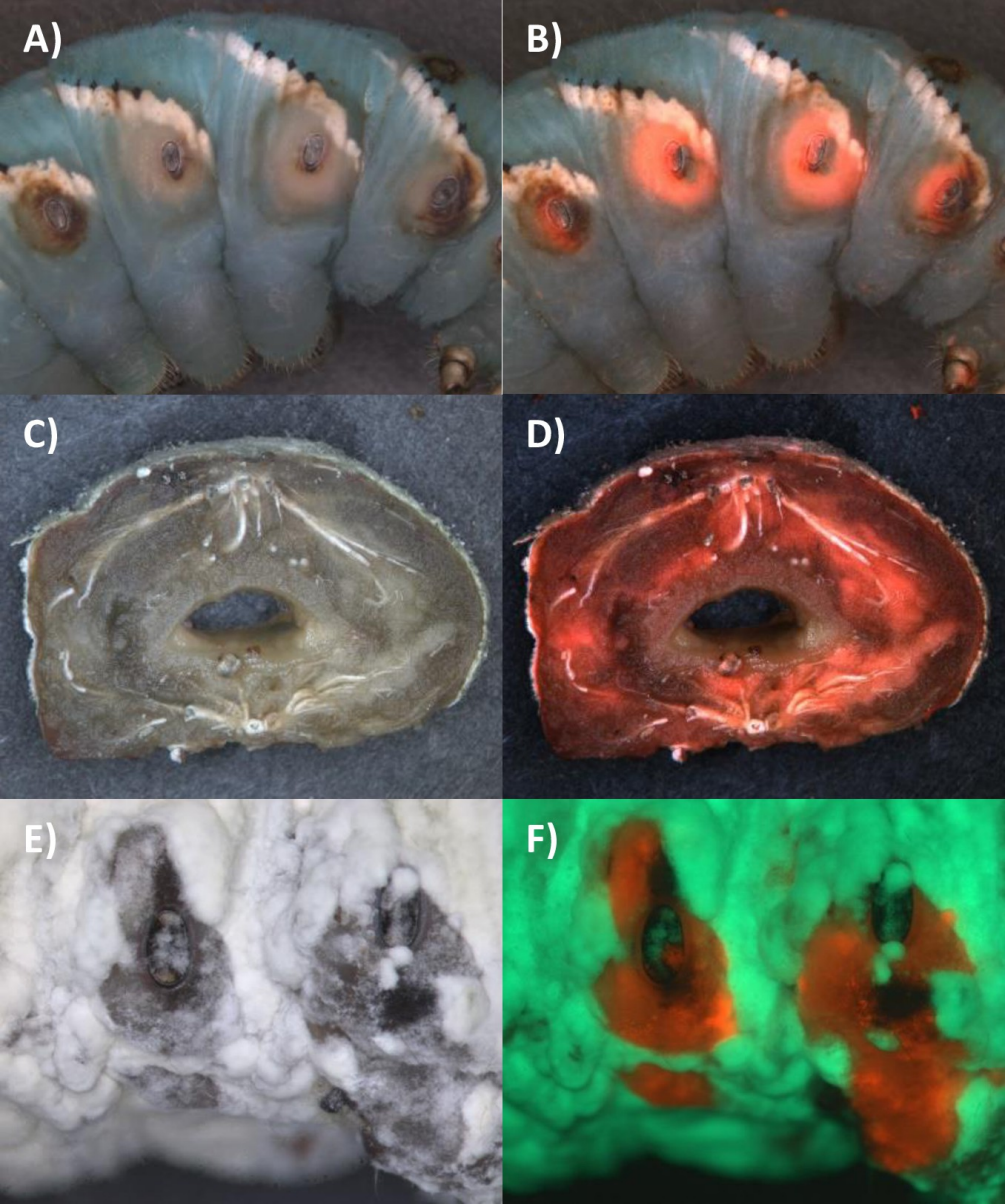
Topical infection of fifth instar *M*. *sexta* larvae by Cherry labelled Mr2575 is sometimes localized to spiracles and major trachea. A) bright field and B) bright field overlaid with cherry image showing cherry fluorescence localized to area around spiracles three days after topical infection with Mr2575 and one day before death. The fluorescent zones have lost green pigmentation and are surrounded by melanin rings. C) bright field and D) Cherry overlay of bright field showing a section of the same caterpillar in A) and B) two days postmortem with Mr2575 localized around major trachea radiating from spiracles. E) bright field and F) overlay of GFP and Cherry images following topical infection with Ma549-GFP + Mr2575-Cherry showing localization of Mr2575 to the spiracles. The respective GFP and Cherry images comprising the overlay (panel F) are shown in S4Gi Fig. S4Gii Fig shows a cadaver section of a mixed infection with Mr2575 predominating around the spiracle but Ma549 localized around the trachea. S4Giii Fig shows another contrasting cadaver with Mr2575 predominating around the spiracle and trachea.

Mixed injections should homogenously disperse Ma549 and Mr2575 spores in the host. Nevertheless, there was a remarkable spatial distribution of fluorescence with the majority of Mr2575-Cherry induced darkening and growth being localized to the front of individual segments with Ma549-GFP at the rear (Fig 11A–11F). The boundary between the two fungi was often sharply demarcated at segments, and the area of each segment dominated by Mr2575-Cherry usually included between the V-shaped arrangement of black spots on the dorsal surface and the area around the spiracles (Figs 11A–11F and S10). As spores were injected this would not have been because the spiracles provided an infection route, and indeed Ma549-GFP often emerged from the spiracles themselves (Fig 11C and 11D). This was also true in some natural infections with Mr2575-Cherry alone where Mr2575-Cherry was concentrated around the spiracles and major trachea (Fig 12A–12D) and in mixed natural infections where Mr2575-Cherry dominated areas around the spiracles (Fig 12E and 12F). However, Ma549 and Mr2575 showed similar declines in growth under 5% hypoxia (5% oxygen and 95% nitrogen) and 1% hypoxia (1% oxygen) with residual growth at 1% hypoxia of 26.2% (23.4%) for Ma549 (Mr2575) (Table K in S1 Data), indicating similar needs for oxygen.

For comparison, we also performed natural infections of hatchling first instar *M*. *sexta* which weigh about 1 mg [24]. Mixtures of Ma549+Mr2575 resulted in domains of either Ma549 or Mr2575 with neither fungus having an overall advantage (S8 Fig). Also contrasting with fifth instars, we saw no preference by Mr2575 for spiracles or any other first instar caterpillar structure compared to Ma549 showing that the timing of co-infections and the hosts developmental stage effect competitive interactions and the success of the pathogens.

**Fig 13:**
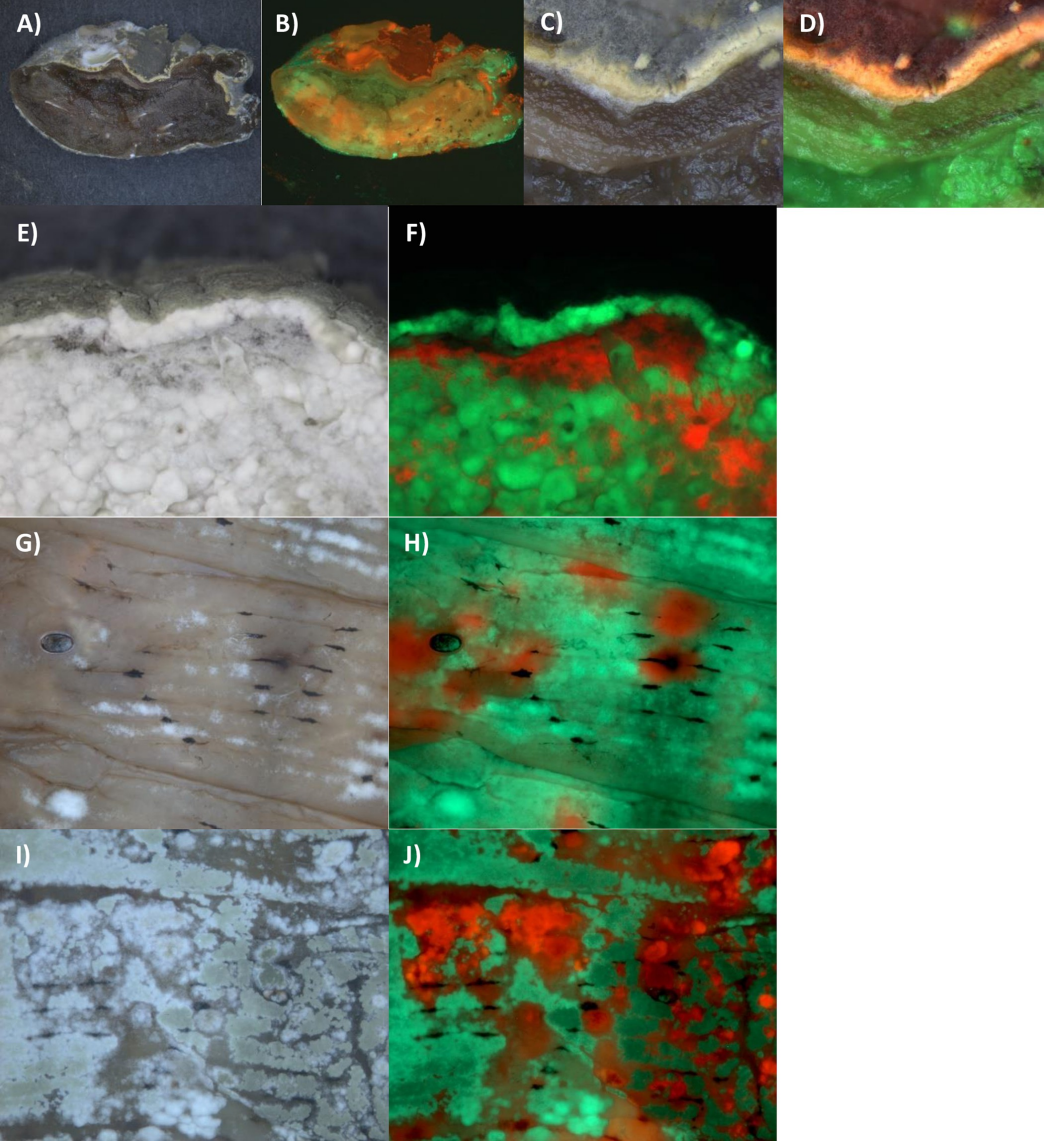
Sporulation by Mr2575 and Ma549 on cadavers does not always predict the identity of the predominant fungus in underlying tissues. A) bright field and B) overlay of Cherry and GFP images of cross section of cadaver 4 days post-mortem. The area in the circle is shown in higher magnification in C) bright field and D) overlay of Cherry and GFP showing sporulating Mr2575 overlying tissue containing predominately Ma549. E) bright field and F) overlay of Cherry and GFP images of cross section of cadaver 6 days post-mortem showing sporulating Ma549 overlying tissue containing predominately Mr2575. The section was incubated overnight to allow fungal outgrowth. G), I) bright field and H), J) overlays of Cherry and GFP images of dissected cuticles with underlying epidermis showing persistence of localization at dorsal sites between spots and around spiracles. The respective GFP and Cherry images comprising the overlays are shown in S4H Fig.

Cross sections of mummified fifth instar *M*. *sexta* cadavers showed that sporulating Ma549-GFP or Mr2575-Cherry usually overlay cadaver tissues where the same genotype predominated, but we also found exceptions where a thin layer of sporulating Mr2575-Cherry overlay tissues that were predominately Ma549-GFP (Fig 13A–13D) and vice versa (Fig 13E and 13F). We dissected fresh cadavers cutting along the ventral midline and removing hemolymph, gut, heart, the fat body surrounding tissues and the muscle layer but leaving the epidermis and some of its underlying fat body. When the isolated cuticles were incubated on plain agar both Ma549-GFP and Mr2575-Cherry retained the distribution they showed in whole insects. Mr2575-Cherry preferentially emerged between the V-shaped black spots and in the vicinity of the spiracles, and showed some demarcation at segments though this was less defined than in whole cadavers (Fig 13G and 13J). Whether we infected the insects naturally or via injection we observed no fungal growth on isolated cuticles stripped of fat body and epidermis, showing that the cuticle by itself does not provide sufficient nutrients for fungal outgrowth.

**Fig 14:**
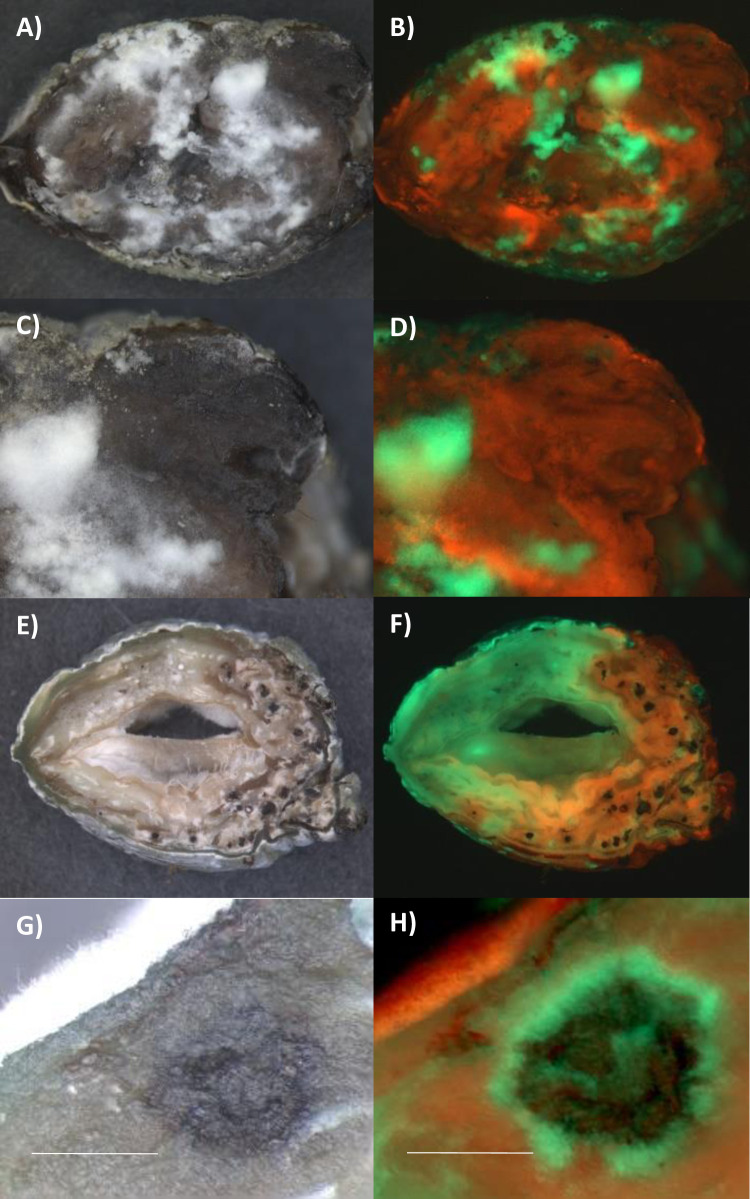
The melanin-based immune response of fifth instar *M*. *sexta* larvae injected with Cherry labelled Mr2575 and GFP-labelled Ma549. A) bright field and B) overlay of Cherry and GFP of a cadaver section 4 days postmortem. The cadaver was sectioned one day in advance of these pictures and the sections placed on water agar to allow fungus to grow out. Ma549 grew more abundantly than Mr2575. The area in the circle is shown in higher magnification in C) bright field and D) overlay of Cherry and GFP showing a darkly melanized region containing predominately Mr2575. E, G, I) bright field and F, H, J) corresponding overlays of Cherry and GFP of the preceding bright field image showing that following injection of conidia melanized nodules form within the hemolymph. These frequently had haloes emitting strong GFP signals indicating that Ma549 can survive and breakthrough the encapsulation. The respective GFP and Cherry images comprising the overlays are shown in S4I Fig along with an additional example of a cadaver section with melanized domains containing Mr2575. Bar = 50 μm.

Cross sections of mummified cadavers also showed unequal darkening. We incubated these sections overnight to allow fungal growth (Fig 14). Ma549-GFP grew more profusely than Mr2575 and derived mostly but not exclusively from less melanized tissues whereas Mr2575 was mostly in darkly melanized tissues (Fig 14A–14D). Cross sections of larvae injected with conidial suspensions usually contained dark (melanized) nodules that were easily visible against the fluorescent background (Fig 14E–14H). Nodules are a well-characterized cellular response to infection formed from insect hemocytes aggregating around microbes and releasing immune enzymes, including phenoloxidases [29]. Much higher levels of nodulation were observed following injection of spores into insects, relative to natural infections, and occurred throughout the infected insect, providing evidence that the *M*. *sexta* melanin-based immune response is activated against both Ma549 and Mr2575 spores. Nodules were counted in sections of the seventh abdominal segment; they were more abundant in caterpillars injected with Ma549 (17.3 ± 1.5189, N = 20) than Mr2575 (11.95 ± 1.048, N = 20) (t = 3.43, p = 0.0014) (Table K S1 Data). Hydrophobic cells are more likely to be encapsulated [30]. A phase exclusion assay was used to compare hydrophobicity (Table L in S1 Data). With this technique spores that migrate to an organic layer are more hydrophobic than spores left in an aqueous layer. Approximately 45% of Ma549-gfp conidia stayed in the water fraction compared to 65% of Mr2575-cherry conidia (Kruskal-Wallis H = 21.77, P< 0.00001). Consequentially, Ma549 conidia are significantly more hydrophobic than those of Mr2575. However, in larvae injected with both Mr2575 and Ma549 we found that nodules were much more abundant in zones dominated by Mr2575 (Fig 14E and 14F), though in many cases we observed green fluorescent growth surrounding nodules indicating that at least some melanin-encapsulated Ma549 survive in Mr2575 dominated territories and break through the encapsulation (Fig 14G and 14H).

#### The impact of melanization on Ma549 and Mr2575

**Fig 15:**
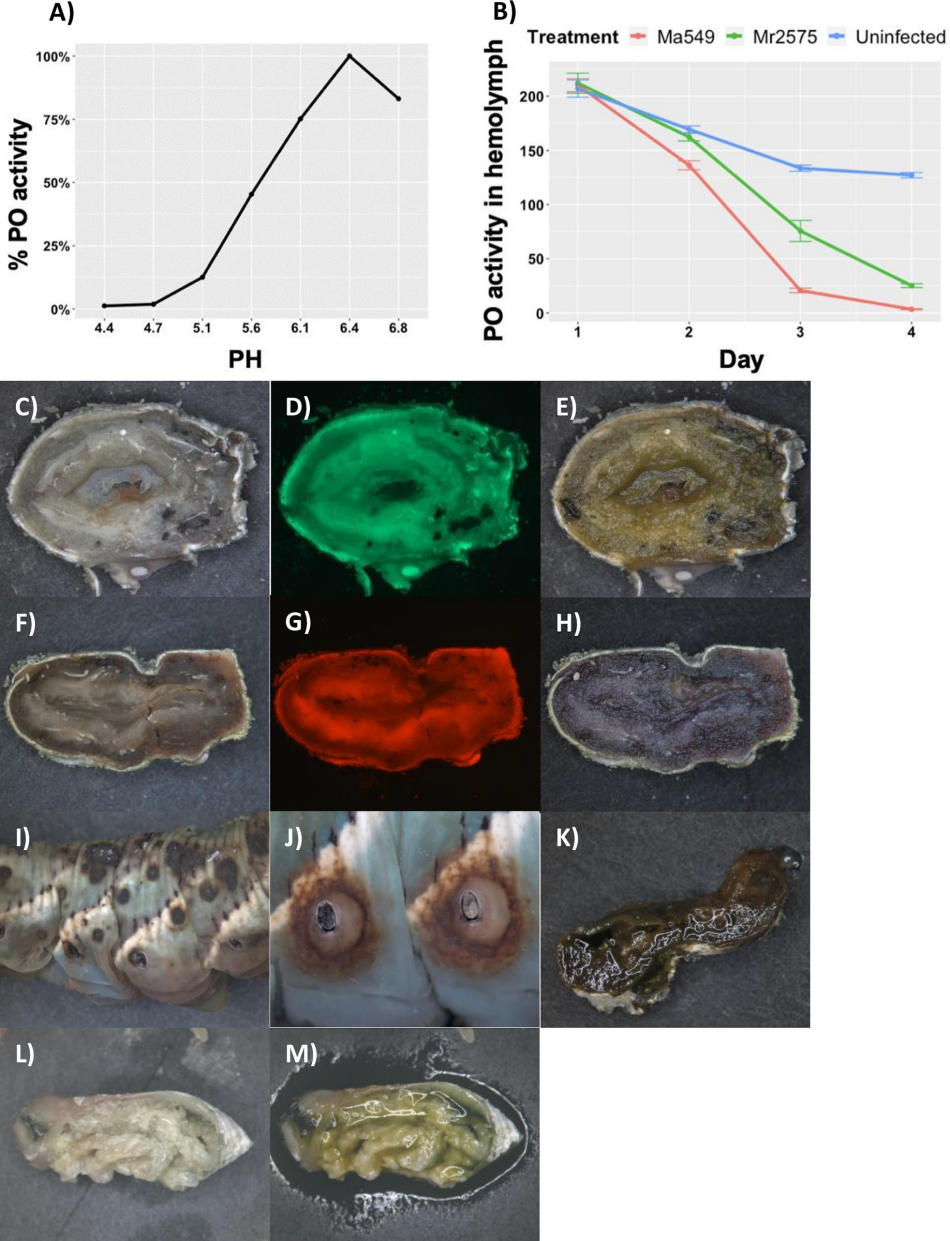
The melanin-based immune response of fifth instar *M*. *sexta* larvae. A) pH optimum of *M*. *sexta* phenoloxidase (PO) activity was ~6.5 with very little residual activity below pH 5. B) Time course of PO activity in hemolymph showing that Ma549 is more effective than Mr2575 at reducing PO activity. C) and F) bright field images of cadaver cross sections 6-days post infection with Ma549-GFP (C) and Mr2575-Cherry (F), respectively, showing larger number of nodules in the Ma549 infected caterpillar although the Mr2575 killed caterpillar was darker overall. D) GFP and G) Cherry fluorescence of the sections shown in C and F. E) and H) the sections shown in C and F stained with Bromocresol purple demonstrates the Mr2575 colonized cadaver has a pH ≥ 6.8 while the Ma549 cadaver has a pH of ≤ 4.5. I) Infection with mutant Mr2575 Acid + that overexpresses oxalic acid caused very extensive melanin patches on the cuticle, J) like WT Mr2575 Acid + frequently infected in the vicinity of the spiracles but little darkening post-mortem as compared to its parent Mr2575. Darkening postmortem occurred in Acid + infected caterpillars that stained violet with bromocresol purple (K) whereas no melanization occurred in caterpillars that stained yellow with bromocresol purple (L and M).

Melanin has been appreciated as a key part of the insect immune defense against pathogens for several decades though curiously we could not find a published pH optimum for *Manduca* phenol oxidase (PO). We determined that the pH optimum of *M*. *sexta* PO was ~6.5 with very little residual activity below pH 5 (Fig 15A and Table N in S1 Data). Consistent with this the pH of the hemolymph of uninfected caterpillars was approximately 6.7, and it remained constant at 6.7 in caterpillars infected with Mr2575 or Ma549 until post-mortem. PO activity decreased in non-infected caterpillars as they enlarged (Fig 15B and Table N in S1 Data), perhaps because PO production was not keeping pace with the increased volume of hemolymph. However, PO declined particularly sharply in infected caterpillars and was negligible in caterpillars infected with Ma549 in the day preceding death (Fig 15B and Table N in S1 Data).

Post-mortem, the pH dropped to about 4.5 in 3 out of 11 cadavers colonized by Ma549; all three had carried a large fungal load preceding death and none showed signs of internal melanization. The drop in pH could be detected using bromocresol purple which turned yellow (pH <5.2) applied to these Ma549 cadavers (Fig 15C–15E) but stayed violet (pH > 6.8) in all larvae infected with Mr2575 (Fig 15F–15H). Ma549 is known to produce more oxalic acid than Mr2575 [31] and the acidification of the cadaver by Ma549 might block melanization. However, the Mr2575 constitutive oxalic acid over-producing mutant Acid + caused very dark and extensive melanin patches on the cuticle (Fig 15I), that like the parent Mr2575 often involved the spiracles (Fig 15J). Darkening post-mortem occurred in Acid +-infected caterpillars that stained violet with bromocresol purple (Fig 15K) whereas no melanization was noted in caterpillars that stained yellow with bromocresol purple (Fig 15L and 15M).

Melanin is believed to kill microbes through the production of toxic intermediates and oxidative damage [32]. To determine if melanization killed Ma549 or Mr2575, we incubated spores of Ma549 or Mr2575 for 18 hours with melanizing *Manduca* hemolymph bled from fifth instar larvae. This melanizing milieu allowed >99% germination and hyphal growth of both Mr2575 and Ma549 despite the hemolymph becoming black and precipitating large melanin granules. This result suggests that immune melanization does not inhibit the growth of *Metarhizium* strains *in vitro*. Likewise, melanin made by mixing dopamine with *Manduca* PPO did not inhibit mycelial growth when added to PDA plates (Table P in S1 Data). Nor was growth reduced on PDA amended with 2mM l-dopa or dopamine. However, in four days the melanin precursor 5,6-dihydroxyindole (DHI) reduced growth of Mr2575 by 40% from 17.6 ± 0.22 mm to 10.53 ± 0.148 mm (t = 26.47, p < 0.00001, N = 20), and Ma549 by 16.4% from 11.26 ± 0.14 mm to 9.41 ± 0.145 mm (t = 9.16, p < 0.00001) (N = 20) (Table P in S1 Data).

#### Yields of conidia

We evaluated the effect of mixed Ma549 and Mr2575 infections on their transmission potential by calculating spore production in single and mixed infections. At high humidity, fatal infections by Ma549 always resulted in a cadaver covered in sporulating hyphae with an external conidial yield of 3.75 x 10^8^ ± 0.25 x 10^8^ per g of cadaver (N = 20) (Table Q in S1 Data). Caterpillars topically infected with Mr2575 that became deeply melanized and contained bacteria in the hemolymph did not support sporulation well, and spores were usually localized in small patches (S5 Fig); we did not attempt to quantify these. We focused on the ~ 70% of caterpillars killed by Mr2575 that showed extensive sporulation over the entire cadaver, although even with these the yields (1.77 x 10^8^ ± 0.11 x 10^8^ spores per g of cadaver) were ~ 50% lower than Ma549 (Kruskal-Wallis test: H = 14.28, p = 0.00016). Caterpillars co-infected with Ma549 and Mr2575 either by the natural route or by injection produced discrete non-overlapping territories of spores. The yield of Ma549 coinfected with Mr2575 by the natural route was 3.34 x 10^8^ ± 0.20 x 10^8^ per g of cadaver which was similar (H = 0.7032, p = 0.402) to the yield produced by pure Ma549 showing that it is little influenced by the presence of Mr2575. However, the yield of Mr2575 was reduced 115-fold (2.03 x 10^6^ ± 0.43 x 10^6^ spores per g of cadaver) compared to Mr2575 applied alone.

The roughly equal representation of Ma549 and Mr2575 in cadavers following injection resulted in a mixture of 3.02 x 10^8^ Ma549 + Mr2565 spores per g cadaver, that was 11.1% less (H = 4.01, p = 0.04532) than the yield from single infections with Ma549. Compared to single infections, mixed genotype yields were reduced by 47.7% (Ma549) and 37% (Mr2575) indicating that the strains are competing. However, the yield of Mr2575 spores was significantly more than half (H = 4.92, p = 0.02655) the yield of single infections despite sharing the cadaver with the high yielding Ma549. This may be because of resource partitioning if the spatial localization of Mr2575 occurred in areas of the cadaver favorable to its spore production. There was a moderately negative association between yields of Ma549 and Mr2575 (r = -0.733, p = 0.00236), explainable by the finite surface area of the cadaver for sporulation.

#### Colonization of *Manduca* cuticles

**Fig 16:**
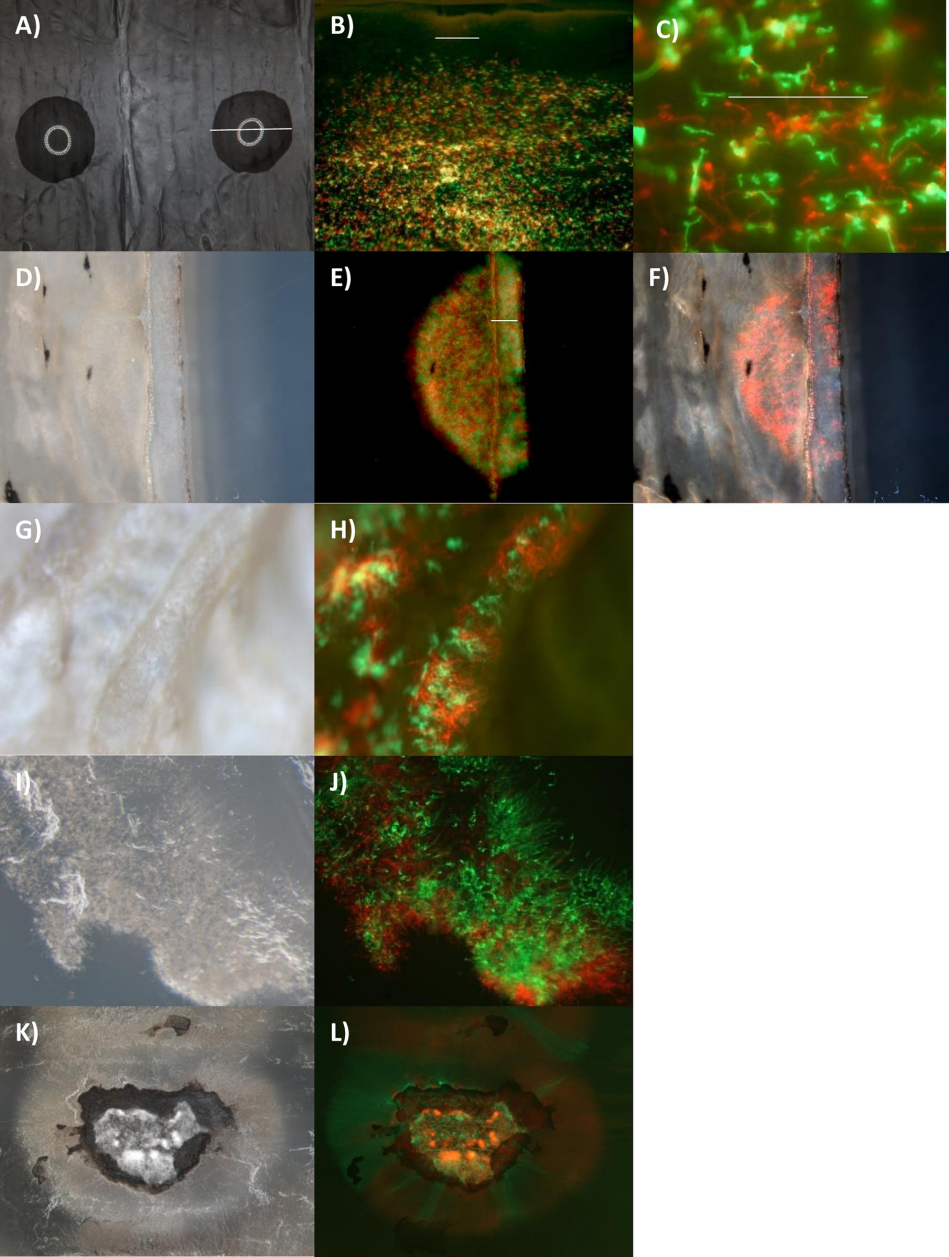
Colonization of isolated fifth instar *M*. *sexta* cuticles placed on agar. A) Fungal spores were applied in 2 μl water droplets to cuticle surfaces. The diameter of the droplet indicated by the white bar was 3.7 mm. The cuticle in this picture had been soaked overnight in 1% l-DOPA causing melanization. B) and C) Mr2575-Cherry and Ma549-GFP spores germinating on cuticles 12 hrs. post application (bar = 100 μm). Cuticles were sectioned at an oblique angle in the middle of the droplet application sites. D) bright field, E) overlay of Cherry and GFP and F) overlay of Cherry, GFP and bright field of section 24 hrs. post application showing penetration of the cuticle. The bar in E) shows the width of the cuticle estimated to be 0.6 mm. G) bright field and H) overlay of cherry and GFP showing penetrant hyphae growing over the underside of the cuticle 28 hrs. post application. I) bright field and J) overlay of Cherry and GFP showing Mr2575 and Ma549 growing through the agar underneath the cuticle 32 hrs. post application (penetrant hyphae were attaching the cuticle to the agar so that when the cuticle was lifted a plug of agar remained attached to the cuticle. These photos show fungus lining the resulting hole). By 36 hrs. post application the cuticle was disintegrating. K) bright field and L) overlay of Cherry and GFP showing the application zone remaining attached to the agar when the cuticle was lifted. Cherry labelled Mr2575 and GFP-labelled Ma549 are growing down through the agar. The respective GFP and Cherry images comprising the overlays plus other examples are shown in S4J Fig.

Attachment to the cuticle surface and penetration rate might also influence the competitive ability of *Metarhizium* strains [13]. It is possible that the order in which Ma549 and Mr2575 infect could allow Ma549 to establish in hosts faster. Cuticles were isolated from fifth instar larvae and placed on 1.5% water agar. We infected isolated cuticles with spores of either or both Mr2575-cherry and Ma549-GFP suspended in 2 μl drops of water that beaded up on the hydrophobic epicuticle (Fig 16A). Growth on and through the cuticle was monitored for 72 hours. Mr2575 and Ma549 both germinated within 10 hours on the cuticle (Fig 16B and 16C), and breached isolated cuticles simultaneously about 26 hours post inoculation (Fig 16D–16F) to grow over the undersurface of the cuticle (Fig 16G and 16H). This similar performance suggests that the behavior of the fungus post cuticle penetration determines their different strategies and relative abundance in the hemolymph. We found no difference in penetration of cuticles pre-treated with PTU (to inhibit melanization) and cuticles soaked for 24 hours with l-DOPA. L-DOPA soaked cuticles were jet black (Fig 16A), but melanization only extended for a couple of millimeters through the undersurface of the endocuticle. Cuticles colonized by either fungus for 48 hours were attached to the agar by an extensive growth of penetrant hyphae; when the cuticles were pulled off the agar, they frequently took a plug of agar with them (Fig 16I and 16J). By 72 hours the cuticles disintegrated when they were lifted, leaving some of the cuticle attached to the agar (Fig 16K and 16L), consistent with degradation of cuticle polymers.

## Discussion

Interactions in cultures do not predict how strains interact in the host. Mr2575 grew faster *in vitro* and produced more blastospores in nutrient-rich SDB than Ma549. Conversely, Ma549 produced far more blastopores than Mr2575 in hemolymph, and blastospore production in insects is a better predictor of competitive ability during mixed infections of fifth instar *M*. *sexta* than growth on or through the cuticle, *in vitro* growth rate, or toxin production. We found no evidence of chemical antagonism between Mr2575 and Ma549, although a previous study indicated that Mr2575 represses germination and differentiation of the insect pathogen *Beauveria bassiana* in insects, possibly through anti-competitor toxins, although *B*. *bassiana* dominates *in vitro* cultures [10].

Both Mr2575 and Ma549 are generalists, but they allow for comparison between a strain that proliferates within a living host (Ma549) and a toxin-producing strain that proliferates after a host has died (Mr2575). The availability of diverse fluorescent markers for tagging strains allowed us to directly monitor their distinctive interactions with hosts and with each other in a manner that was not previously possible. Plant pathogenic bacteria have a resource allocation trade-off between virulence factor production (enzymes, toxins) and proliferation in hosts [33], and by this criteria Ma549 and Mr2575 have arrived at a different allocation of toxins and growth. Our results quantified previous observational studies with Ma549 and Mr2575 [14] and showed that following natural infection of *M*. *sexta* larvae, Mr2575 remains localized to melanized infection sites until the host is moribund, whereas Ma549 rapidly disperses through the living host. Theory predicts that generalist pathogens are more likely to find themselves in mixed infections than specialists, exposing them to any transmission costs [34]. The Th1-Th2 trade-off is a key paradigm of within-host interactions which posits that the presence of one parasite species enhances the survival of another parasite by reducing the impact of the host immune response [35]. Mr2575 is closely related to *M*. *robertsii* ARSEF 23 (Mr23) which also produces destruxins [36]. A previous study showed that co-infections of wax moth larvae (*Galleria mellonela*) with Mr2575 and Mr23 produced insect survival curves that were intermediate to those of single infections [10]. We obtained a similar result with mixed Ma549 + Mr2575 co-infection in male *Drosophila*, whereas in female *Drosophila* and fifth instar *M*. *sexta* larvae, the longevity of the host was not significantly different from that of infection with the least virulent Mr2575. In these examples the Th1-Th2 trade-off has no noticeable effect, as the outcome of infection appears to be dependent on the less aggressive genotype. A possible explanation is that the recognition of Mr2575 leads to a faster and stronger host response that also impacts Ma549.

We previously demonstrated that Ma549 and Mr2575 elicit similar levels of the *Drosophila* anti-microbial peptide drosomycin [13]; however, in our current study, we found that Mr2575 elicits a stronger melanization response in *M*. *sexta*. At least one intermediate of the melanization cascade (dihydroxyindole) inhibits the growth of both Ma549 and Mr2575, and the most melanized parts of a fifth instar cadaver are the least supportive of growth. Similarly, we previously found that Mr2575 exhibited poor growth on melanin-cuticle complexes owing to its toxic effects and the ability of melanin to shield the cuticle from enzymatic attack [37]. However, in contrast to opportunistic insect pathogens such as *Candida albicans* and *Cryptococcus neoformans* [38] we found no evidence that melanization directly kills Mr2575 and Ma549 during immune reactions within the insect.

Unlike *M*. *sexta*, we found no evidence that *Drosophila* would disproportionally transmit the more virulent Ma549, but females were more likely than males to show distinct territories of Mr2575 localized to thoraxes. Male *Drosophila* are generally smaller than females and better able to limit virulence and pathogen proliferation [12], suggesting that they may be a more difficult resource to exploit and, potentially, these fungi may find it more difficult to establish territories in male’s pre-mortem. Blastospores provide Ma549 with a competitive advantage in the hemolymph of *Drosophila* and *Manduca* and may contribute to Ma549 killing both species faster than Mr2575. However, large areas of co-infected *Drosophila* cadavers become covered in Mr2575 spores, suggesting that sporulation (transmission potential) is weakly linked, if at all, to proliferation in the living fly. We tested whether host body size could influence the ability of Ma549 and Mr2575 to establish territories using different instars or taxa of insects. Like *Drosophila*, Mr2575 and Ma549 appeared to be roughly equally competitive on first instar *M*. *sexta* caterpillars, indicating that blastospore production is a specific adaptation to increase competitive abilities for pathogen transmission during co-infection of large hosts. This suggests that small insects are more likely than large insects to act as reservoirs for pathogen diversity, at least in the case of Mr2575 and Ma549.

Under natural infections, Mr2575 is unable to substantially establish itself in large *M*. *sexta* larvae if rapidly proliferating Ma549 is present, presumably because the Ma549 strategy of producing blastospores favors faster dispersal and exploitation rates of large hosts as it competes with Mr2575. In other words, Ma549 being established throughout the host, there is less space left unoccupied for Mr2575. This is akin to the “priority effect,” where a parasite that infects a host first can have a relative advantage [39]. Similarly, Ma549 rarely encroached on melanized areas colonized by Mr2575 during its initial infection, suggesting that infection sites are a sanctuary for Mr2575 unaffected by Ma549 proliferation in the hemocoel and this allows Mr2575 to be competitive in small insects. The life history traits of Ma549 and Mr2575 were similar in single and mixed infections of *Drosophila* and *Manduca*, with localized Ma2575 during the life of the insect and the rapid colonization of living insects by Ma549 blastospores. This suggests that Ma549 and Mr2575 do not respond in a plastic manner to a host genotype, or to whether they are in a single or mixed infection of a large or small insect, even though host genotypes and phenotypes have a strong effect on the outcome of competition.

Proliferation in living fifth instar *M*. *sexta* is a strong anti-competitor behavior for Ma549, as it predominates in mixed topical infections, and Mr2575 infected caterpillars often carry bacterial and yeast loads in the hemolymph which we did not observe with Ma549. This suggests that high virulence in single infections resulting from toxin production does not confer a highly competitive ability. Injecting destruxin A (the principal destruxin produced by Mr2575) into *Drosophila* specifically suppresses the production of antibacterial peptides, thereby increasing susceptibility to bacterial infections [40]. Perhaps by selectively reducing antibacterial peptides, Mr2575 creates an environment in which bacteria can proliferate, thereby accelerating the demise of the host. The production of toxins capable of killing many insects allows a fungus to be successful against a broad range of potential hosts without the need to adapt to the defenses of a particular insect. Thus, a toxin-producing strategy with necrotrophic post-mortality proliferation might be selected if it enables diverse insects to be killed before their immune systems are activated. However, Mr2575 activated melanization in fifth instar *M*. *sexta* and was not as successful as Ma549 in suppressing it.

Competitive differences between Ma549 and Mr2575 did not relate directly to toxin production, as bypassing the cuticle by direct injection of mixed spores produced a much less one-sided infection. On semi-solid agar and perhaps in insect tissues, as these are also semi-solid, colonies of Mr2575 and Ma549 coexist as discrete individuals with each strain having a competitive effect on itself and the other strain, so they cease to expand at their boundary and produced a spatially structured community. A similar phenomenon was observed between colonies of Mr2575 and the root colonizing endophyte *Trichoderma atroviride* [41] suggesting that these opportunistic fungi may be adapted to stably co-exist with diverse competitors in several habitats.

Injecting mixed Ma549 and Mr2575 conidial populations into the hemolymph produces a homogenous distribution of Ma549 and Mr2575 throughout the hemocoel. Nevertheless, we found that following death, there was a striking segregation of Mr2575 at the front of individual segments and Ma549 at the rear of the segments. This presumably reflects some environmental variation between these locales that impacts the local growth rates and survival of Ma549 and Mr2575 differently. We are not aware of any previous evidence of an anteroposterior chemical gradient within each segment or of any features of caterpillar tissues that particularly favor colonization by Ma549 or Mr2575. Given the open circulatory system of *Manduca*, it is unlikely that chemical gradients (e.g., oxygen, pH, or redox) could sharply constrain Mr2575 at intersegments. Furthermore, Ma549 and Mr2575 showed similar declines in growth under 5% hypoxia (5% oxygen and 95% nitrogen) and 1% hypoxia (1% oxygen), indicating that Mr2575 does not require more oxygen than Ma549 does.

Natural single-strain infections with Mr2575 are often localized to areas around the spiracles and major trachea indicating that Mr2575 does not shift the use of host tissues in response to Ma549. Mr2575 was also localized around the spiracles and trachea when spores were injected, which would not have been because the spiracles provided an infection route, although the spiracles could provide a structural focus that a fungus could orientate too. Segmental structures also include the dorsal aorta (heart), which forms a series of chambers roughly under the V-shaped arrangement of dorsal spots where Mr2575 aggregates. However, cuticles and underlying epidermis dissected from injected caterpillars retained segregation of Mr2575 and Ma549 in the absence of the heart and trachea, suggesting that if segmental structures are responsible for initiating segregation their presence is not required to maintain it.

The roughly equal relative representation of Mr2575 and Ma549 in cadavers of injected fifth instar *M*. *sexta* was expected to result in mixed genotype yields, lying somewhere between the yields caused by pure Mr2575 and Ma549. However, niche partitioning could reflect resource partitioning between Mr2575 and Ma549 in the segments, which could result in increased total fungal yields owing to the more complete conversion of host resources into spores. We found that mixed genotype yields were 11% lower than those for Ma549 alone, the highest yielding single genotype. However, the Mr2575 yield in mixed infections was significantly higher than that predicted based on single infections, suggesting that Mr2575 was spatially localized at sites it was adapted to utilize.

Mr2575 and Ma549 grew equally well on insect cuticles and breached isolated cuticles simultaneously, suggesting that the subsequent behavior of the fungus determines their different strategies. Ma549 was much more likely to produce swollen cells on cuticle extracts, mimicking infection morphologies such as appressoria and blastospores, whereas Mr2575 grew as long hyphae in several nutrient-poor media that supported less germination and growth of Ma549. Mr2575 also germinates in response to lower levels of nutrients than plant endophytic *Trichoderma* spp., perhaps as an adaptation that allows rapid colonization of plant roots before competitors [17]. The production of far-reaching explorative hyphae could be a beneficial strategy in a patchy, ephemeral resource landscape [42] allowing the fungus to travel from insect cadavers to plant roots and cover root surfaces despite diverse competitors. It might also facilitate the spread of Mr2575 over cadavers if it is constrained to its initial infection sites by competitors. From the perspective of Mr2575, its beneficial associations with plants and virulence to insects are simply a means of establishing a nutritional relationship with these hosts; Mr2575 spores applied to *M*. *sexta* larvae are not infectious when provided with a supplementary nutrient source [43].

*Primae facie*, selection should favor the entomopathogen strain that produces the most spores on a host in a mixed infection. Thus, blastopore production and virulence of Ma549 may be selected based on how it affects sporulation on cadavers. If a *Metarhizium* strain is frequently exposed to conspecific strains during infection that will likely impose selection pressures for stronger competitive abilities. It also seems feasible that large hosts will accumulate more mixed infections than small hosts. Thus, selection could favor *Metarhizium* genotypes that retain high levels of blastospore production in large hosts. However, it is unclear whether predictions of transmission-virulence trade-offs in insects hold for the known plant endophyte Mr2575, as it is at least partially disconnected from the link between virulence to insects and transmission. Instead, the reproductive output of Mr2575 may be tied to colonizing plant roots, Mr2575’s principal habitat, where it is likely to be surrounded by diverse root-feeding insects that it has greater access to than non-root colonizers. If Mr2575 faces conflicting selection pressures on insects and plants, then virulence on insects likely evolved depending on the way that entomopathogenicity affects changes in transmission to plants (its long-term host) [6].

We lack adequate knowledge of the extent to which different *Metarhizium* strains associate with different plants in nature and the mechanisms by which these associations occur. For example, if a fungus proliferates chiefly on plant roots will that relax selection on the ability to sporulate on cadavers? Although *M*. *anisopliae* is a root colonizer under laboratory conditions, in nature *M*. *robertsii* and *M*. *brunneum* often predominate in soils [44]. *M*. *robertsii* and *M*. *brunneum* have primarily Holarctic distributions [45], with seasonal environments that may require adaptation to abiotic conditions and roots. Ma549 belongs to a lineage of *M*. *anisopliae* that is prevalent throughout Brazil [46]. This lineage is the most prominent *Metarhizium* pathogen of insects in Brazil, whereas *M*. *robertsii* is primarily isolated from soil [47]. Therefore, although both *M*. *robertsii* and *M*. *anisopliae* colonize plant roots in the laboratory, it has not yet been confirmed that roots are a major habitat for the Ma549 lineage in nature.

This study raises several additional issues that require further investigation. The unexpected partitioning of insects by Ma549 and Mr2575 requires explanation. Is it a common feature of entomopathogenic fungi, only fungi that vary in the production of toxins and/or blastospores, or is it unique to Ma549 and Mr2575? We do not have an explanation as to why the Acid + Mr2575 mutant elicited more extensive melanization of the cuticle than wild type Mr2575, although this phenomenon might explain why Ma549 acidifies cadavers rather than living insects. We did not establish the cause or effect as to whether the bacterial contaminants in many Mr2575 infected caterpillars contributed to the desiccation of the cadavers or were the result of desiccation. Overall, the possibility that some fungi may be adapted to partner with other microbes to overcome insects is worth investigating, particularly if this was mainly a feature of fungi for which monopolizing a host is a reduced priority because of a principal habitat on plants.

## Methods

### Organisms and growth

*M*. *anisopliae* ARSEF 549 (Ma549) is the active ingredient in the commercial product Metabiol; it was originally isolated in Brazil from a spittlebug *(Mahanarva* spp.). *M*. *robertsii* ARSEF 2575 (Mr2575) was isolated from the pecan weevil *Curculio caryae* in North Carolina, USA. Ma549 and Mr2575 were obtained from the U.S. Department of Agriculture Entomopathogenic Fungus Collection in Ithaca, N.Y. We also used a chemically mutagenized Mr2575 mutant, Acid (+), that produces 30-fold higher levels of oxalic acid compared to the wild type 2575 (7.5 mg ml^-1^ versus 0.23 mg ml^-1^), but other organic acids were not detected [31]. Cultures were maintained on potato dextrose agar. To facilitate studies of strain differences, we used Ma549 and Mr2575 strains that express green fluorescent protein (GFP), mCherry or dsRed obtained in previous studies and selected based on WT growth in culture and WT levels of virulence [7, 13].

For inoculation into the liquid media [Sabouraud dextrose broth (SDB)], conidia were harvested from plates, filtered through glass wool, and spore concentrations were determined by direct count using a Neubauer hemocytometer. Liquid broth cultures were inoculated with 5 x 10^5^conidia/ml. Cultures were grown in shake cultures (250 rpm) at 27.5°C, and samples examined microscopically at various intervals.

### Sources, treatments, and rearing of *Manduca sexta* larvae

*Manduca sexta* eggs and commercial pre-mixed diet were obtained from Carolina Biological Supply Company. Eggs were placed in petri dishes along with diet and incubated in an insect incubator with a 16-h/8-h light/dark photoperiod at room temperature. The larvae were individually housed in 150 ml cups (7 cm diameter) after the second instar and maintained at room temperature (21–24°C) with a 12 h light: 12 h dark light cycle. Food was provided *ad libitum*. All experiments were initiated one day after the molt to the fifth instar. Animals were matched by weight to the different treatment groups.

### Topical natural-route inoculation

To prepare inoculum, conidia were harvested from 10-14-days-old potato dextrose agar plates, suspended in sterile distilled water, vortexed for 2 minutes and filtered through Miracloth (22–25μm) (Andwin Scientific) to remove mycelia. Spore concentrations were determined using a Neubauer hemocytometer and adjusted to the required concentration with water.

Protocols for infecting *Drosophila melanogaster* DGRP line 808 are described in [12,19]. Flies (2-4-days old) were vortexed with spore suspensions (20 ml, 2.5x10^4^ conidia/ml) for 30 seconds, collected by filtering the suspensions through Miracloth, and transferred into vials containing fresh food (5% sucrose/2% agar media). Flies were cultured at 27°C, ~85% relative humidity. Each vial contained at least 25 flies with five replicates per sex. The survival of the flies was monitored every 12 hours up to 14 days after infection. Data from all replicates were pulled together, and the log-rank test (Mantel-Cox test) was used to evaluate differences between treatments. The log-rank test was also performed between each treatment.

*Sarcophaga bullata* pupae were obtained from Carolina Biological Supply Company. Once the adults emerged, they were kept in a gauze cage and supplied with sugar water *ad libitum*. Flies (2-4-days old) were chilled at 4°C for five minutes and then infected with a spore suspension (total concentration: 5x10^6^ spores/mL) prepared from 8- to 15-day-old cultures of. Batches of seven *S*. *bullata* were submerged in 20 mL of spore suspension, vortexed for 10 seconds, then transferred into plastic cups with sugar and water. They were maintained at 27°C with 85% relative humidity under a 12-hour light-dark cycle.

Newly hatched first instar *M*. *sexta* larvae (weighing ~1mg) were inoculated with a 10 μl drop of conidial suspension in water. Most caterpillars crawled out of the drop and as they did so were picked up by their horns and placed on a damp filter paper for 5 hours before placing on diet. First-day fifth instar *M*. *sexta* larvae (weighing 1 to 1.5 grams) were inoculated by dipping the larvae briefly into conidial suspensions in 0.01% Tween 80. Controls were dipped in 0.01% Tween 80 or autoclaved (dead) spores in 0.01% Tween 80. All inoculations were conducted 1–2 hours before lights off. Fifth instar larvae were starved overnight to allow germination of the conidia (the diet contained antifungal compounds) and then returned to their normal food. Three replicates were performed for each treatment with at least eight *Manduca* for each replicate.

### Inoculation by injection of *M*. *sexta*

Prior to either injection or bleeding, larvae were chilled on ice for 30 min and the cuticle surrounding the puncture site was disinfected using 70% ethanol. In all cases larvae were injected with 15 μl of 5 x 10^5^conidia/ml in phosphate-buffered saline (Sigma), at an angle (less than 45°) behind one of the abdominal prolegs using a 30-gauge needle attached to a 25 μl Hamilton syringe. The needle was sterilized by rinsing 3 times each in 2 tubes of 95% ethanol, followed by one tube of sterile H_2_O between uses.

Whether infected topically of by injection, all infected *M*. *sexta* and their controls were maintained at 25°C under a 17 h light and 7 h dark photoperiod. Insects were weighed daily at 5–7 hours after lights-on, and assessed for symptoms of infection, including loss of responsiveness (measured as the time it took for a larva to right itself after being turned over). and mortality (when the larva did not respond to prodding).

### Harvesting hemolymph from *M*. *sexta* larvae

Hemolymph was removed from anesthetized larvae (chilled on ice for 15 min), by making an incision below the horn. Larvae were held over a sterile chilled Eppendorf to collect the hemolymph. In some experiments, the Eppendorf’s contained crystals of phenylthiourea (PTU) to inhibit melanization, and in some experiment’s hemolymph samples were centrifuged at 7,500 for 15 min at 4°C and the cell free supernatants stored at -80°C.

### Cuticle collection and dissection

Five-day fifth instar *M*. *sexta* larvae were dissected by cutting along the dorsal midline and removing hemolymph, gut, fat body and for some experiments the muscle layer and epidermis. For some experiments, cuticles were gently stirred overnight in 5 mM l-DOPA to melanize. Alternatively, cuticles were washed twice in 0.002% PTU, and stored at −80°C. Fungus-killed larvae were sliced into sections using a razor blade. Melanized dark nodules within the seventh abdominal segment were counted using a fluorescence stereomicroscope (Zeiss SteREO Discovery. V12). This same microscope was used for most of our pictures of intact cadavers.

### Estimates of spore yields

Sporulation on cadavers was estimated 11 days post death (cadavers disintegrated after this time). Pre-weighed cadavers were transferred to moist chambers to encourage sporulation. Conidia from each caterpillar were harvested by shaking in 2 ml of 1% Triton X-100 in 25 ml plastic centrifuge tubes. After 20 minutes, a loop was used to gently scrap remaining conidia of each cadaver, and the cadaver was removed. The suspension in the tube was vortexed to break up any spore clumps, and a preliminary spore count made using an improved Neubauer hemocytometer (Weber Scientific International Ltd., UK). If a preliminary sample had more than 100 conidia per counting chamber, each stock suspension from that day was serially diluted. After all suspensions were brought into the range 20–100 conidia, the final hemocytometer count was replicated three times. The number of spores per gram of caterpillar was estimated as the weighted yield. For co-infections, total numbers of conidia were estimated with a hemocytometer, and the relative ratio of GFP-Fungus to Cherry/dsRed-fungus was calculated using a fluorescence microscope (Zeiss MicroImaging GmbH 37081). The Kruskal-Wallis test calculator was used to test for significant differences in sporulation loads.

### Behavior of *M*. *anisopliae* and *M*. *robertsii* germinating against plastic or glass

To test the effects of nutrients on strain differentiation against a smooth hydrophobic surface, conidia were induced to germinate by growing them in 5.5-cm polystyrene petri dishes containing 2 ml of water or water supplemented with yeast extract (YE at 0.0125 or 0.1%), root exudate (RE at 0.0125 or 0.1%) or *Manduca* cuticular surface lipids. Plant root exudates were obtained by culturing wheat roots in sterile water for 30 days. The exudate was freeze-dried and resuspended in distilled water to obtain 0.1% and 0.0125% RE solutions. To obtain cuticular lipids, groups of 5 or more 3-day fifth instar *M*. *sexta* larvae were killed by freezing, freeze-dried, immersed in three- to fourfold excess of dichloromethane, and gently shaken for 10 min to extract cuticular lipids [48]. All types of lipids tend to dissolve in dichloromethane, which is of intermediate polarity. The extracts were collected in glass petri dishes, dried in a hood, and suspended in absolute ethanol. Samples of the ethanol suspension were evaporated on preweighed aluminum dishes to determine the dry weight of extract and stored at −20°C.

Various amounts of each extract were pipetted onto glass coverslips and evaporated, leaving a white greasy layer on one side. The coverslips were then placed in the polystyrene petri dishes with the lipid layer facing up, and the petri dishes inoculated with spore suspensions of Mr2575 and/or Ma549.

We modified Riddel’s [49] method of slide culturing to examine growth against a dry glass surface. Two small cubes (0.5 cm x 0.5 cm) of agar containing dichloromethane cuticle extract (100 μg ml^-1^) were cut from poured plates and transferred to sterile slides about 0.2 cm apart. The four sides of each block were inoculated with Ma549 or Mr2575 and a flamed cover slip applied centrally upon the agar bocks. The slides were incubated at 27°C on glass rods over filter paper soaked in 10% glycerol to maintain high RH.

The anti-*Metarhizium* activity of being in a melanizing milieu was measured in petri dishes containing one ml of freshly harvested and centrifuged (7,500 for 15 min at 4°C) hemolymph. One hundred spores from each of four replicates were scored microscopically to assess germination frequency. The adherence of conidia or germlings to polystyrene surfaces was determined by applying a jet of water from a pipette and counting the spores retained.

### Measurement of pro-phenoloxidase (pro-PO) Activation

Enzyme activities were determined by microplate assays using a FilterMax F5 microplate reader. The pro-PO activity in 2 μl plasma or filter-sterilized 0.85% NaCl (control), was activated by addition of 1 μg *Micrococcus luteus* at room temperature for 10 min, and then PO activity was measured by adding 200 μl of 2 mM dopamine as substrate in 50 mM sodium phosphate, pH 6.5. Absorbance at 450 nm was monitored continuously using a microplate reader (MolecularDevices). One unit of PO activity was defined as 1,000 × ΔA_450_/minute. Three replicates with four different larval plasma samples were examined. The pH-activity profile for the oxidation of dopamine by *M*. *sexta* hemolymph PO was determined using 0.1 M sodium acetate, pH 4.0 to 5.5, and 0.1 M sodium phosphate, pH 6.0 to 7.0. The reaction was initiated with the addition of hemolymph. PO activity was determined as described above.

### Hypoxia conditions

Petri dishes with PDA spot inoculated with Ma549 or Mr2575 were introduced into a Billups-Rothenberg (del Mar, California) MIC-101 modular incubator chamber which was sealed and flushed for 15 min with a mixture of either 5% oxygen:90% nitrogen or 1% oxygen:95% nitrogen (National Welders). To remove any gases trapped in the media we re-flushed the system after one hour as described in the manufacturers manual. The MIC is designed to maintain constant gas levels for a minimum of 72 hours, but we re-flushed the system every 24 hours. Colony diameters were measured after 72 hours.

### Spore hydrophobicity

The hydrophobicity of Mr2575-cherry and Ma549 GFP conidia obtained from PDA plates was determined by a phase exclusion assay [50]. Conidia were vortexed for 30 sec in 0.2 M tris buffer (pH. 7.0) and filtered through miracloth. The suspensions were adjusted for each sample to give an OD reading of 0.6 at 610 nm and 500 μl aliquots were transferred to microfuge tubes containing 500 ul toluene (anhydrous, 99.8%, Sigma-Aldrich). The mixtures were vortexed for 30 sec and incubated for 30 min RT to achieve phase separation. The percentage of conidia remaining in the water phase compared to the initial OD610 reads were compared using Kruskal-Wallis nonparametric tests. The experiment was repeated three times with spores prepared from different PDA plates.

## Supporting information

S1 Data All data used for statistical analyses and to generate graphs. (XLSX)

S1 Fig Growth of Mr2575 and Ma549 on potato dextrose agar. Top panels: bright field (left) and overlay of cherry and GFP (right) showing 5-day old colonies of Ma549-GFP and Mr2575-Cherry touching each other showing heavy sporulation by Ma549. Bottom panels: bright field (left) and overlay of GFP and dsRed (right) showing hyphae of Mr2575-GFP overlapping the edge of a Ma549-dsRed colony.
(DOCX)

S2 Fig Colonization post-mortem of female and male *Drosophila melanogaster* by Cherry labelled Mr2575 and GFP labelled Ma549. The fungi were applied topically either singly or together and 10 randomly selected male or female hosts from each treatment were visualized at one day intervals with both bright field and epifluorescence, with filters set to detect GFP fluorescence or Cherry. Images are segregated by sex and day. For each sex and day row 1 are flies infected by Ma549 and row 6 are flies infected by Mr2575; rows 2 to 5 mixed Ma549 + Mr2575 infections with row 2 bright field, row 3 GFP, row 4 overlay of Cherry and GFP, row 5 Cherry.
(PDF)

S3 Fig Sporulation on *Sarcophaga bullata* cadavers five-days post-mortem by Mr2575-Cherry and Ma549-GFP. The fungi were applied topically either singly or together and 10 hosts (not segregated by sex) from each treatment were visualized with both bright field and epifluorescence, with filters set to detect GFP fluorescence or Cherry.
(DOCX)

S4 Fig A-J The GFP and Cherry/dsRed images used to generate the overlays in manuscript figures.
(PDF)

S5 Fig A) bright field and B) green fluorescence of Ma549-GFP colony forming units (CFUs) in 2 μl of hemolymph from a larva 4-day post infection. The hemolymph of Ma549-infected insects appeared to be a monoculture of Ma549. C) bright field D) Cherry and E) bright field overlaid with Cherry image of a hemolymph sample from a larva 4 days post infection with Mr2575-Cherry showing a single Mr2575 colony surrounded by bacterial and yeast contaminants. F) Darkened surface appearance of a caterpillar with hemolymph contaminated with bacteria shortly after death (5-days post infection with both Mr2575-GFP and Mr2575-Cherry). G) GFP and H) Cherry images showing light fluorescent zone. I) bright field of zone and J) overlay of Cherry and GFP images seven days post infection showing sporulation of Mr2575-Cherry and Mr2575-GFP.
(DOCX)

S6 Fig GFP images of Ma549 budding blastospores and pseudohyphae in *M*. *sexta* hemolymph four days after topical infection with Ma549-GFP. (DOCX)

S7 Fig Bright field image of cadaver section approximately 6 hrs. post-mortem showing tufts of Ma549 hyphae emerging through the cuticle. B) surface of the same cadaver showing preferential emergence of Ma549 hyphae through hair sockets. C) bright field, D) GFP, E) Cherry, F) GFP/Cherry overlay showing adjacent Ma549-GFP and Mr2575-Cherry colonies on a *M*. *sexta* cadaver approximately 12 hrs postmortem
(DOCX)

S8 Fig Pictures show recent (24 hrs) cadavers of first instar *M*. sexta infected by Ma549-GFP + Mr2575-Cherry. Bright field, GFP, Cherry and overlay showing roughly equal distribution of Mr2575 and Ma549 over cadavers.
(PDF)

S9 Fig Infection of fifth instar *M*. *sexta* larvae by simultaneous topical application of Cherry labelled Mr2575 and GFP-labelled Ma549 showing Mr2575 producing a round clump of non-sporulating aerial hyphae on mature cadavers (following sporulation). This figure supplements Fig 9.
(DOCX)

S10 Fig Images of multiple *Manduca* cadavers infected by Ma549-GFP + Mr2575-Cherry. Bright field, GFP, Cherry and overlays showing segregation of Mr2575 and Ma549 in segments, with Mr2575 producing local melanization. These images are additional examples to Fig 11.
(DOCX)
