# Supplementary material for: Metarhizium fight club: Within-host competitive exclusion and resource partitioning
Source: PLoS Pathog. 2024 Nov 7;20(11):e1012639. doi: 10.1371/journal.ppat.1012639 (PMC11542789; doi:10.1371/journal.ppat.1012639)

**S1 Fig. Growth of Mr2575 and Ma549 on potato dextrose agar.** Top panels: bright field (left) and overlay of cherry and GFP (right) showing 5-day old colonies of Ma549-GFP and Mr2575-cherry touching each other showing heavy sporulation by Ma549. Bottom panels: bright field (left) and overlay of GFP and dsRed (right) showing hyphae of Mr2575-GFP overlapping the edge of a Ma549-dsRed colony.


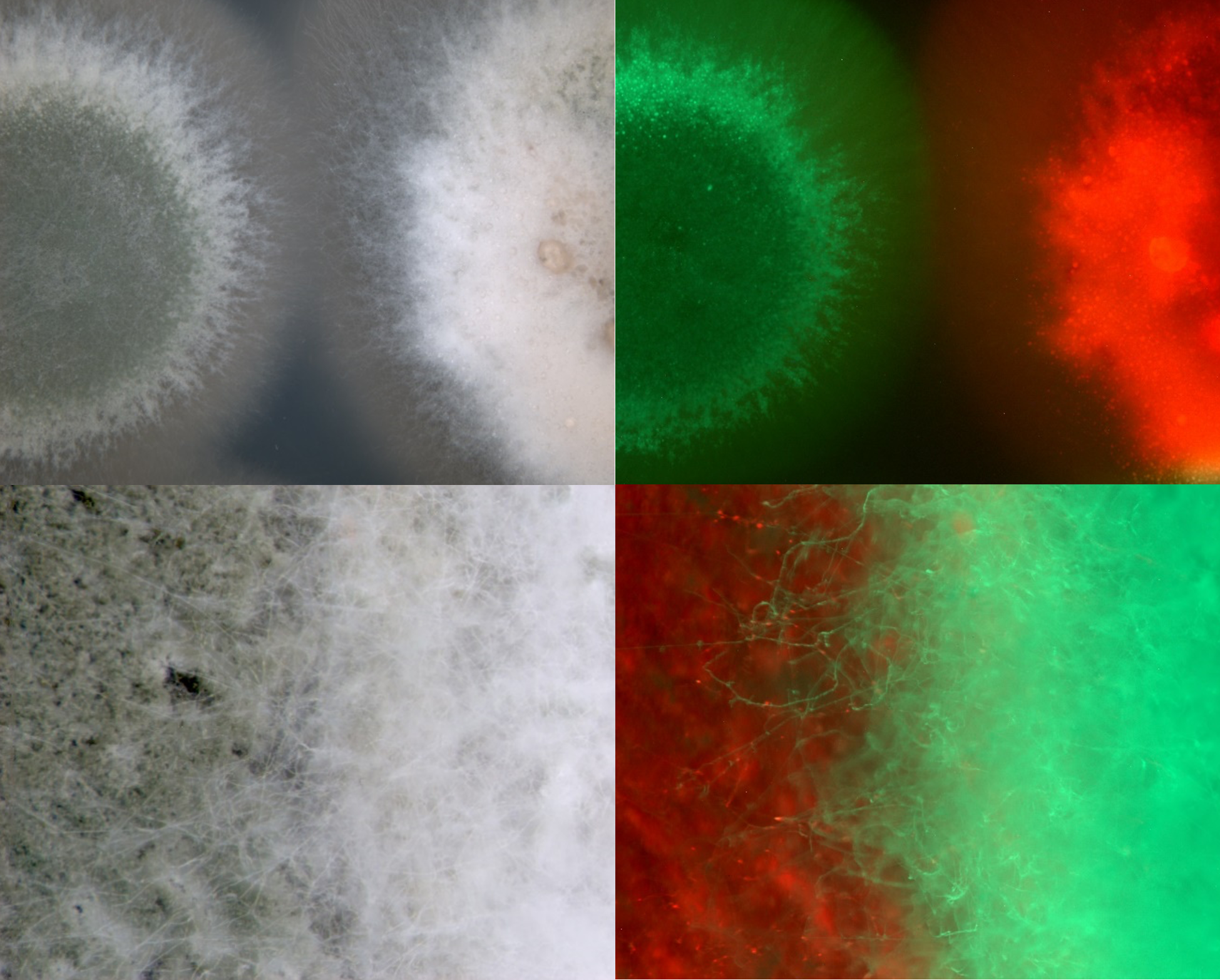

Supplement: S1 Fig — Top panels: bright field (left) and overlay of cherry and GFP (right) showing 5-day old colonies of Ma549-GFP and Mr2575-Cherry touching each other showing heavy sporulation by Ma549. Bottom panels: bright field (left) and overlay of GFP and dsRed (right) showing hyphae of Mr2575-GFP overlapping the edge of a Ma549-dsRed colony. (DOCX) [file ppat.1012639.s002.docx]
