# Supplementary material for: Metarhizium fight club: Within-host competitive exclusion and resource partitioning
Source: PLoS Pathog. 2024 Nov 7;20(11):e1012639. doi: 10.1371/journal.ppat.1012639 (PMC11542789; doi:10.1371/journal.ppat.1012639)

row 1 - only 549 row 2 - bright field of both row 3 - GFP from mixed
row 4 – mixed layover row 5 - RFP from mixed row 6 - only 2575

Female day 0

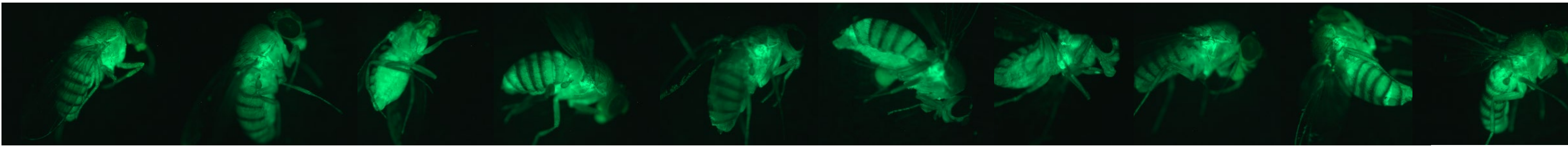

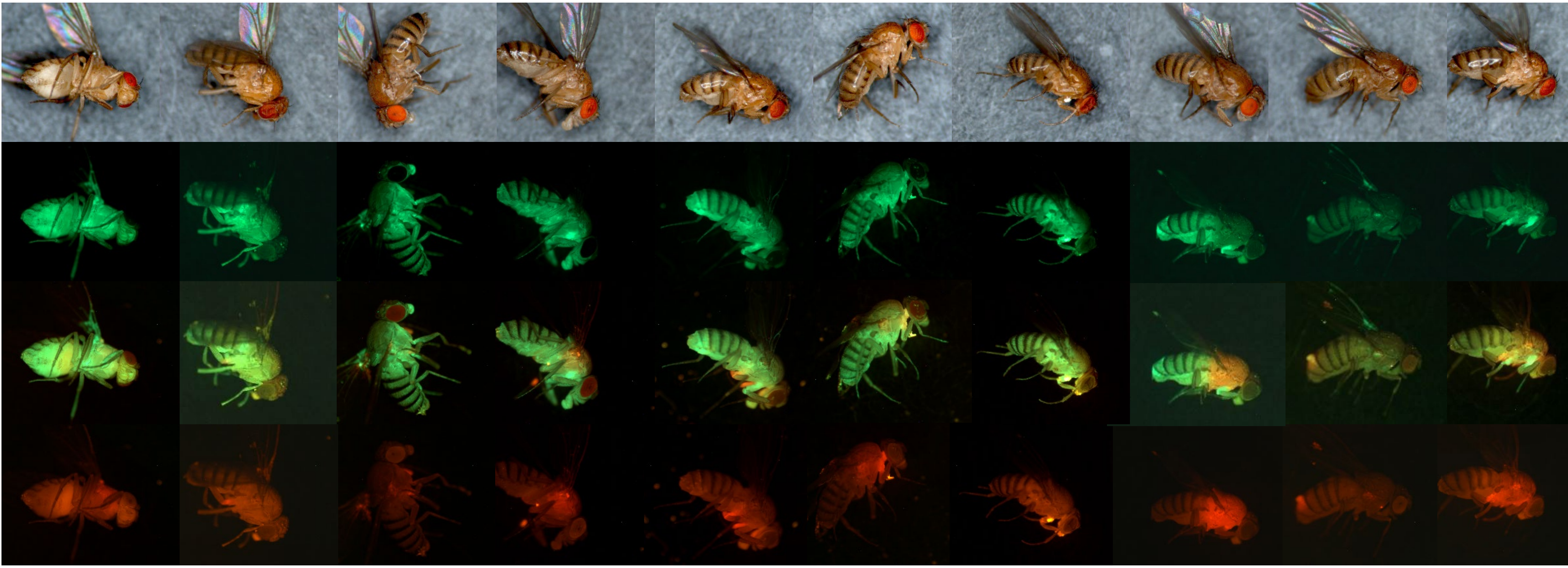

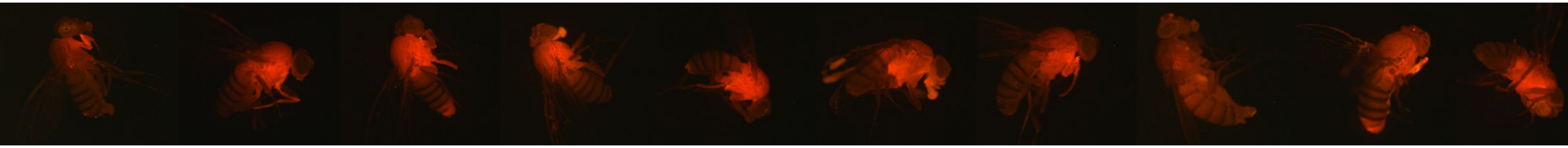

row 1 - only 549 row 2 - bright field of both row 3 - GFP from mixed
row 4 – mixed layover row 5 - RFP from mixed row 6 - only 2575

Male day 0

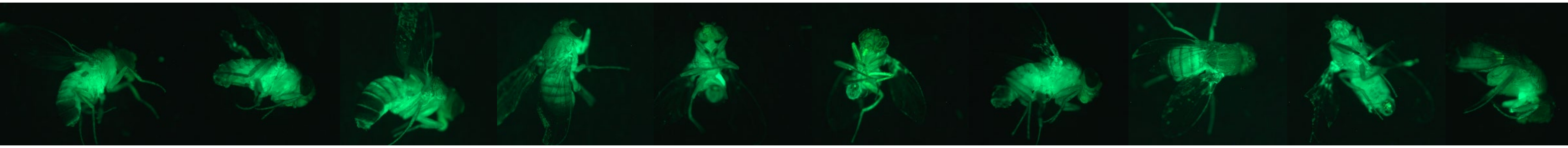

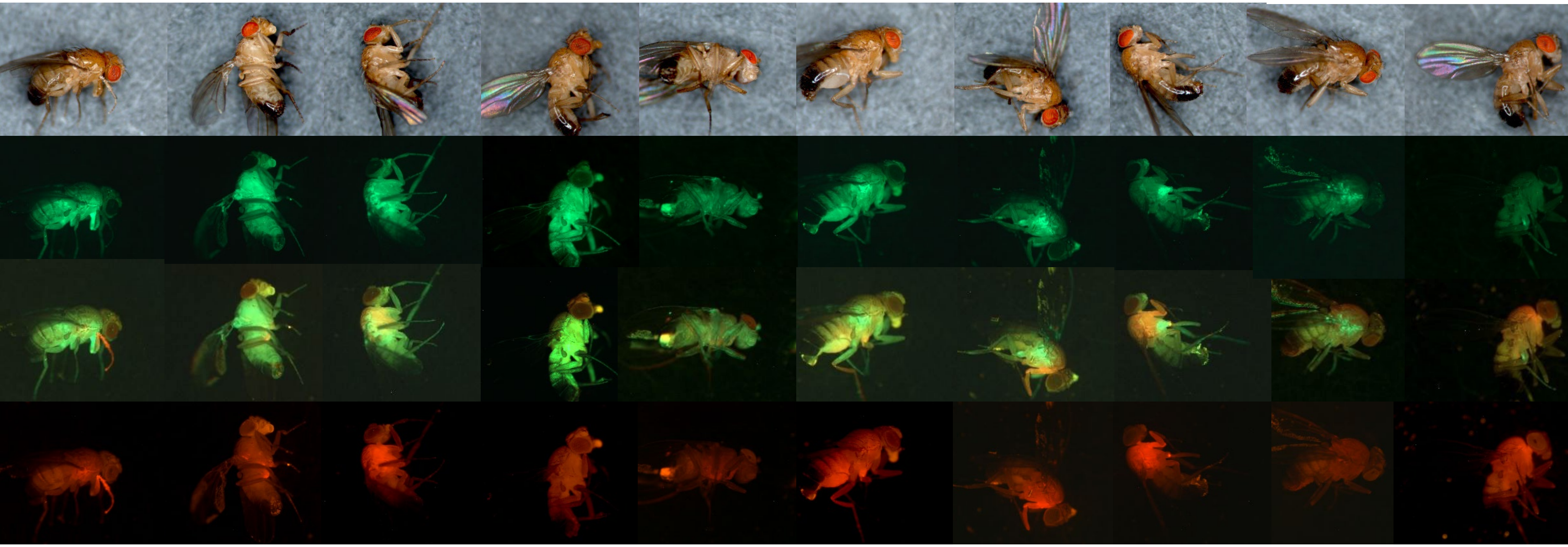

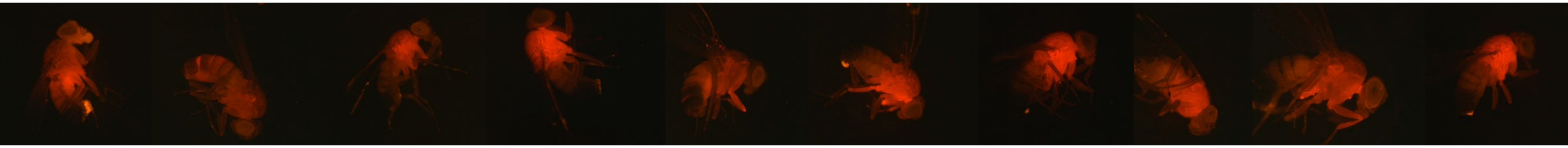

row 1 - only 549
row 2 - bright field of both
row 3 - GFP from mixed
row 4 – mixed layover
row 5 - RFP from mixed
row 6 - only 2575

Mixed Female day 1

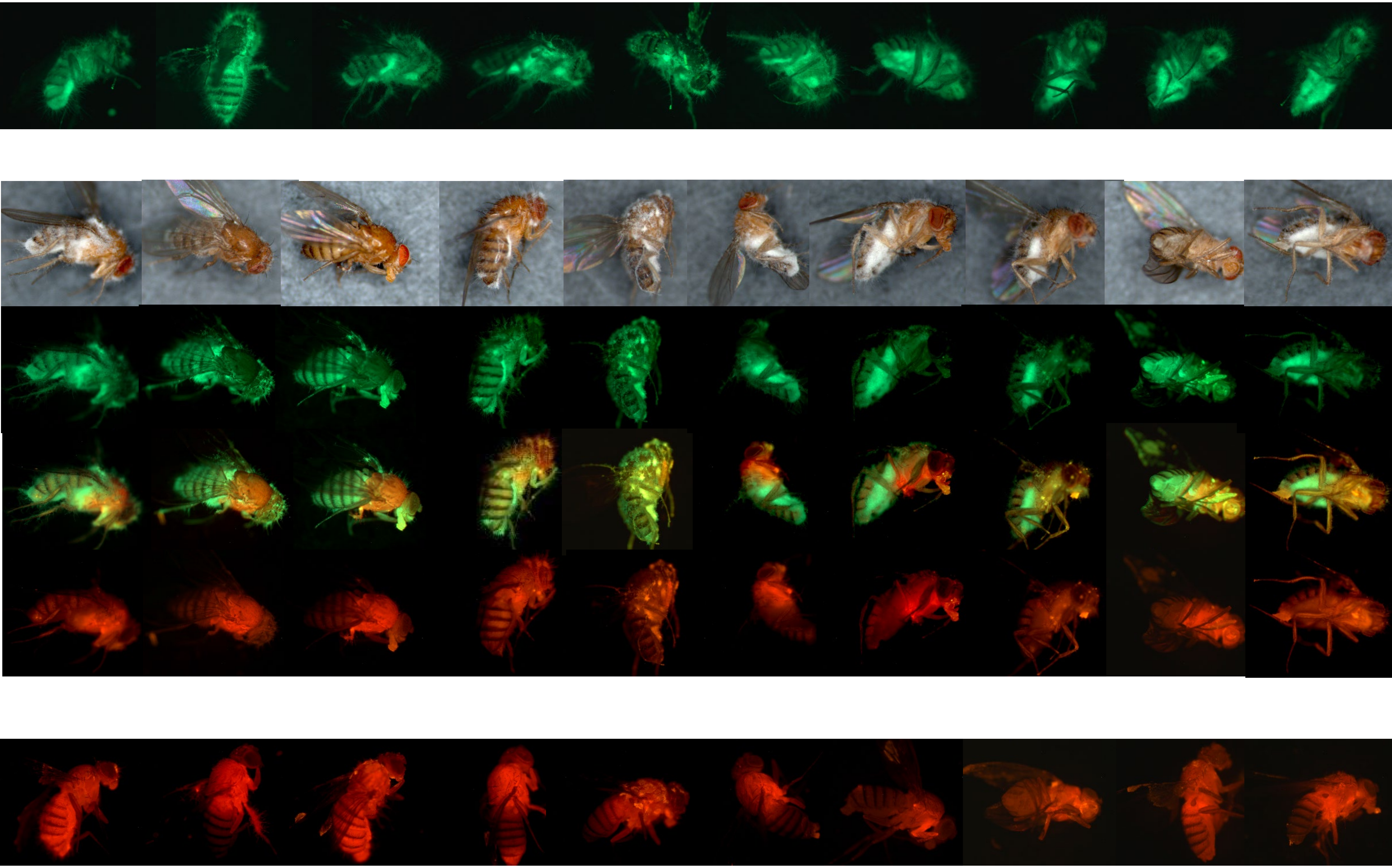

row 1 - only 549 row 2 - bright field of both row 3 - GFP from mixed
row 4 – mixed layover row 5 - RFP from mixed row 6 - only 2575

Male day 1

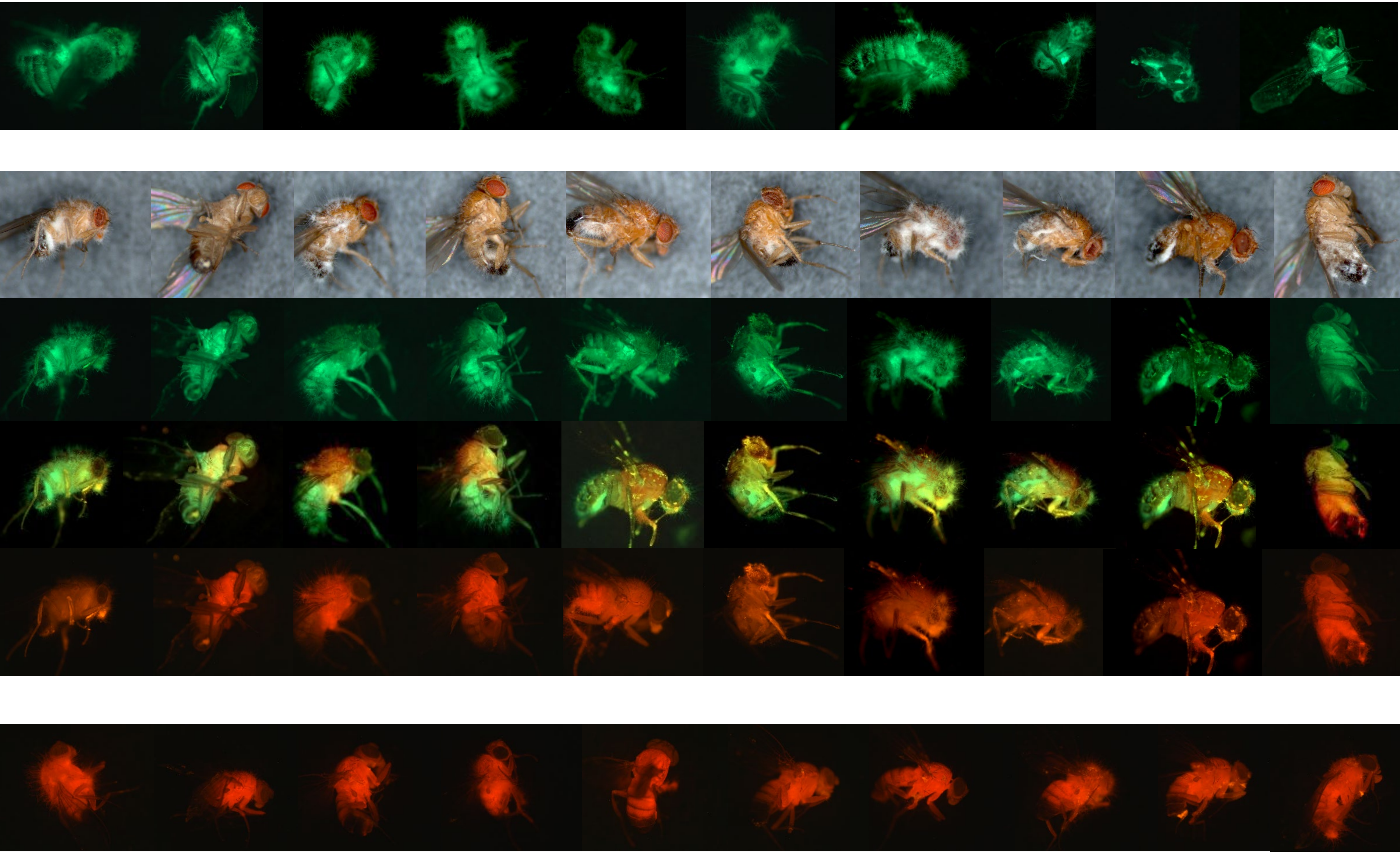

row 1 - only 549
row 2 - bright field of both
row 3 - GFP from mixed
row 4 – mixed layover
row 5 - RFP from mixed
row 6 - only 2575

Female day 2

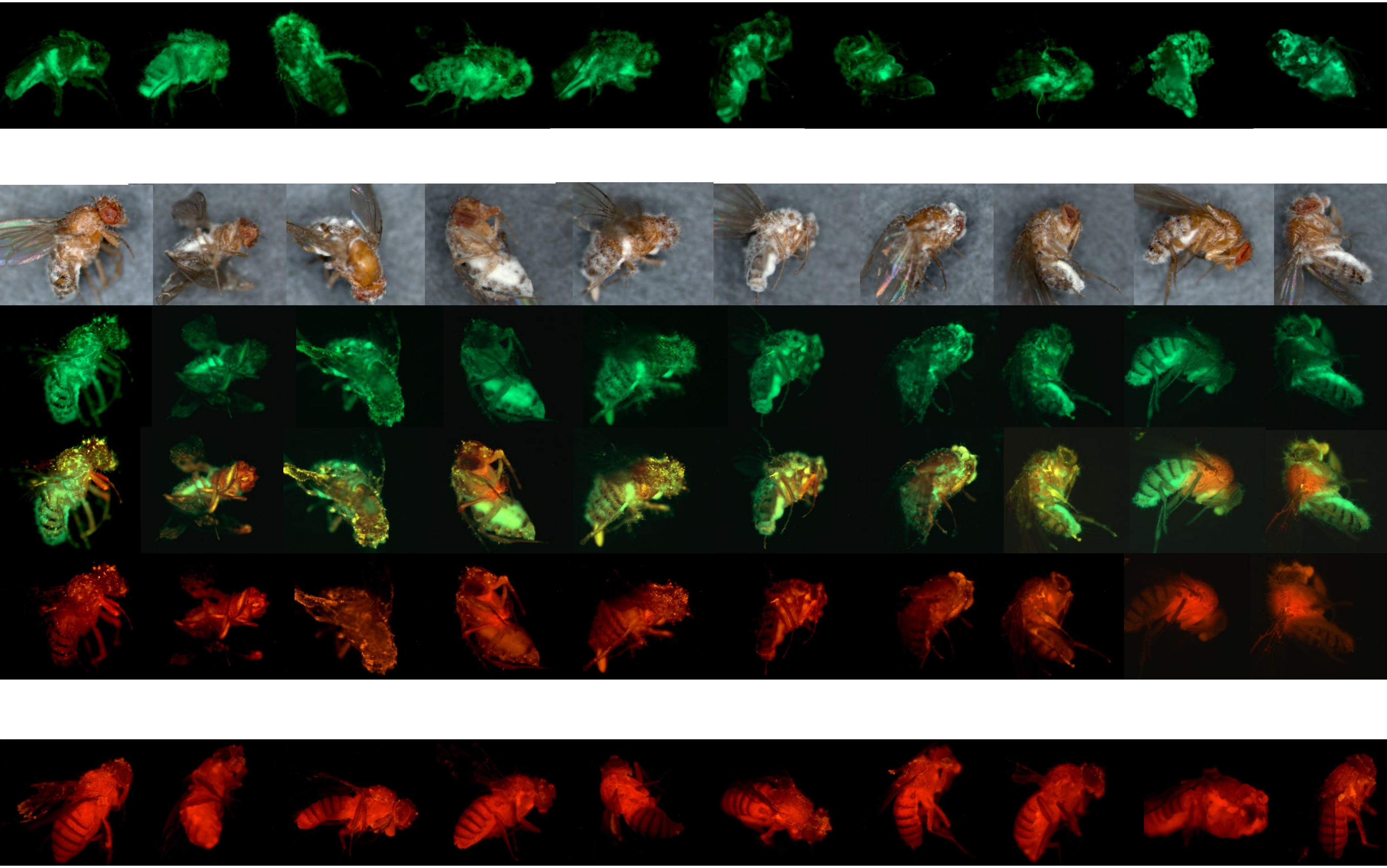

row 1 - only 549
row 2 - bright field of both
row 3 - GFP from mixed
row 4 – mixed layover
row 5 - RFP from mixed
row 6 - only 2575

Male Day 2

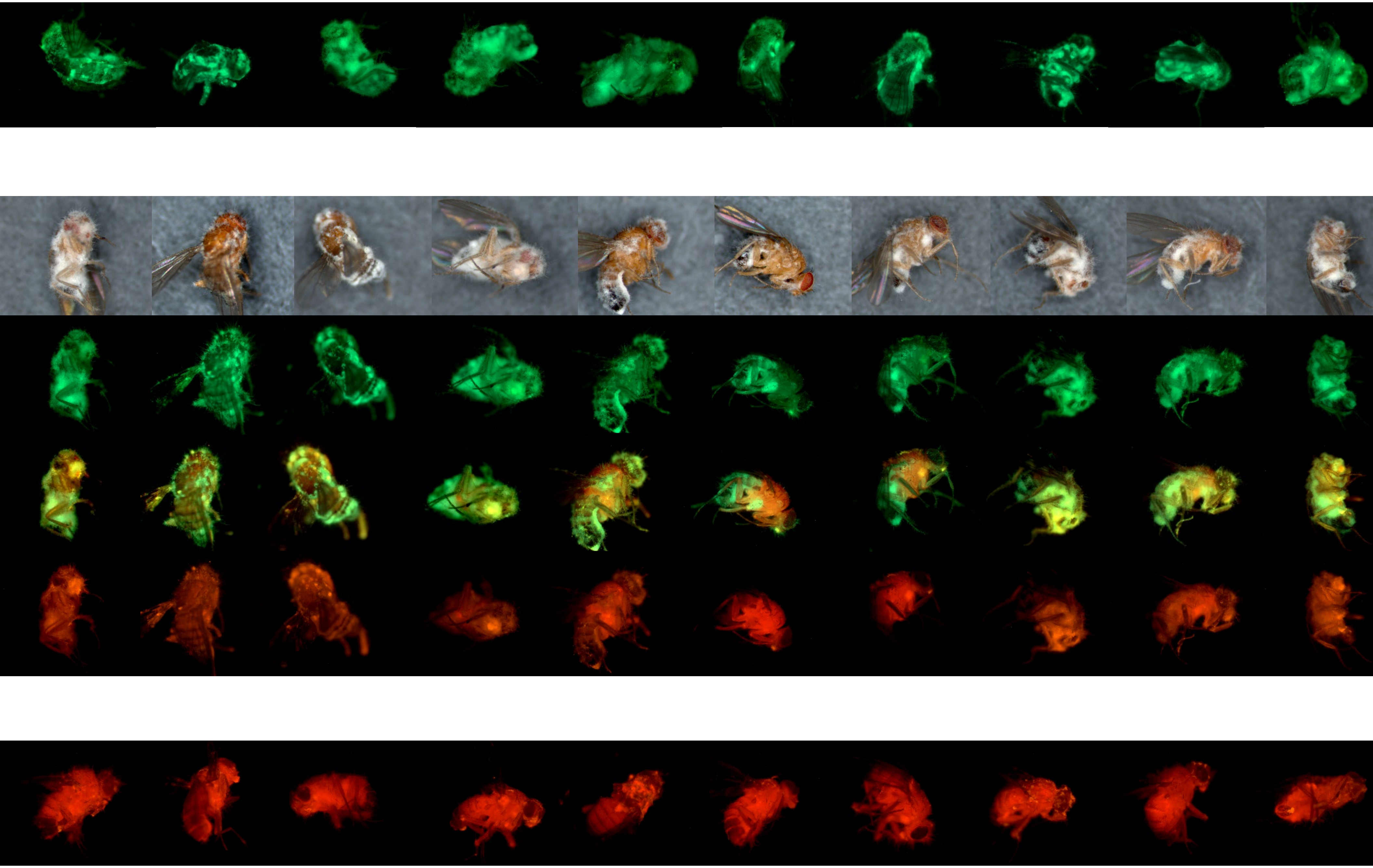

row 1 - only 549
row 2 - bright field of both
row 3 - GFP from mixed
row 4 – mixed layover
row 5 - RFP from mixed
row 6 - only 2575

Female day 3

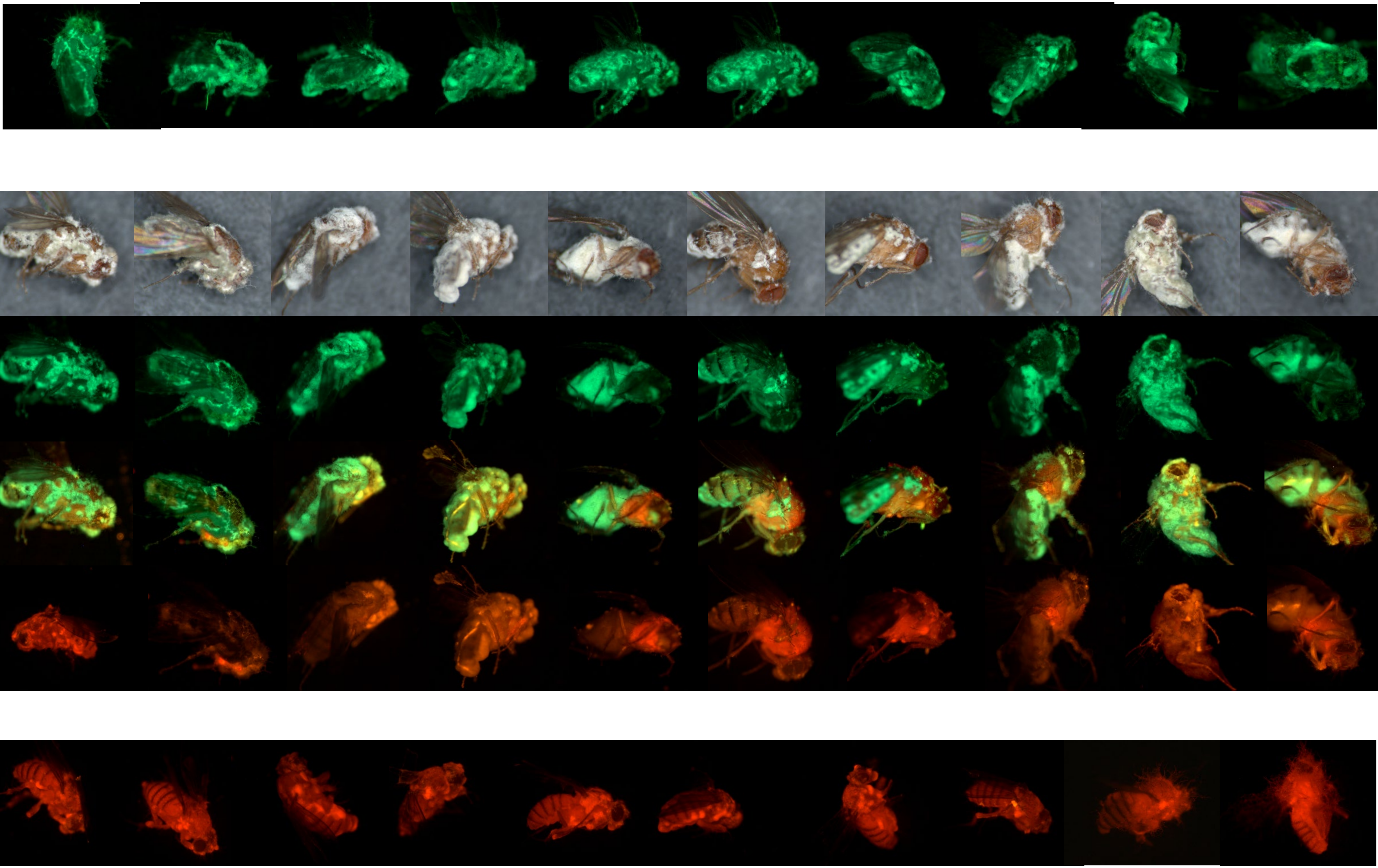

row 1 - only 549 row 2 - bright field of both row 3 - GFP from mixed
row 4 – mixed layover row 5 - RFP from mixed row 6 - only 2575

Male day 3

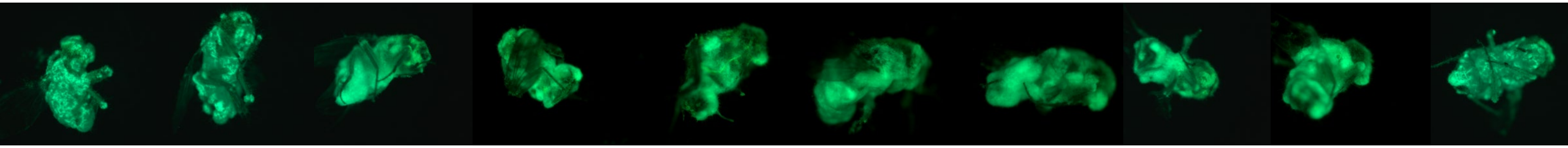

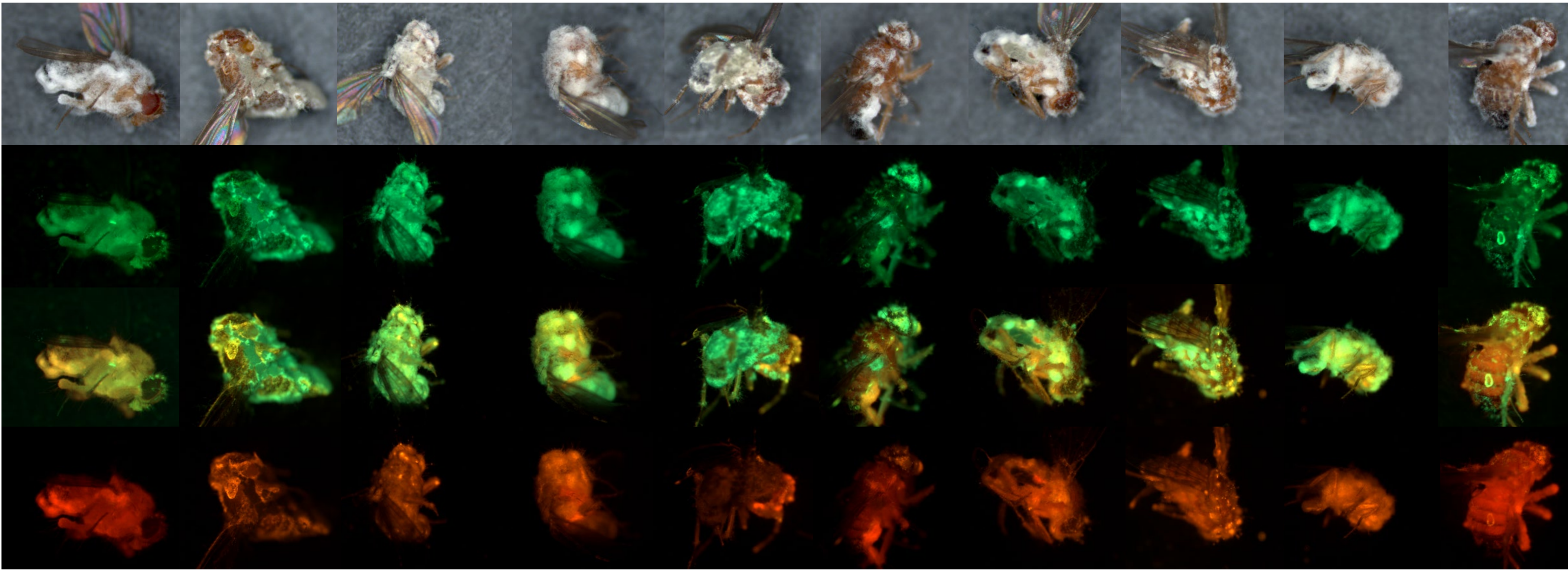

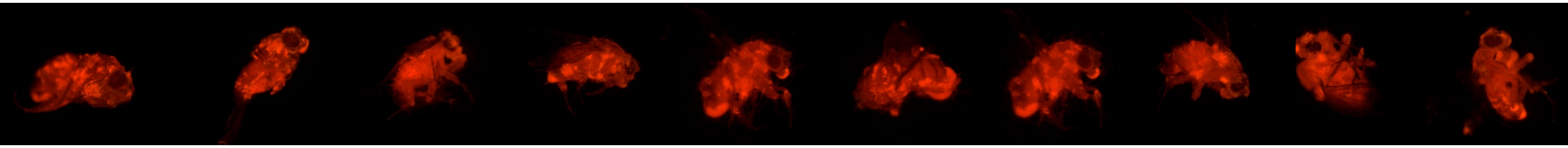

row 1 - only 549 row 2 - bright field of both row 3 - GFP from mixed
row 4 – mixed layover row 5 - RFP from mixed row 6 - only 2575

Female day 4

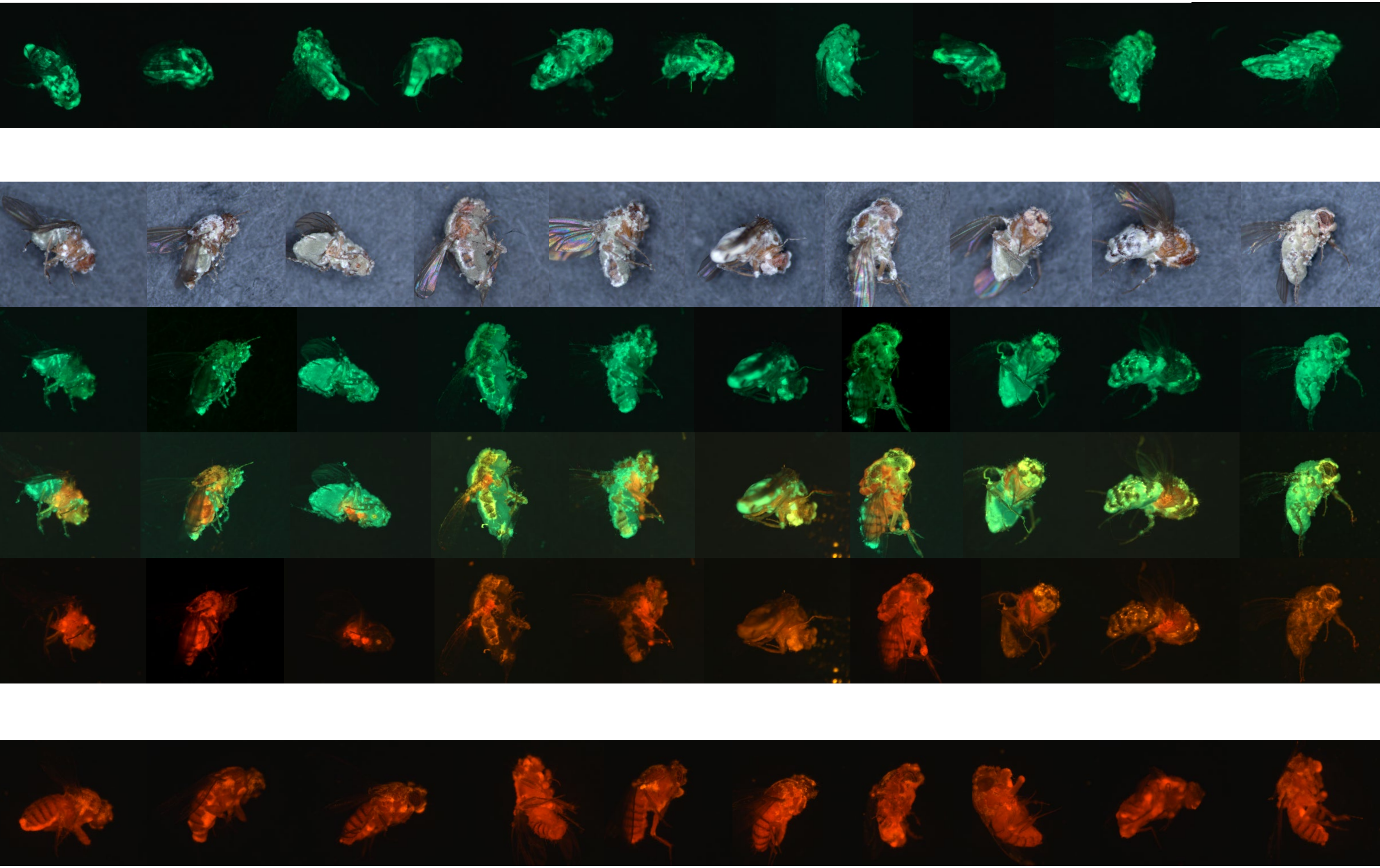

row 1 - only 549 row 2 - bright field of both row 3 - GFP from mixed

row 4 – mixed layover row 5 - RFP from mixed row 6 - only 2575

Male Day 4

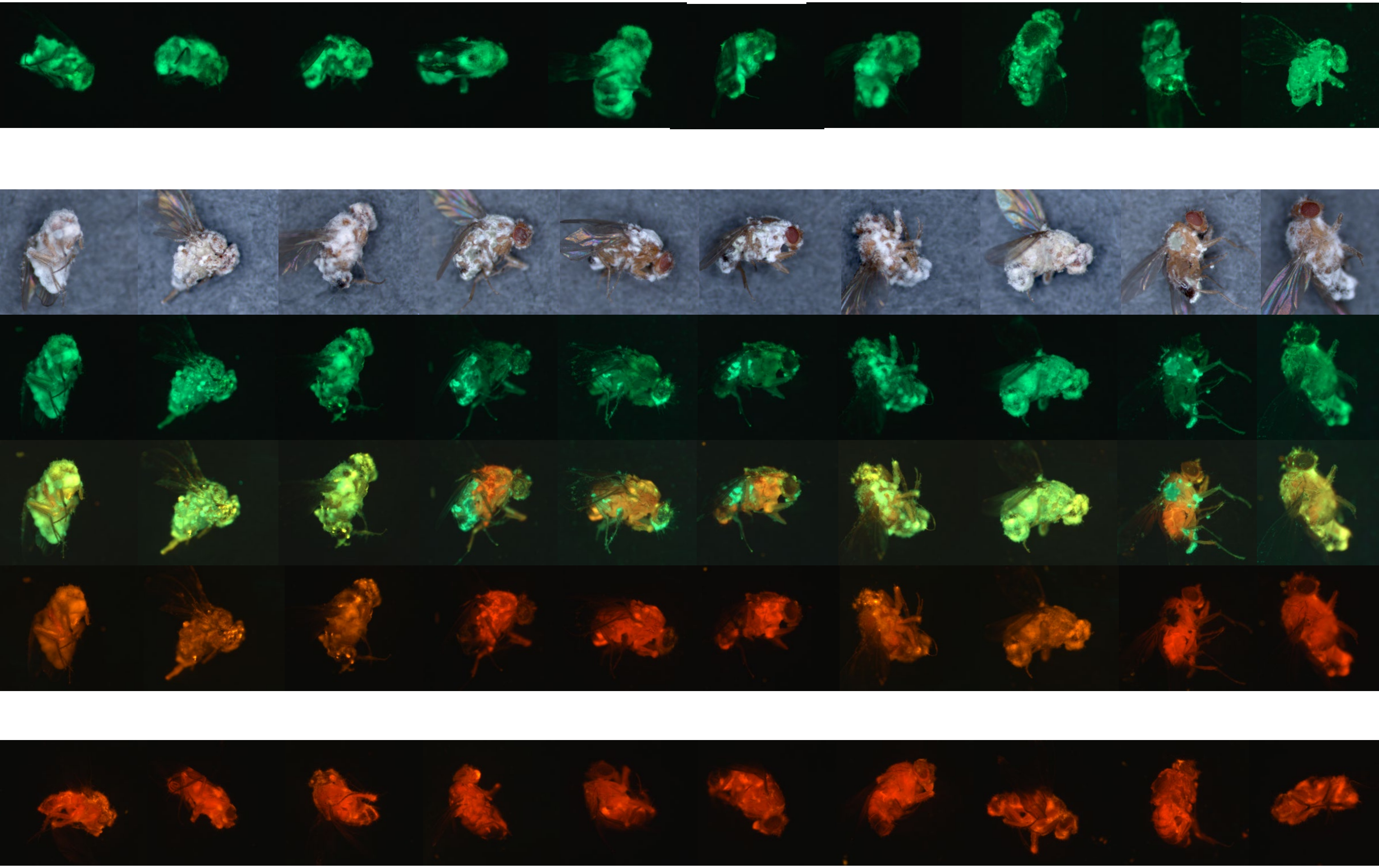

row 1 - only 549 row 2 - bright field of both row 3 - GFP from mixed

row 4 – mixed layover row 5 - RFP from mixed row 6 - only 2575

<u>Female day 5 full sporulation</u>

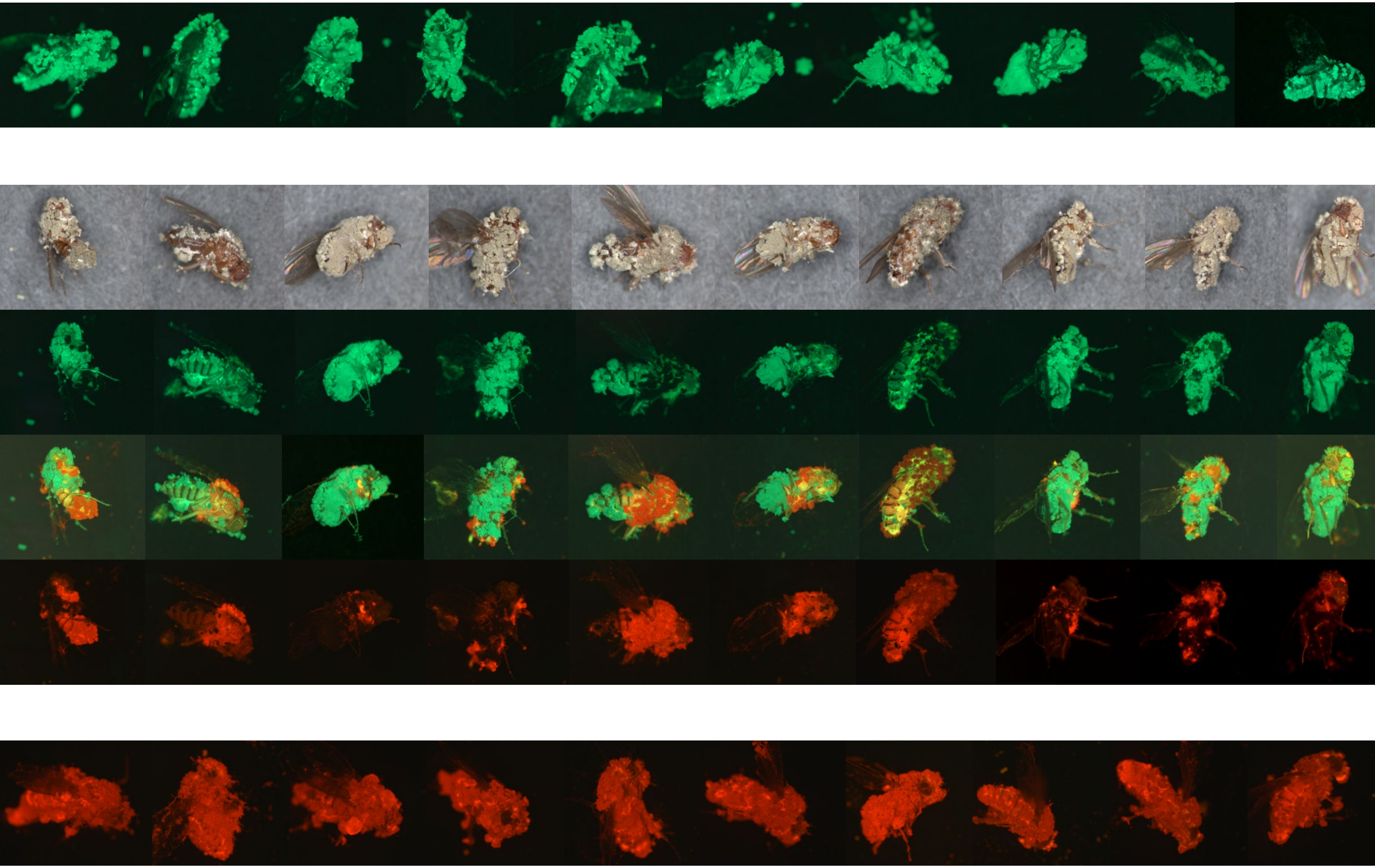

row 1 - only 549
row 2 - bright field of both
row 3 - GFP from mixed
row 4 – mixed layover
row 5 - RFP from mixed
row 6 - only 2575

male day 5 full sporulation

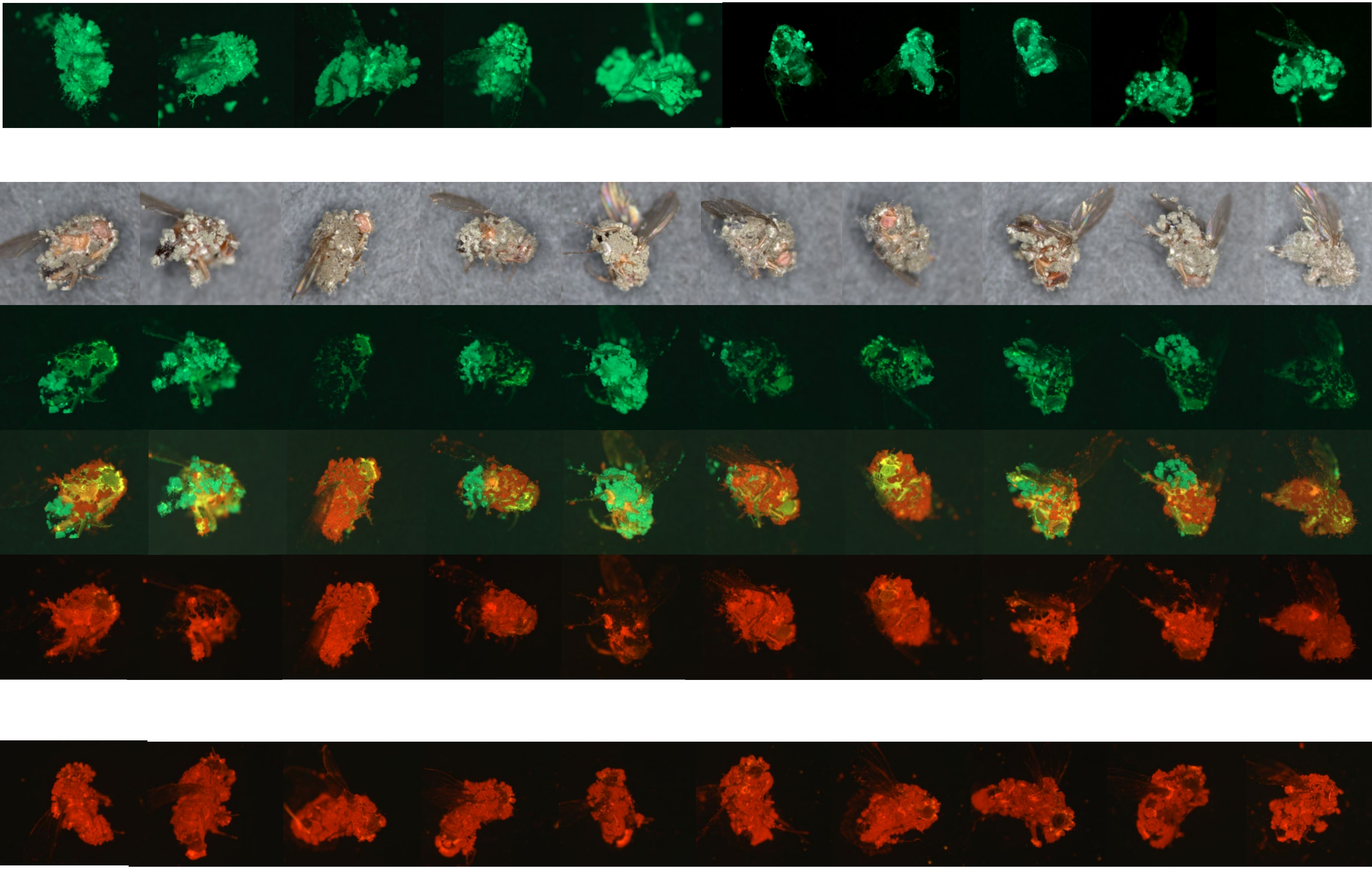

Supplement: S2 Fig — The fungi were applied topically either singly or together and 10 randomly selected male or female hosts from each treatment were visualized at one day intervals with both bright field and epifluorescence, with filters set to detect GFP fluorescence or Cherry. Images are segregated by sex and day. For each sex and day row 1 are flies infected by Ma549 and row 6 are flies infected by Mr2575; rows 2 to 5 mixed Ma549 + Mr2575 infections with row 2 bright field, row 3 GFP, row 4 overlay of Cherry and GFP, row 5 Cherry. (PDF) [file ppat.1012639.s003.pdf]
