# Supplementary figures and images for: Metarhizium fight club: Within-host competitive exclusion and resource partitioning
Source: PLoS Pathog. 2024 Nov 7;20(11):e1012639. doi: 10.1371/journal.ppat.1012639 (PMC11542789; doi:10.1371/journal.ppat.1012639)

Sarcophaga mix


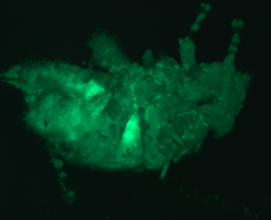

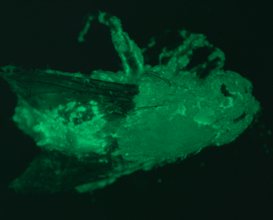

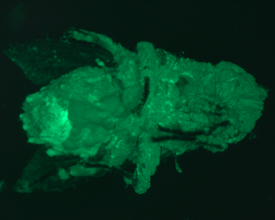

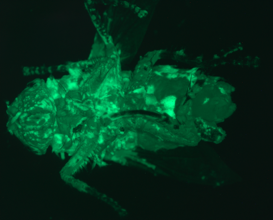

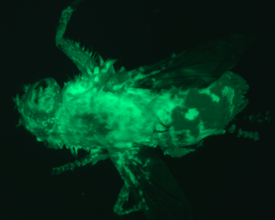

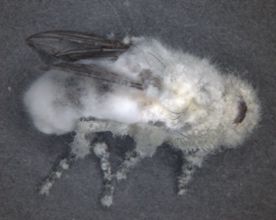

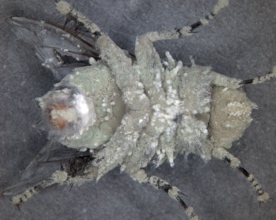

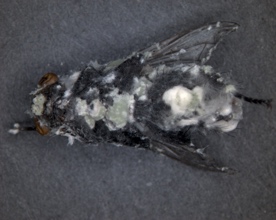

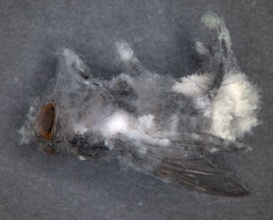

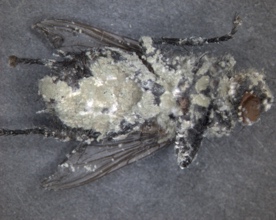

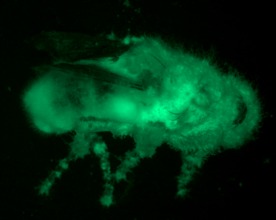

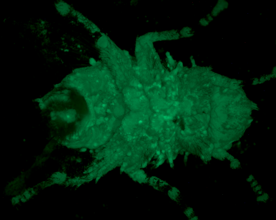

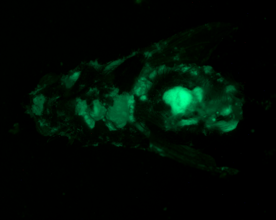

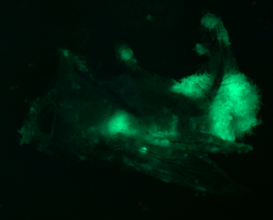

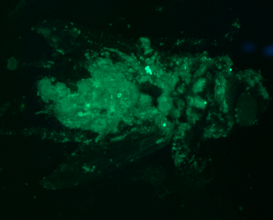

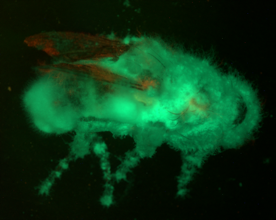

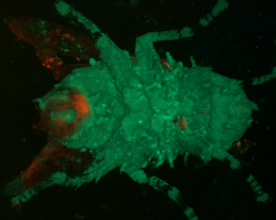

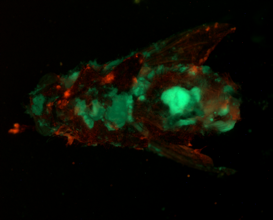

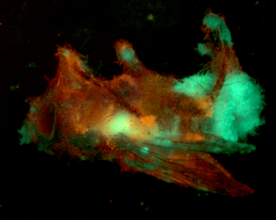

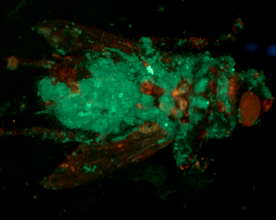

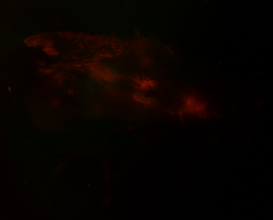

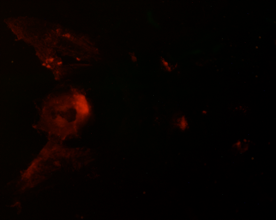

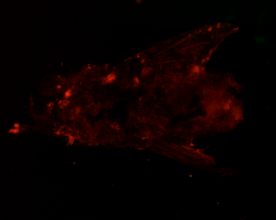

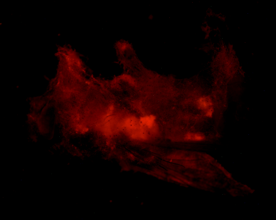

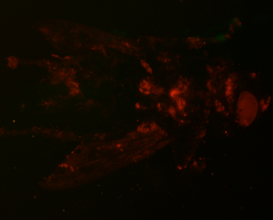

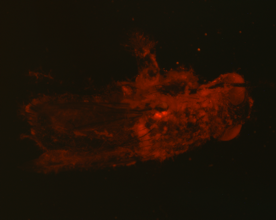

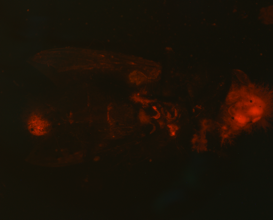

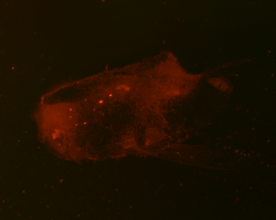

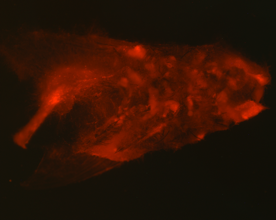

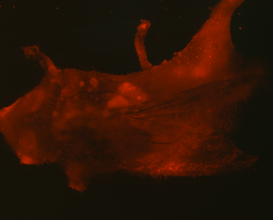


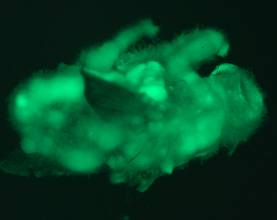


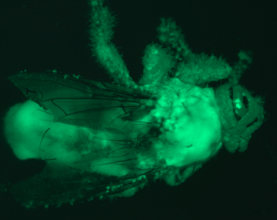

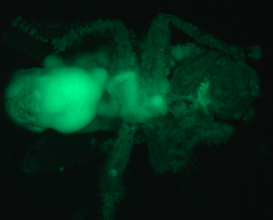

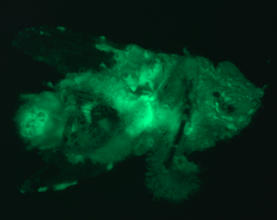

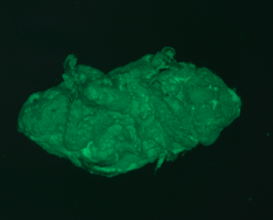

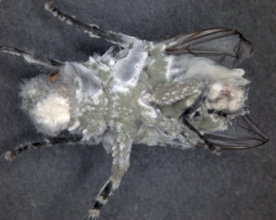

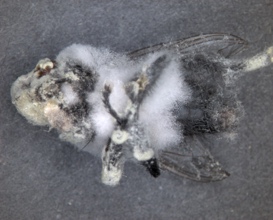

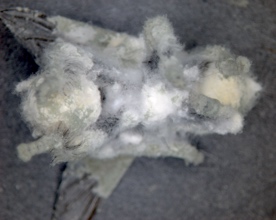

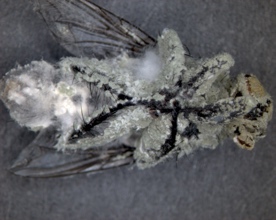

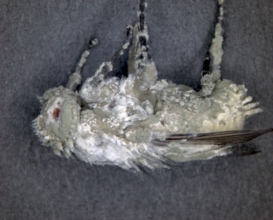

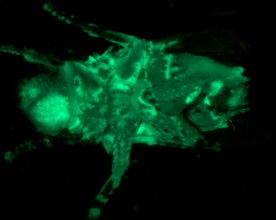

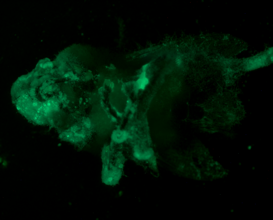

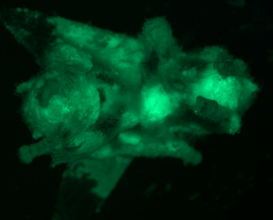

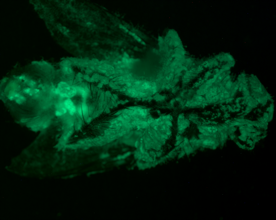

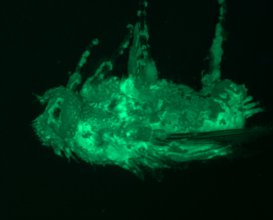

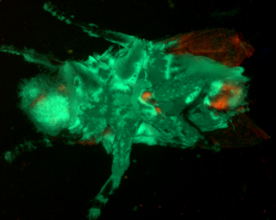

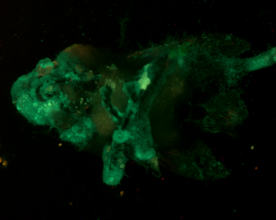

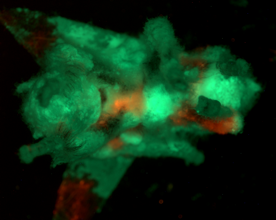

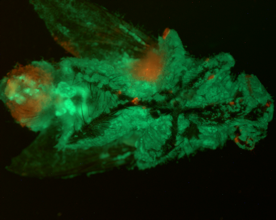

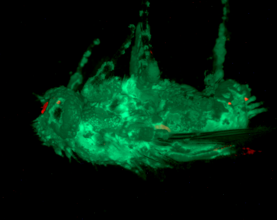

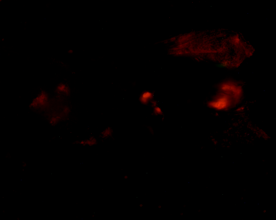

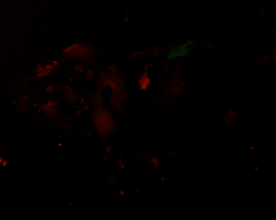

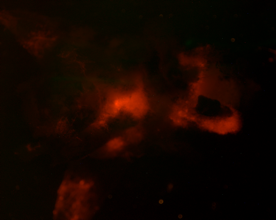

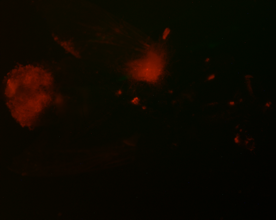

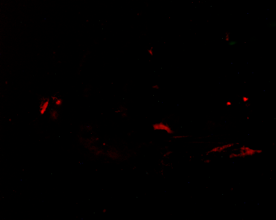


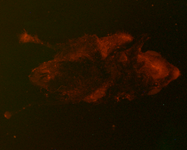


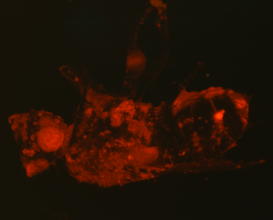

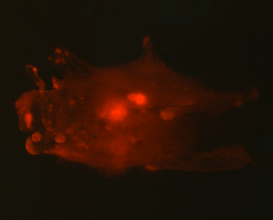

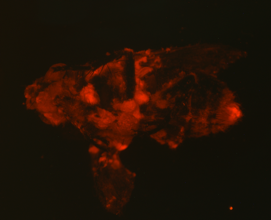

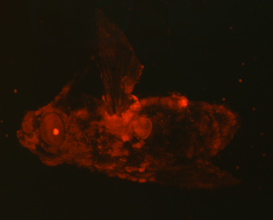

Supplement: S3 Fig — The fungi were applied topically either singly or together and 10 hosts (not segregated by sex) from each treatment were visualized with both bright field and epifluorescence, with filters set to detect GFP fluorescence or Cherry. (DOCX) [file ppat.1012639.s004.docx]
