# Supplementary material for: Metarhizium fight club: Within-host competitive exclusion and resource partitioning
Source: PLoS Pathog. 2024 Nov 7;20(11):e1012639. doi: 10.1371/journal.ppat.1012639 (PMC11542789; doi:10.1371/journal.ppat.1012639)

Supplementary figures and their captions

Fig S4A i) GFP, Cherry image and overlay for panel A in Fig 1.

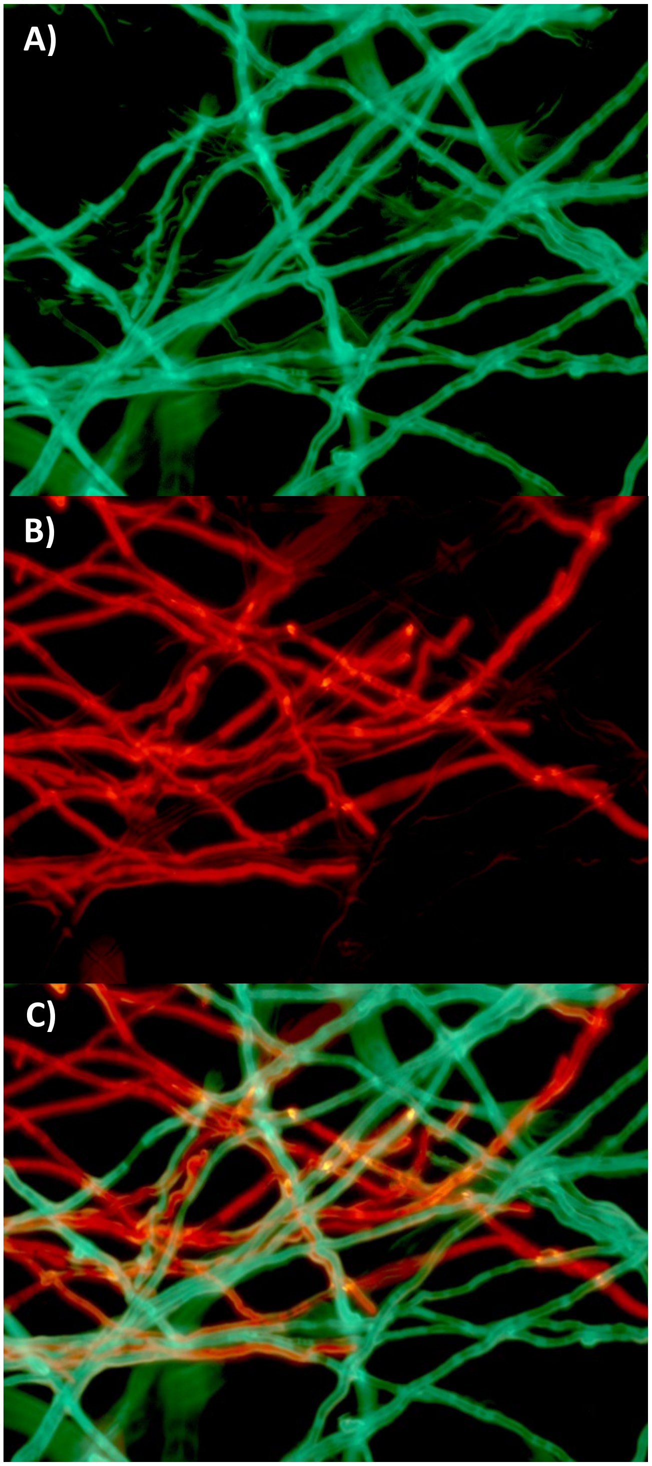

Fig S4A ii) GFP, Cherry image and overlay for panel D in Fig 1.

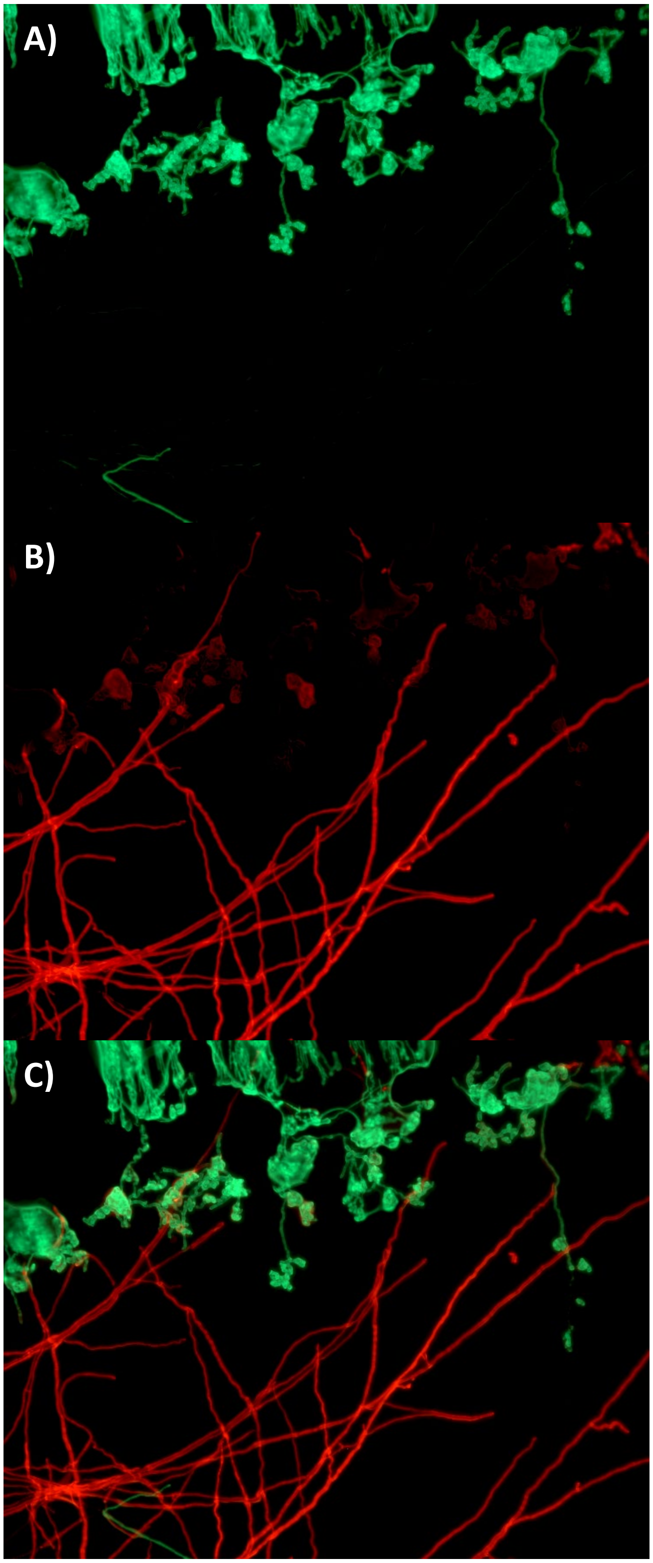

Fig S4B i) GFP, Cherry image and overlay for Fig 6 panel B

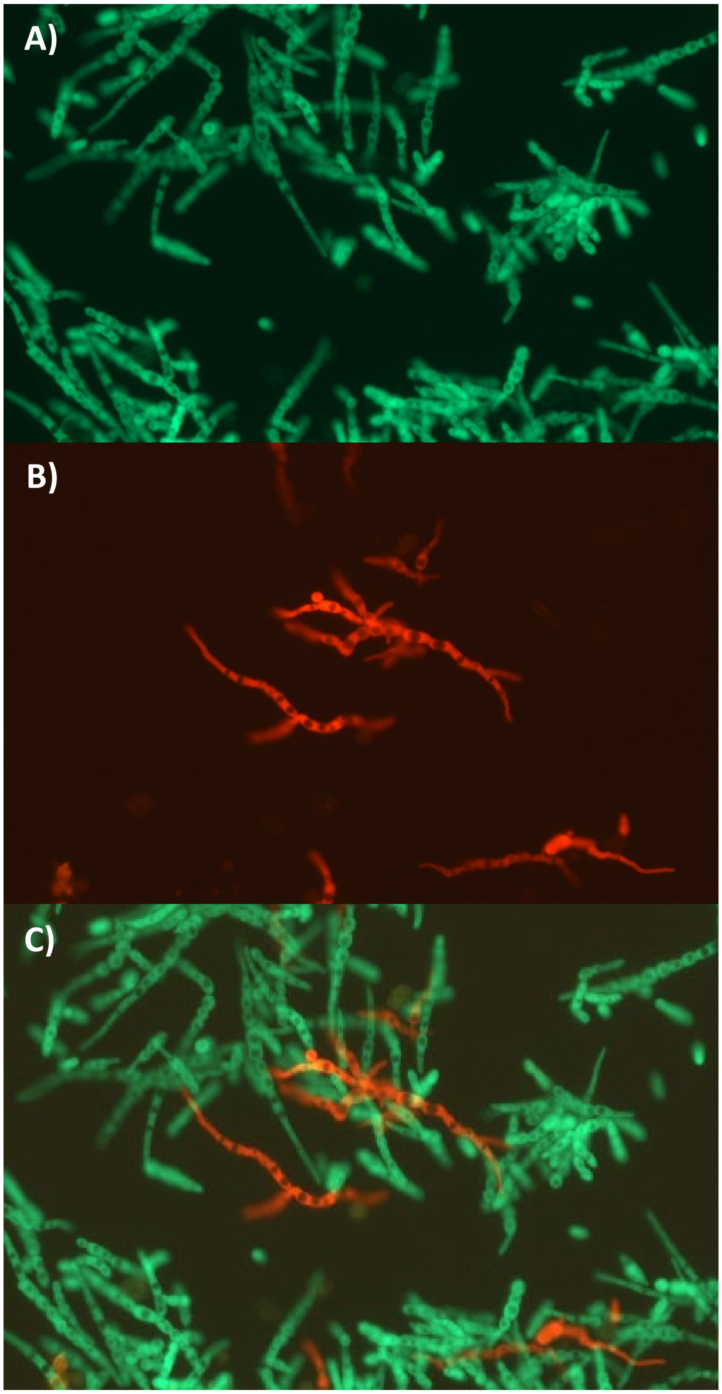

Fig S4C i) GFP, Cherry image and overlay for Fig 7 panel B

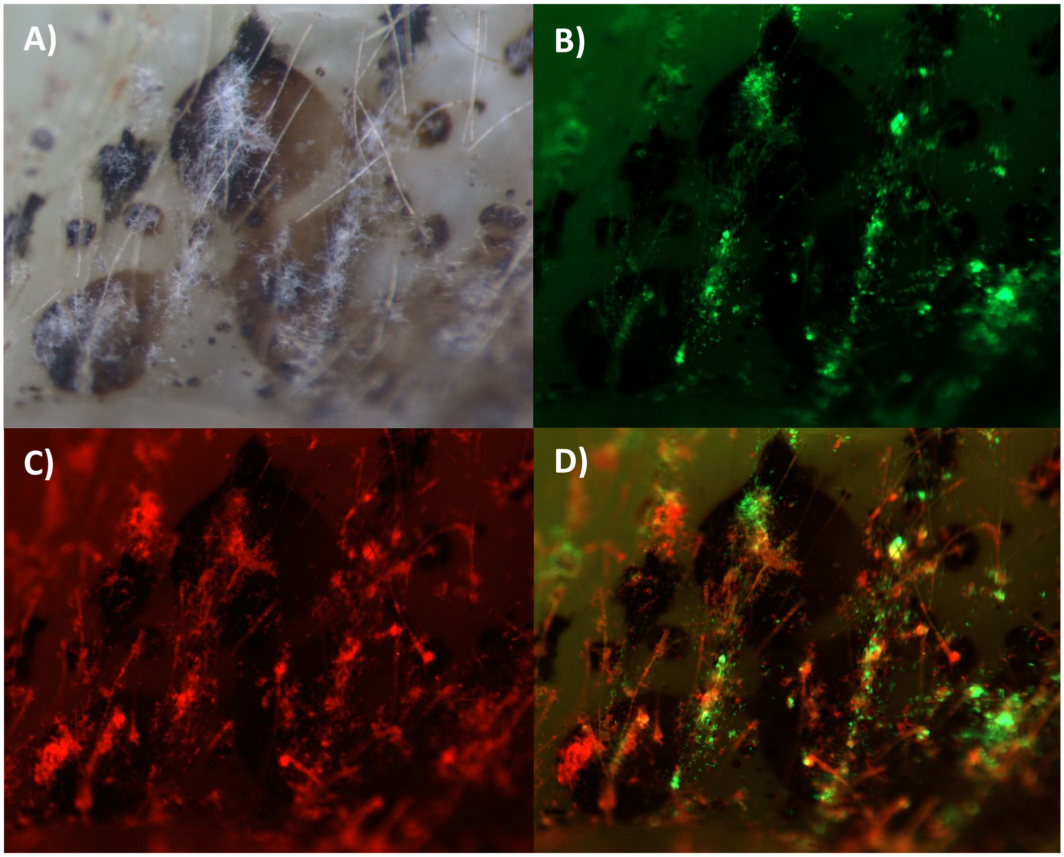

Fig S4C ii) Bright field, GFP, Cherry and overlay for Fig 7 panel D. The overlay of the bright field with the cherry and GFP overlay is shown in the lowest panel

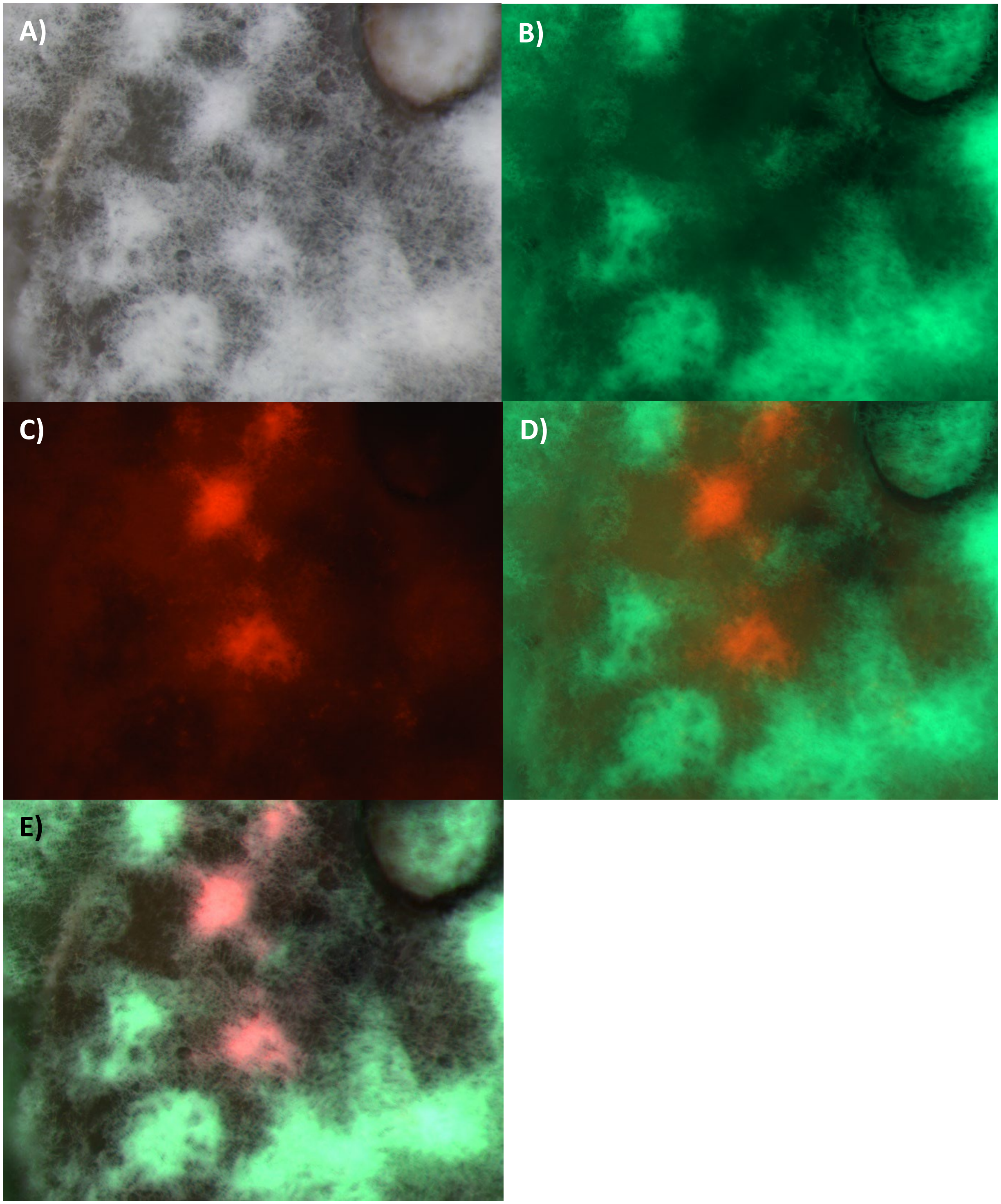

Fig S4C iii) Bright field, GFP, Cherry and overlay for Fig 7 panel F.

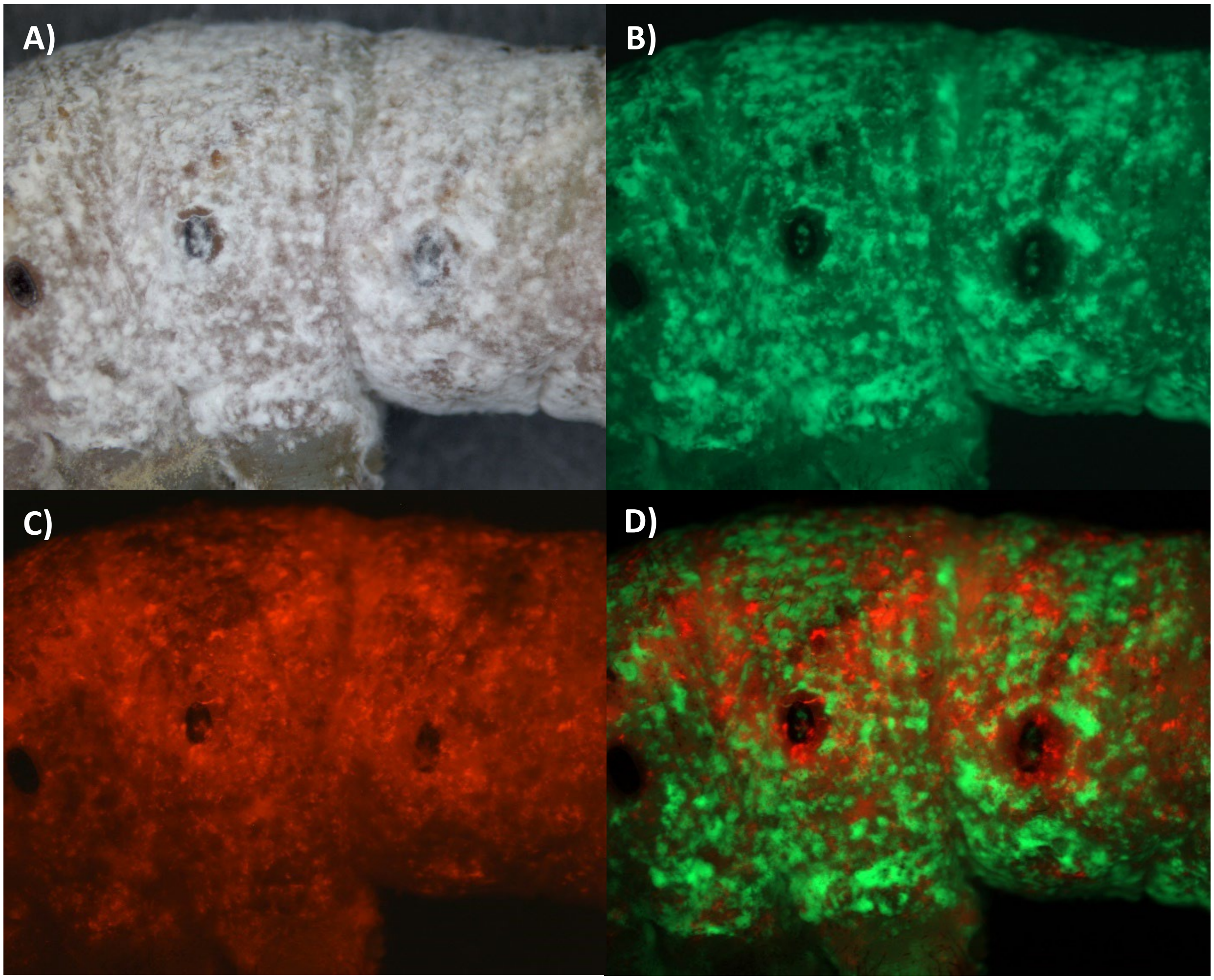

SFig 4D i) Bright field, GFP, Cherry and overlay for Fig 8 panel B.

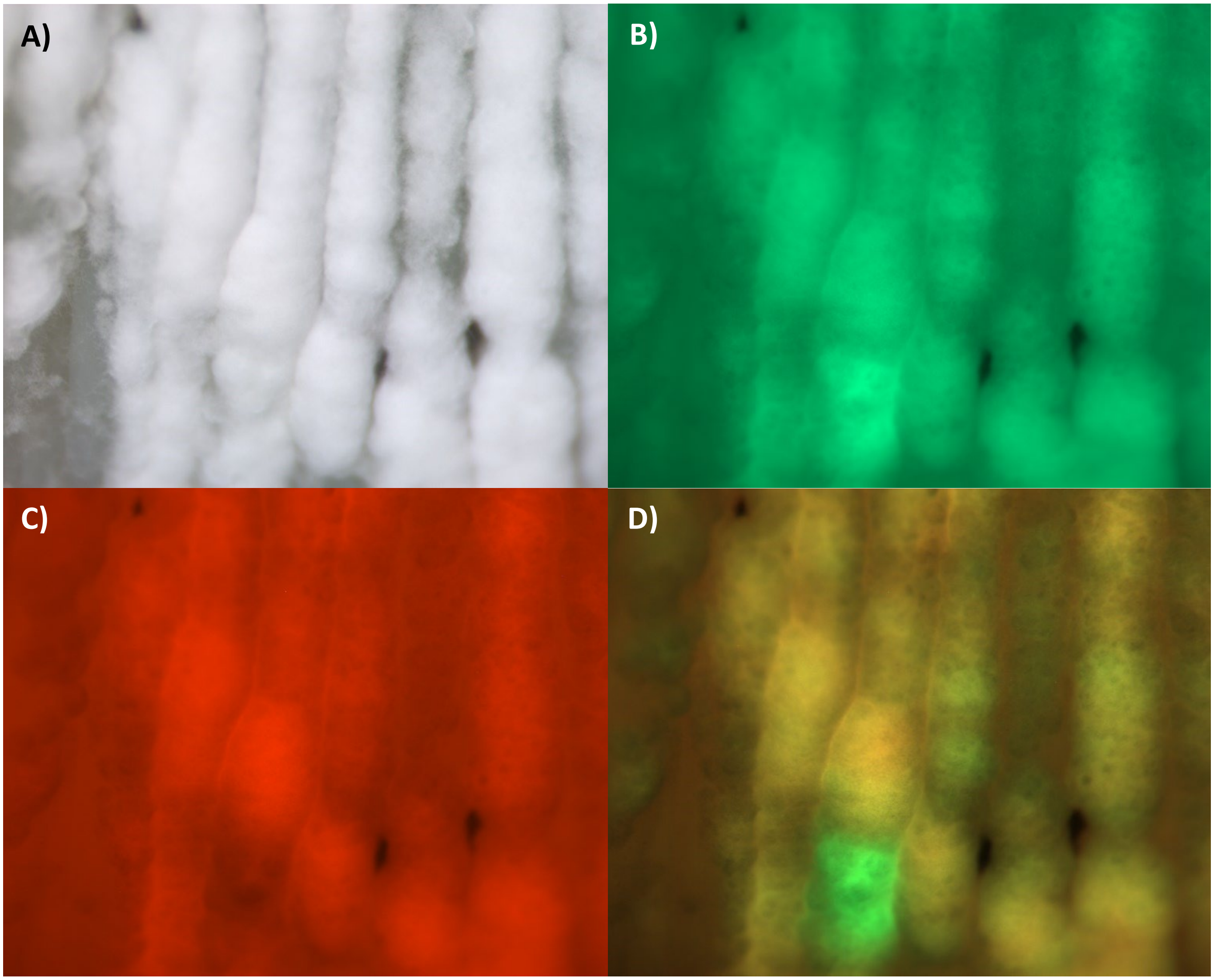

SFig 4D ii) Bright field, GFP, Cherry and overlay for Fig 8 panel D.

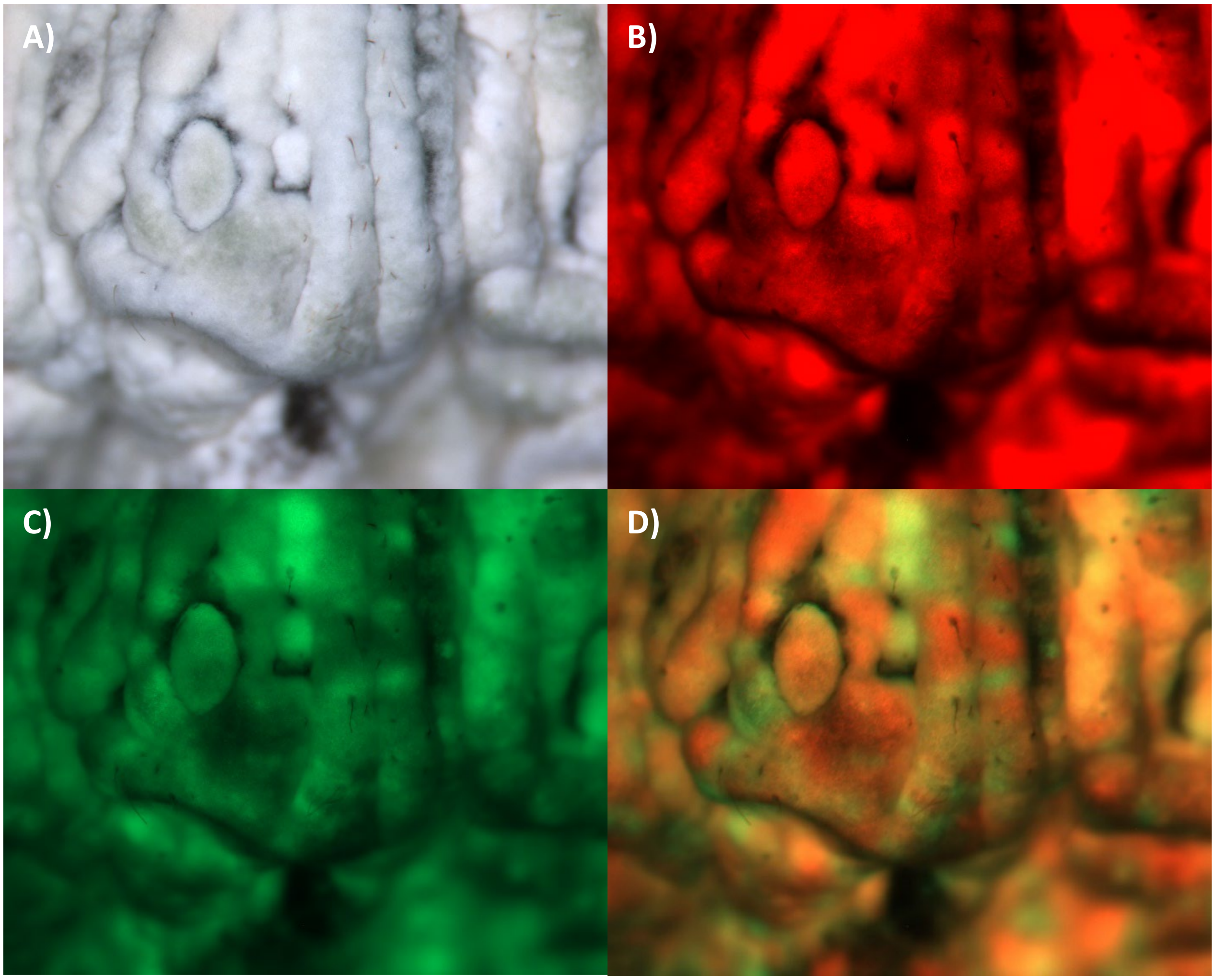

SFig 4D iii) Bright field, GFP, Cherry and overlay for Fig 8 panel F.

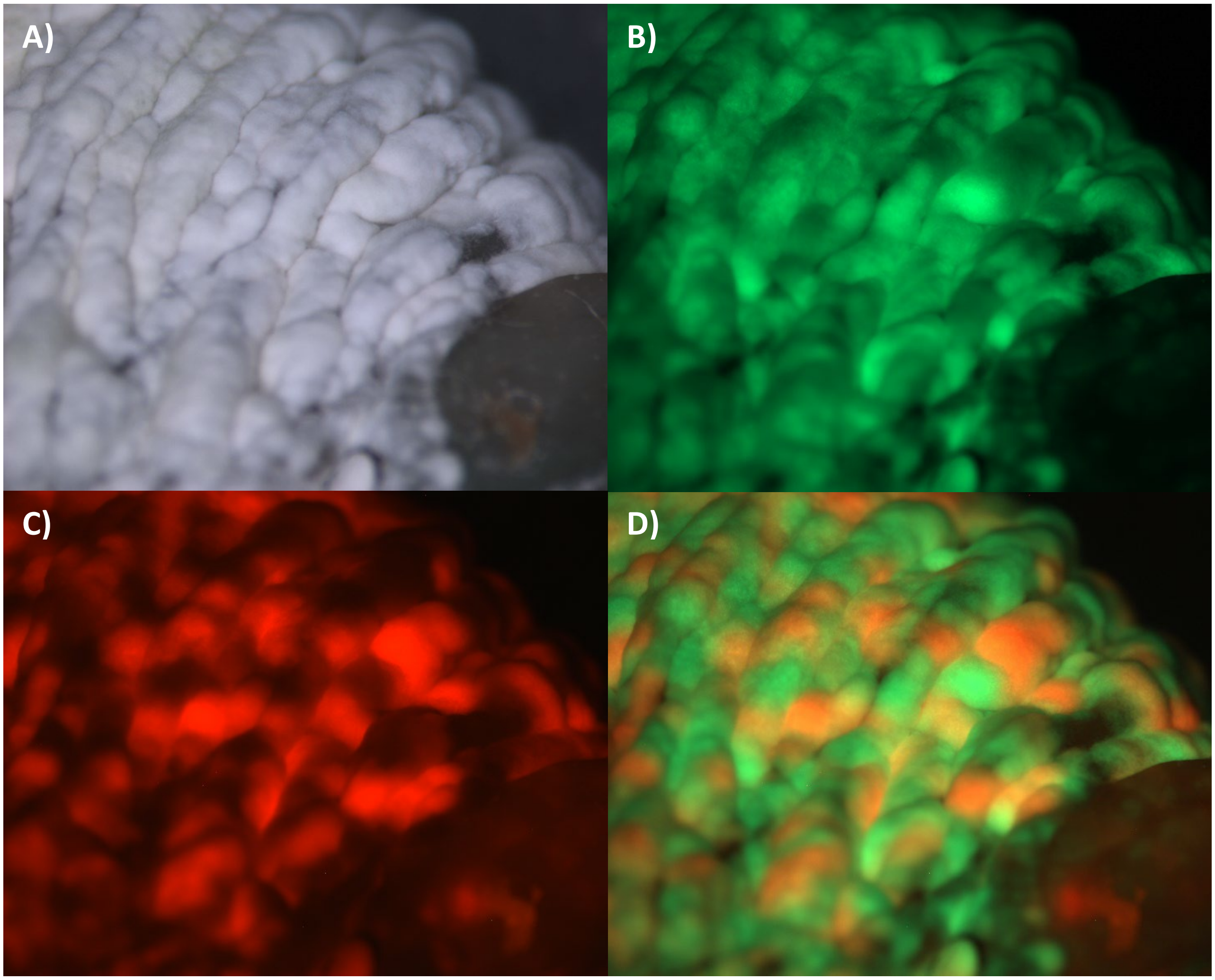

SFig 4D iv) Bright field, GFP, Cherry and overlay for Fig 8 panel H.

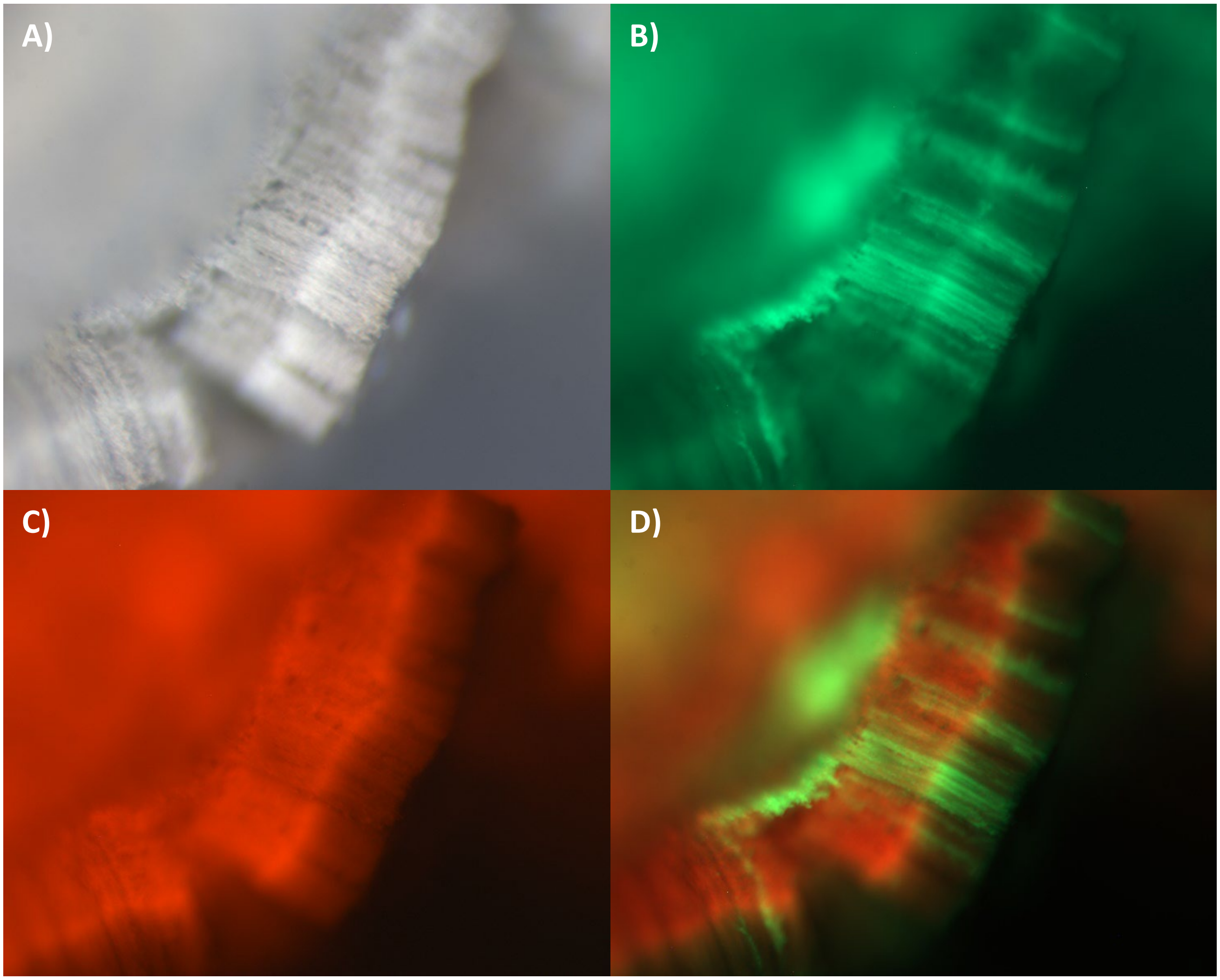

SFig 4E i) Bright field, GFP, cherry/dsRed and overlay images of caterpillar (infected with equal spore doses of Mr2575-cherry, Mr2575-GFP and Ma549-dsRed) for Fig 10 B.

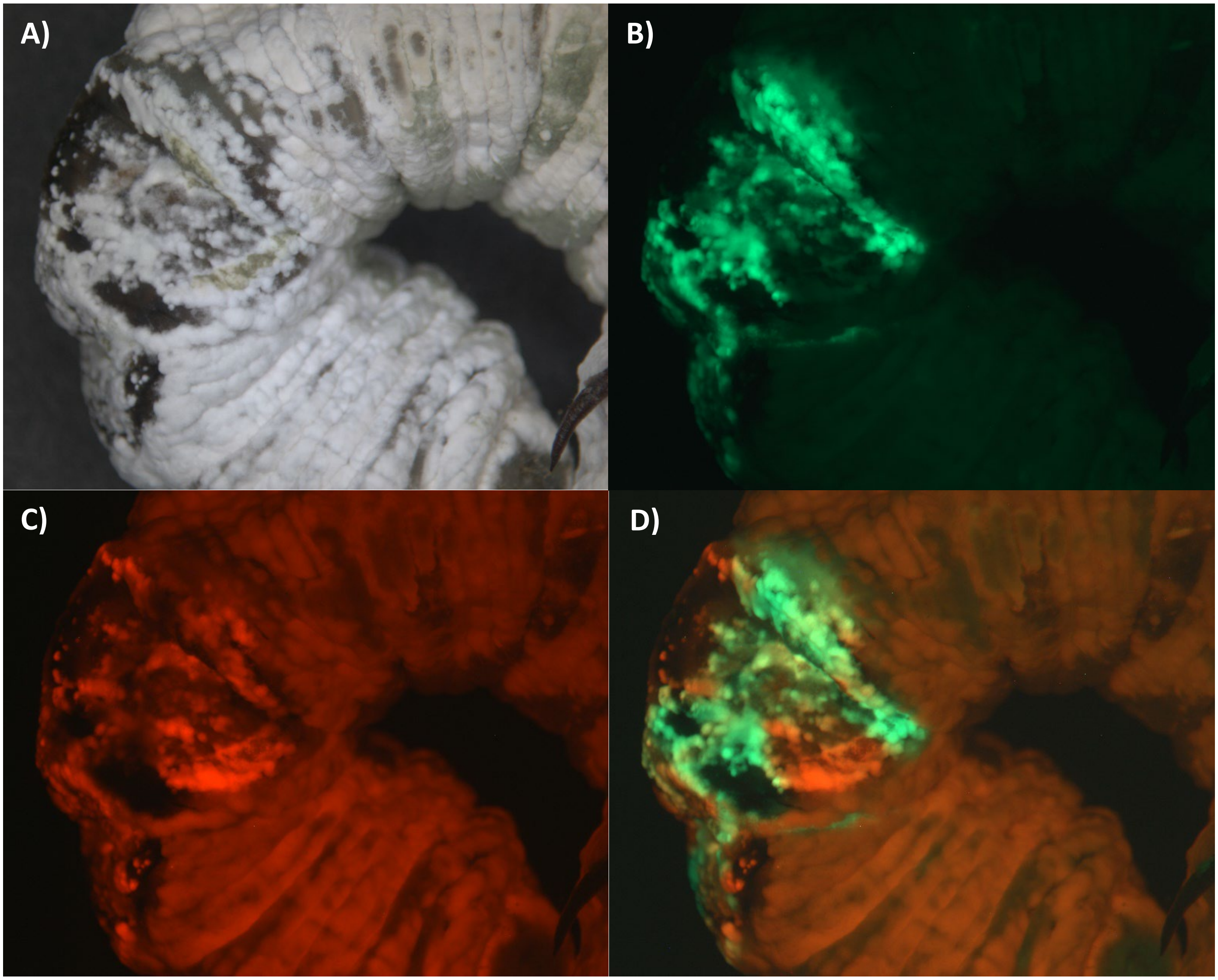

Sfig 4E ii) Bright field, GFP, Cherry and overlay for Fig 10 panel D.

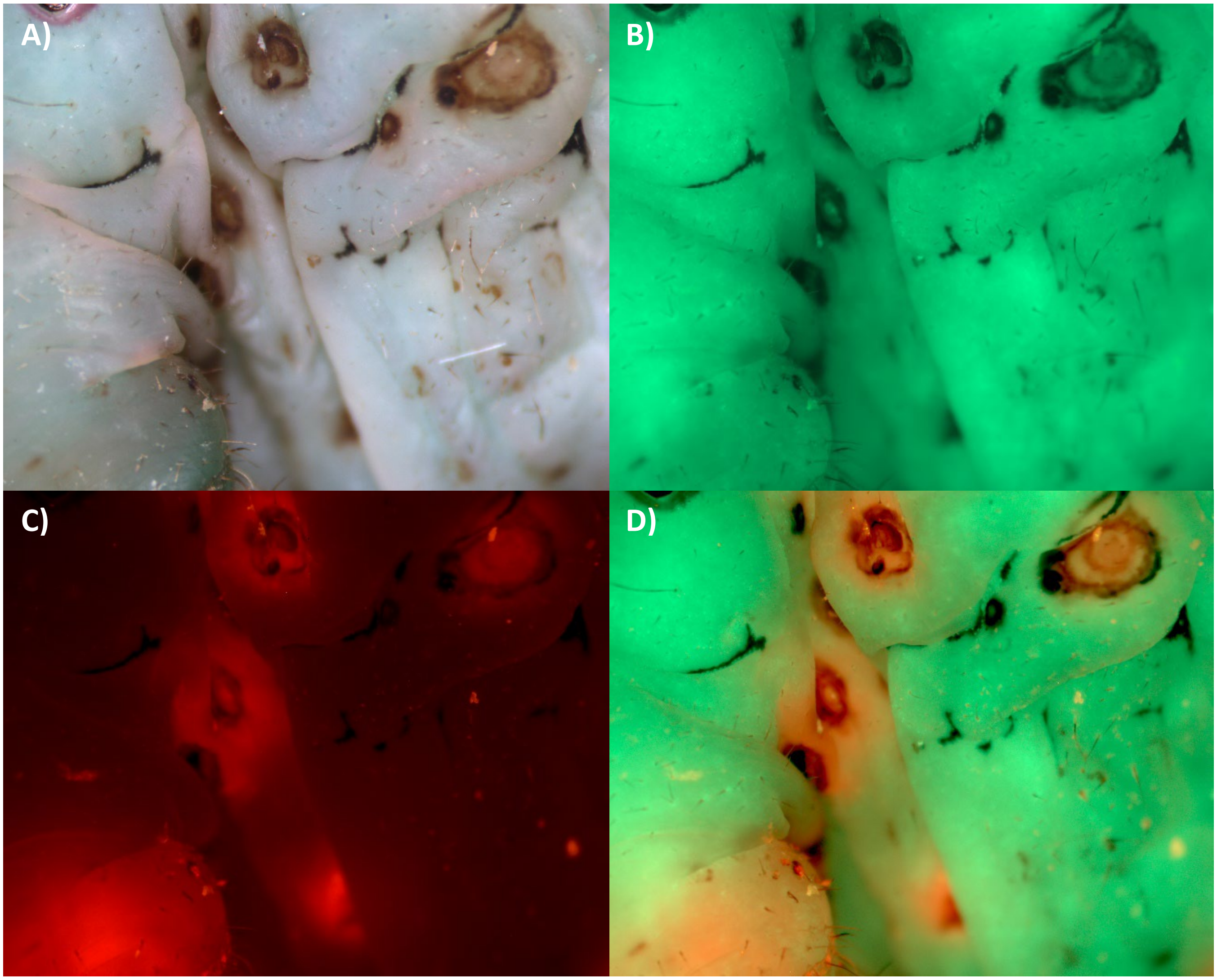

SFig 4E iii) Bright field, GFP, Cherry and overlay for Fig 10 panel F

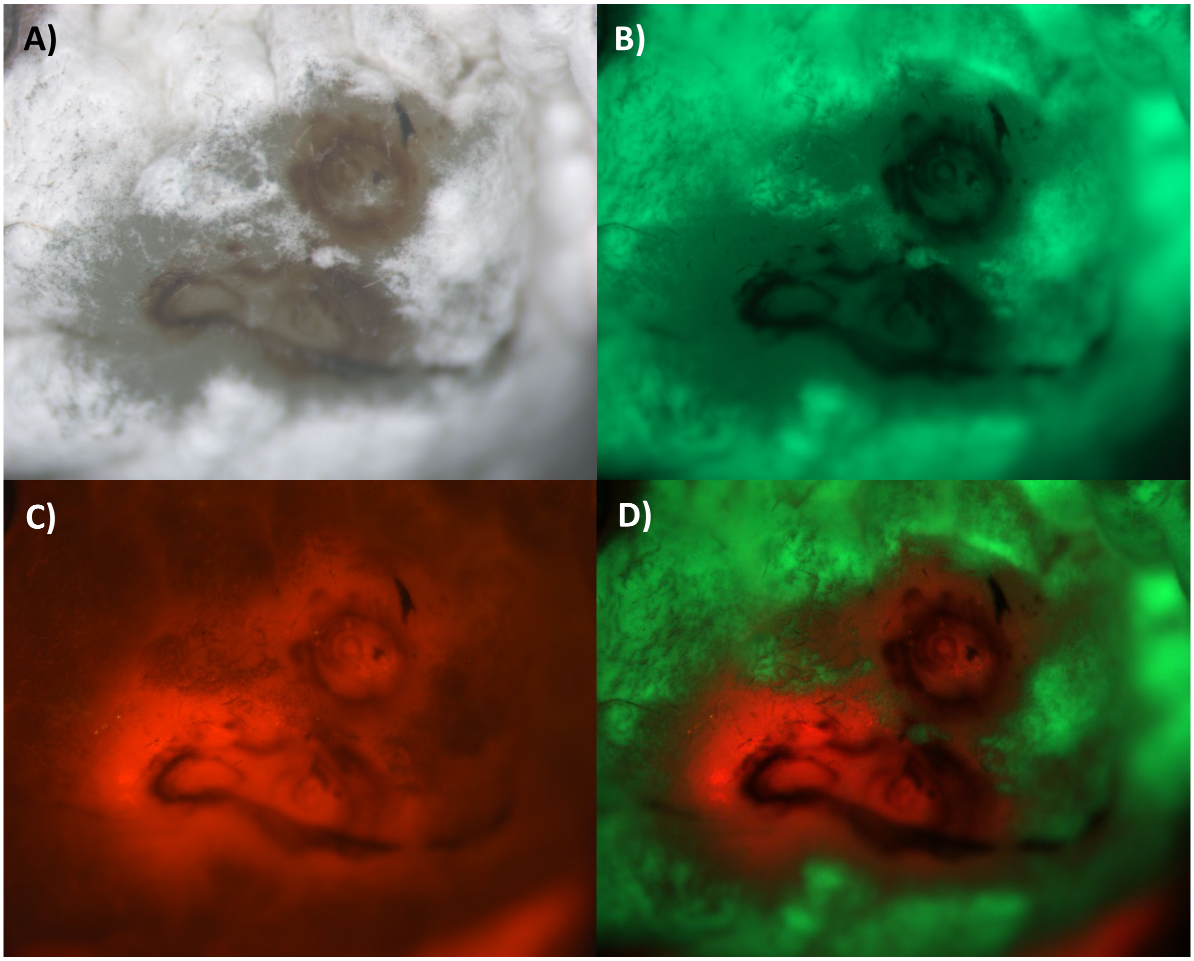

SFig 4E iv) Bright field, GFP, Cherry and overlay for Fig 10 panel H

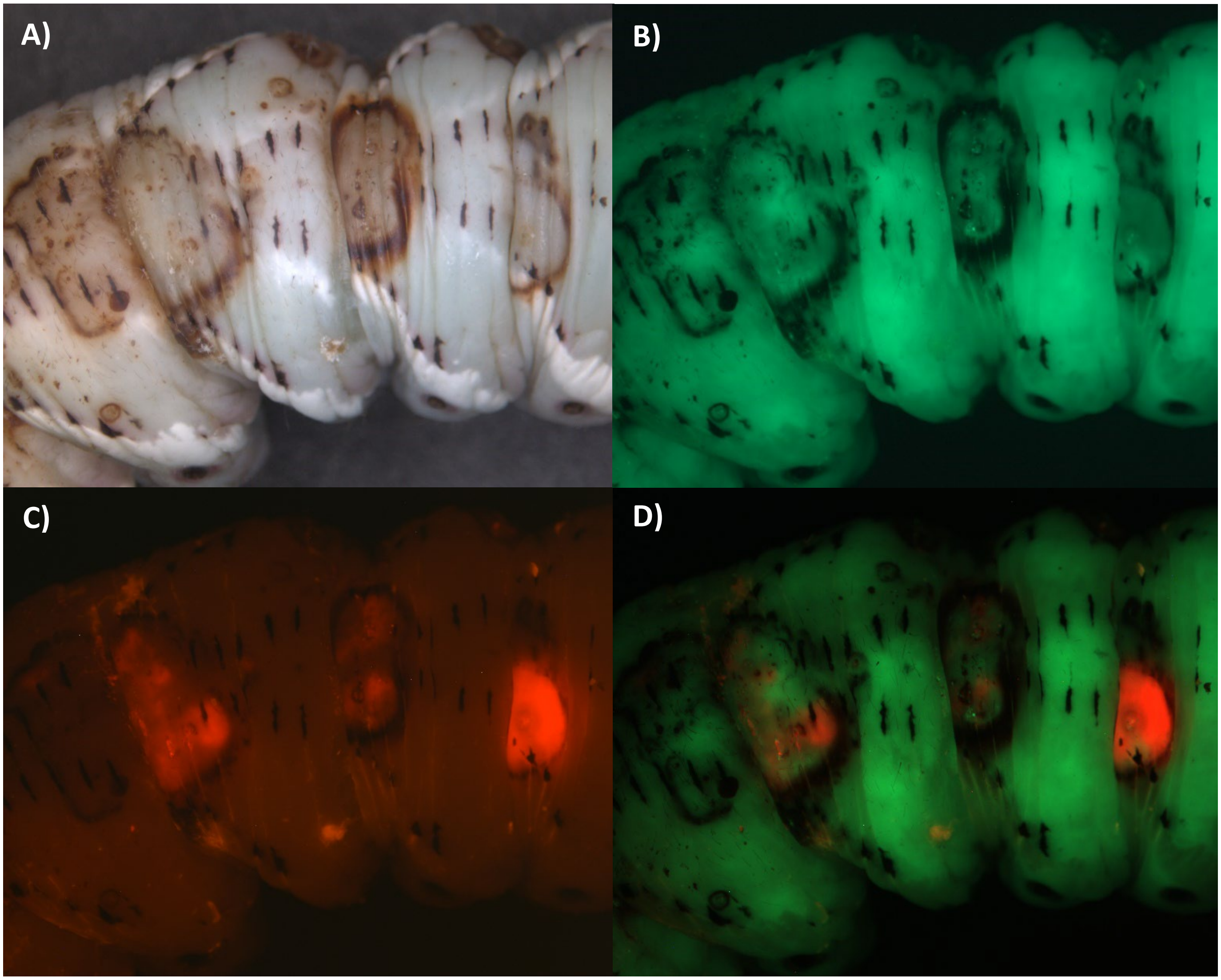

SFig E v) Bright field, GFP, Cherry and overlay for Fig 10 panel J

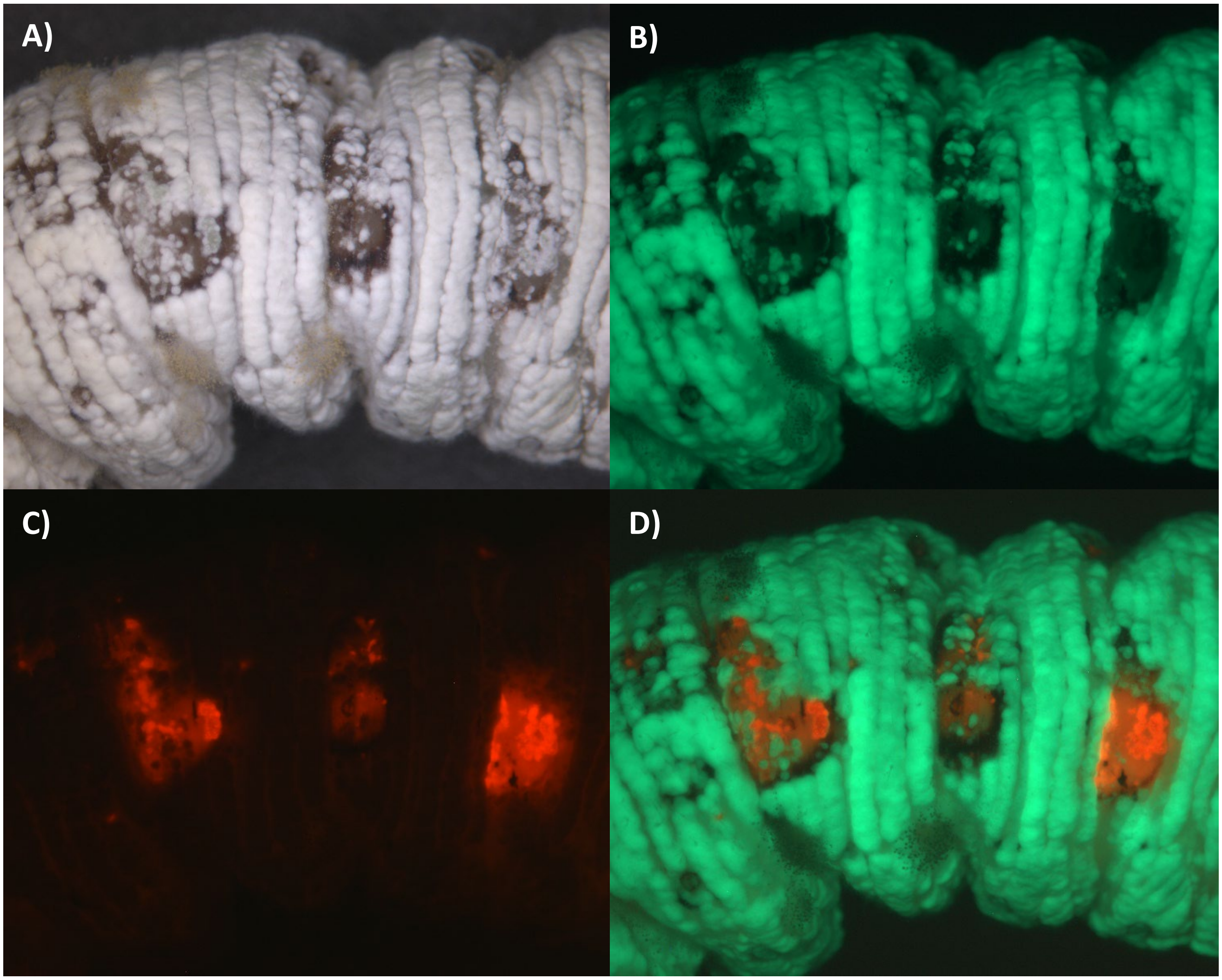

SFig 4F i) Bright field, GFP, Cherry and overlay for Fig 11 panel B

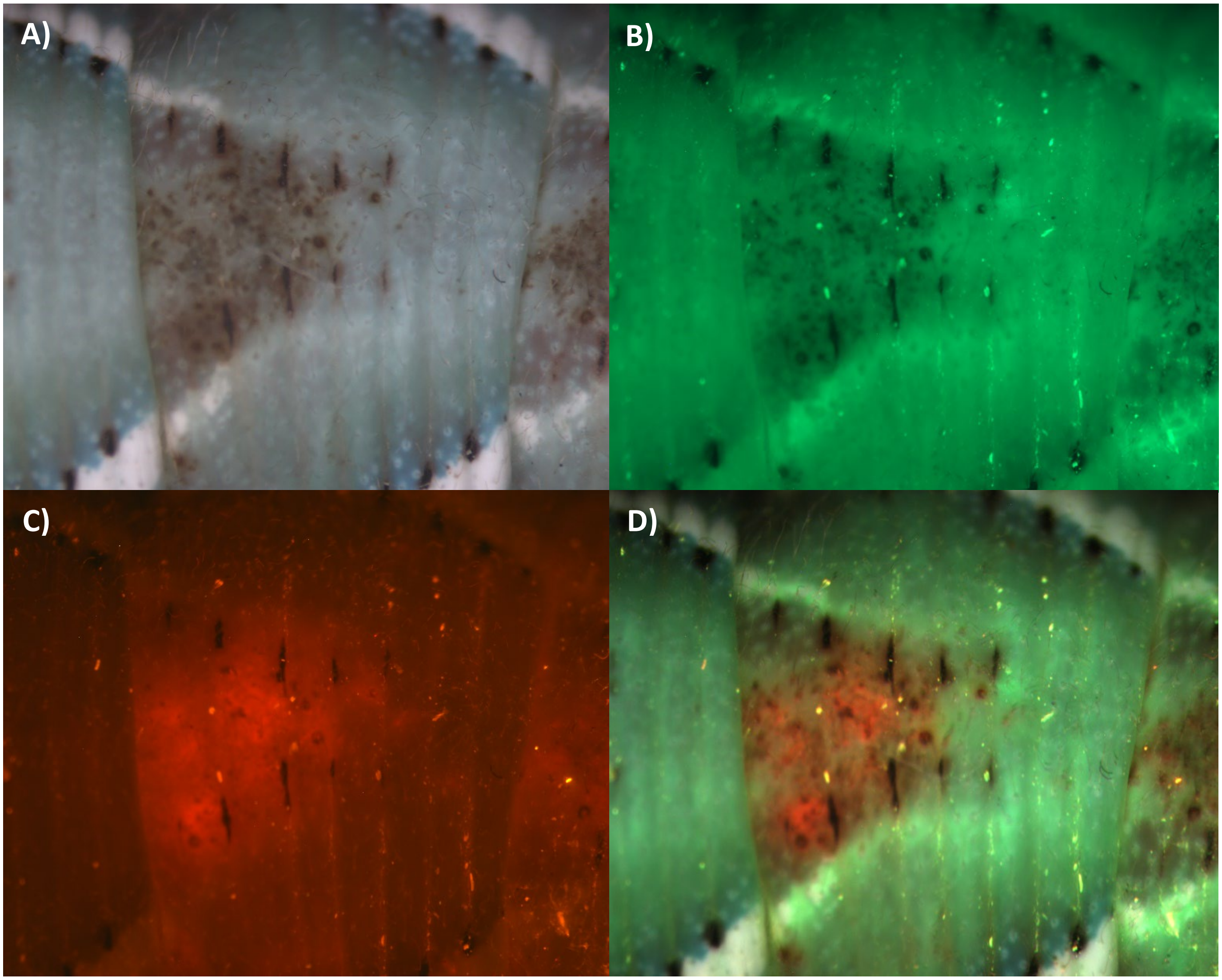

SFig 4F ii) Bright field, GFP, Cherry and overlay for Fig 11 panel D

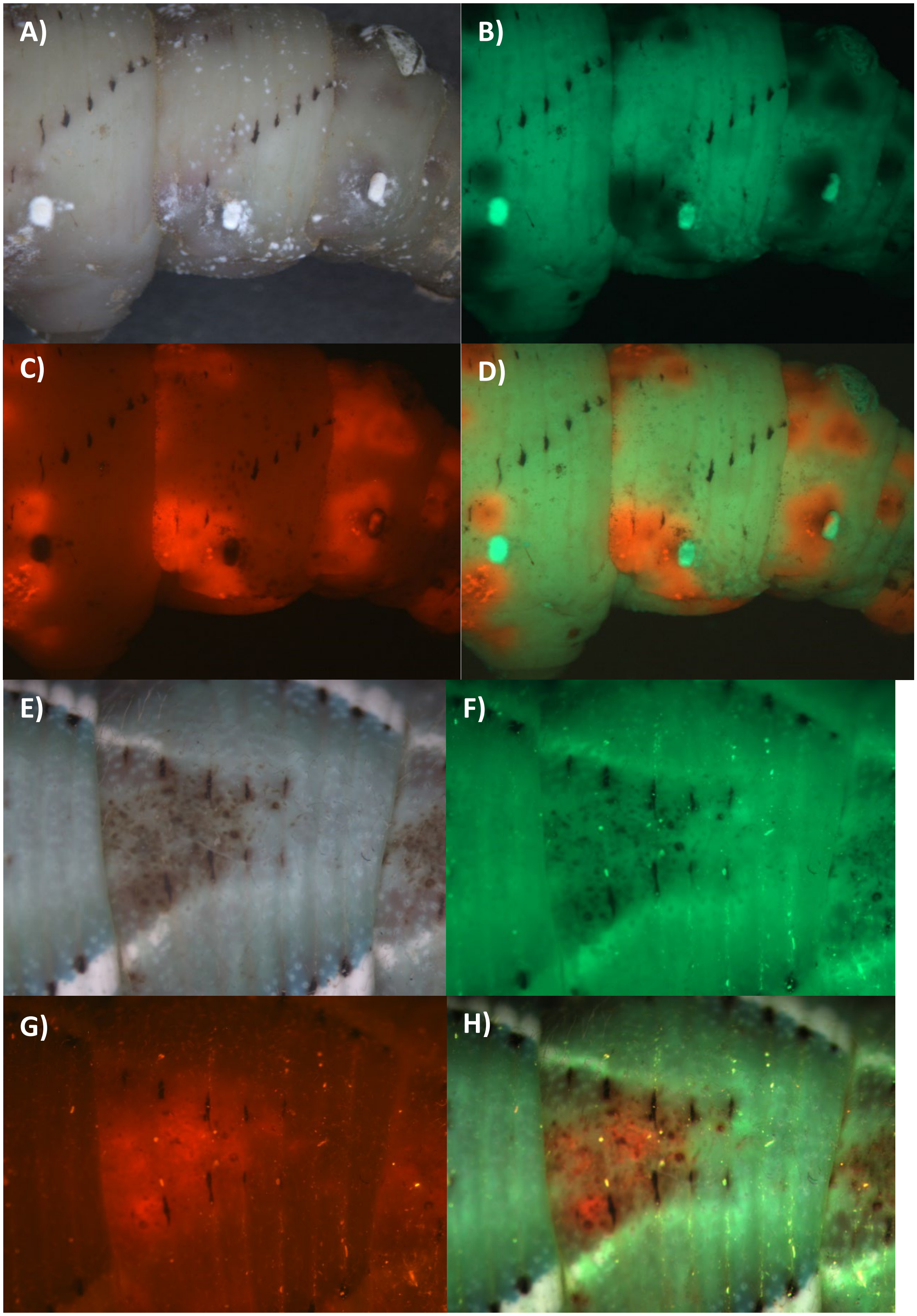

SFig 4F iii) Bright field, GFP, Cherry and overlay for Fig 11 panel F

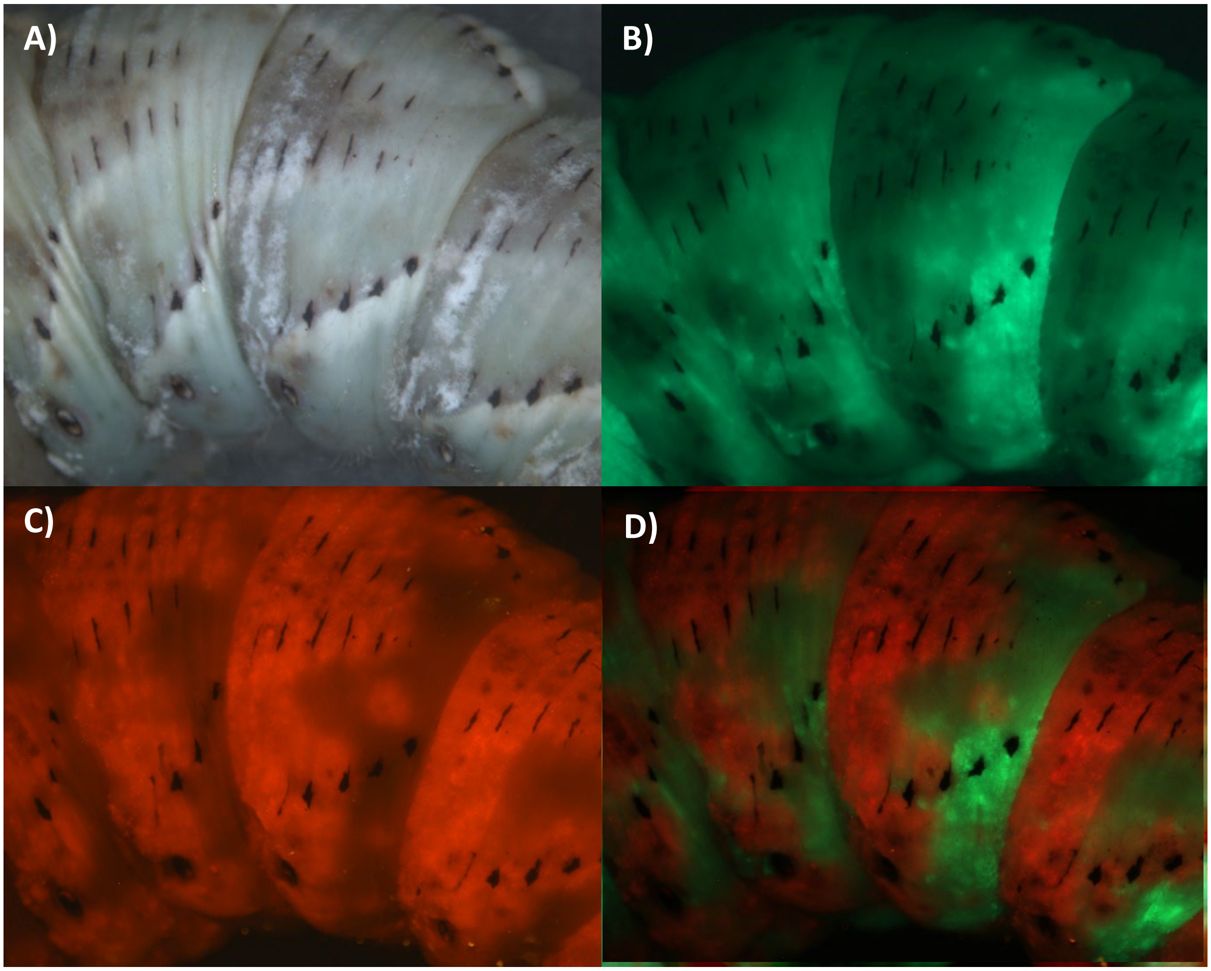

SFig 4Gi) Bright field, GFP, Cherry and overlay for Fig 12 panel F

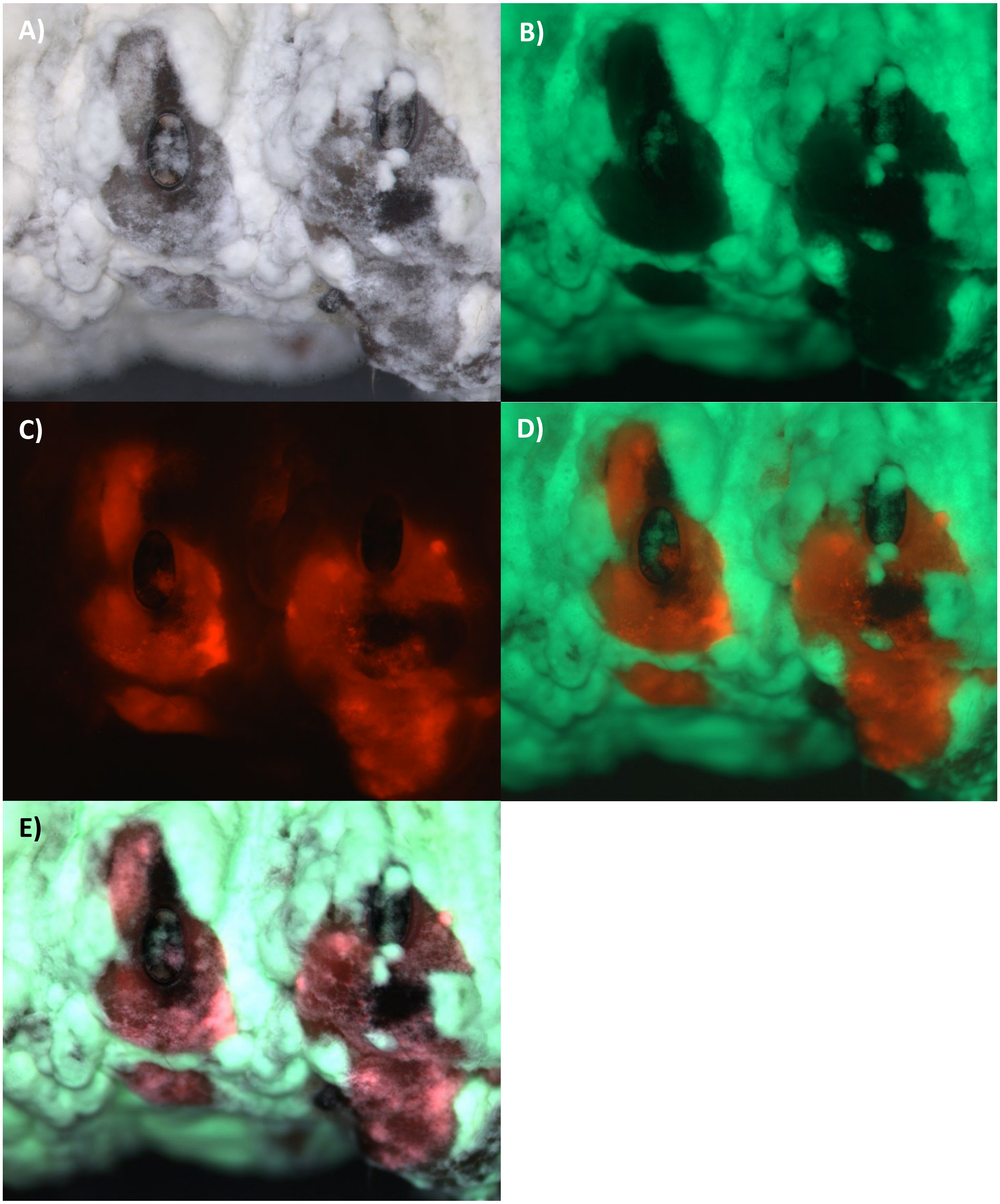

SFig 4Gii) Bright field, GFP, Cherry, GFP/Cherry overlay and bright field/GFP/Cherry overlay of a cadaver showing localization of Mr2575 to the spiracles but Ma549 localized around the trachea.

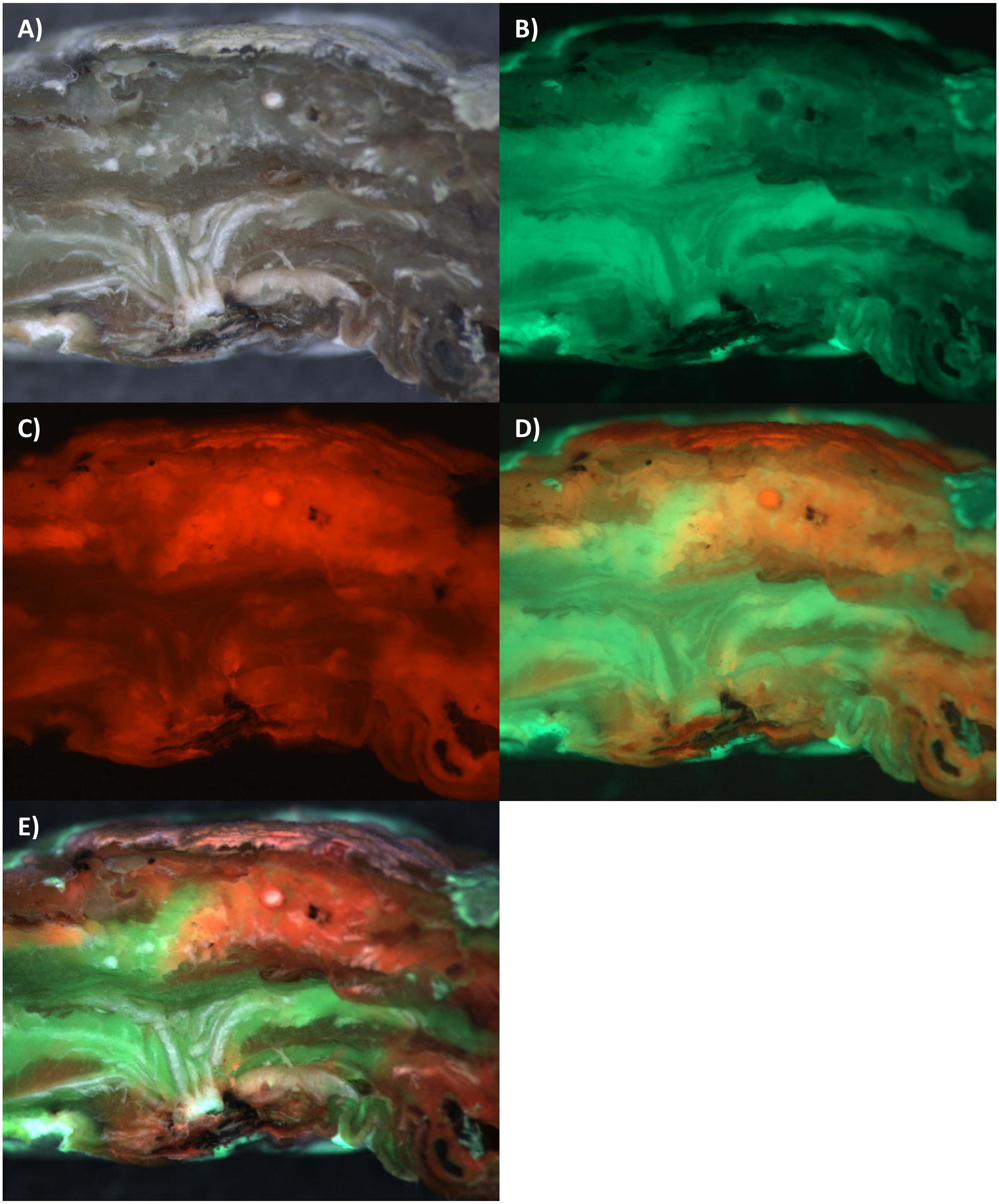

SFig 4Giii) Bright field, GFP, Cherry, GFP/Cherry overlay and bright field/GFP/Cherry overlay of a cadaver showing localization of Mr2575 to the spiracles and trachea.

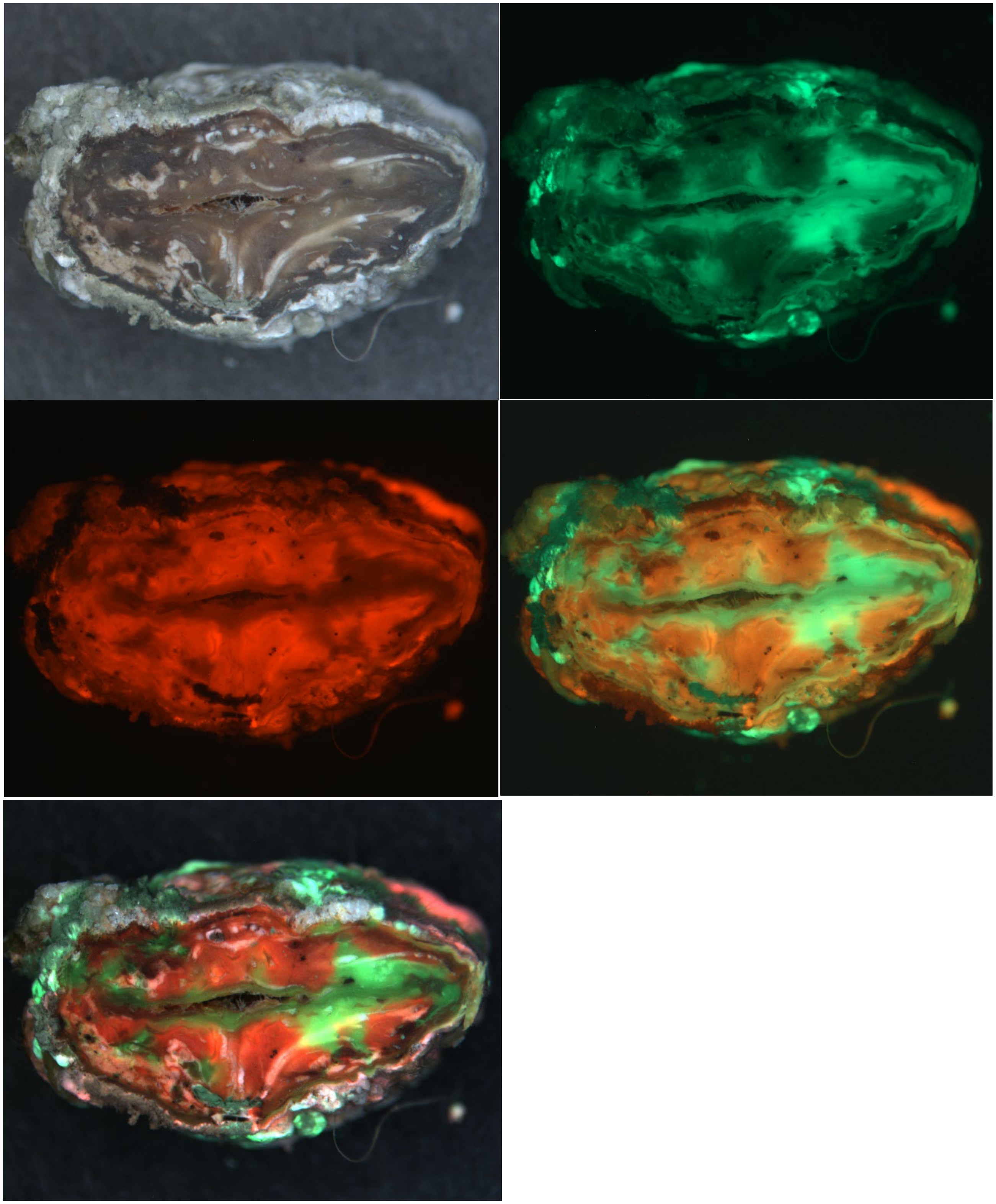

SFig 4H i) Bright field, GFP, Cherry and overlay for Fig 13 panel B

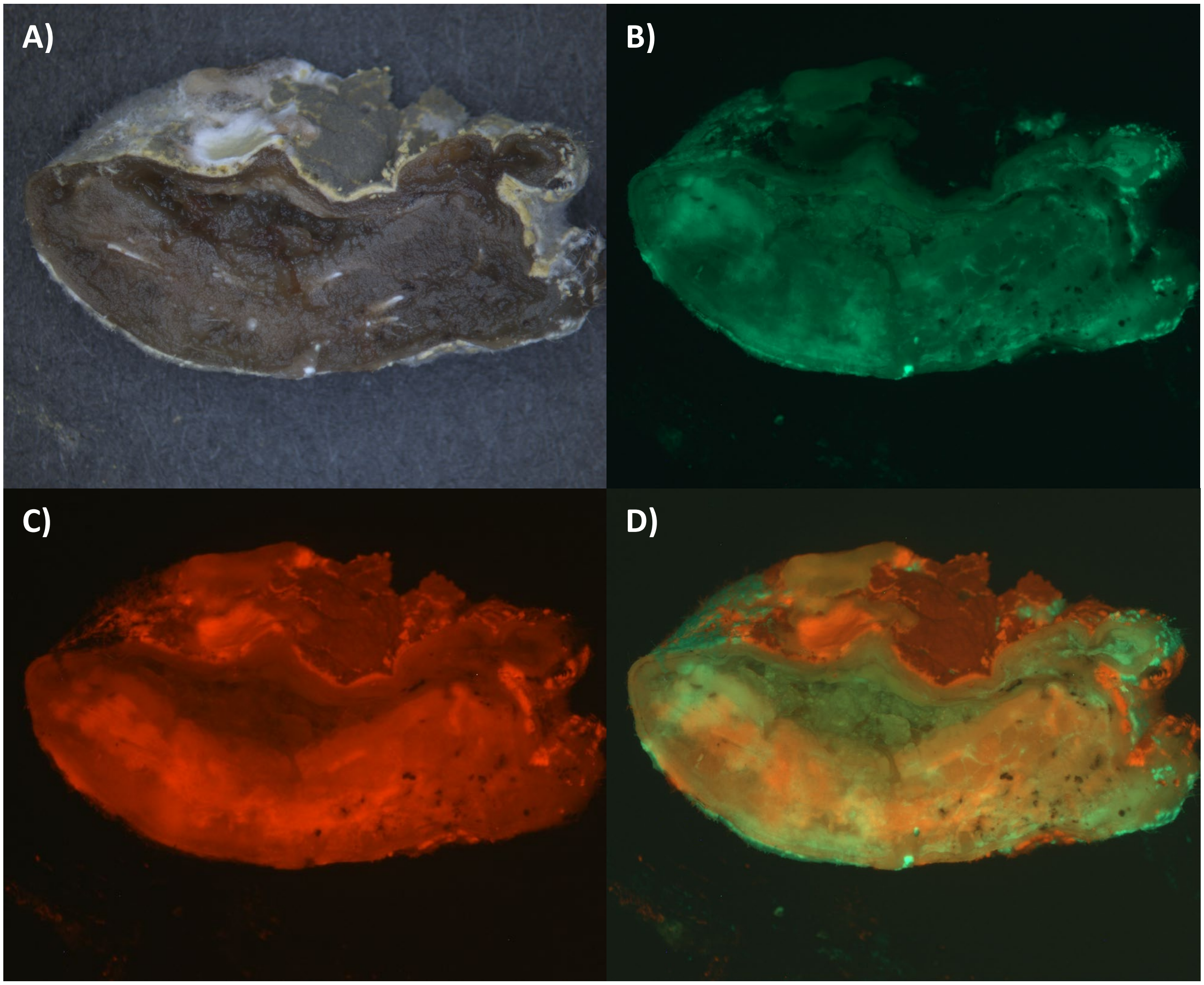

SFig 4H ii) Bright field, GFP, Cherry and overlay for Fig 13 panel D

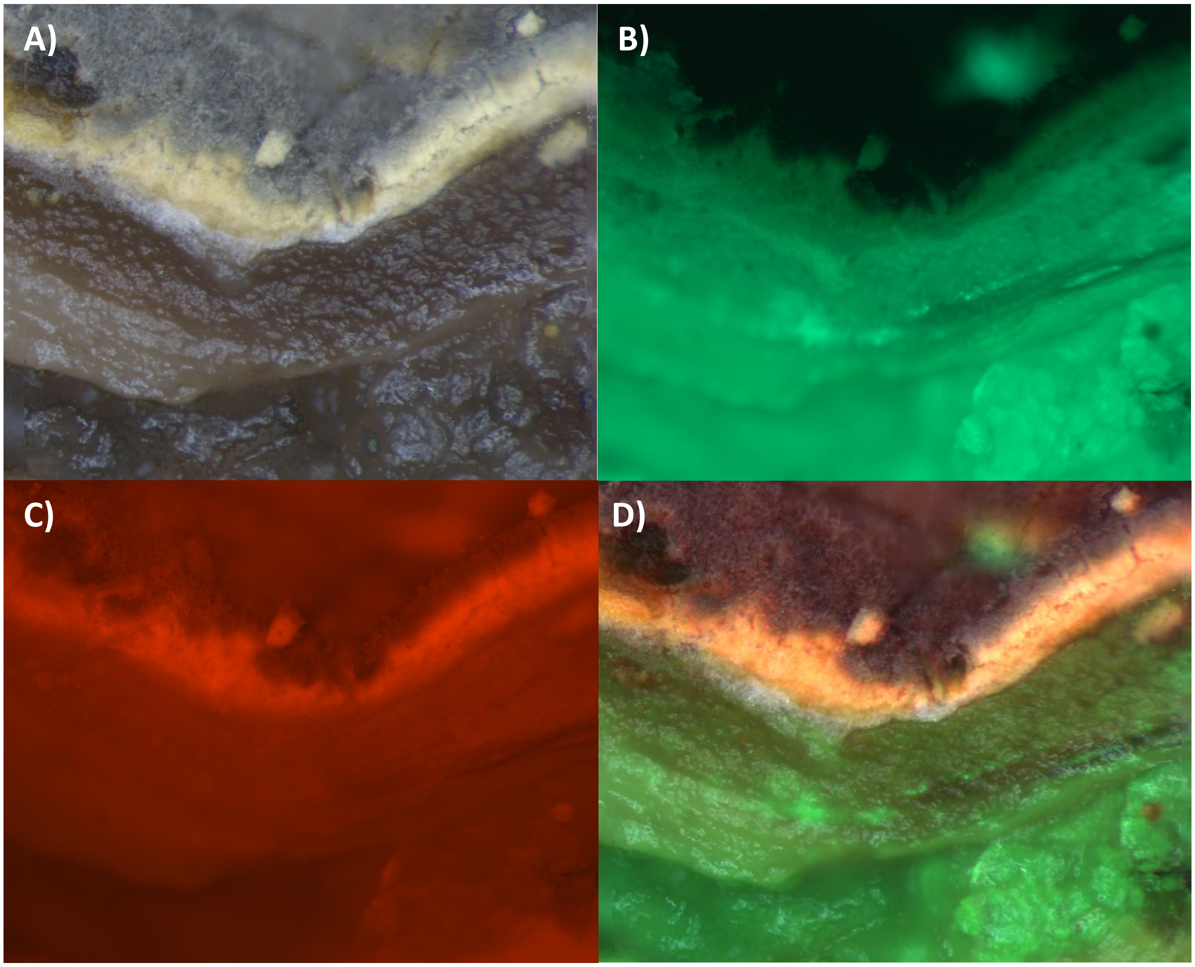

SFig 4H iii) Bright field, GFP, Cherry and overlay for Fig 13 panel F

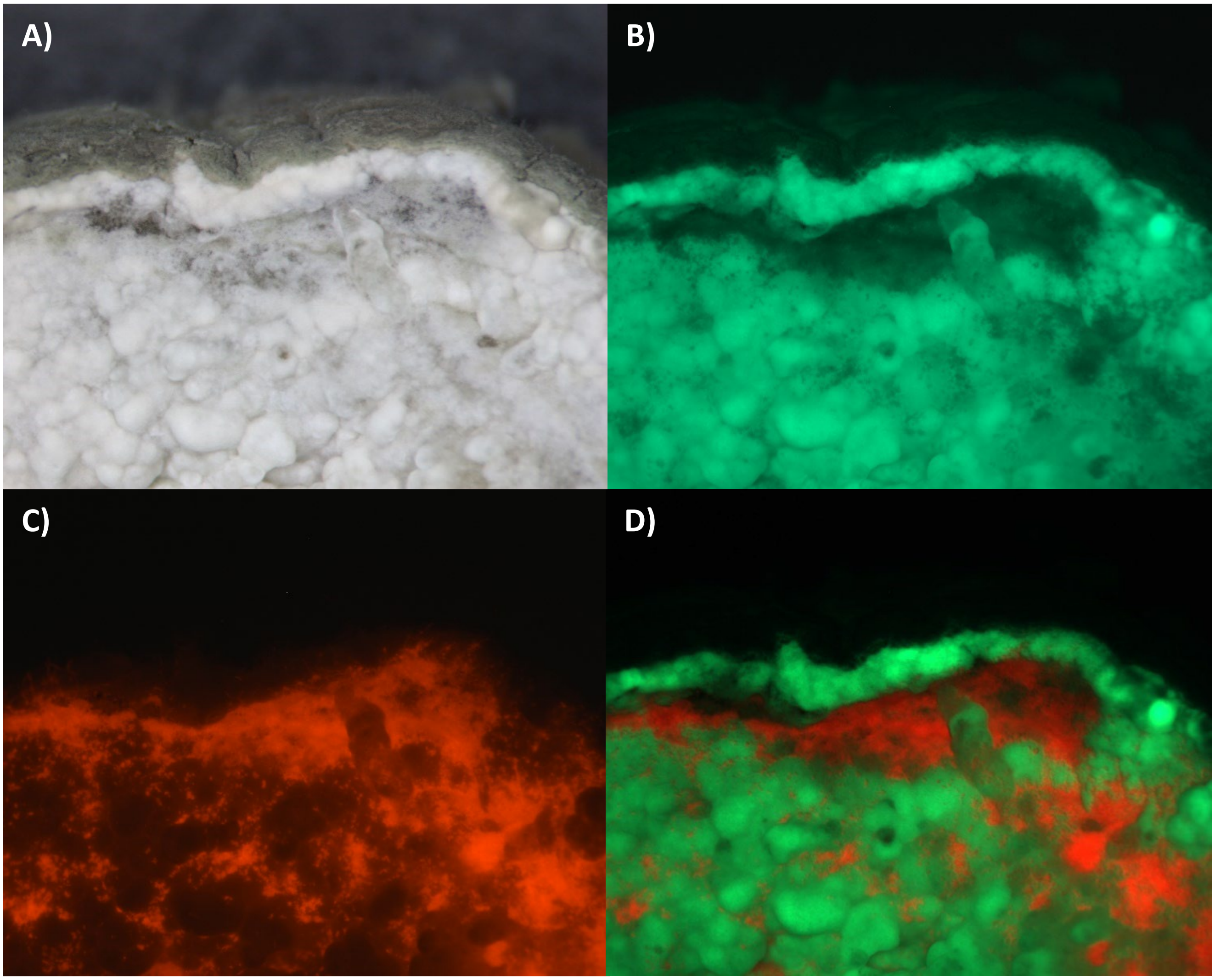

SFig 4H iv) Bright field, GFP, Cherry and overlay for Fig 13 panel H

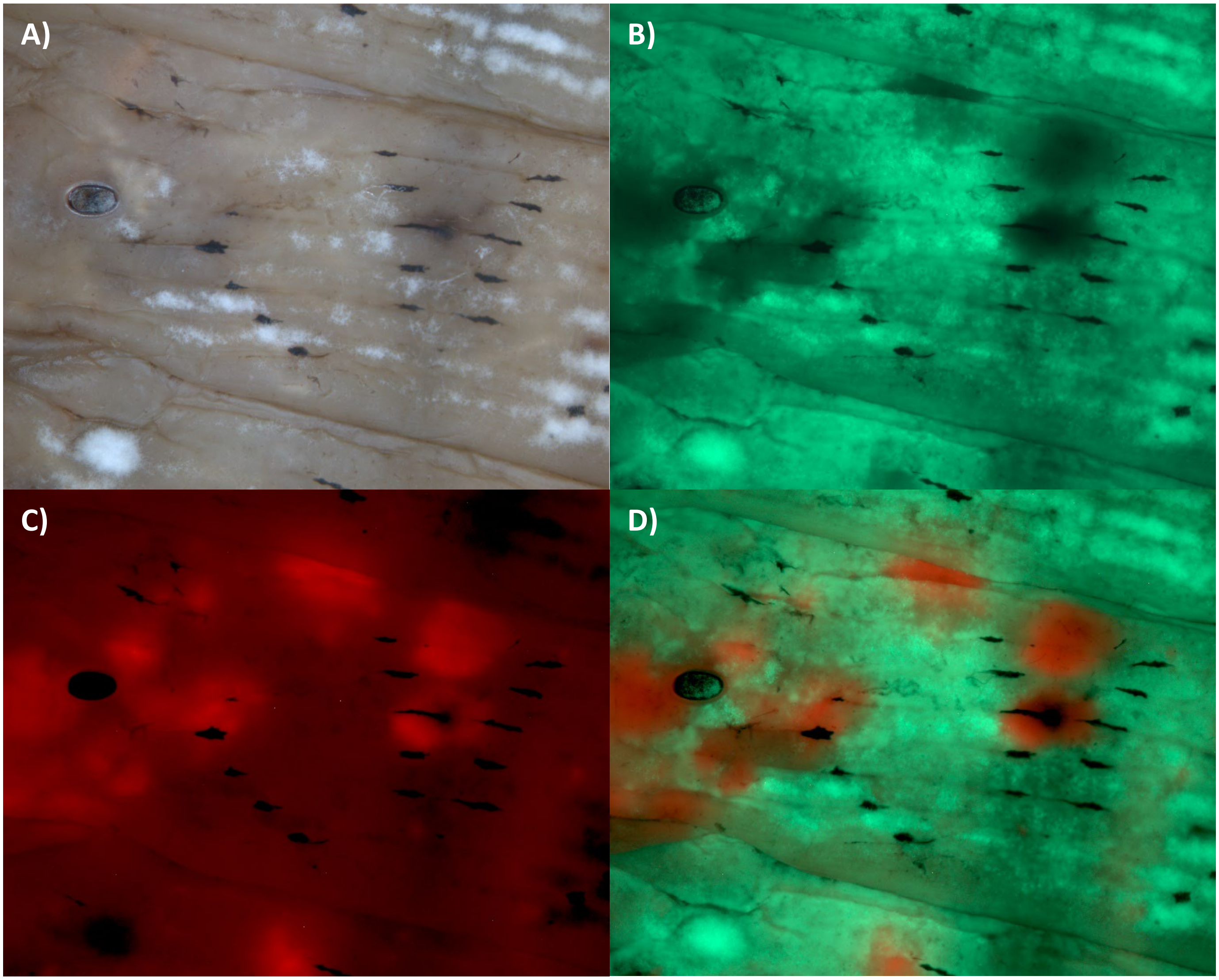

SFig 4H v) Bright field, GFP, Cherry and overlay for Fig 13 panel J

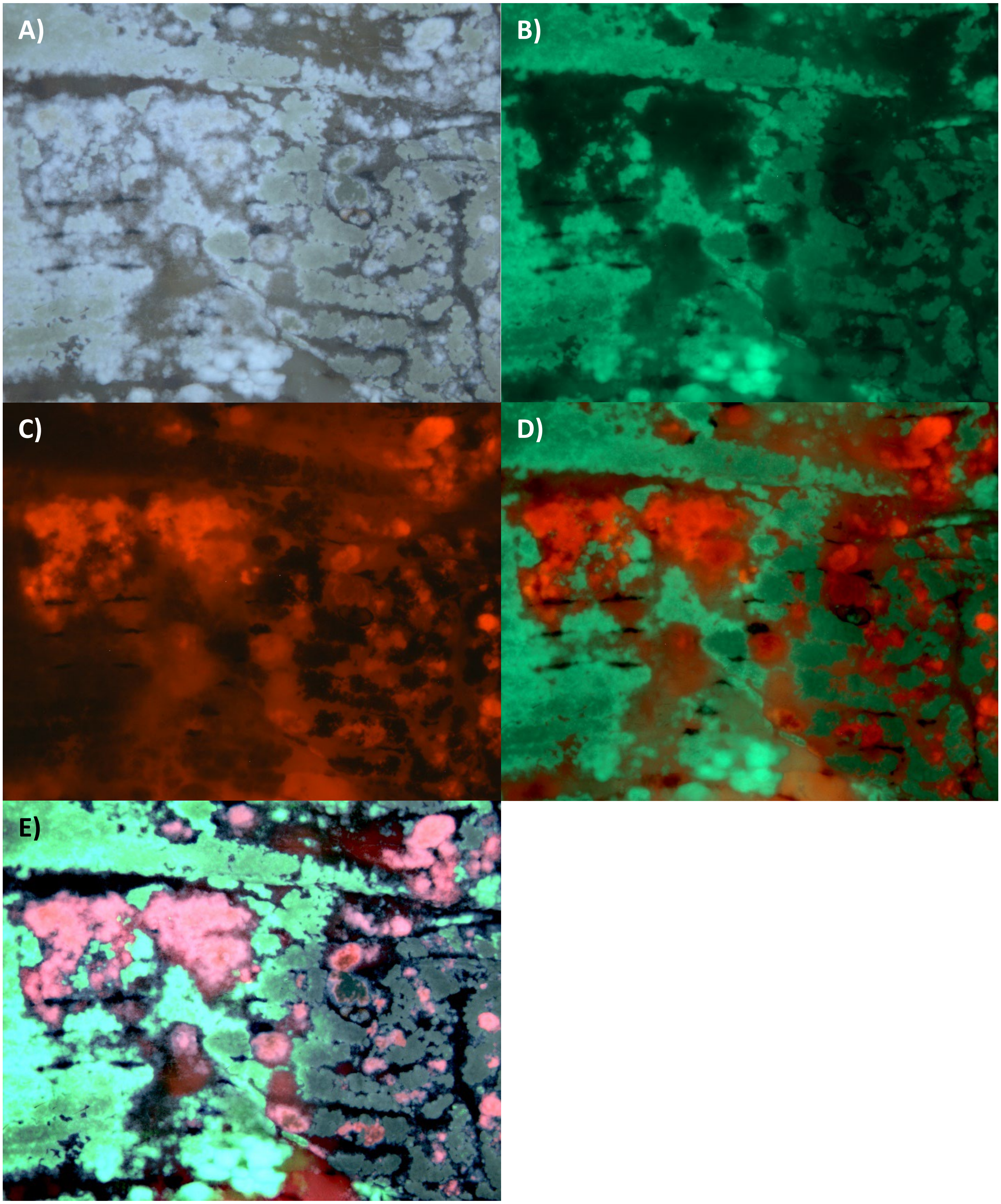

SFig 4I i) Bright field, GFP, Cherry and overlay for Fig 14 panel B

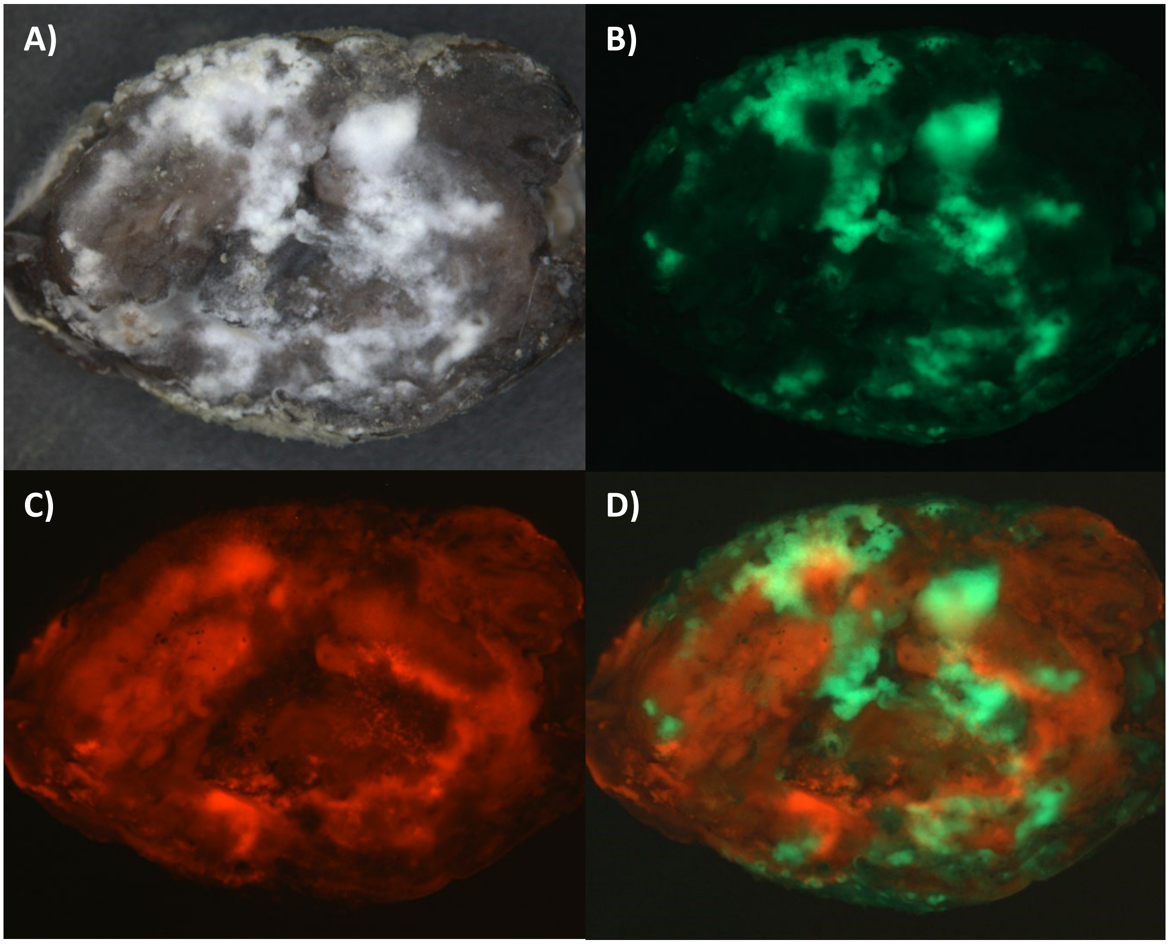

SFig 4I ii) Bright field, GFP, Cherry and overlay for Fig 14 panel D

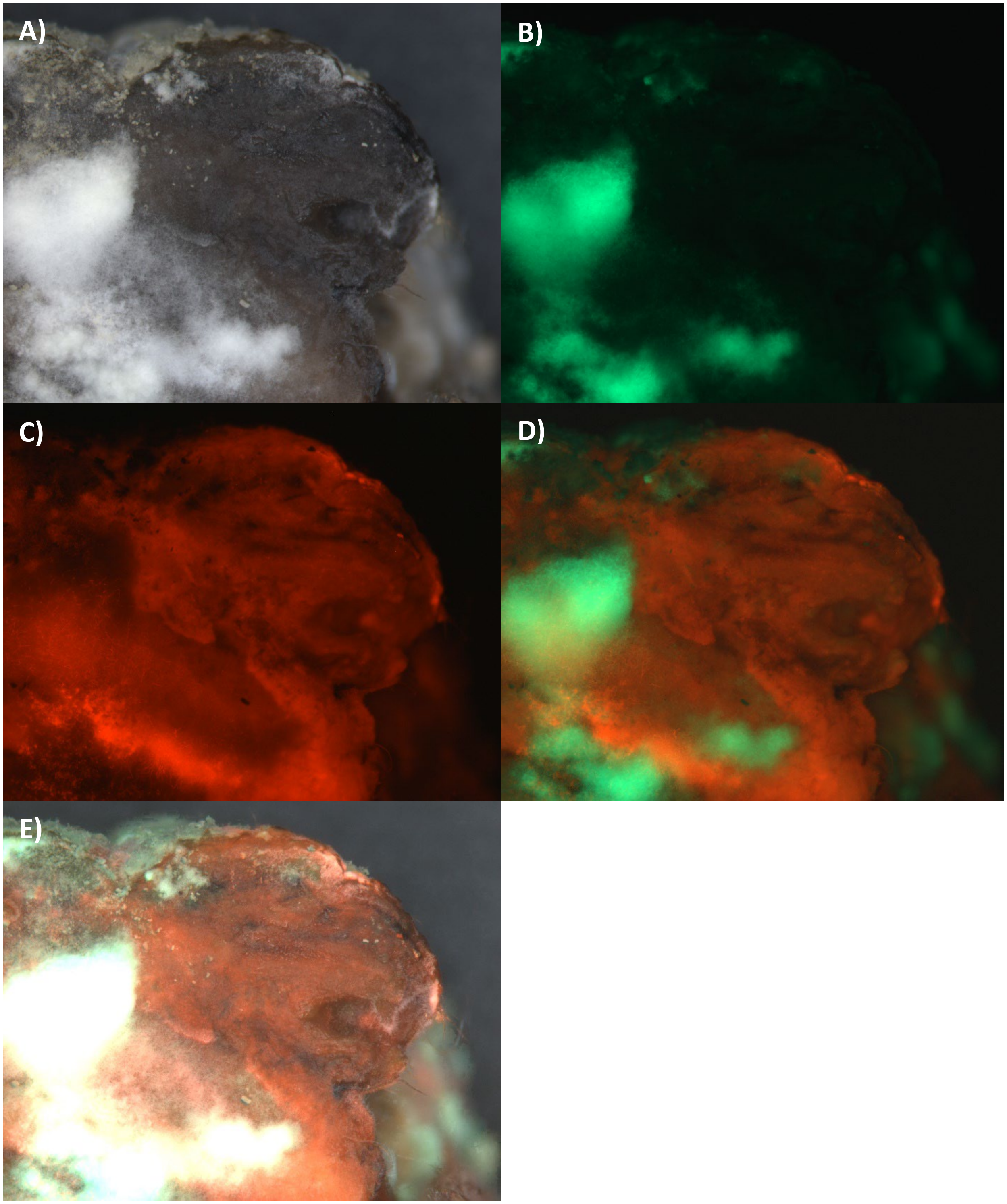

SFig 4I iii) Bright field, GFP, Cherry and overlay for Fig 14 panel F

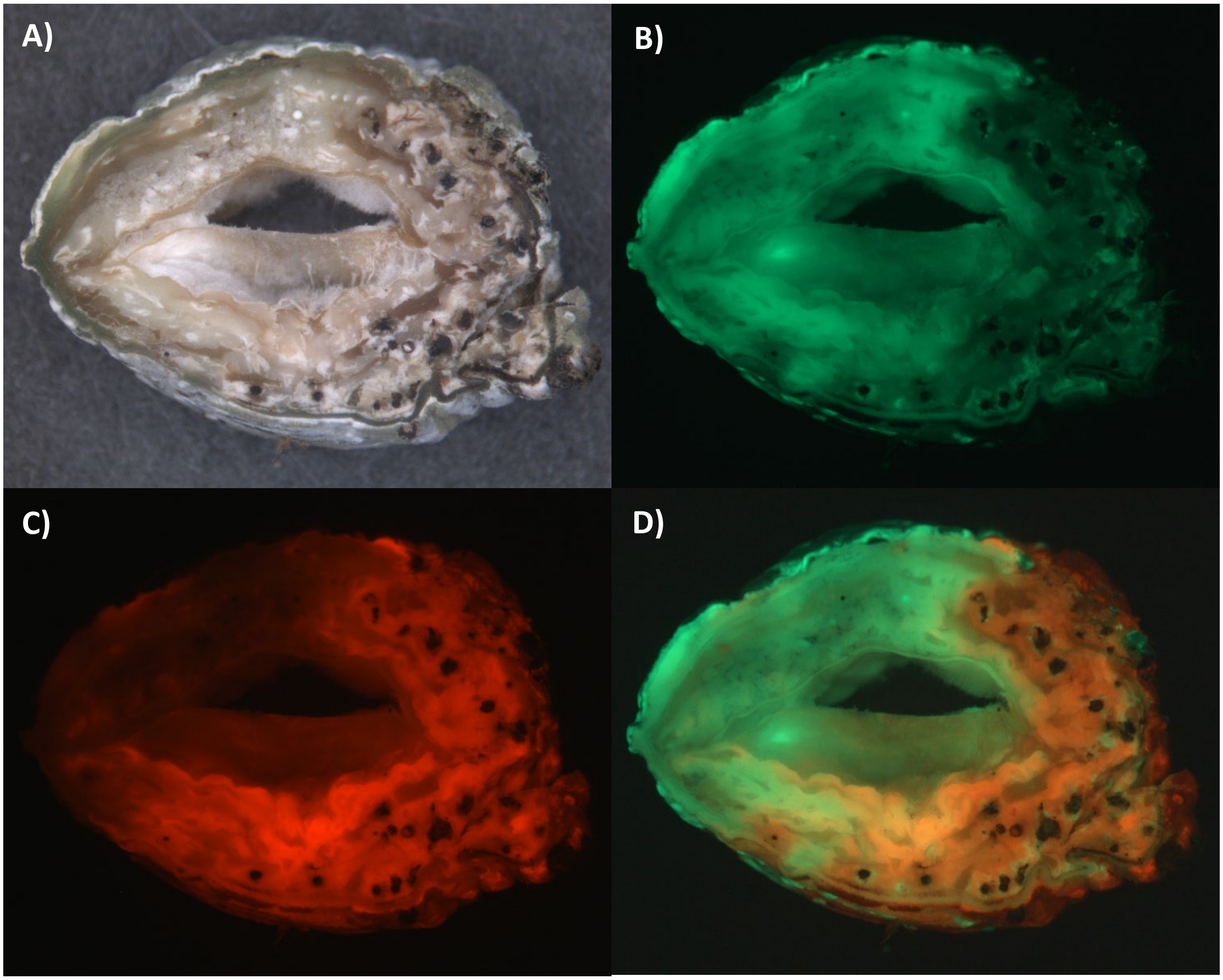

SFig 4I iv) Bright field, GFP, Cherry and overlay for Fig 14 panel H

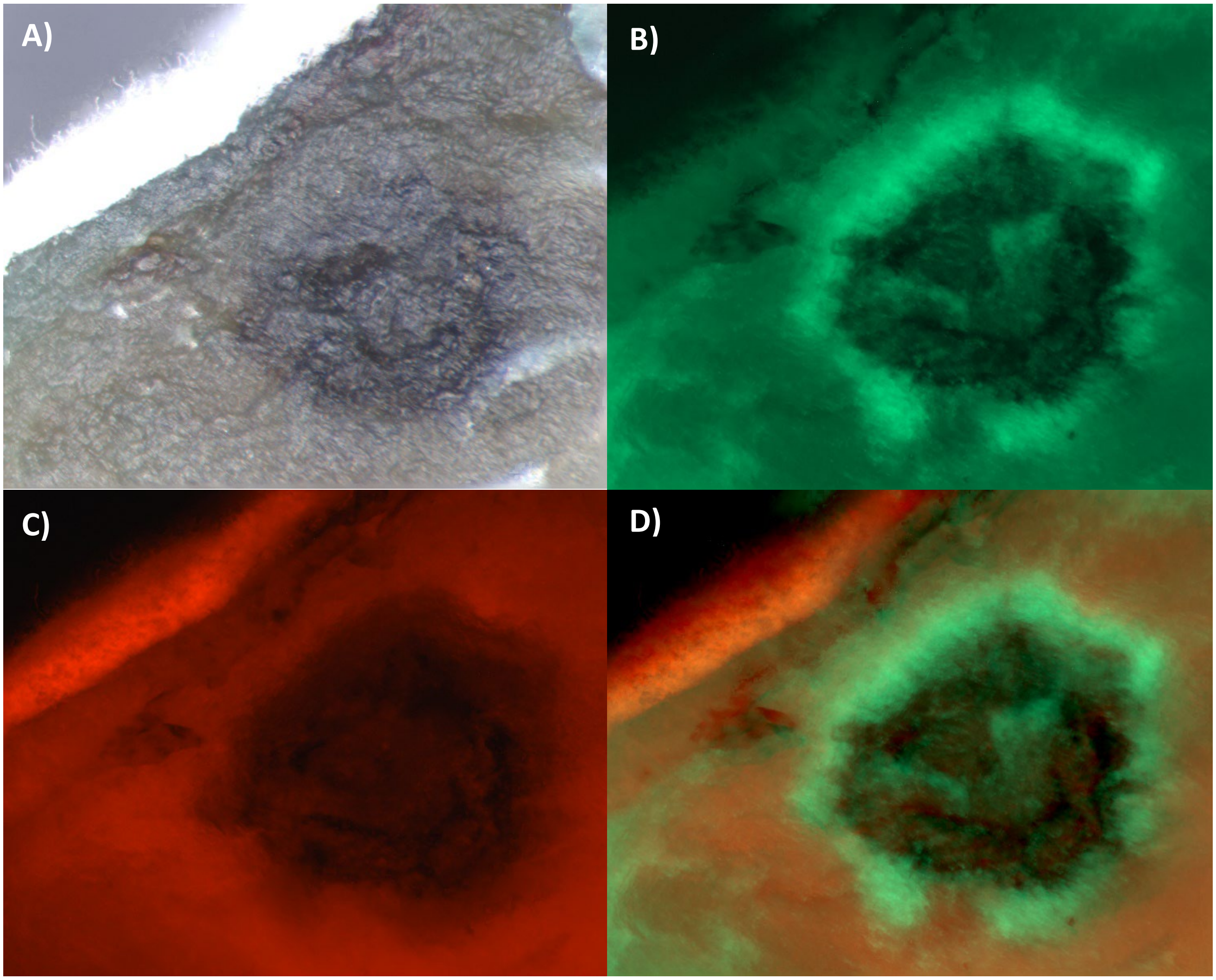

SFig 4I v) Bright field, GFP, Cherry and overlay of a cadaver section 3 days post mortem showing darkly melanized regions containing predominately Mr2575-Cherry. This is an additional example of a cadaver section with melanized domains containing Mr2575 to complement Fig. 14

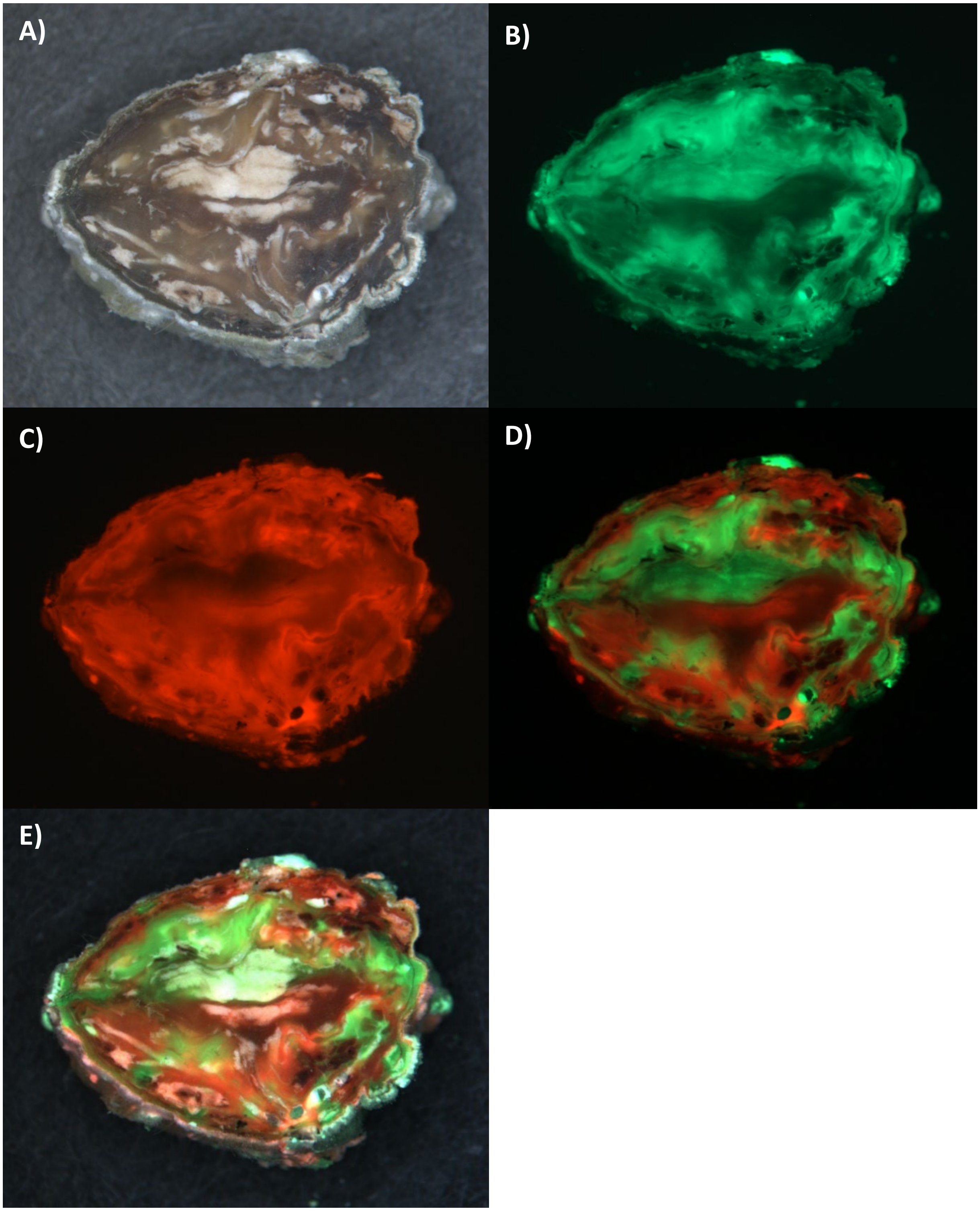

SFig 4J i) Bright field, GFP, Cherry and overlay for Fig 16 panel B

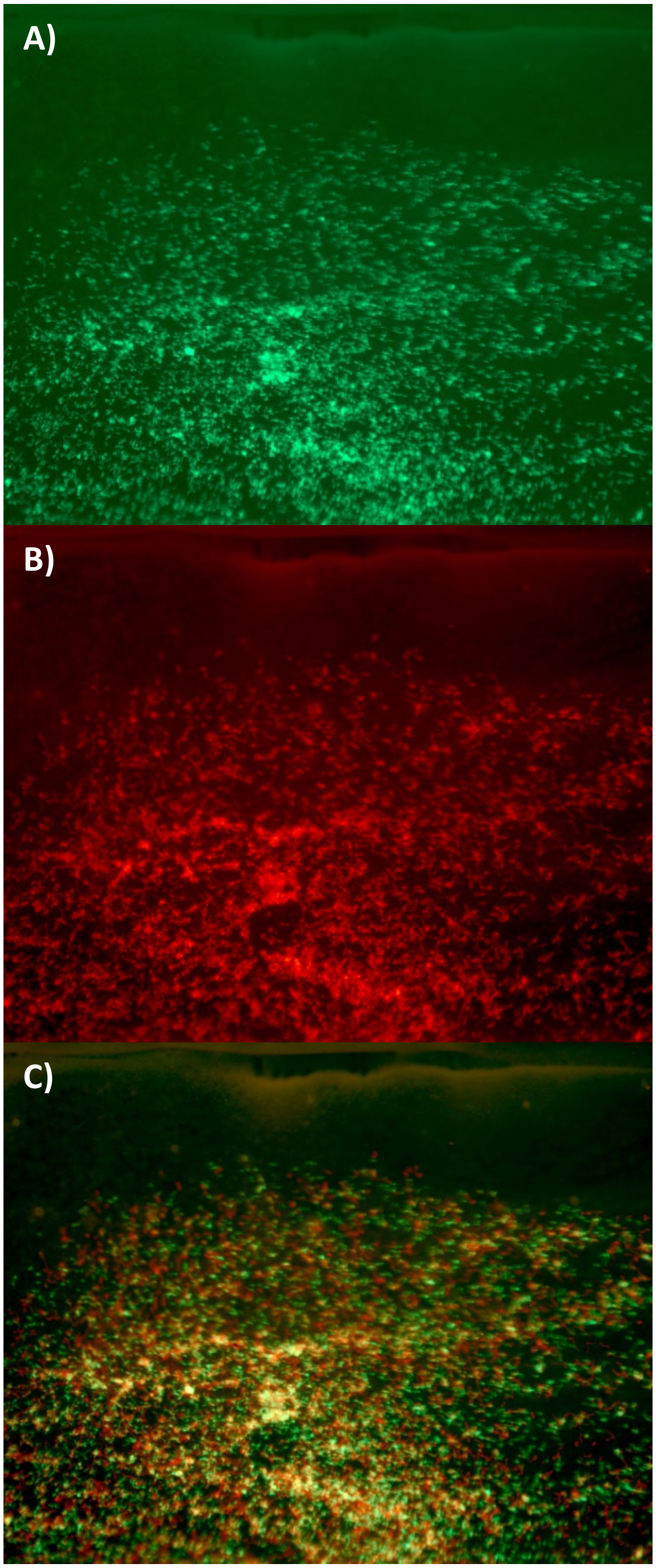

SFig 4J ii) Bright field, GFP, Cherry and overlay for Fig 16 panel

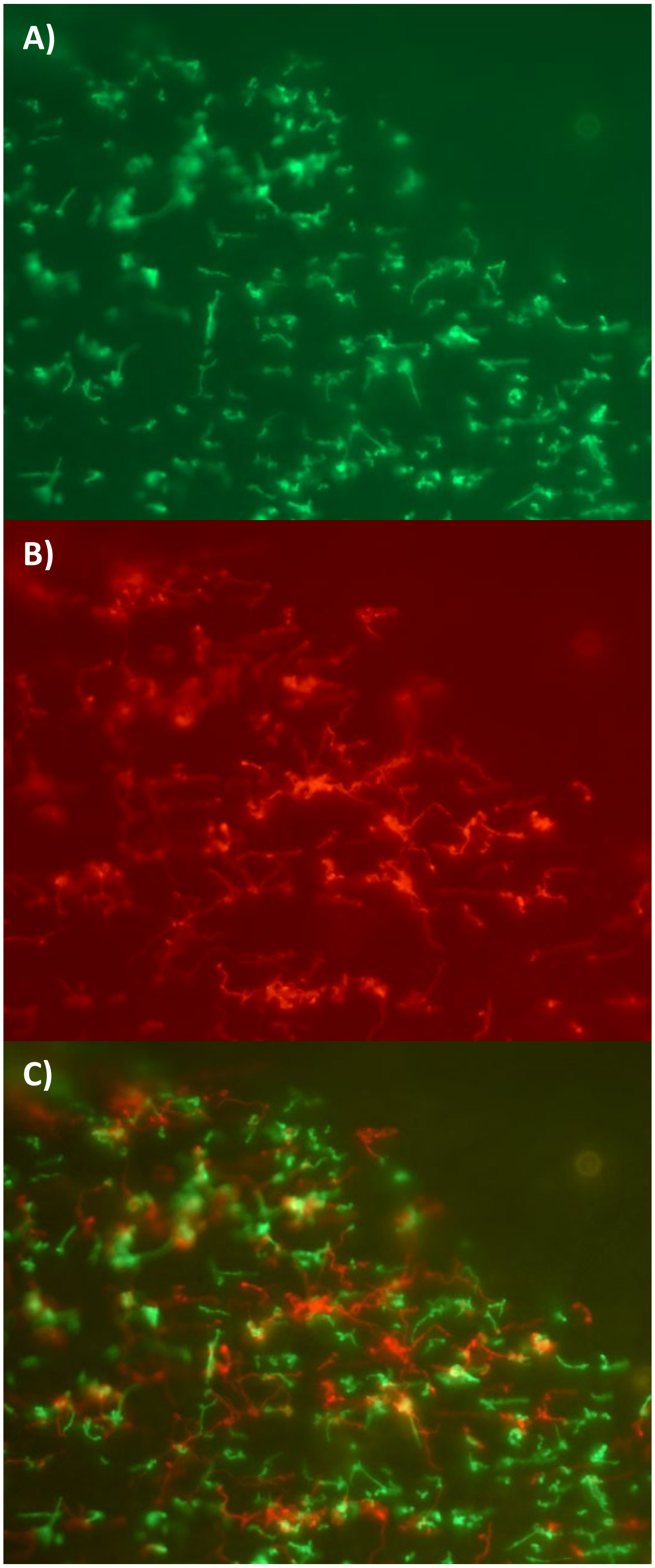

SFig 4J iii) Bright field, GFP, Cherry, GFP?Cherry overlay and Bright field/GFP/Cherry/ overlay for Fig 16 panel F

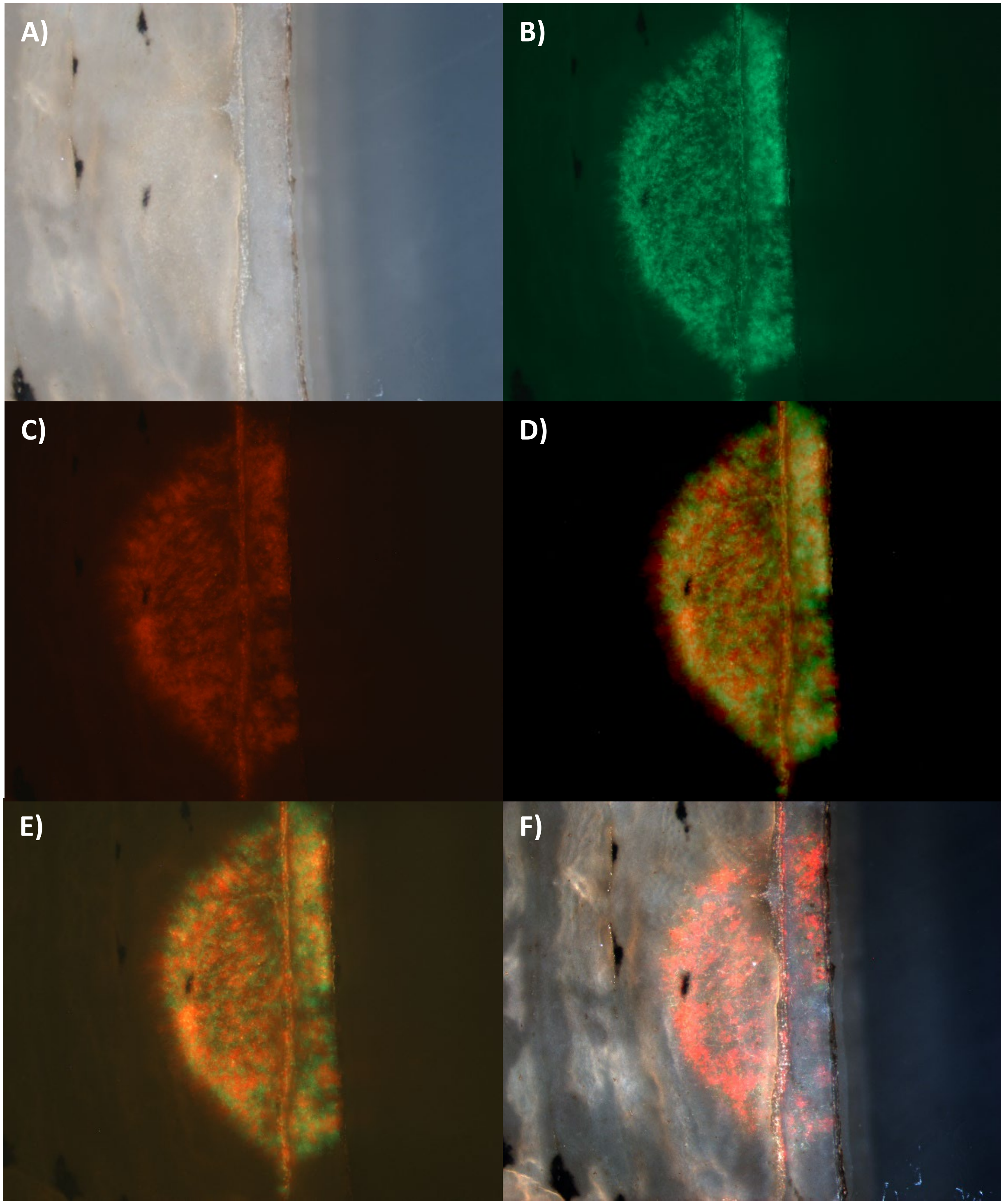

SFig 4J iv) Bright field, GFP, Cherry, GFP/Cherry overlay and Bright field/GFP/Cherry/ overlay for Fig 16 panel H

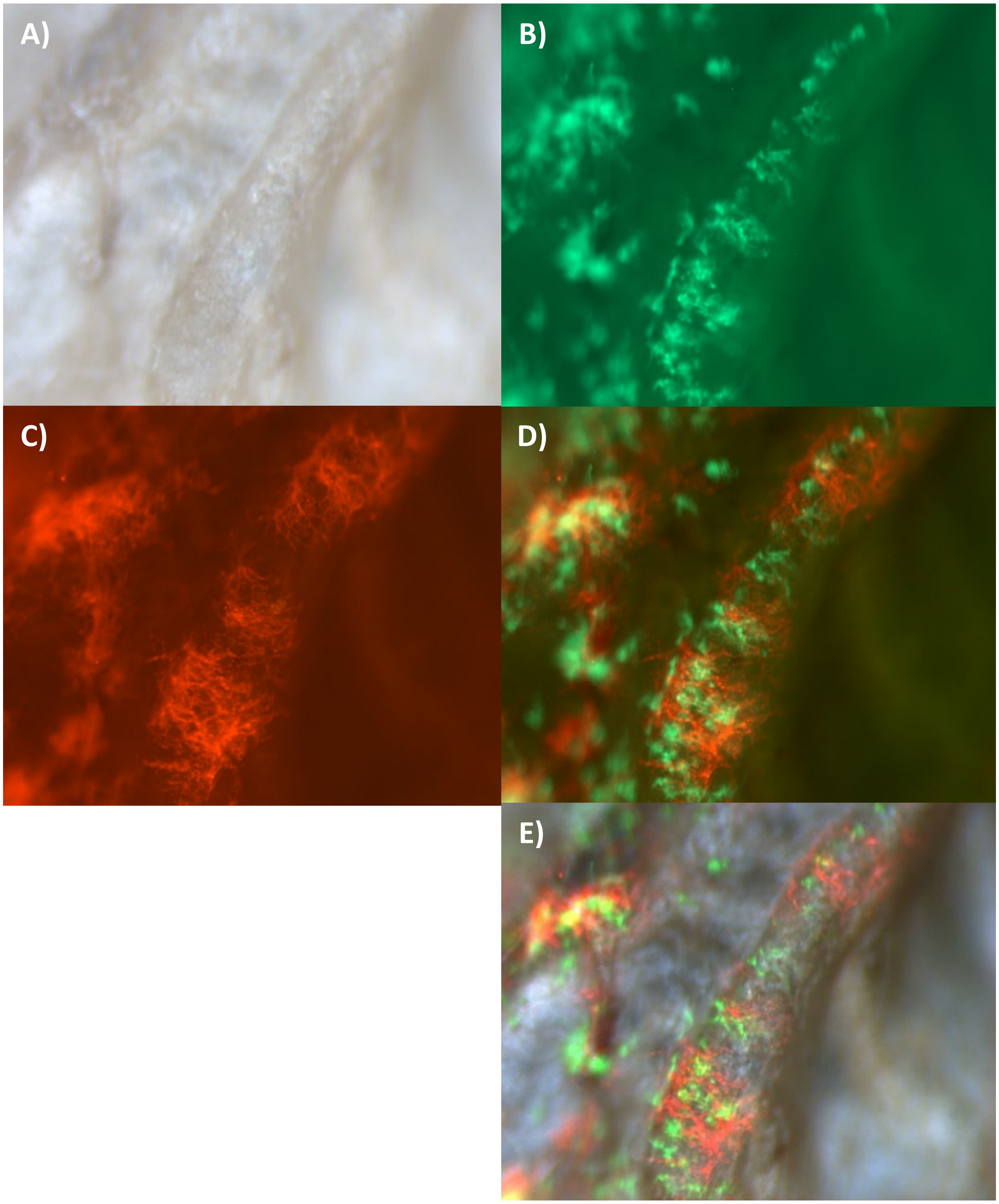

SFig 4J v) Bright field, GFP, Cherry, GFP/Cherry overlay for Fig 16 panel J

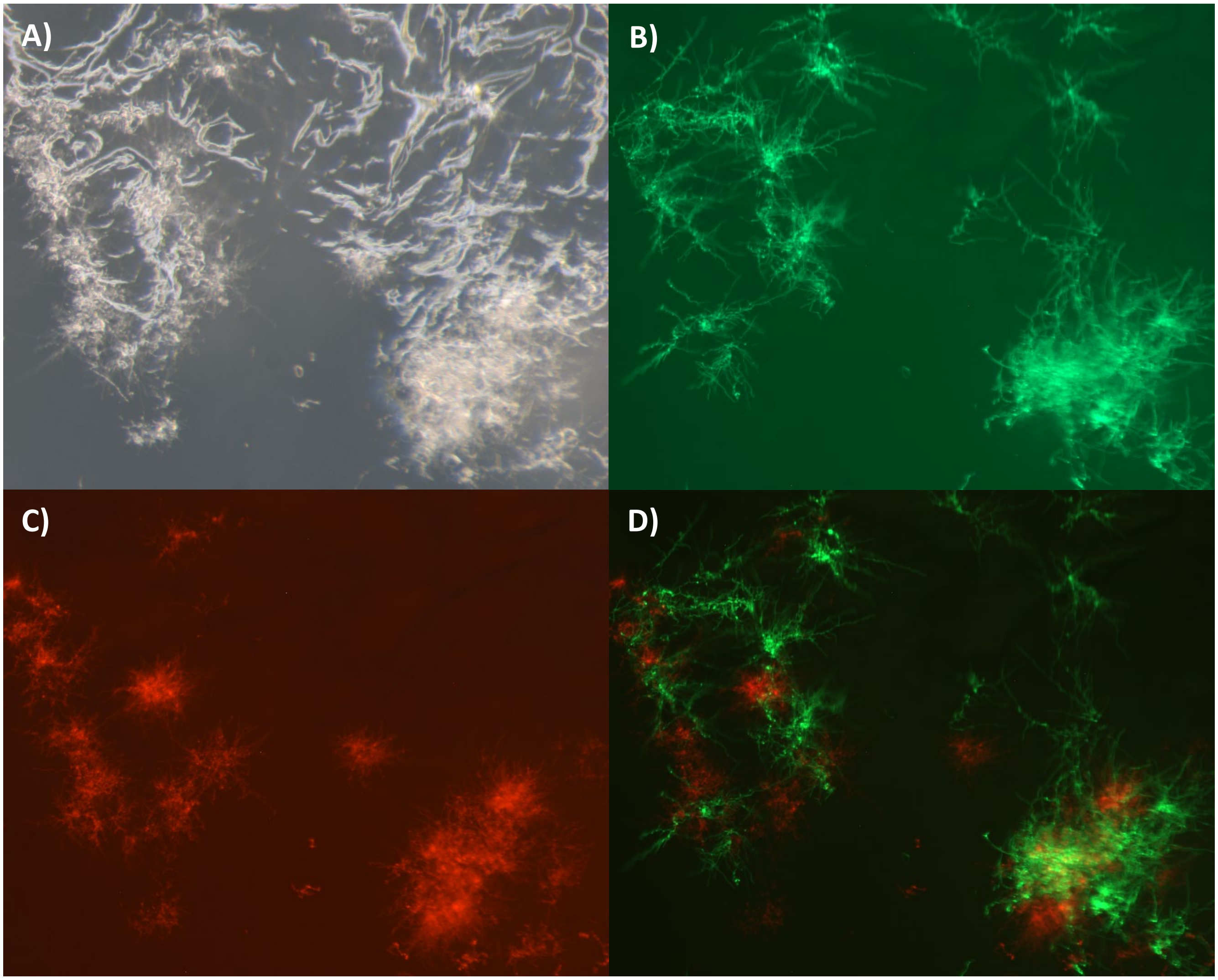

SFig 4J vi) Bright field, GFP, Cherry, GFP/Cherry overlay for Fig 16 panel L

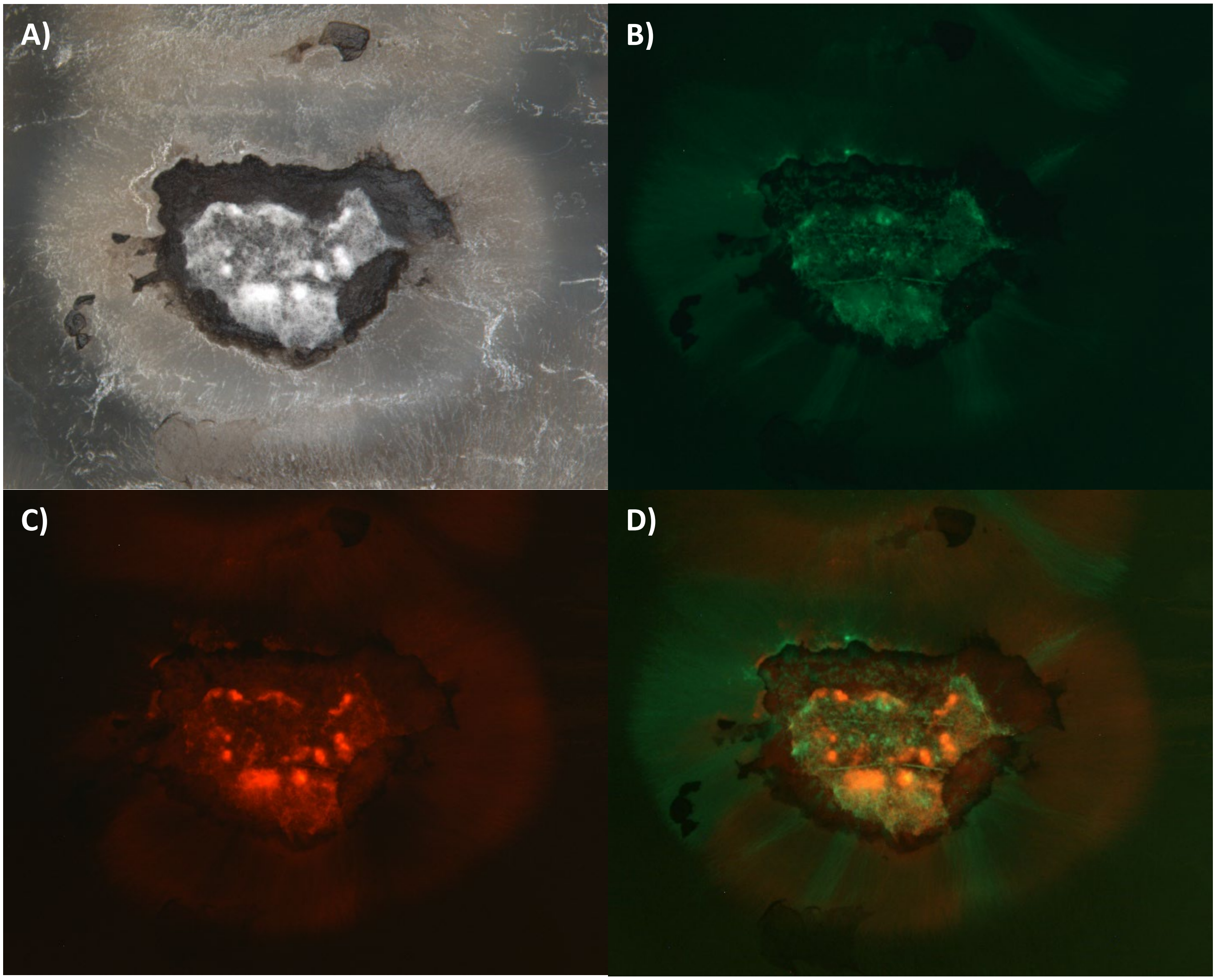

Supplement: S4 Fig — A-J The GFP and Cherry/dsRed images used to generate the overlays in manuscript figures. (PDF) [file ppat.1012639.s005.pdf]
