# Supplementary material for: Metarhizium fight club: Within-host competitive exclusion and resource partitioning
Source: PLoS Pathog. 2024 Nov 7;20(11):e1012639. doi: 10.1371/journal.ppat.1012639 (PMC11542789; doi:10.1371/journal.ppat.1012639)
Supplement: S5 Fig — A) bright field and B) green fluorescence of Ma549-GFP colony forming units (CFUs) in 2 μl of hemolymph from a larva 4-day post infection. The hemolymph of Ma549-infected insects appeared to be a monoculture of Ma549. C) bright field D) Cherry and E) bright field overlaid with Cherry image of a hemolymph sample from a larva 4 days post infection with Mr2575-Cherry showing a single Mr2575 colony surrounded by bacterial and yeast contaminants. F) Darkened surface appearance of a caterpillar with hemolymph contaminated with bacteria shortly after death (5-days post infection with both Mr2575-GFP and Mr2575-Cherry). G) GFP and H) Cherry images showing light fluorescent zone. I) bright field of zone and J) overlay of Cherry and GFP images seven days post infection showing sporulation of Mr2575-Cherry and Mr2575-GFP. (DOCX) [file ppat.1012639.s006.docx]

S5 Fig. A) bright field and B) green fluorescence of Ma549 colony forming units (CFUs) in 2 µl of hemolymph from a larva 4 days post infection. The hemolymph of Ma549-infected insects appeared to be a monoculture of Ma549. C) bright field D) cherry and E) bright field overlaid with cherry image of a hemolymph sample from a larvae 4 days post infection with Mr2575 showing a single Mr2575 colony surrounded by bacterial and yeast contaminants. F) Darkened surface appearance of a caterpillar with hemolymph contaminated with bacteria shortly after death (5-days post infection with both GFP-Mr2575 and Cherry-Mr2575). G) GFP and H) cherry images showing light fluorescent zone. I) bright field of zone and J) overlay of cherry and GFP images seven days post infection showing sporulation of Cherry-Mr2575 and GFP-Mr2575

**A)**


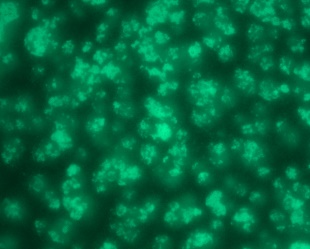

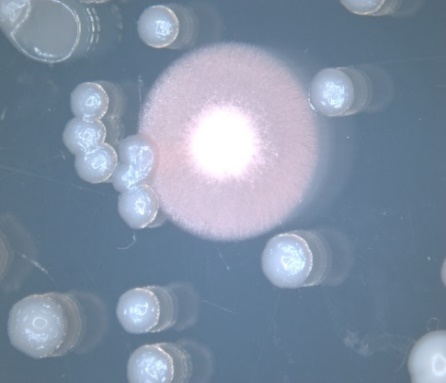

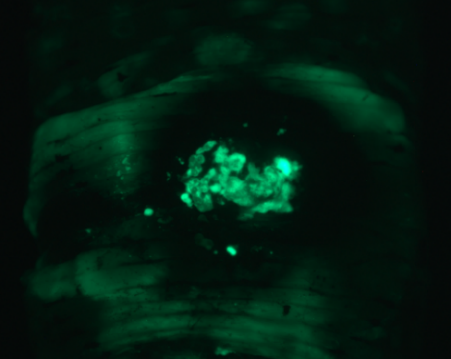

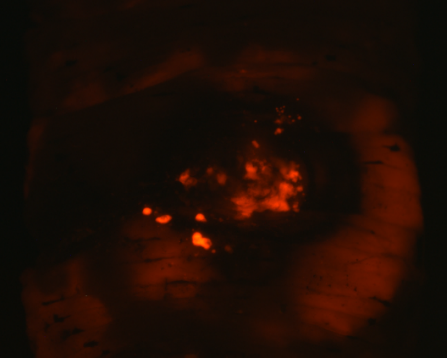

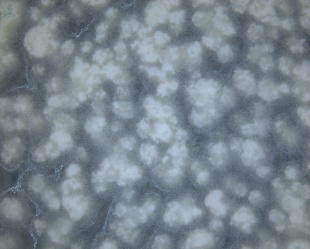

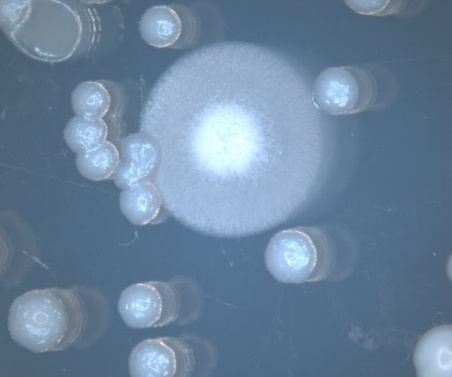

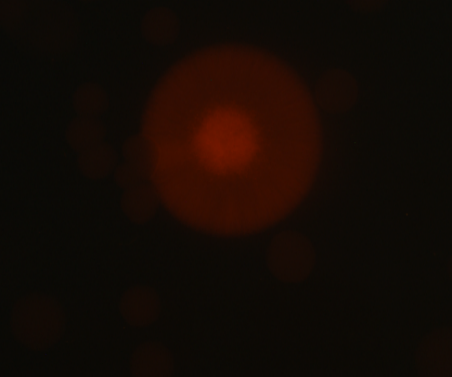

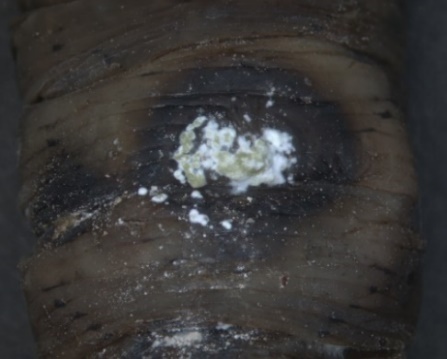

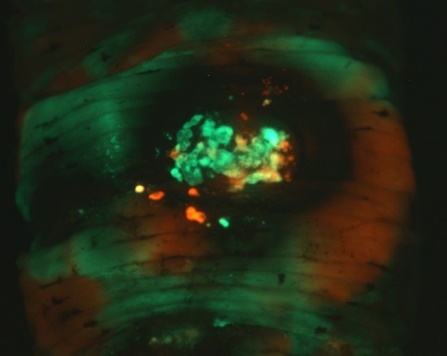

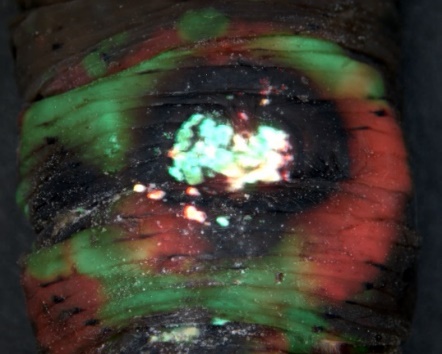


**A)**

**C)**

**E)**

**H)**

**G)**

**F)**

**D)**

**B)**

**J)**

**I)**

SFig. 2 A) GFP, Cherry image and overlay for Fig 4 panel B

**C)**

**B)**

**A)**
