# Supplementary material for: Metarhizium fight club: Within-host competitive exclusion and resource partitioning
Source: PLoS Pathog. 2024 Nov 7;20(11):e1012639. doi: 10.1371/journal.ppat.1012639 (PMC11542789; doi:10.1371/journal.ppat.1012639)
Supplement: S6 Fig — (DOCX) [file ppat.1012639.s007.docx]

S6 Fig GFP images of Ma549 budding blastopores and pseudohyphae in *M. sexta* hemolymph four days after topical infection with Ma549-GFP

**)**

**A)**


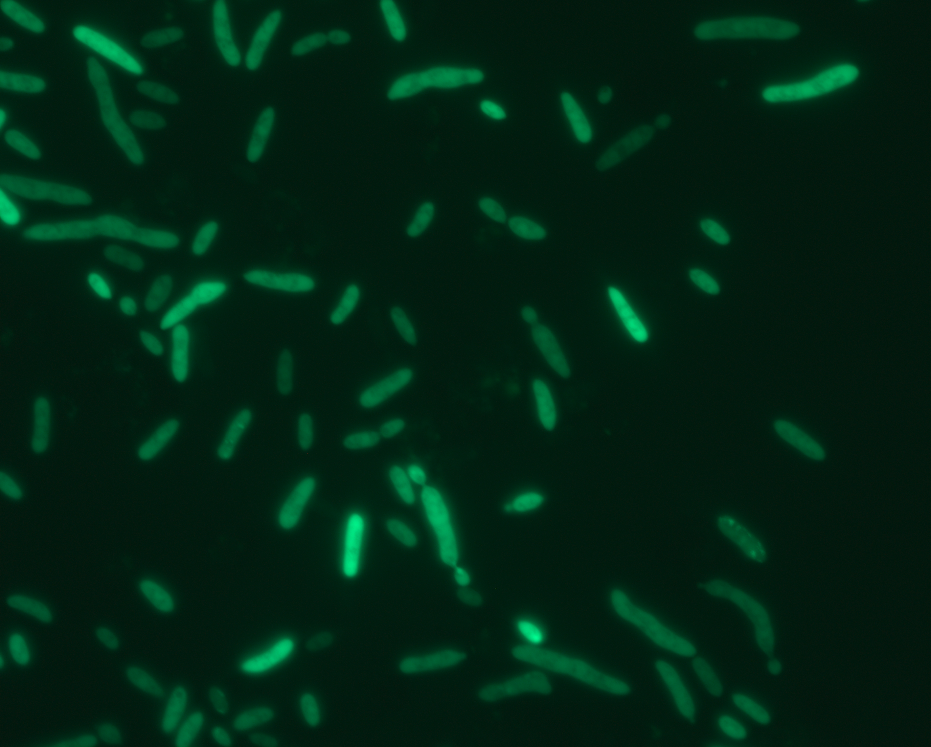

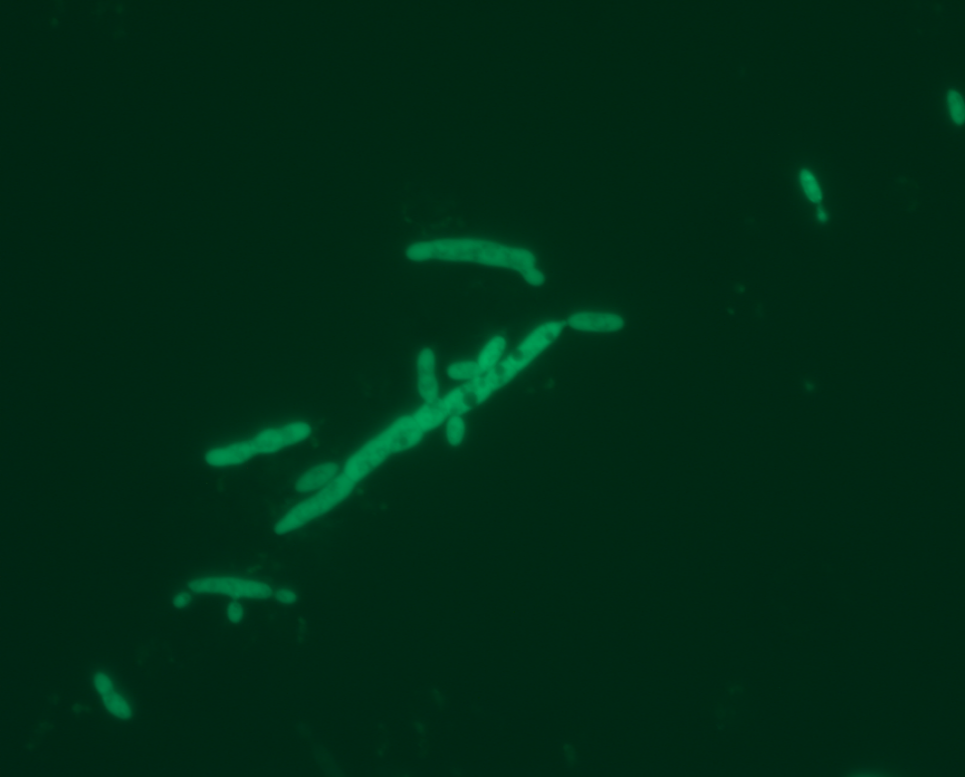

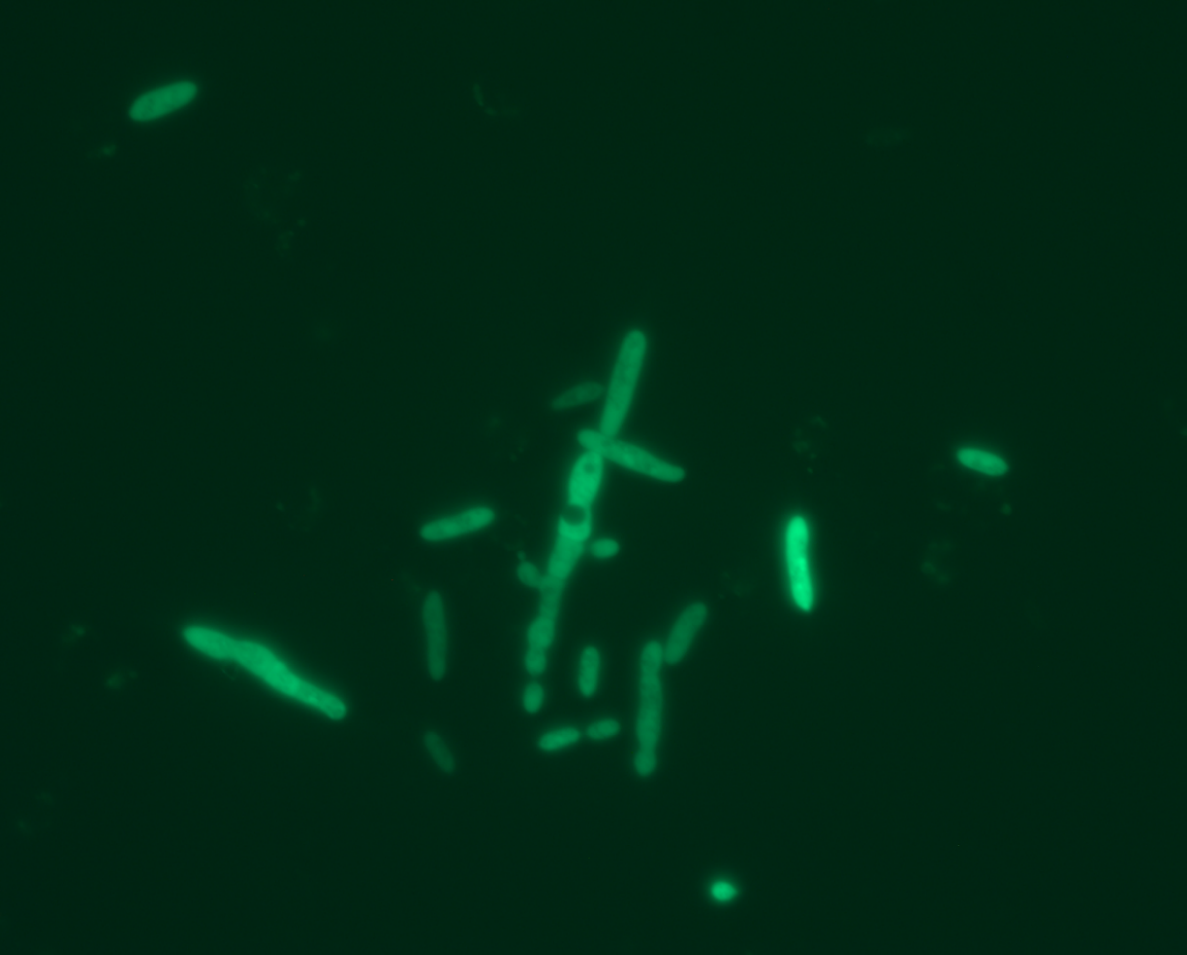


**A)**

**B)**

**C)**
