# Supplementary material for: Metarhizium fight club: Within-host competitive exclusion and resource partitioning
Source: PLoS Pathog. 2024 Nov 7;20(11):e1012639. doi: 10.1371/journal.ppat.1012639 (PMC11542789; doi:10.1371/journal.ppat.1012639)
Supplement: S7 Fig — B) surface of the same cadaver showing preferential emergence of Ma549 hyphae through hair sockets. C) bright field, D) GFP, E) Cherry, F) GFP/Cherry overlay showing adjacent Ma549-GFP and Mr2575-Cherry colonies on a M. sexta cadaver approximately 12 hrs postmortem (DOCX) [file ppat.1012639.s008.docx]

S7 Fig) Bright field image of cadaver section ~6 hrs post-mortem showing tufts of Ma549 hyphae emerging through the cuticle. B) surface of the same cadaver showing preferential emergence of Ma549 hyphae through hair sockets. C) bright field, D) GFP, E) cherry, F) GFP/Cherry overlay showing adjacent Ma549-GFP and Mr2575-Cherry colonies on a *M. sexta* cadaver ~12 hrs post mortem.


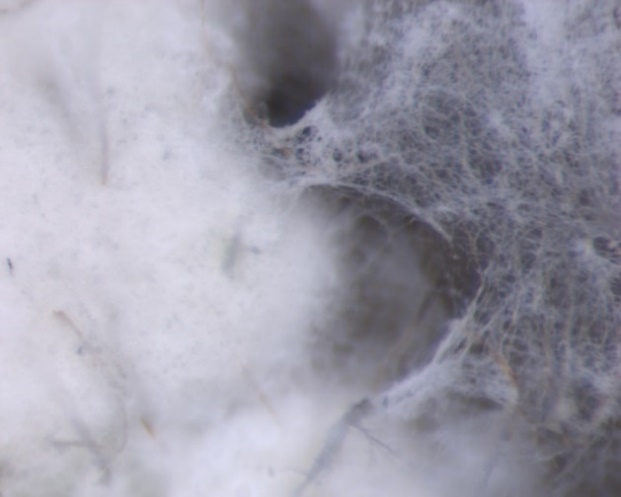

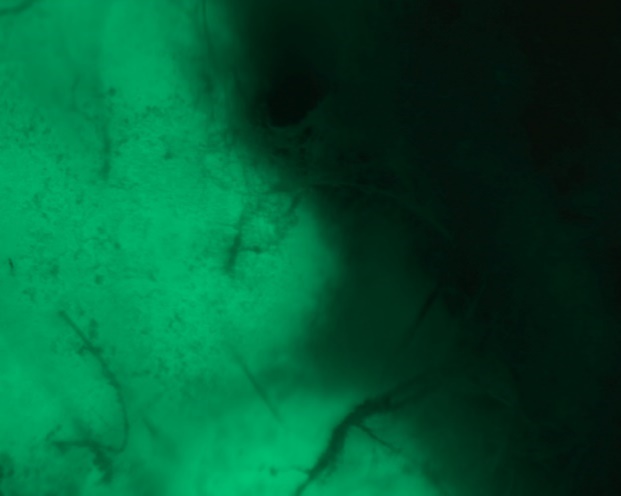

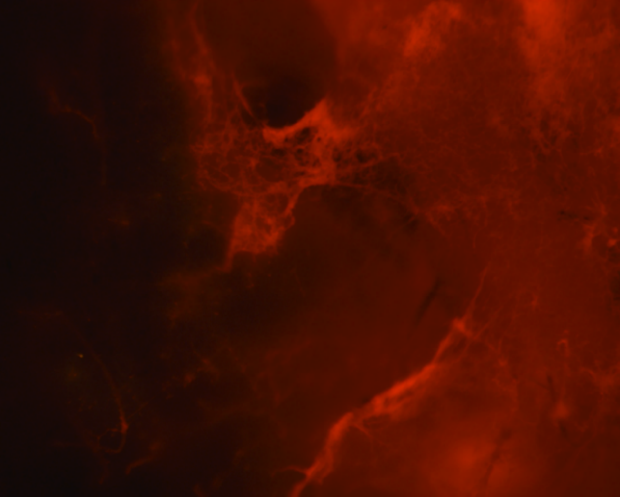

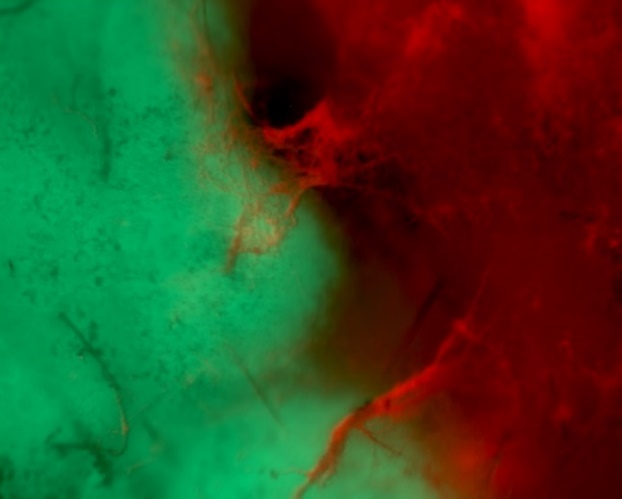

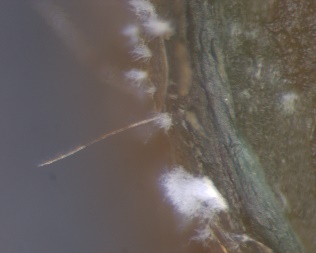

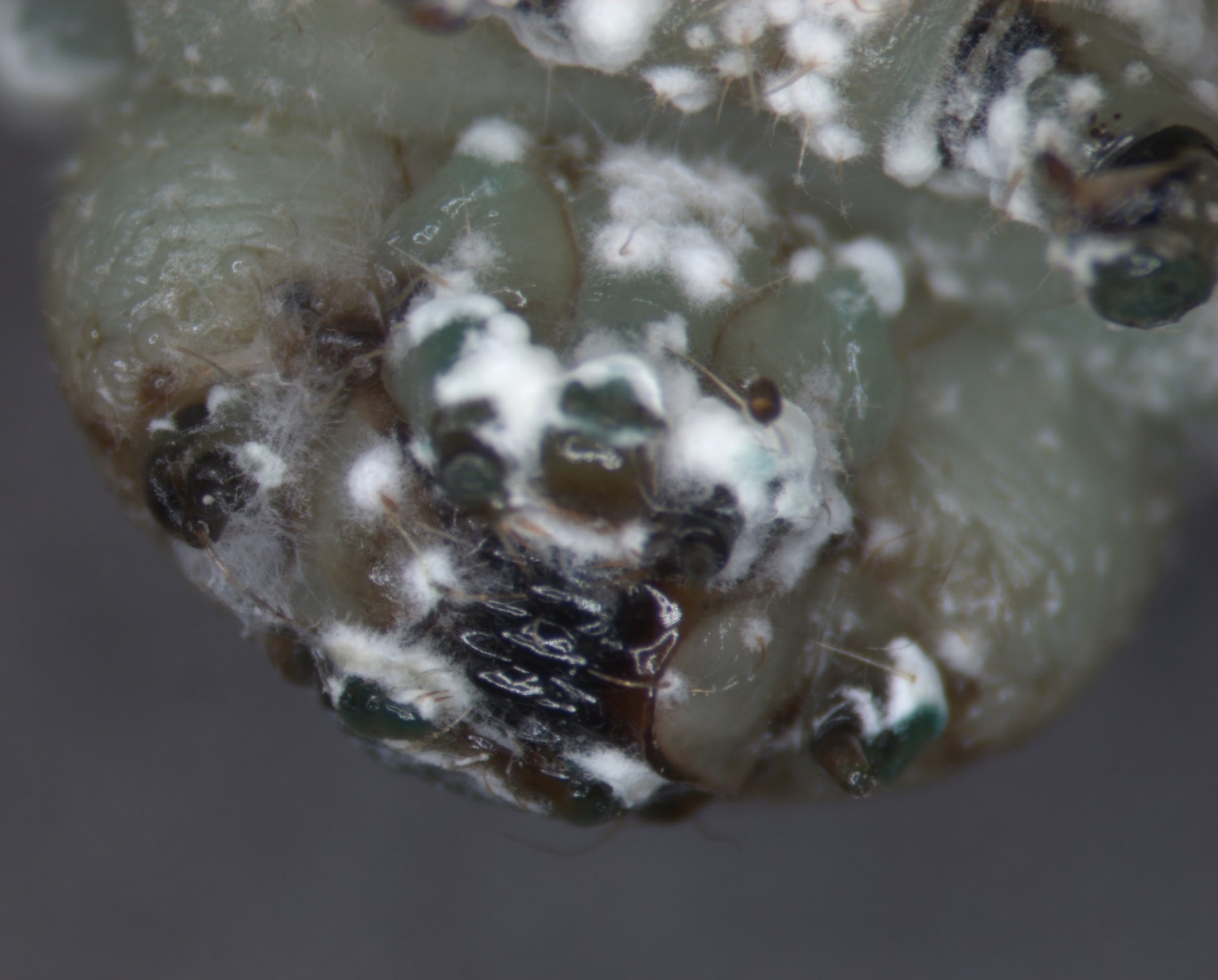


**A)**

**B)**

**C)**

**D)**

**E)**

**F)**

**A)**
