# Supplementary material for: Metarhizium fight club: Within-host competitive exclusion and resource partitioning
Source: PLoS Pathog. 2024 Nov 7;20(11):e1012639. doi: 10.1371/journal.ppat.1012639 (PMC11542789; doi:10.1371/journal.ppat.1012639)

S8 Fig. Pictures show recent (24 hrs) cadavers of first instar *M. sexta* infected by Ma549-GFP+Mr2575-Cherry. Bright field, GFP, cherry and overlay showing roughly equal distribution of Mr2575 and Ma549 over cadavers.

A)

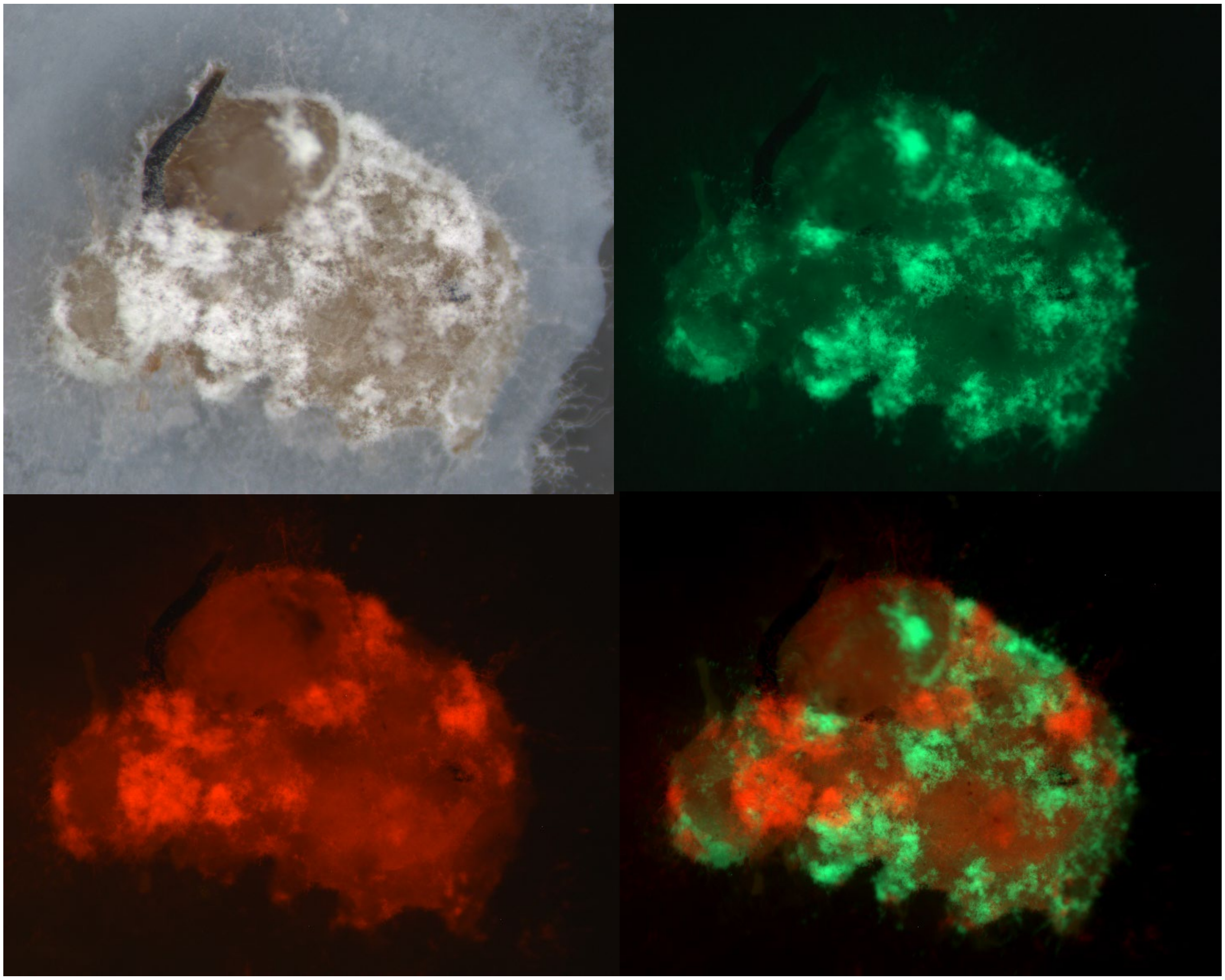

B)

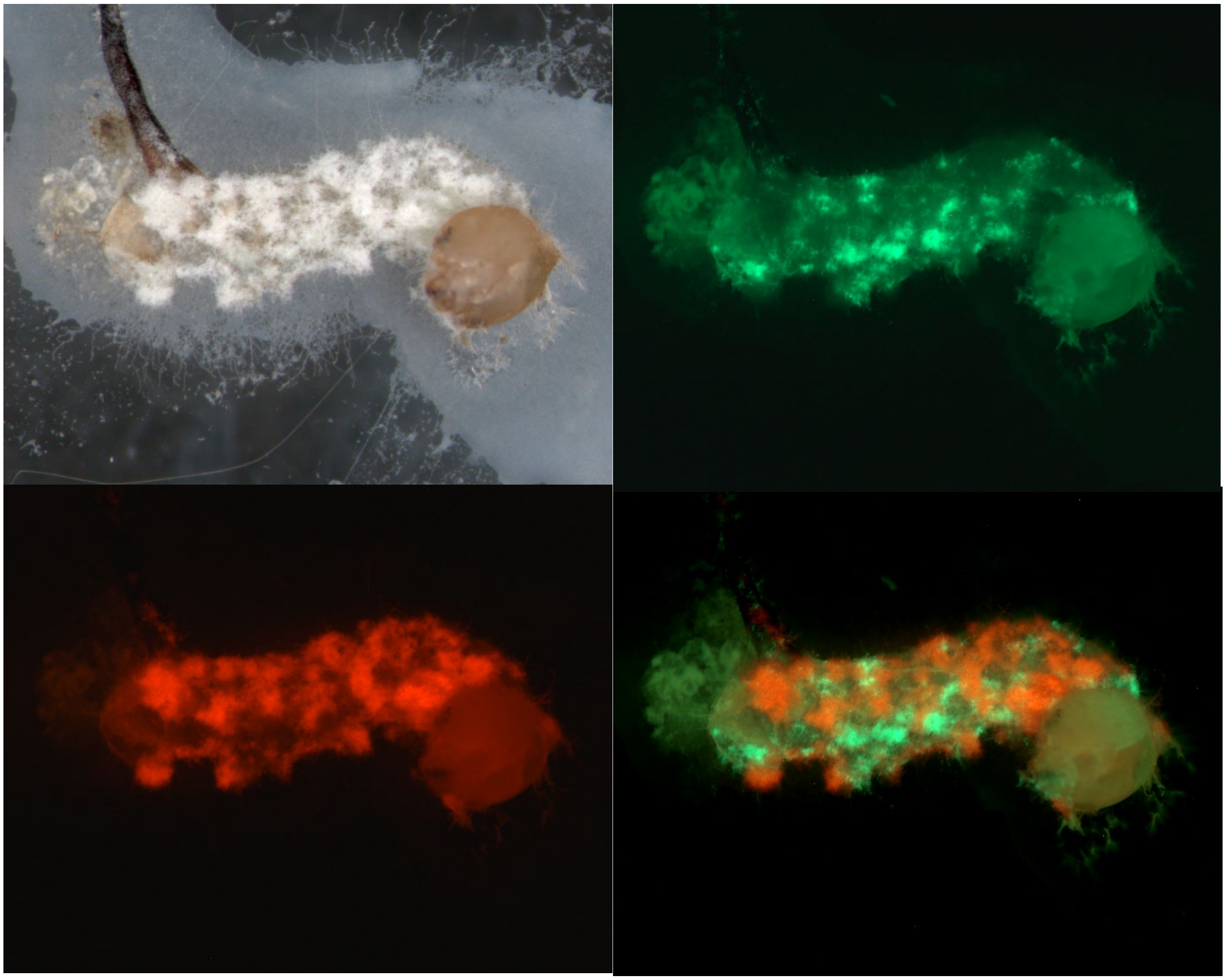

C) Higher magnification of cadaver shown in B)

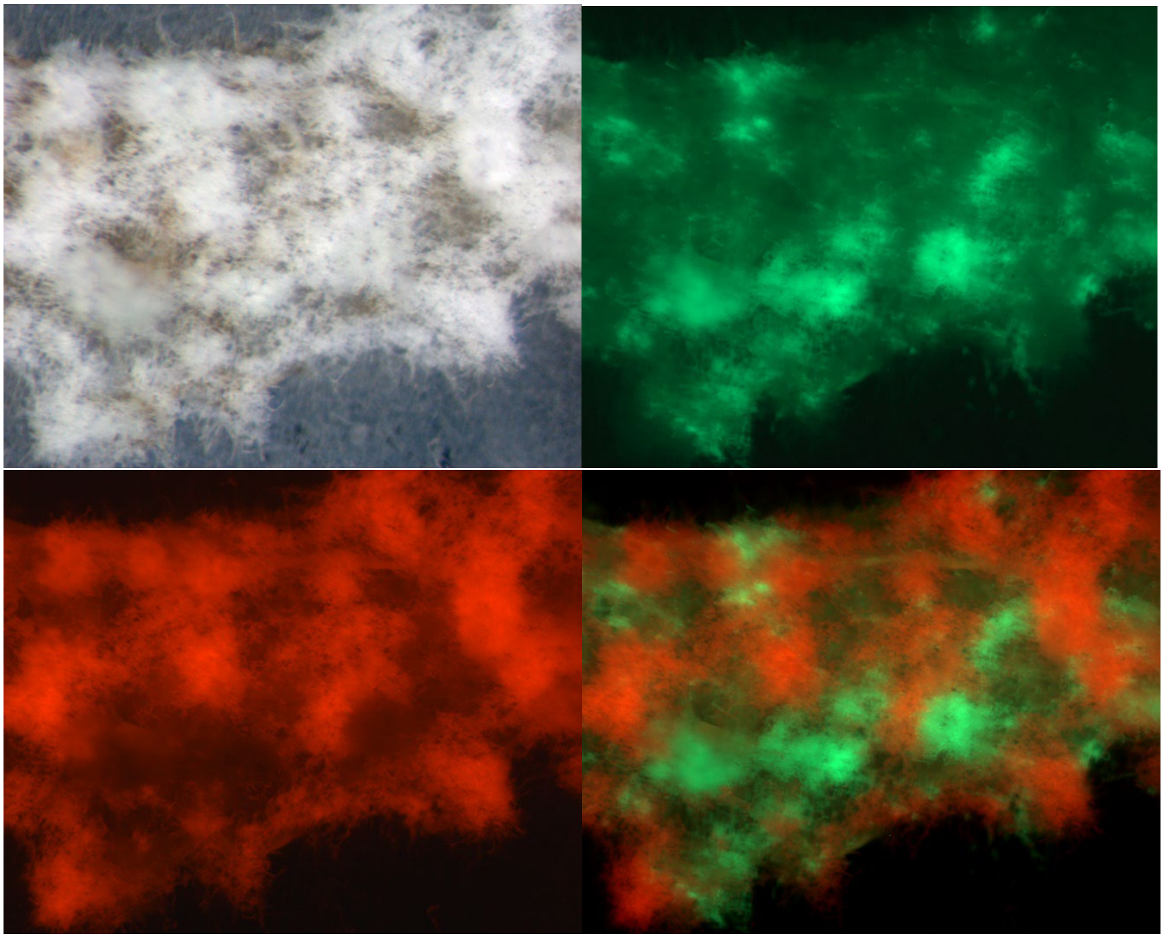

D)

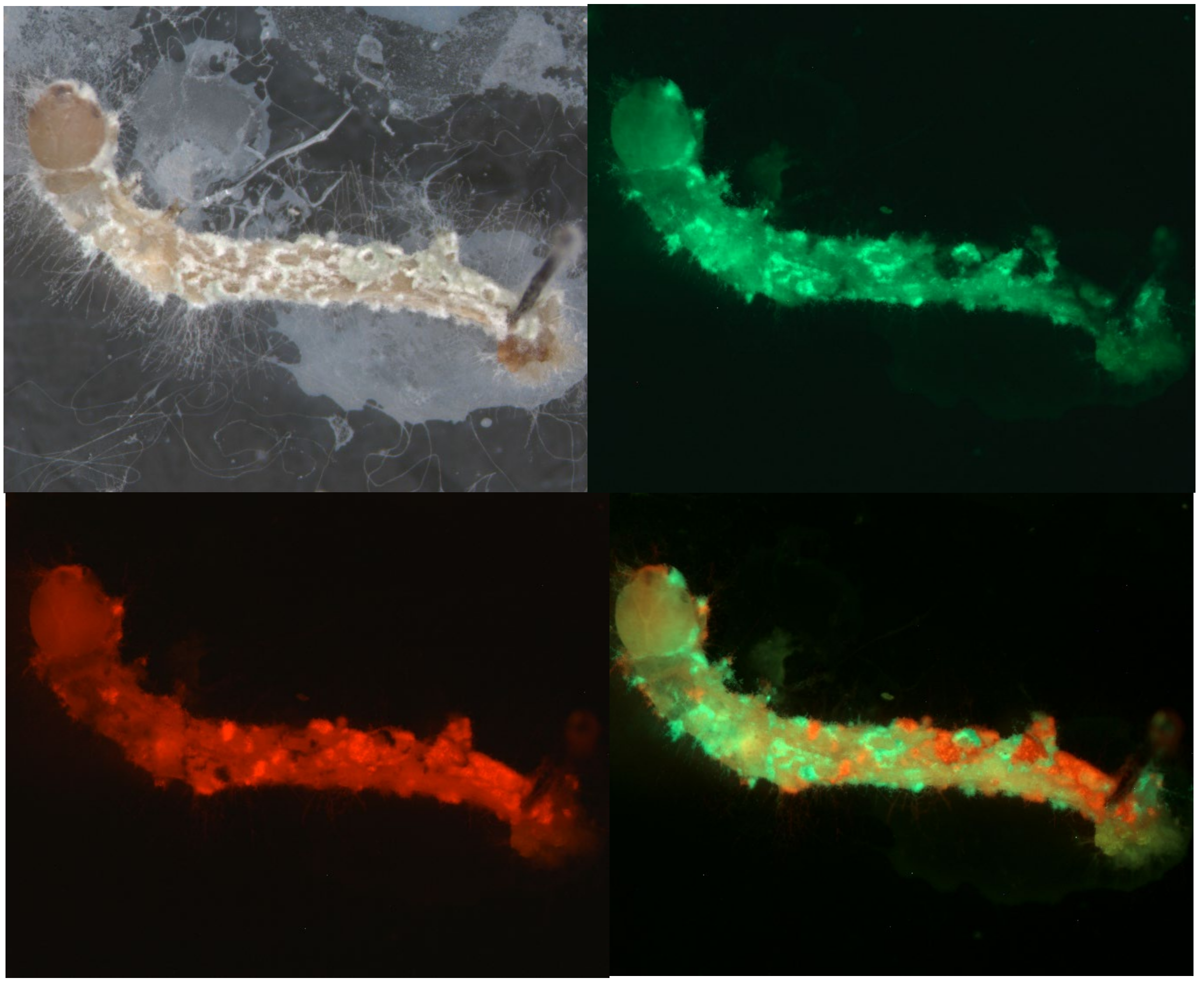

E) Higher magnification of cadaver shown in D) showing sporulation of Ma549 and Mr2575

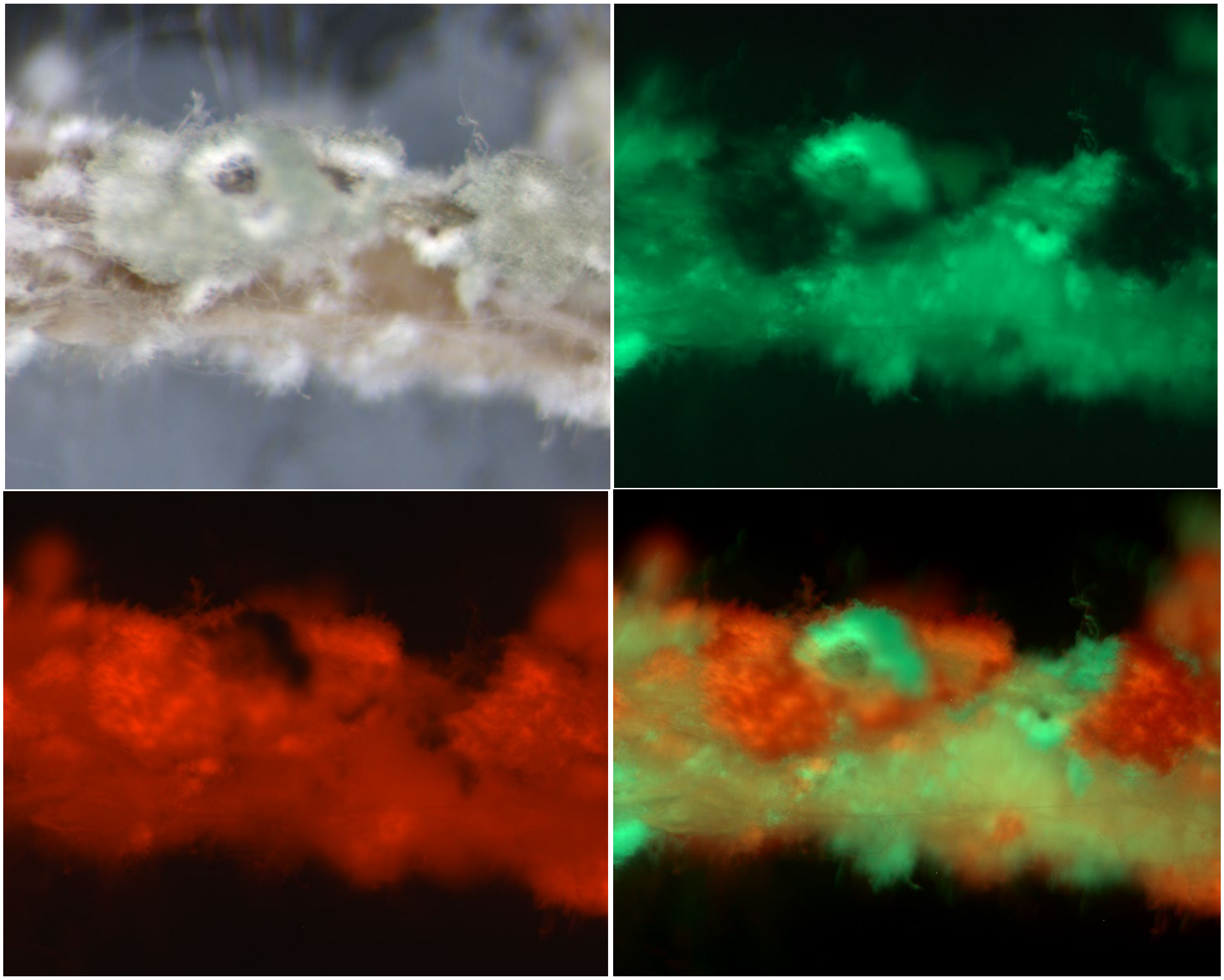

F)

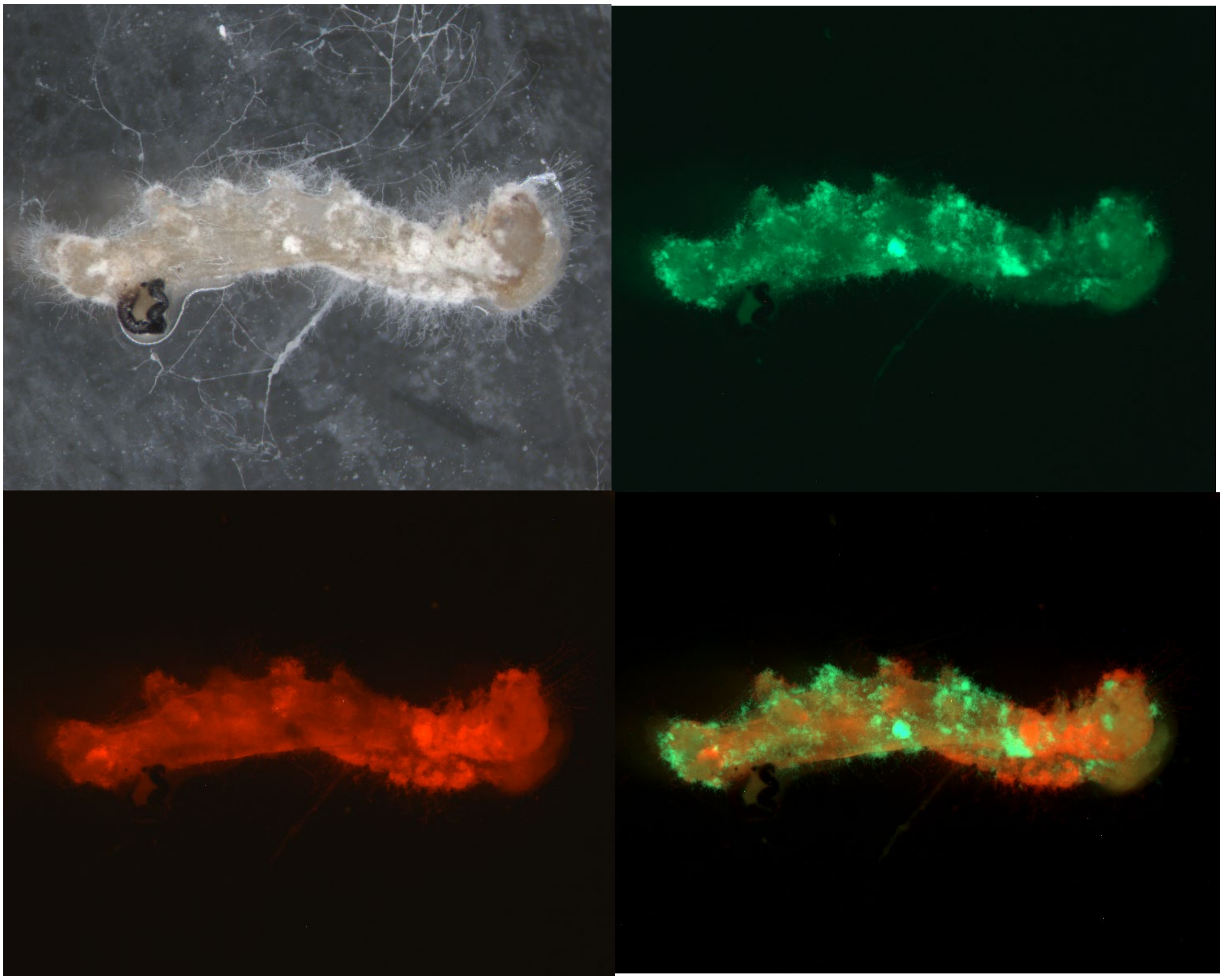

Supplement: S8 Fig — Bright field, GFP, Cherry and overlay showing roughly equal distribution of Mr2575 and Ma549 over cadavers. (PDF) [file ppat.1012639.s009.pdf]
