# Supplementary material for: Metarhizium fight club: Within-host competitive exclusion and resource partitioning
Source: PLoS Pathog. 2024 Nov 7;20(11):e1012639. doi: 10.1371/journal.ppat.1012639 (PMC11542789; doi:10.1371/journal.ppat.1012639)
Supplement: S9 Fig — This figure supplements Fig 9. (DOCX) [file ppat.1012639.s010.docx]

S9 Fig. Bright field and cherry images of Mr2575 producing round clumps of non-sporulating aerial hyphae on mature cadavers (following sporulation). This figure supplements Fig 9


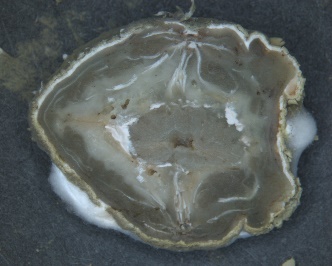

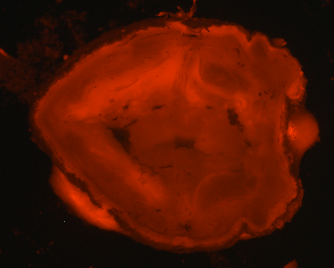


**A)**

**B)**

S9 Infection of fifth instar *M. sexta* larvae by simultaneous topical application of cherry labelled Mr2575 and GFP-labelled Ma549 showing Mr2575 producing a round clump of non-sporulating aerial hyphae on mature cadavers (following sporulation). This figure supplements Fig 9.


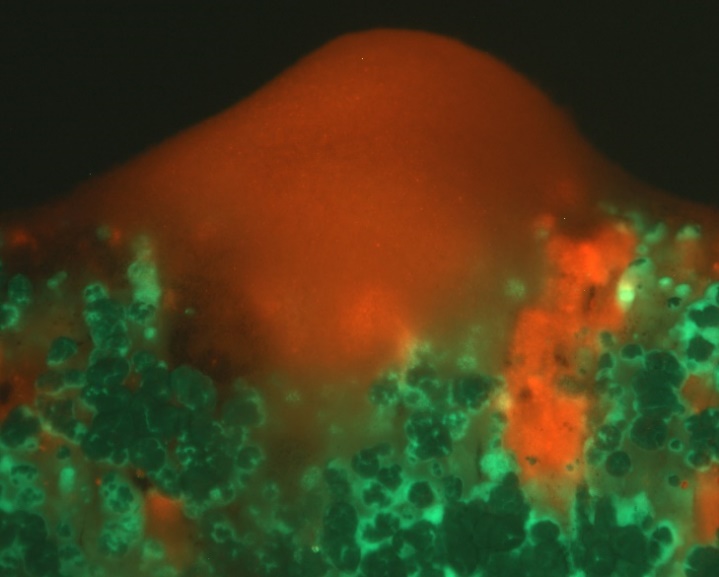

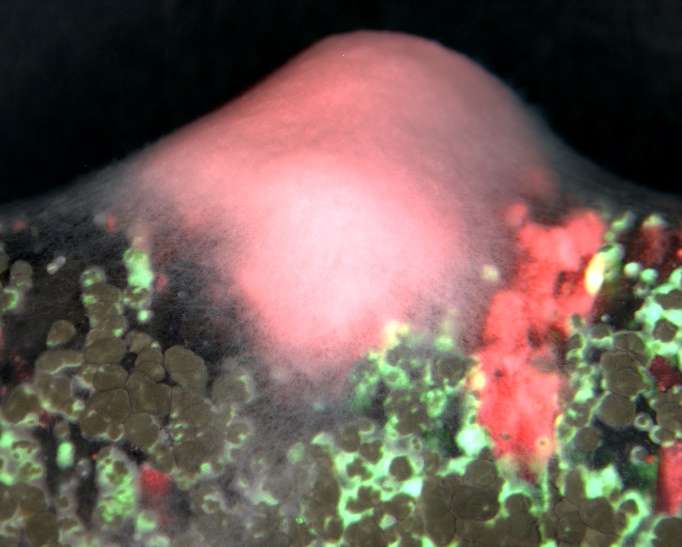

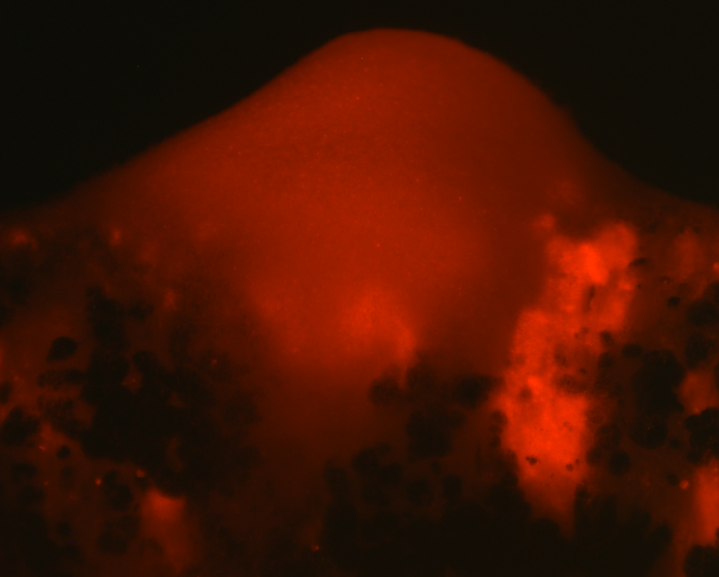

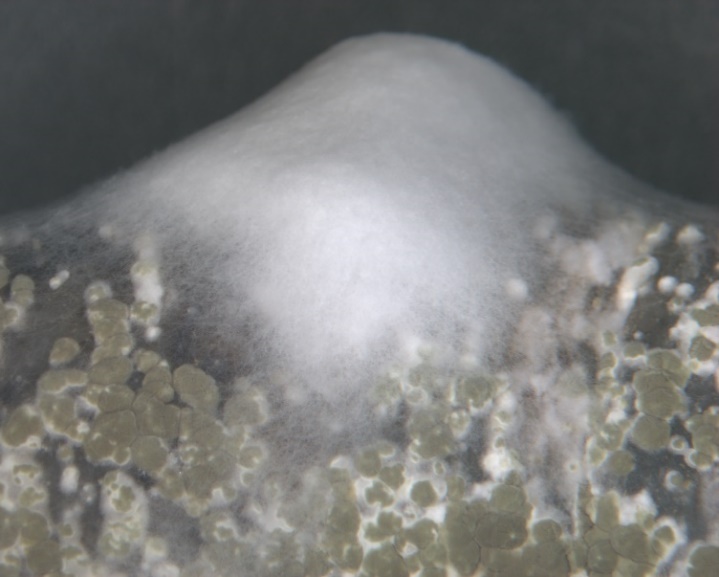

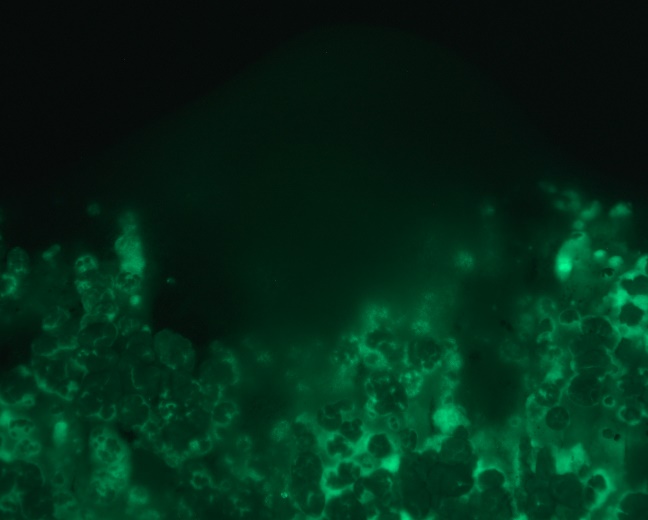


**A)**

**B)**

**C)**

**D)**

**E)**
