# Supplementary material for: Metarhizium fight club: Within-host competitive exclusion and resource partitioning
Source: PLoS Pathog. 2024 Nov 7;20(11):e1012639. doi: 10.1371/journal.ppat.1012639 (PMC11542789; doi:10.1371/journal.ppat.1012639)
Supplement: S10 Fig — These images are additional examples to Fig 11. (DOCX) [file ppat.1012639.s011.docx]

**Supplementary figures and their captions for Fig 11**

Bright field, GFP, Cherry and overlay for Fig 11 panel B


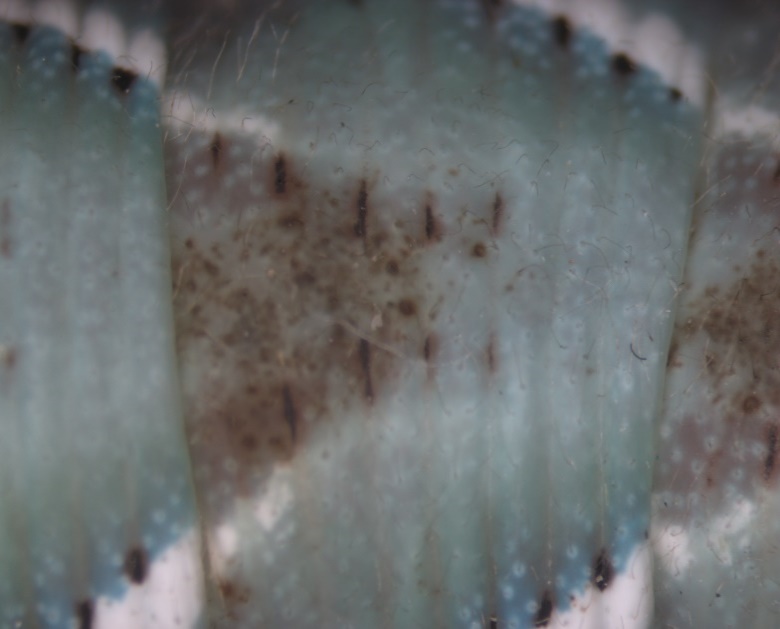

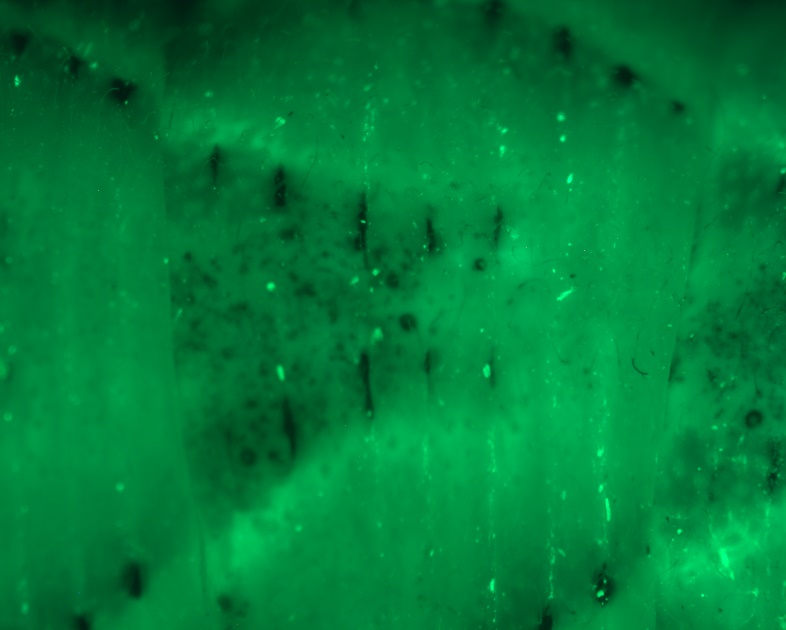

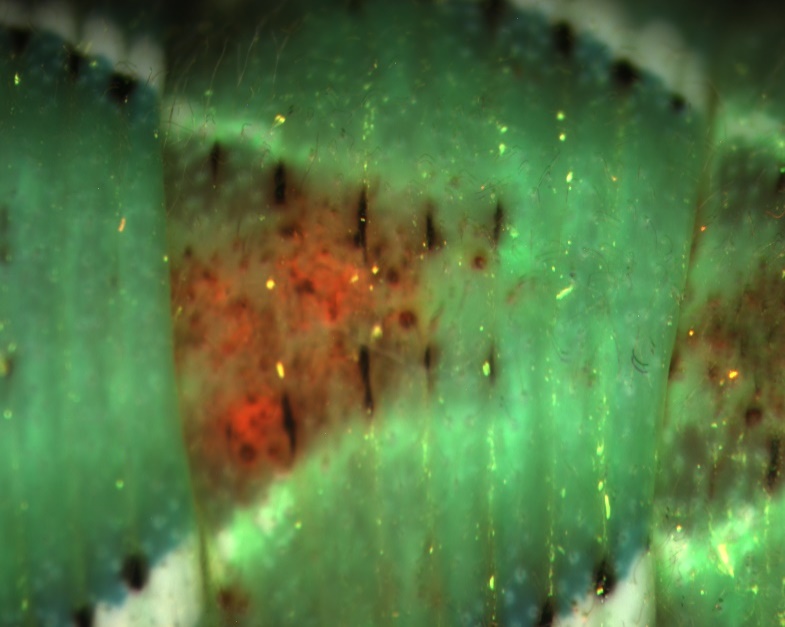

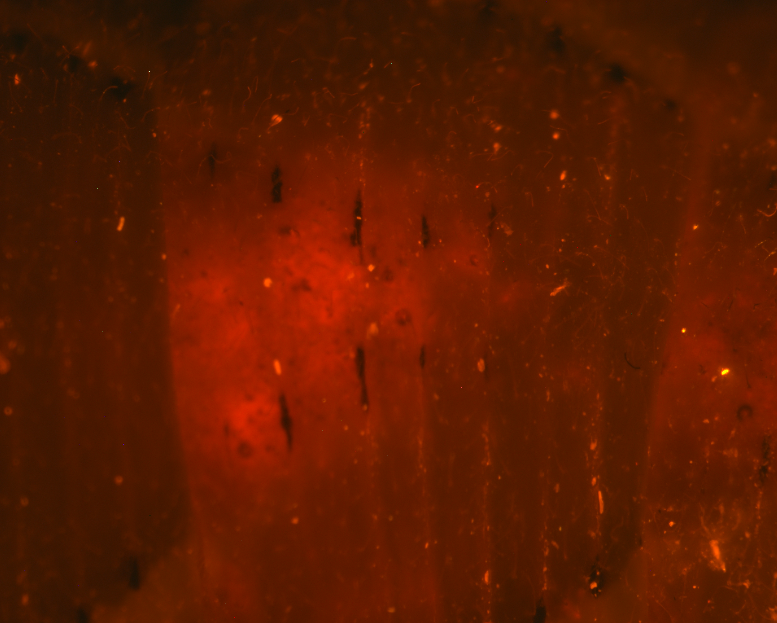


**A)**

**C)**

**D)**

**B)**

Bright field, GFP, Cherry and overlay for Fig 11 panel D


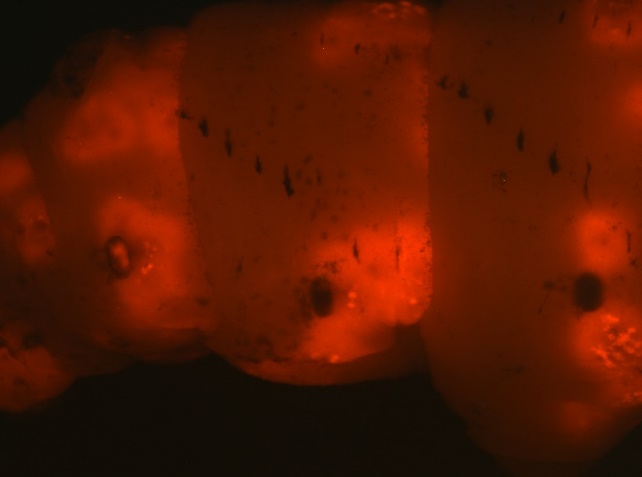

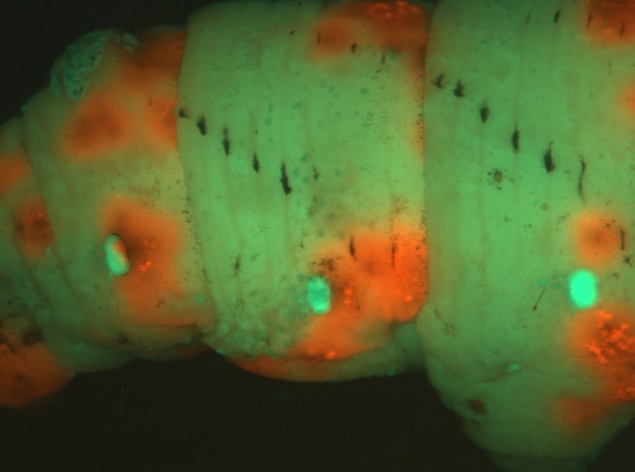

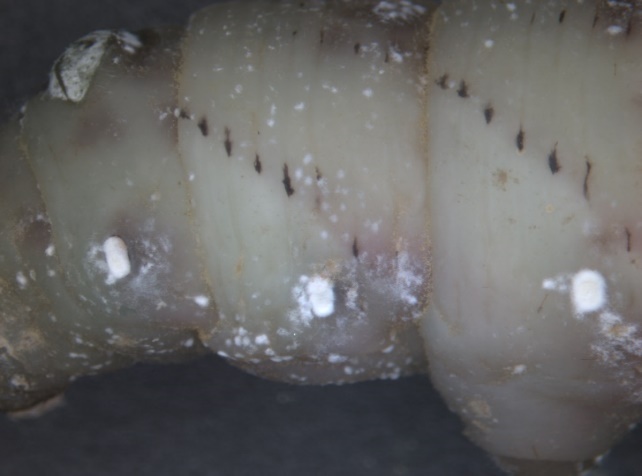

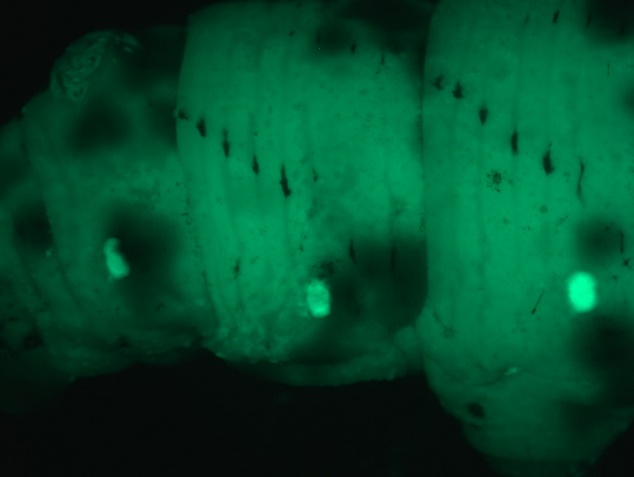

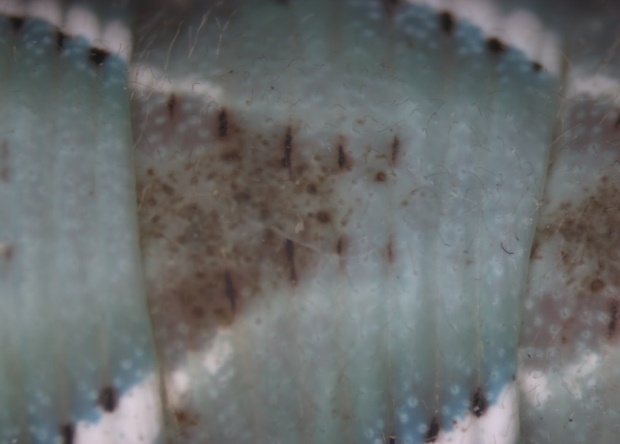

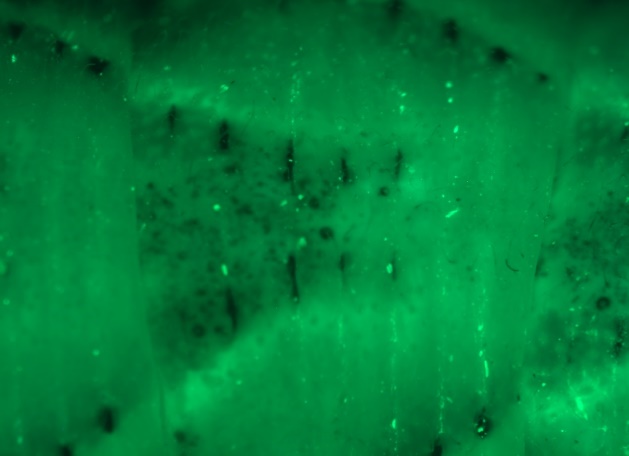

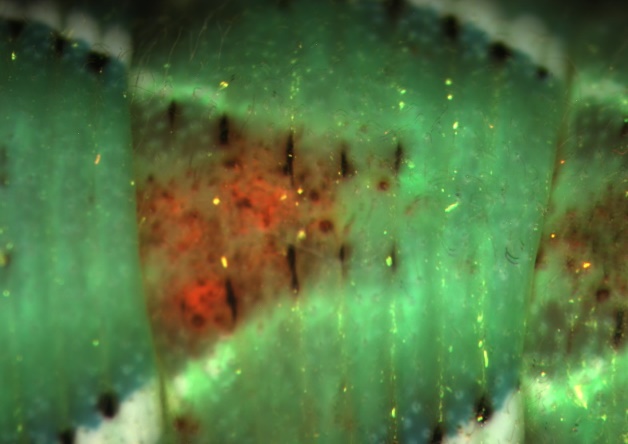

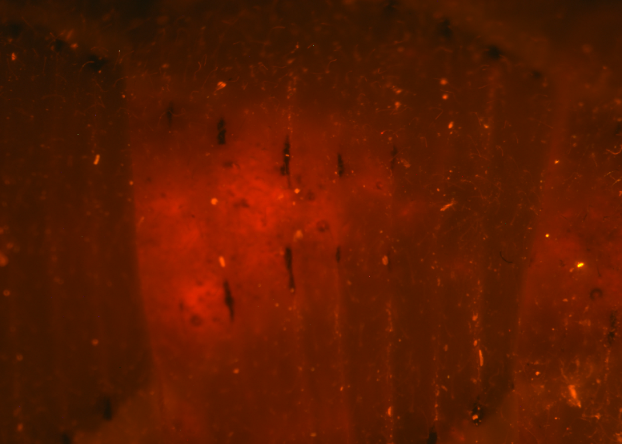


**A)**

**B)**

**C)**

**D)**

**E)**

**G)**

**H)**

**F)**

Bright field, GFP, Cherry and overlay for Fig 11 panel F


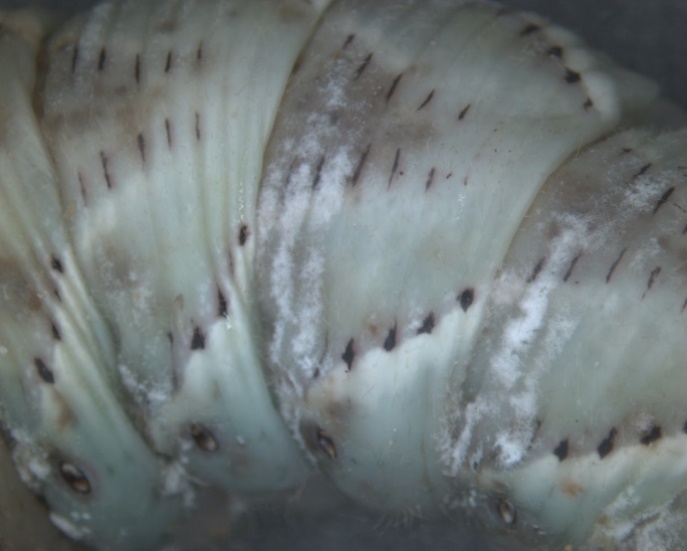

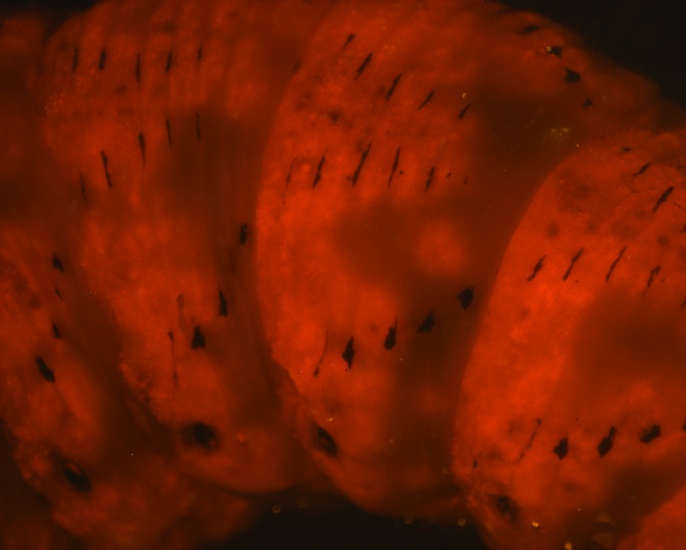

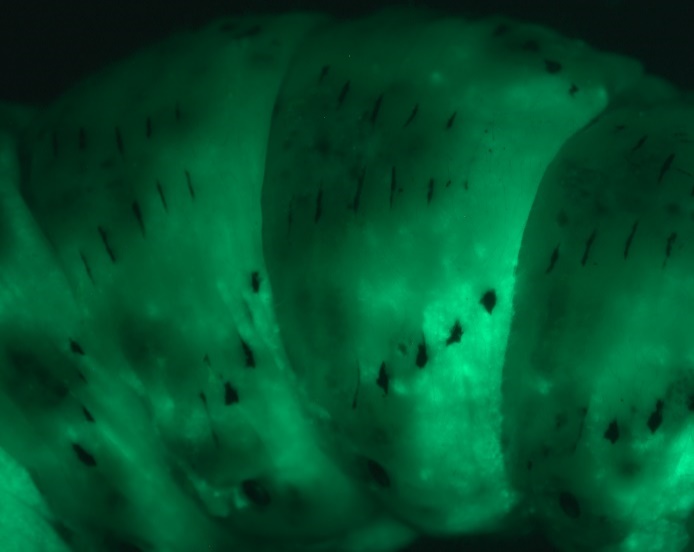

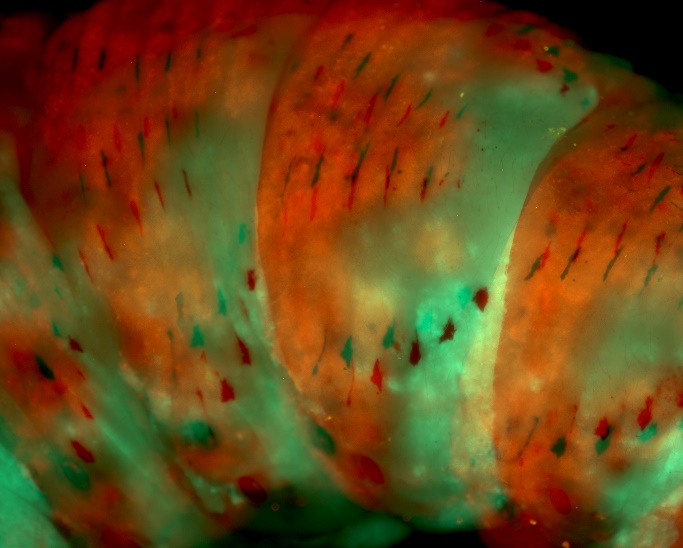


**A)**

**B)**

**C)**

**D)**

**The following are images of additional caterpillars showing segregation of Mr2575 and Ma549.**

Pictures show a recent (~8 hrs) cadaver of *Manduca* infected by Ma549-GFP+Mr2575-Cherry. Bright field, GFP, cherry and overlay showing localization of Mr2575 to melanized patches within the dorsal spots

Pictures show recent (< 18 hrs) cadavers of *Manduca* infected by Ma549-GFP+Mr2575-Cherry. Bright field, GFP, cherry and overlay showing localization of Mr2575 to melanized patches near the front of segments


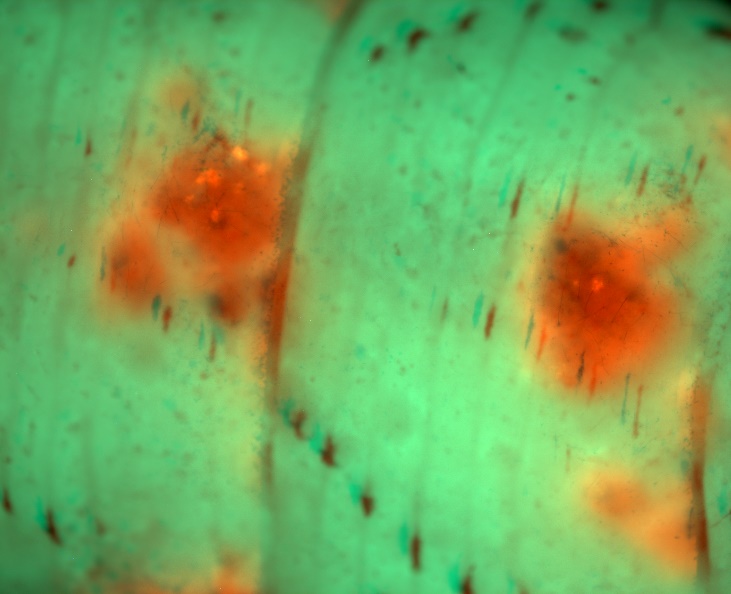

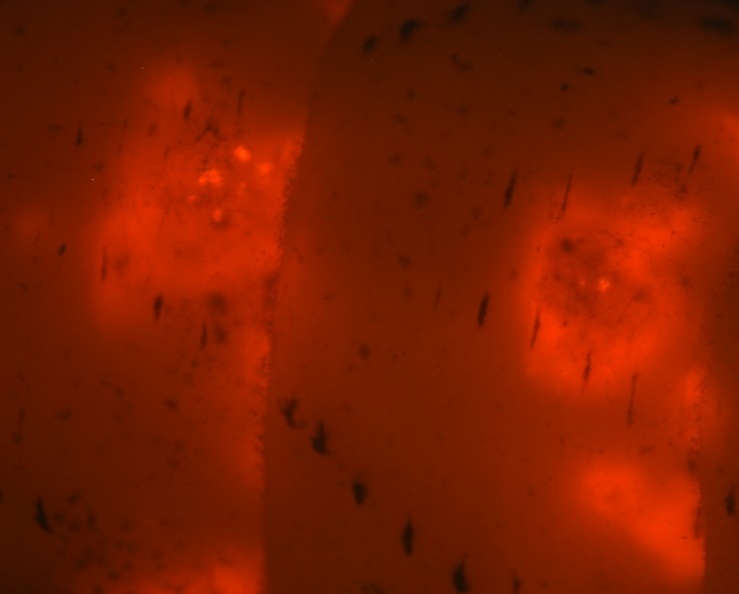

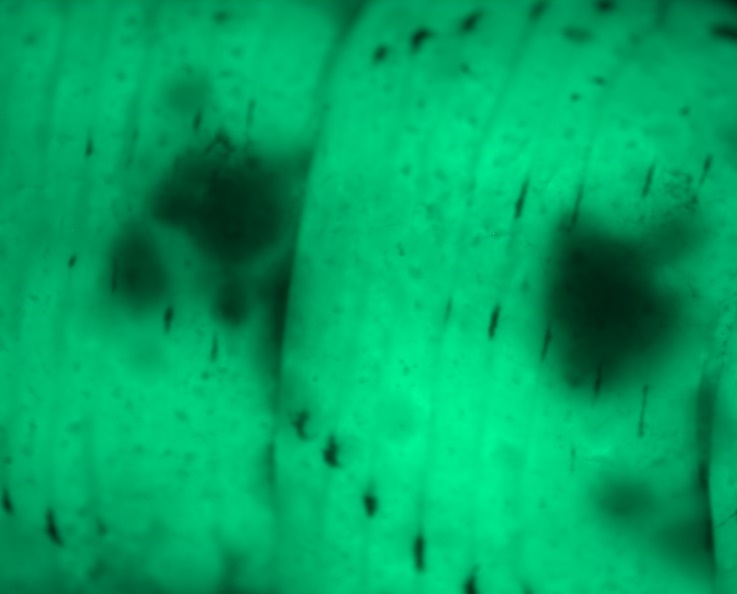

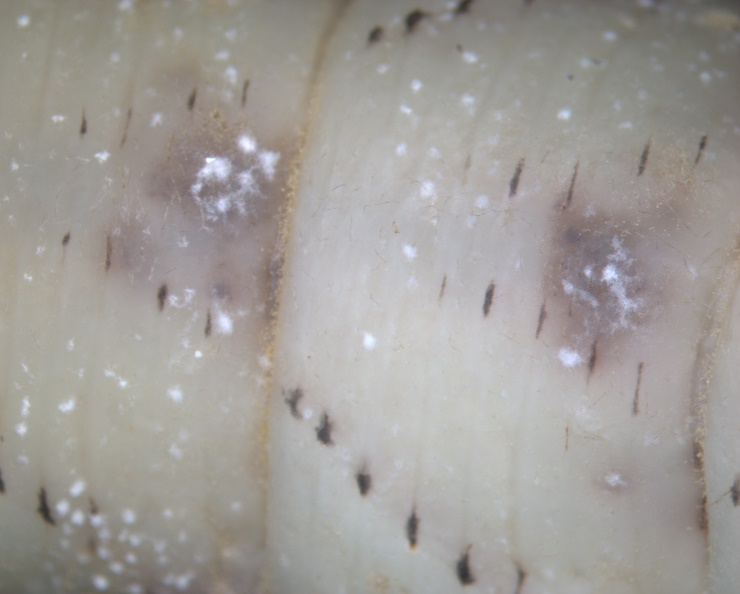


**A)**

**B)**

**C)**

**D)**


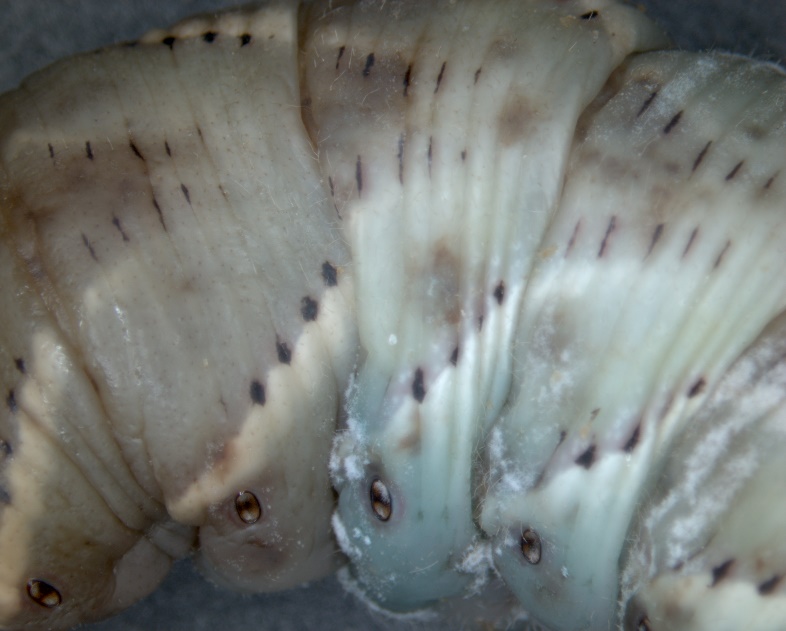

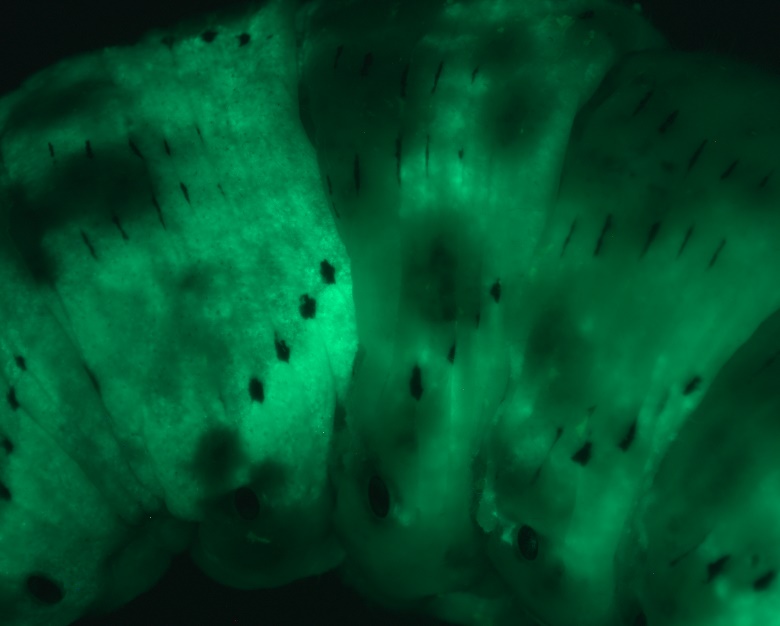

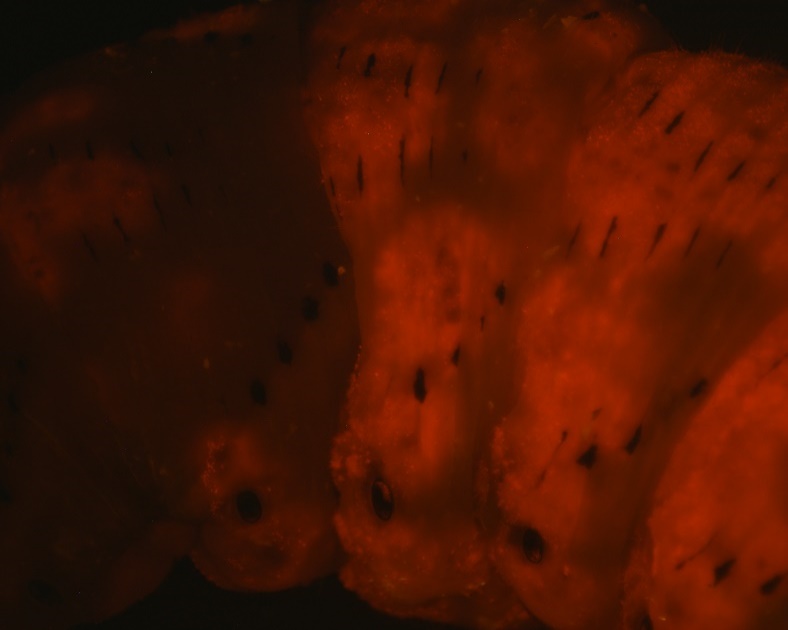

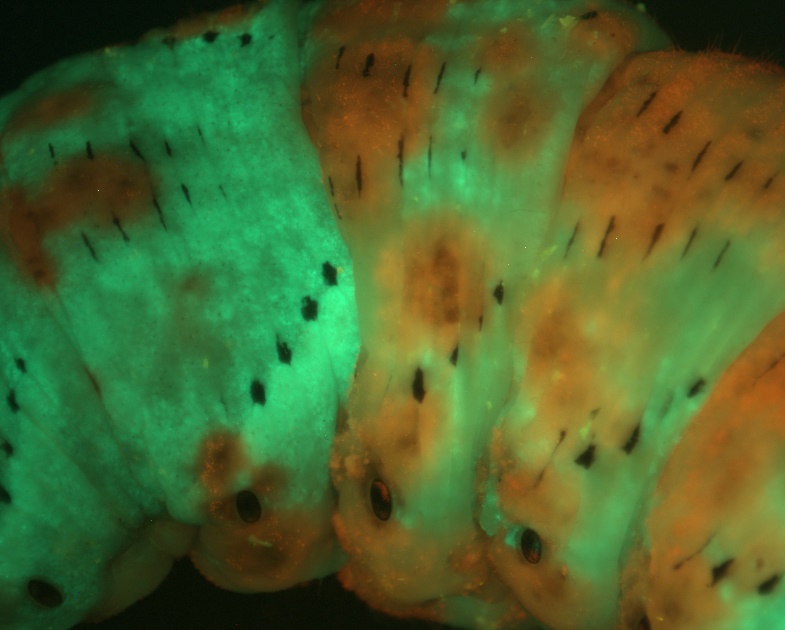


**A)**

**B)**

**C)**

**D)**

Pictures show a recent (< 18 hrs) cadaver of *Manduca* infected by Ma549-GFP+Mr2575-Cherry. Bright field, GFP, cherry and overlay showing localization of Mr2575 to melanized patches near the front of segments


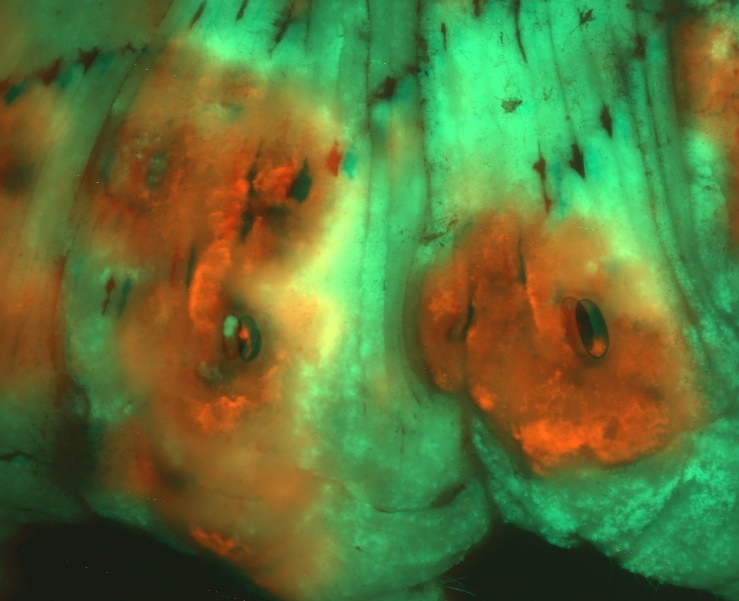

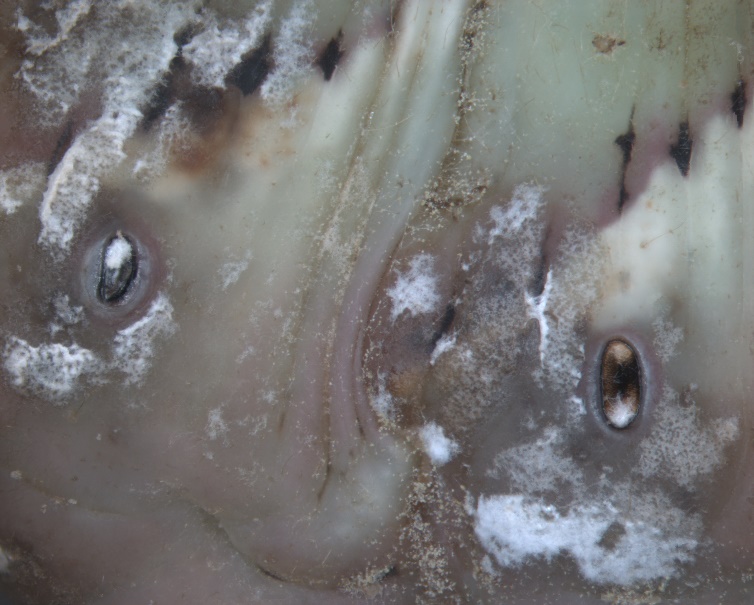

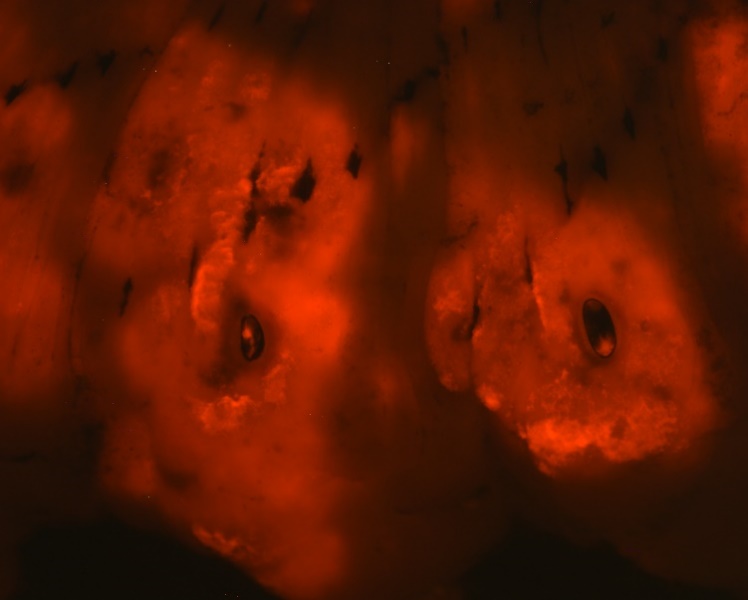

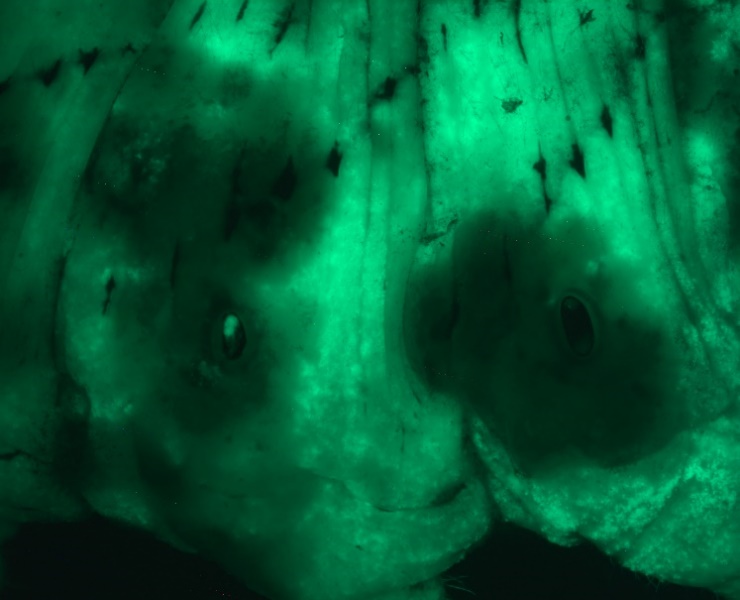


**A)**

**D)**

**C)**

**B)**

Pictures show a recent (< 18 hrs) cadaver of *Manduca* infected by Ma549-GFP+Mr2575-Cherry. Bright field, GFP, cherry and overlay showing localization of Mr2575 to melanized patches near the front of segments


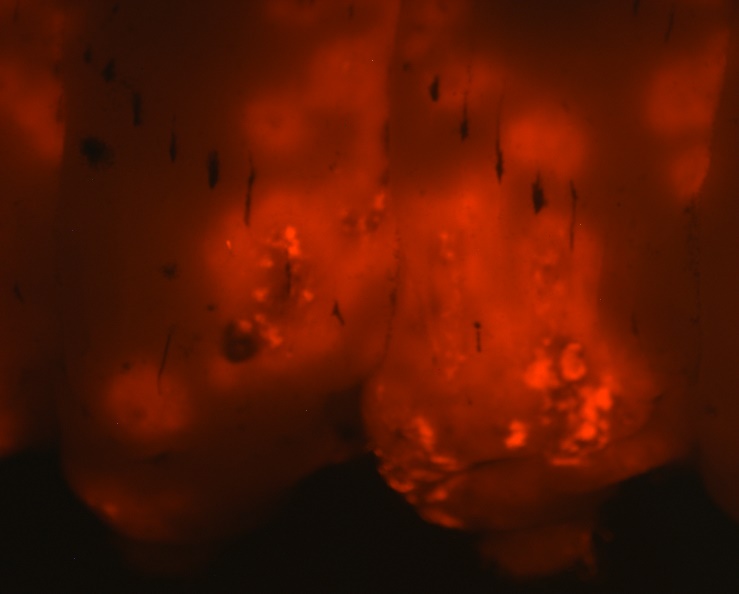

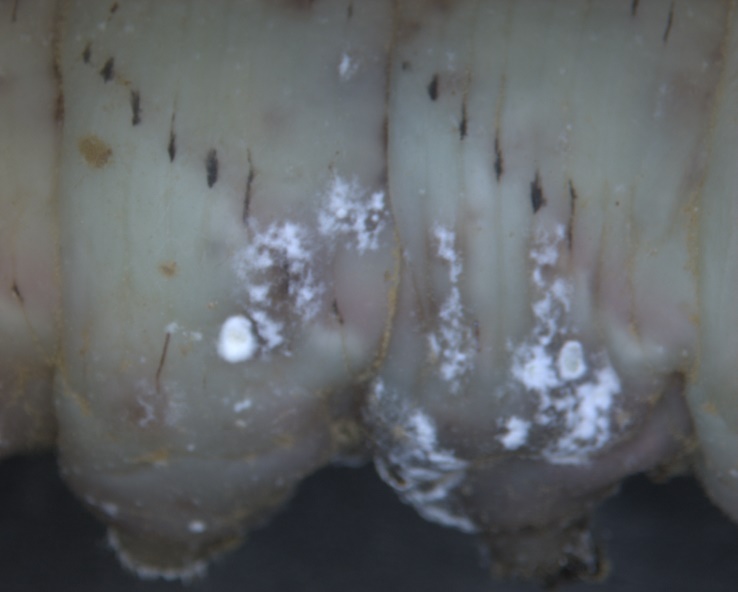

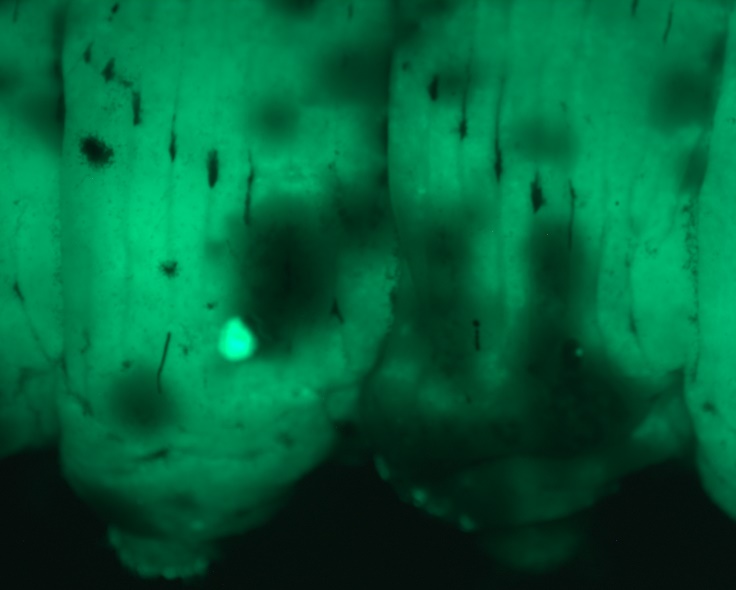

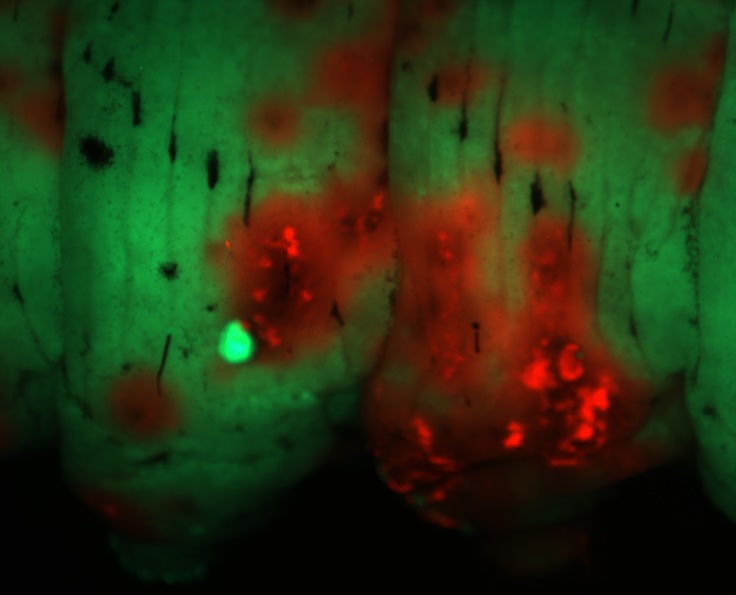


**A)**

**B)**

**C)**

**D)**

Pictures show a cadaver of *Manduca* infected by Ma549-GFP+Mr2575-Cherry approximately 24 hrs post-death. Bright field, GFP, cherry and overlay showing localization of Mr2575 to the front of segments


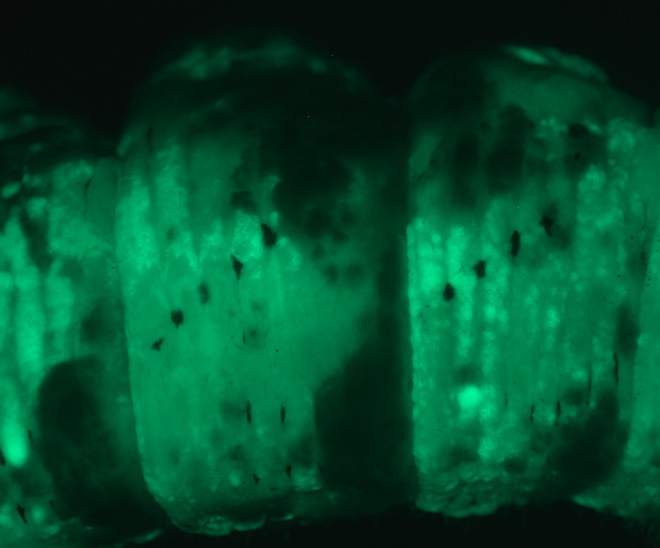

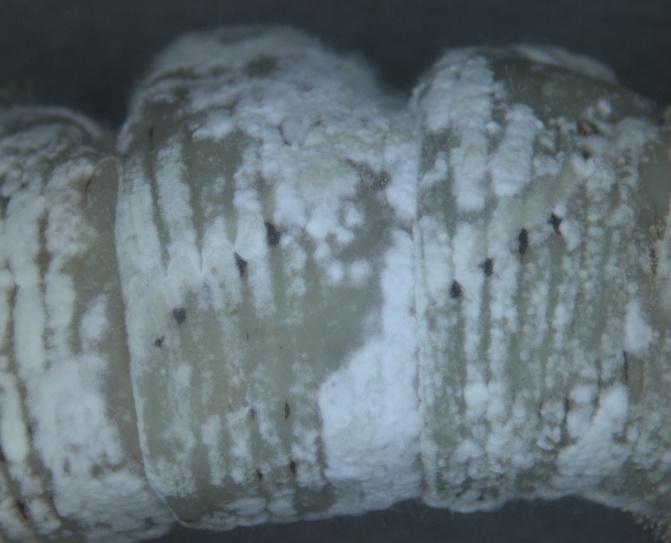

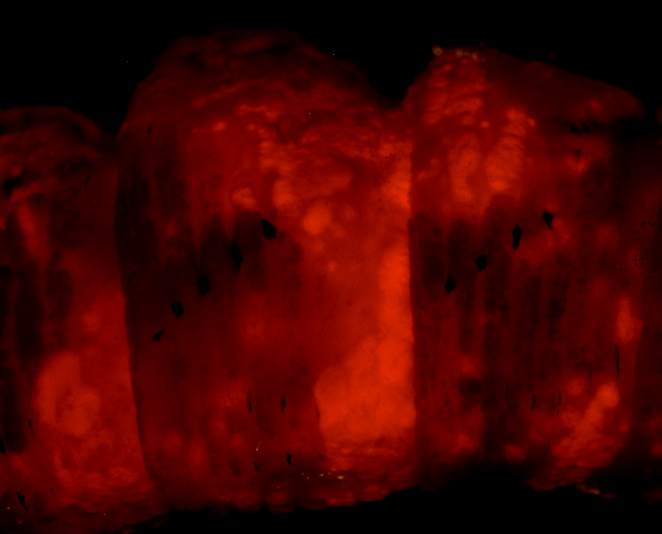

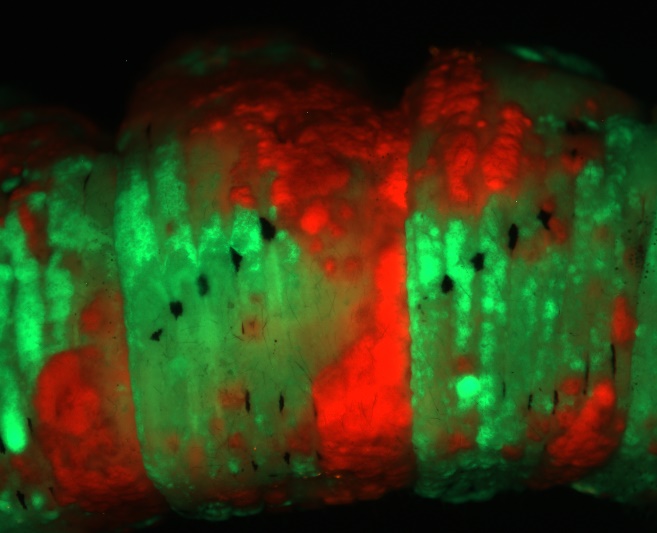


**A)**

**B)**

**C)**

**D)**

Pictures show a cadaver of *Manduca* infected by Ma549-GFP+Mr2575-Cherry approximately 24 hrs post-death. Bright field, GFP, cherry and overlay showing localization of Mr2575 to the front of segments and spiracles


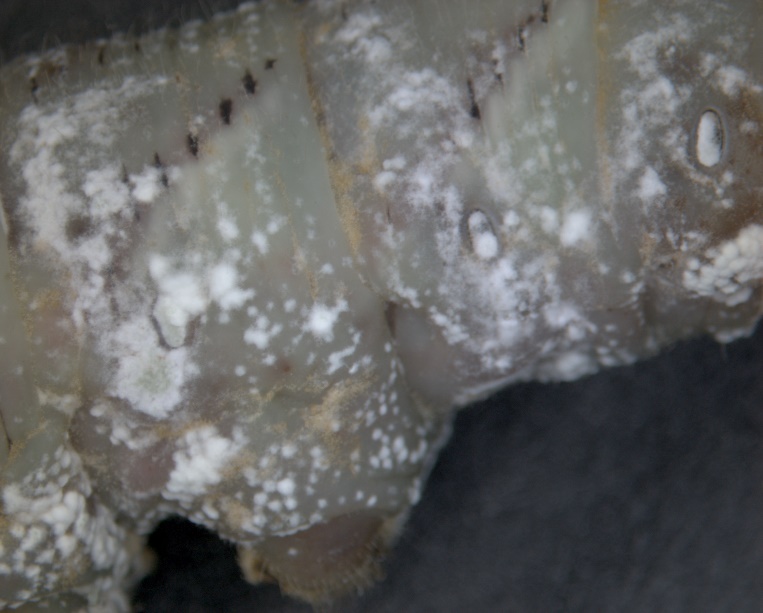

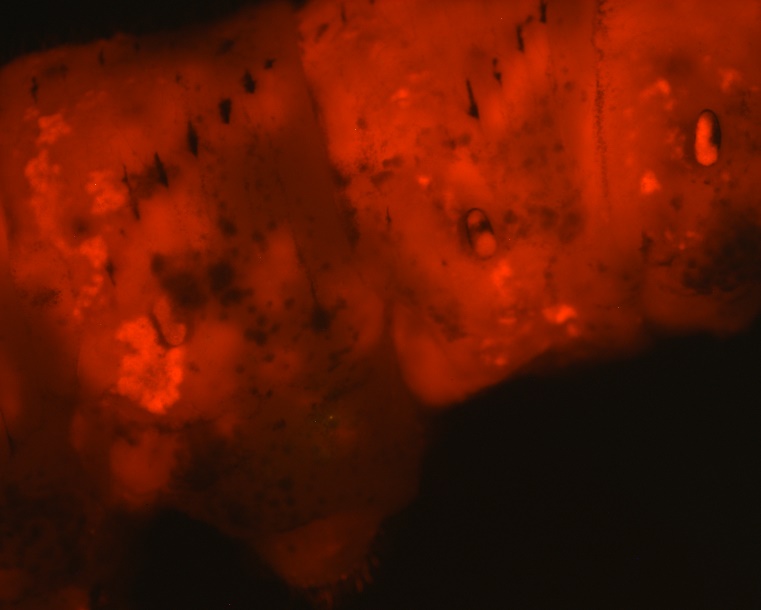

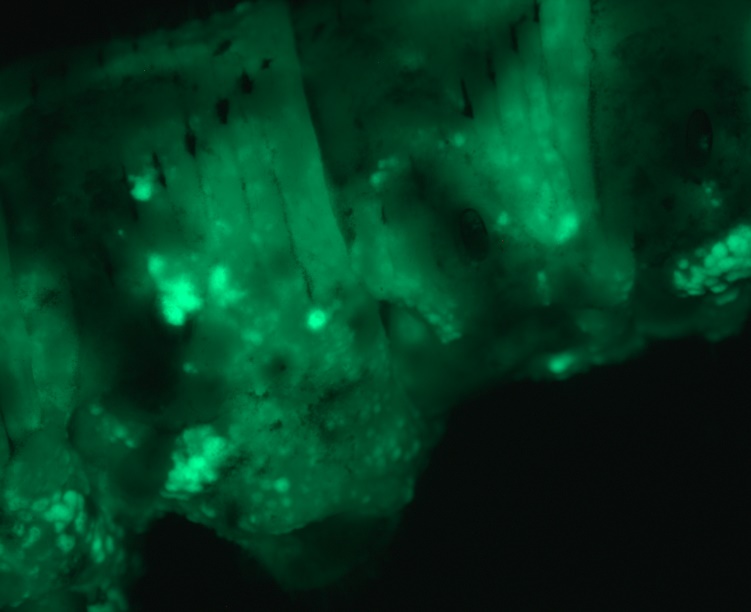

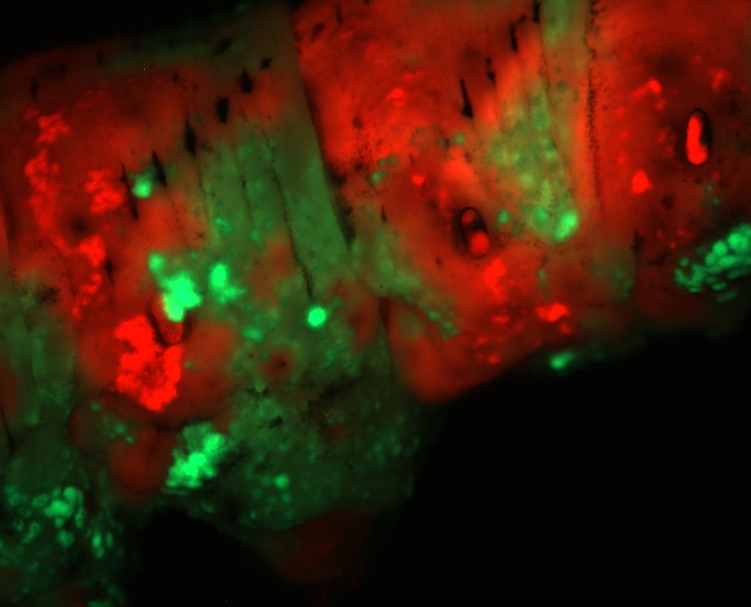


**A)**

**B)**

**C)**

**D)**

Pictures show a cadaver of *Manduca* infected by Ma549-GFP+Mr2575-Cherry approximately 24 hrs post-death. Bright field, GFP, cherry and overlay showing localization of Mr2575 to melanized areas particularly near the front of segments and spiracles


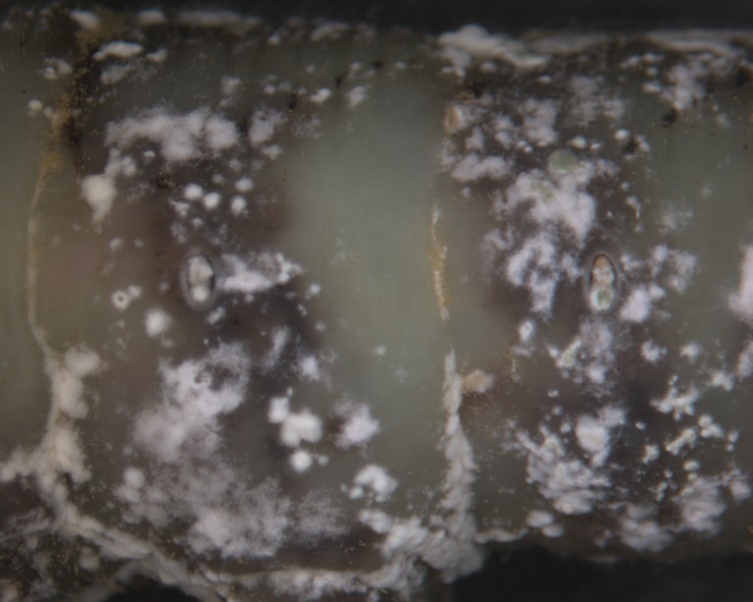

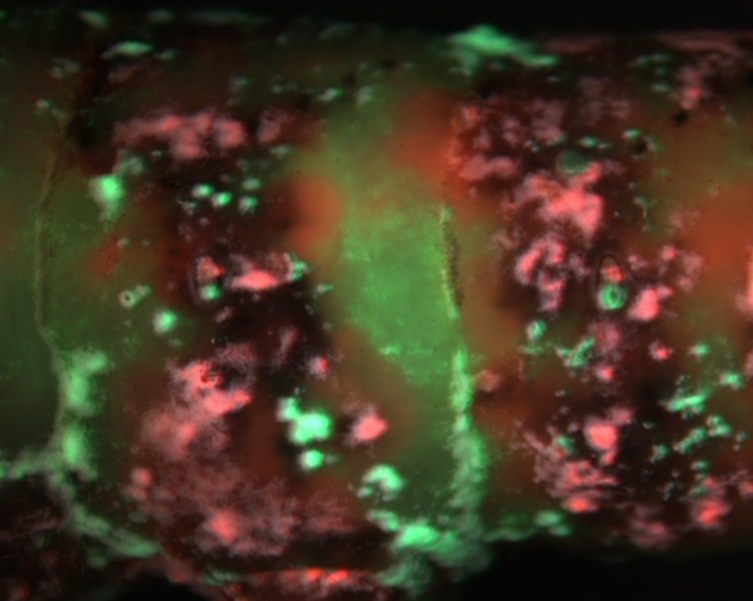

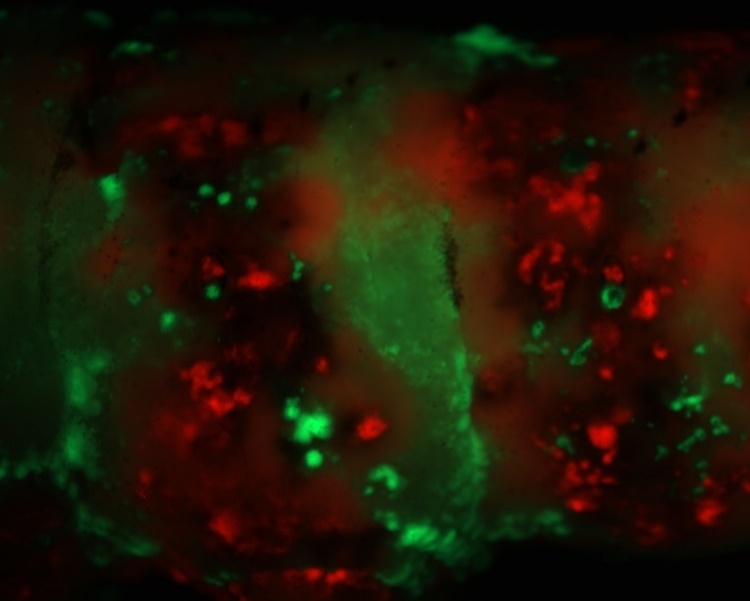

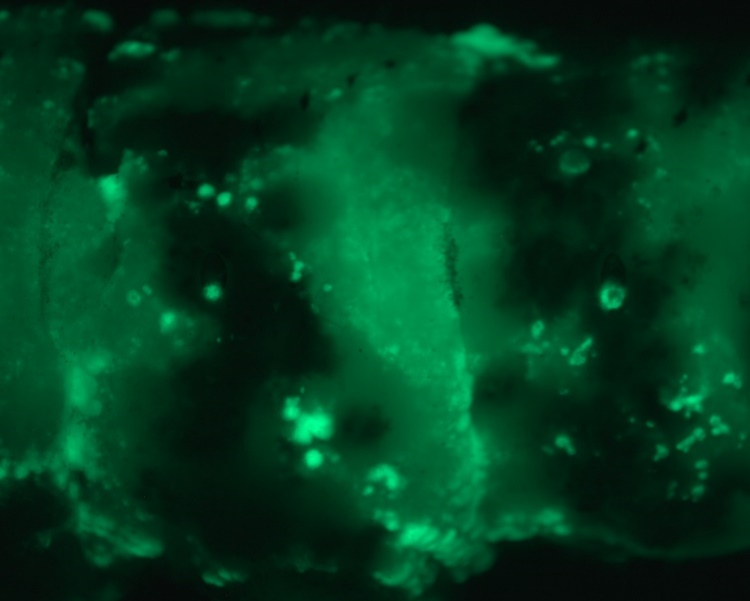

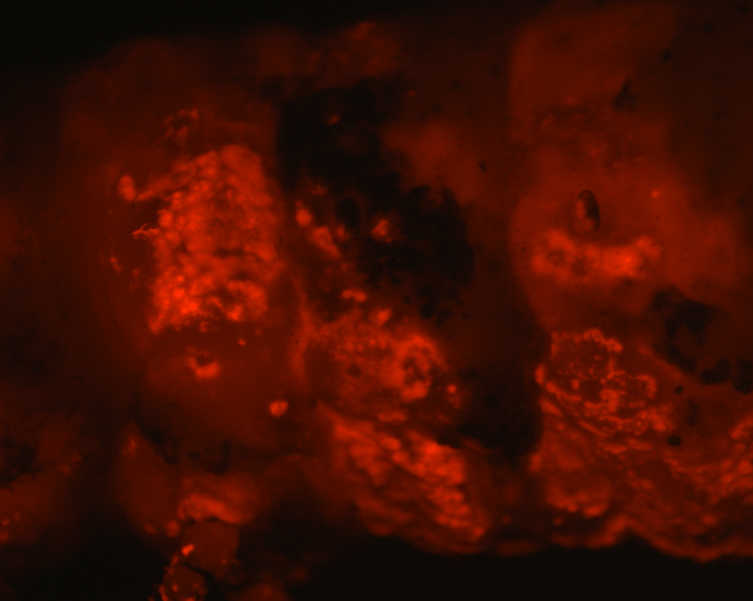


**A)**

**E)**

**C)**

**B)**

**D)**

Pictures show a cadaver of *Manduca* infected by Ma549-GFP+Mr2575-Cherry approximately 24 hrs post-death. Bright field, GFP, cherry and overlay showing localization of Mr2575 to the front of segments and spiracles


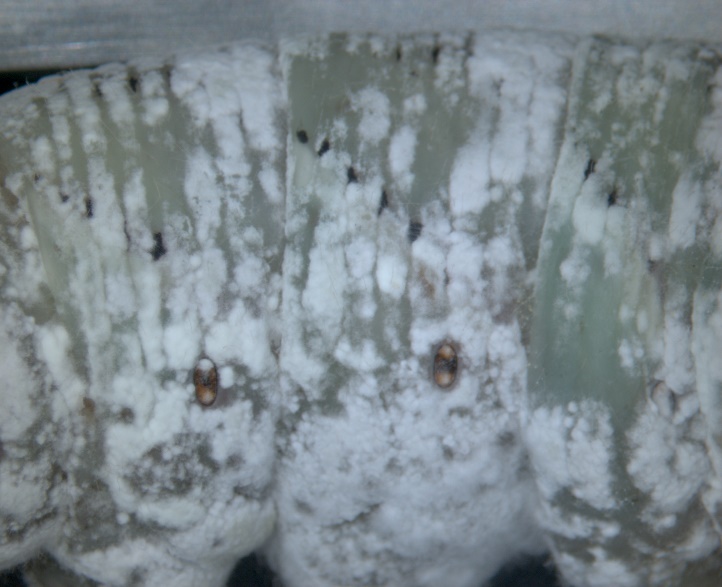

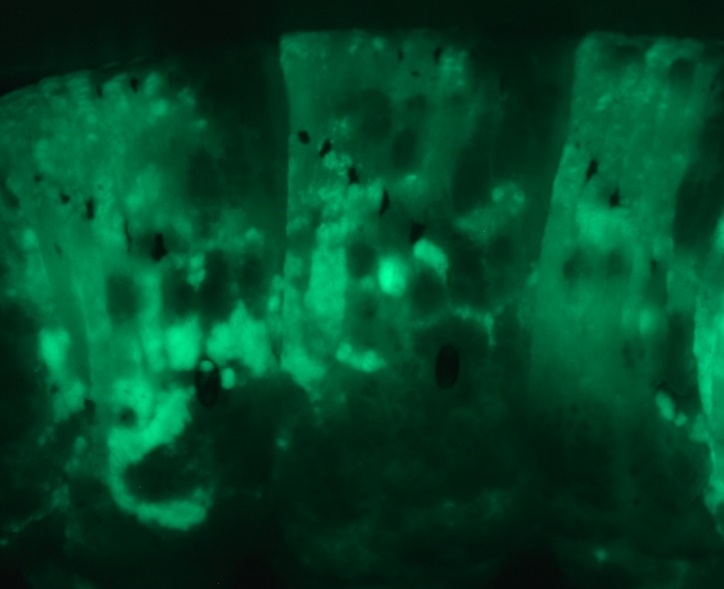

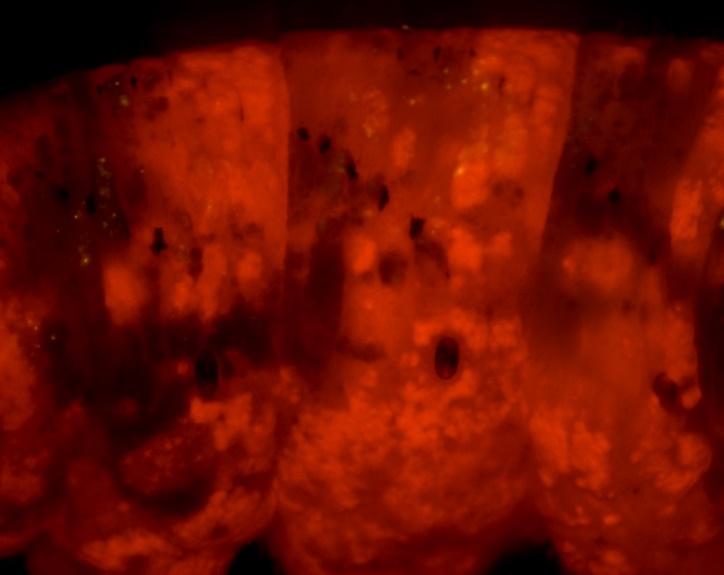

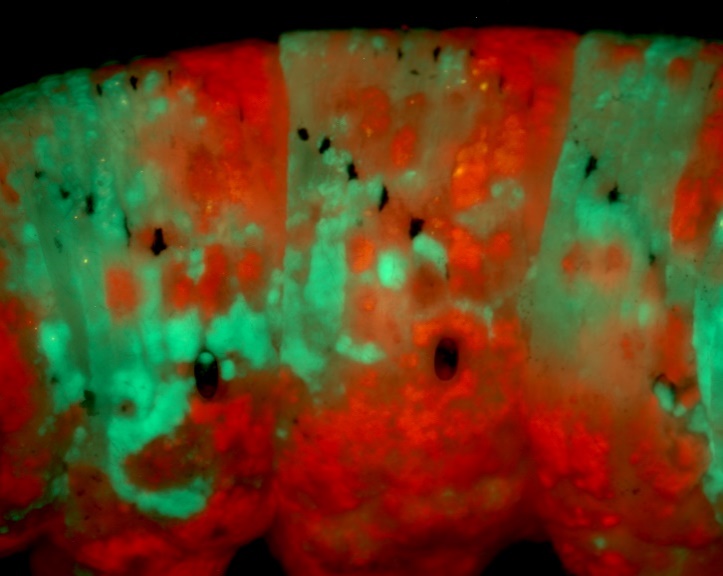


**A)**

**B)**

**C)**

**D)**
